# Photo-induced catalytic halopyridylation of alkenes

**DOI:** 10.1038/s41467-021-26857-w

**Published:** 2021-11-11

**Authors:** Shi-Yu Guo, Fan Yang, Ting-Ting Song, Yu-Qing Guan, Xiang-Ting Min, Ding-Wei Ji, Yan-Cheng Hu, Qing-An Chen

**Affiliations:** 1 Dalian Institute of Chemical Physics, Chinese Academy of Sciences, 457 Zhongshan Road, 116023 Dalian, China; 2 University of Chinese Academy of Sciences, 100049 Beijing, China

**Keywords:** Synthetic chemistry methodology, Photocatalysis

## Abstract

The Mizoroki-Heck reaction and its reductive analogue are staples of organic synthesis, but the ensuing products often lack a chemical handle for further transformation. Here we report an atom-economical cross-coupling of halopyridines and unactivated alkenes under photoredox catalysis to afford a series of alkene halopyridylation products. This protocol with mild and redox neutral conditions contributes broad substrate scope. As a complement to conventional Heck-type reaction, this radical process avoids the involvement of *β*-H elimination and thus useful pyridyl and halide groups could be simultaneously and regioselectively incorporated onto alkenes. The success depends on TFA-promoted domino photocatalytic oxidative quenching activation and radical-polar crossover pathway. Plausible mechanism is proposed based on mechanistic investigations. Moreover, the reserved C − X bonds of these products are beneficial for performing further synthetic elaborations.

## Abstract (web summary)

The Mizoroki–Heck reaction and its reductive analogue are staples of organic synthesis, but the ensuing products often lack a chemical handle for further transformation. Here the authors present a method to add a heterocycle and transformable halide across a double bond via iridium photocatalysis.

## Introduction

**Fig. 1:**
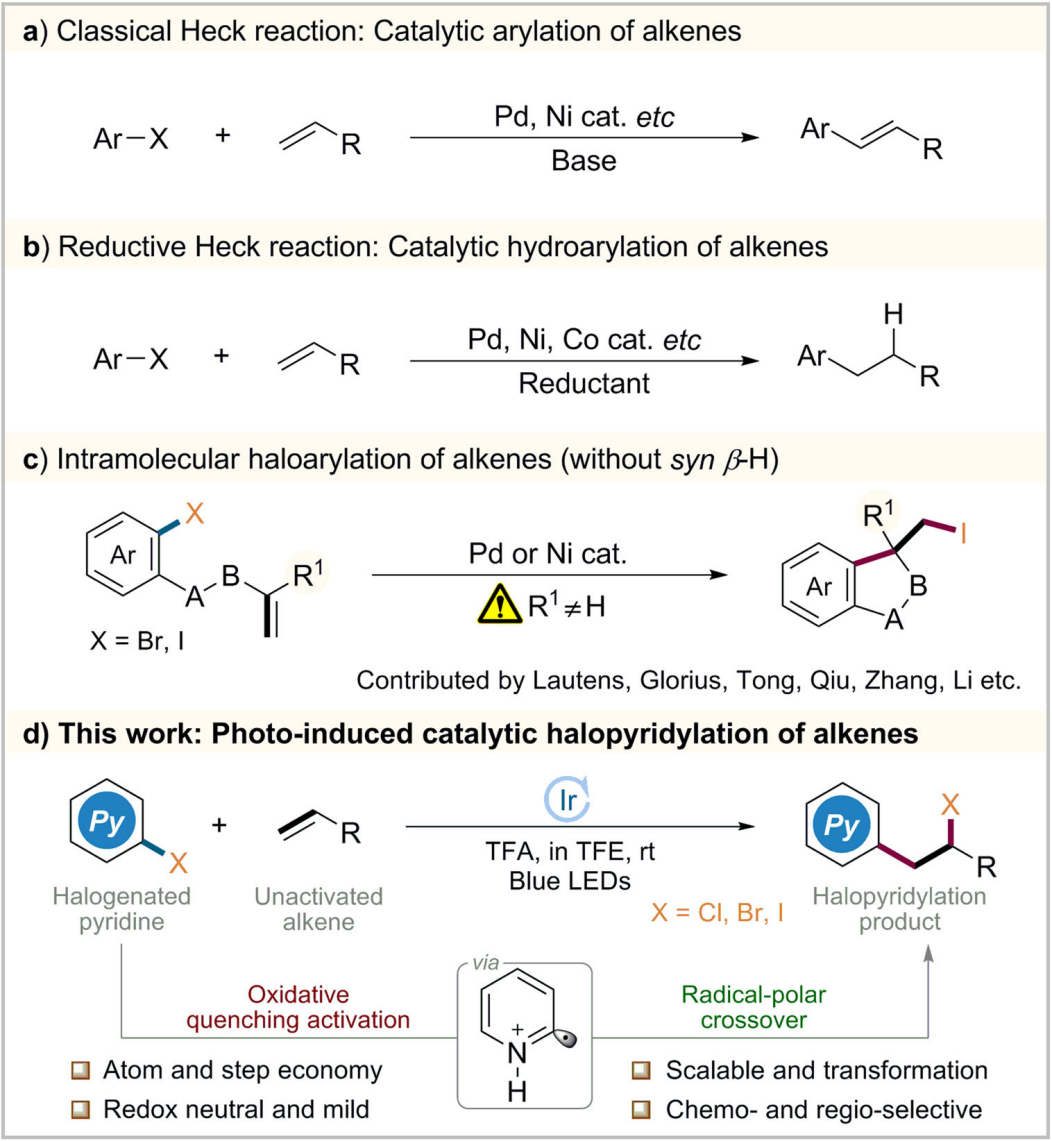
Cross-coupling of aryl halides with alkenes. **a** Classical Heck reaction. **b** Reductive Heck reaction. **c** Intramolecular halo alytic halopyridylation of alkenes. **d** Photo-induced catalytic halopyridylation of alkenes.

The rapid elaboration of molecular complexity from simple substrates has always been a fundamental theme in organic chemistry. Among those known methods, catalytic functionalizations of alkenes have gained much attention owing to the low cost and easy availability^1–9^. Of particular note is classical Heck cross-coupling that provides a rapid access to arylated alkenes (Fig. 1a)^10–14^. Besides, with an introduction of external hydride sources, a variety of reductive Heck-type products could be readily obtained via hydroarylation of alkenes (Fig. 1b)^15–18^. While they have wide applicability, both processes involve an elimination of halide atoms as inevitable waste.

On the other hand, the selective construction of C−X bond is of great significance as it provides an important synthetic handle for the incorporation of diverse functional groups^19–25^. In this context, it is appealing to exploit a complementary cross-coupling featuring installation of new C−C and C−X bonds onto alkenes simultaneously without sacrifice of any atoms. Following this concept, elegant intramolecular haloarylations of alkenes have been established by Lautens, Glorius, Tong etc^26–35^. Their success usually relies on the employment of terminal disubstituted alkenes that can lead to an alkyl Pd or Ni halide intermediate without a *syn β*-H atom (Fig. 1c). Besides, the haloarylation of ethylene and propylene with aryl chlorides normally requires carcinogenic high-energy ultraviolet (UV) light^36^. Despite these impressive advances, visible light-induced catalytic intermolecular haloarylation of wide simple alkenes with aryl halides still remains underexplored.

In recent years, photoredox catalysis^37–43^ represents the state-of-the-art technique to promote functionalizations of alkenes^44–48^. Owing to the ubiquitous role of pyridyl motifs as aromatic heterocycle in ligand scaffolds, natural products, and medically relevant molecules^49–53^, significant efforts have been devoted to visible light-driven pyridylation of alkenes^54–63^. Intrigued by these precedents and our continued interest on alkenes functionalizations^64–67^, we questioned whether it is possible to simultaneously install both pyridyl and halide groups onto alkenes which possess a potential syn *β*-H atom.

Herein, this goal has been achieved by coupling of unactivated alkenes and halopyridines under visible light condition (Fig. 1d). This halopyridylation transformation is enabled by domino photocatalytic oxidative quenching activation and radical-polar crossover pathway. It provides an important complement for known precedents about Heck reactions with respect to product diversity and atom economy.

## Results

### Reaction optimization

Initially, under blue light irradiation, 2-bromo-6-methylpyridine **1a** and 1-hexene **2a** were selected as model substrates to optimize the reaction (Table 1 and Supplementary Tables 1–8 in Supplementary Information). First, as state-of-the-art photocatalytic strategy of pyridyl radical generation, Jui’s elegant protocols^56,59^ were applied to these substrates. Both results clearly shown that there was no detectable bromopyridylation product **3aa** formed under Jui’s conditions (Table 1, entries 1–2). Only a trace amount of product **3aa** could be detected in the absence of HEH (Hantzsch ester) (entries 3–4). Based on these results, Jui’s reductive quenching activation of halopyridine conditions were not suitable for the desired C–X bond formation. According to general principle for the construction of C–X bond, we envisioned that oxidative quenching activation of halopyridine would divert the reactivity from hydropyridylation towards halopyridylation of alkenes.

**Table 1:** Optimization of reaction conditions^a^.

Entry	State-of-the-art photocatalytic methods	Yield^b^ [%]
1	Boyington et al.^56^ (reductive quenching activation) [Ir-PC A (1 mol%), HEH (1.3 eq.), NH_4_Cl (2.0 eq.)]	N. D.
2	Seath et al.^59^ (reductive quenching activation) [Ir-PC A (1 mol%), HEH (1.3 eq.), CySH (5 mol%)]	N. D.
3	Boyington et al.^56^ without HEH [Ir-PC A (1 mol%), NH_4_Cl (2.0 eq.)]	N. D.
4	Seath et al.^59^ without HEH [Ir-PC A (1 mol%), CySH (5 mol%)]	Trace
5	Standard condition (oxidative quenching activation) [Ir-PC A (1 mol%), TFA (1.0 eq.)]	89

Entry	Variation from standard condition	Yield^b^ [%]
6	No Ir-PC A	0
7	In dark	0
8	Ir-PC B, Ir-PC C, or Ir-PC D as catalyst	0, 75, 0
9	Eosin Y or (Acr-Mes)ClO_4_ as catalyst	0, 0
10	No TFA	Trace
11	AcOH, (PhO)_2_PO_2_H, or PhSO_3_H instead of TFA	0, 76, 80
12	EtOH or HFIP instead of TFE	Trace, 86
13	Oil bath at 80 °C in dark	0
14	Air instead of N_2_	Trace


*PC* photocatalyst.

^a^Conditions: **1a** (0.20 mmol), **2a** (0.60 mmol), Ir-PC A (1.0 mol%), TFA (0.20 mmol), TFE (2.0 mL), blue LEDs (*λ*_max_ = 456 nm, 40 W), room temperature, 16 h.

^b^Determined by GC-FID analysis of the crude reaction mixture using mesitylene as internal standard.

After careful evaluation for oxidative quenching activation of bromopyridine **1a**, the desired bromopyridylation product **3aa** was furnished in 89% yield using [Ir(ppy)_2_(dtbbpy)]PF_6_ (Ir-PC A) as photocatalyst and TFA (Trifluoroacetic acid) as additive (Table 1, entry 5). No target product was obtained in the absence of photocatalyst or in dark environment (entries 6 and 7). Strong electron-deficient photocatalyst (Ir-PC B) and tris(2-phenylpyridine)iridium (Ir-PC D) showed no catalytic performance, while 4-fluorophenylpyridine derived catalyst (Ir-PC C) gave a slightly decreased yield (entry 8). Other commonly used organophotocatalysts such as Eosin Y and (Acr-Mes)ClO_4_ were also examined but found to be ineffective (entry 9). Moreover, the Brønsted acid additive was essential for the process and the acidity also exerted an important influence on the outcomes (entries 10 and 11). For instance, weak acid AcOH could not promote the reaction, whereas (PhO)_2_POOH and PhSO_3_H delivered the target product **3aa** in 76% and 80% yields, respectively (entry 11). Strongly polar protic solvent, such as TFE (Trifluoroethanol) and hexafluoroisopropanol (HFIP) were both competent solvents for this reaction, but EtOH was not feasible (entry 12). The reaction could not take place under thermal conditions or air atmosphere (entries 13 and 14).

### Substrate scope

**Fig. 2:**
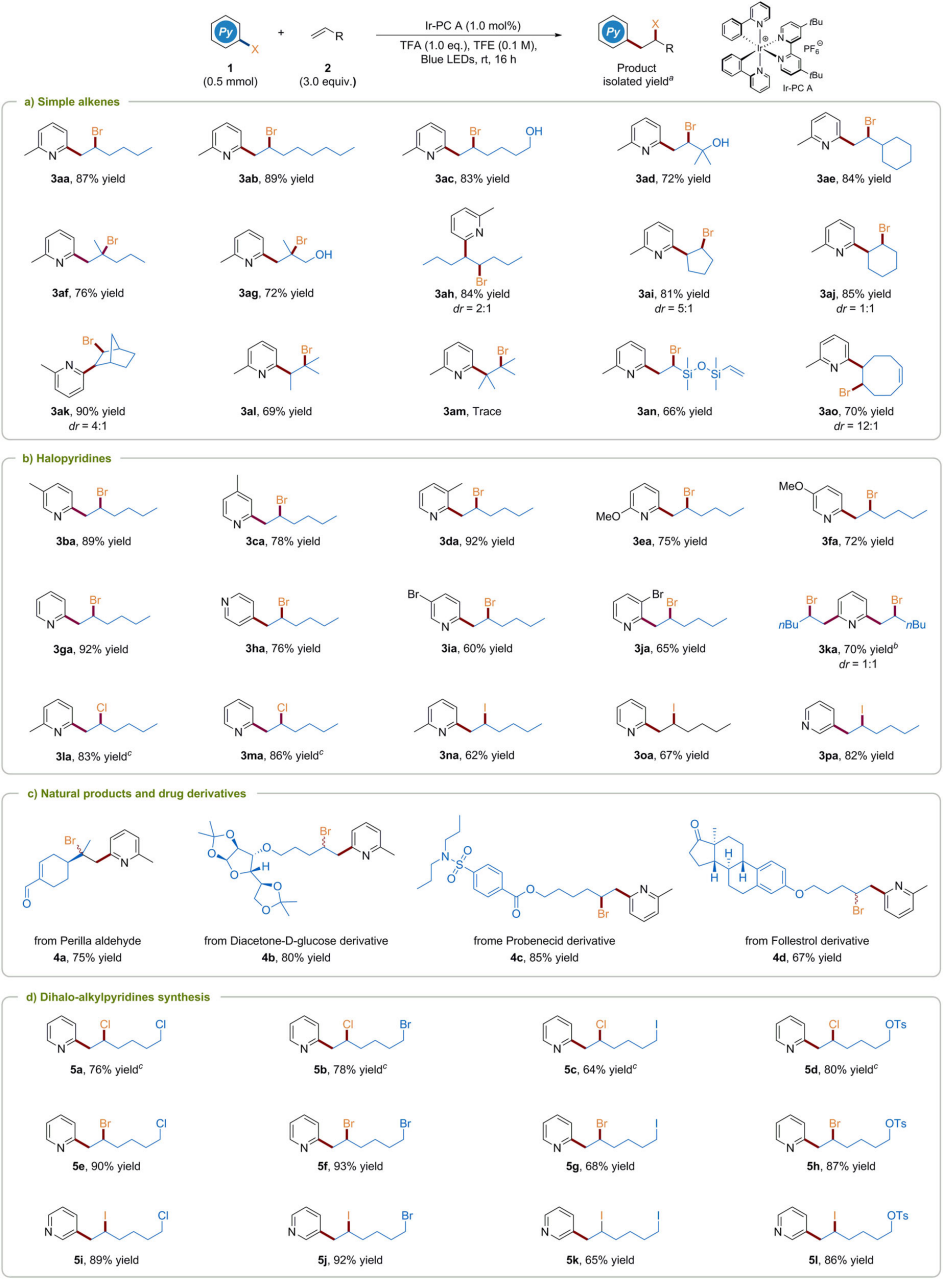
Substrate scope. **a** Simple alkenes scope. **b** Halopyridines scope. **c** Natural products and drug derivatives. **d** Dihalo-alkylpyridines synthesis. ^a^Yields of isolated products. Diastereoselectivity (*dr*) was determined by ^1^H NMR analysis. ^b^4.0 equivalents of **2a** was used. ^c^20 h. PC photocatalyst.

Having identified optimized conditions, the substrate scope with respect to alkenes was examined (Fig. 2a). To our delight, a number of terminal alkenes, including terminal *α*-alkenes (**3aa**-**3ae**) and 1,1-disubstituted alkenes (**3af**, **3ag**) could be successfully bromopyridylated under this protocol. Notably, alkenes with free hydroxyl group were suitable coupling partners as well, affording desired products in moderate to good yields (**3ac**, **3ad**, and **3ag**). In addition to terminal alkenes, this protocol could be extended to internal alkenes (**3ah**-**3al**). For example, bromopyridylation of *trans*-4-octene delivered the expected product **3ah** in 84% yield with 2:1 dr. A variety of cyclic internal alkenes, including cyclopentene, cyclohexene, and norbornene were also well tolerated to furnish the corresponding products in 81–90% yields (**3ai**-**3ak**). Gratifyingly, the treatment of trisubstituted alkene with standard conditions resulted in the generation of single product **3al** in 69% yield. The exclusive regioselectivity is presumably ascribed to the favorable pyridyl radical addition at the less hindered alkene carbon, which could also lead to a more stable tertiary carbon radical or carbocation. Unsurprisingly, only trace amount of bromopyridylated product **3am** was observed for the coupling with tetrasubstituted alkene. When non-conjugated dienes were subjected to this reaction conditions, one carbon–carbon double bond could be selectively reserved for potential product diversity (**3an** and **3ao**).

Subsequently, we turned our attention to the investigation on the scope of halopyridines using 1-hexene as the model alkene. As shown in Fig. 2b, a broad range of bromopyridines, including alkyl-substituted (**3ba**-**3fa**), unsubstituted (**3ga**, **3ha**), and disubstituted bromopyridines (**3ia**-**3ka**) proved to be smoothly accommodated to this protocol. The position of alkyl substituents (methyl and alkoxy) in bromopyridines had no great influence on the yields of products (**3ba**-**3fa**). Simple 2- and 4-bromopyridines were also amenable to the transformation, but the bromine substituent at 2-position seemed to be more reactive (**3ga**, **3ha**). Notably, in the cases of 2,3- and 2,5-dibromopyridines, the reaction selectively took place at the 2-position with the preservation of 5- or 3-bromo group for further synthetic elaboration (**3ia** and **3ja**). In comparison, 2,6-dibromopyridine could react with two molecules of 1-hexene to produce **3ka** in 70% yield. Moreover, this protocol could also be extended to a variety of chloro and iodopyridines (**3la-3pa**) which might be great challenges for traditional metal catalysis in terms of substrate activation or product overreaction. However, bromopyridine substrates bearing strong electron withdrawing groups were not suitable for this reaction. It probably resulted from the protonation of electron-deficient pyridines with TFA may not be favored, which would be detrimental to product formation (Supplementary Table 9 in Supplementary Information).

To magnify the potential merit of current photocatalytic protocol, we assessed the late-stage halopyridylation of several complex scaffolds based on natural products and drug molecules, as illustrated in Fig. 2c. Specifically, terpenes substrate perilla aldehyde was feasibly transformed into the corresponding bromopyridylation product **4a** in 75% yield and the unique chemoselectivity for terminal alkene over internal alkene was displayed. In addition, complex molecules containing diverse structural features, such as carbohydrate (**4b**), sulfonamide (**4c**), and steroid (**4d**) under the standard conditions were also tested to effectively deliver corresponding products in 67–85% yields, indicating that this method could give rapidly access to a wide range of valuable halide and pyridyl containing scaffolds.

Additionally, owing to the versatile role of organohalides, we further explored the reactions between different halopyridines and halogenated alkenes to synthesize a series of dihalogenated alkylpyridine compounds (Fig. 2d). To our delight, (pseudo)halo-substituted (Cl-, Br-, I-, and TsO-) alkenes could undergo halopyridylation smoothly to afford the dihalogenated alkylpyridine products **5a**-**5l** in moderate to good yields. These results above adequately exemplify the mild nature of the photocatalytic system by bearing the high tolerance of various functional groups and demonstrate the attractive synthetic utility of this halopyridylation of unactivated alkenes method.

### Mechanistic investigations

**Fig. 3:**
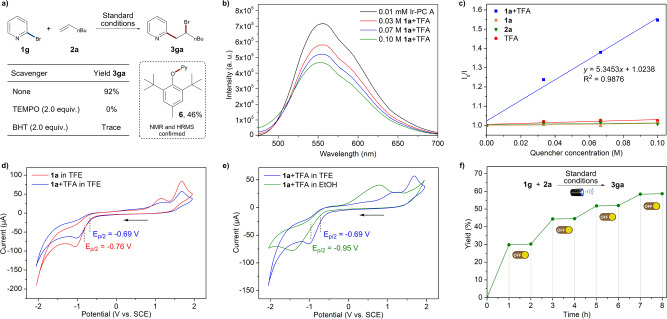
Mechanistic investigations. **a** Radical trapping experiments. **b** Fluorescence quenching experiments of Ir-PC A with various concentrations of **1a** + TFA in TFE solution. **c** Stern−Volmer plots of Ir-PC A with different quenchers. **d** Cyclic voltammograms of **1a** and **1a** + TFA in 0.1 M TBAPF_6_ TFE solution. **e** Cyclic voltammograms of **1a** + TFA in 0.1 M TBAPF_6_ TFE solution and EtOH solution. **f** Light on/off experiments.

**Fig. 4:**
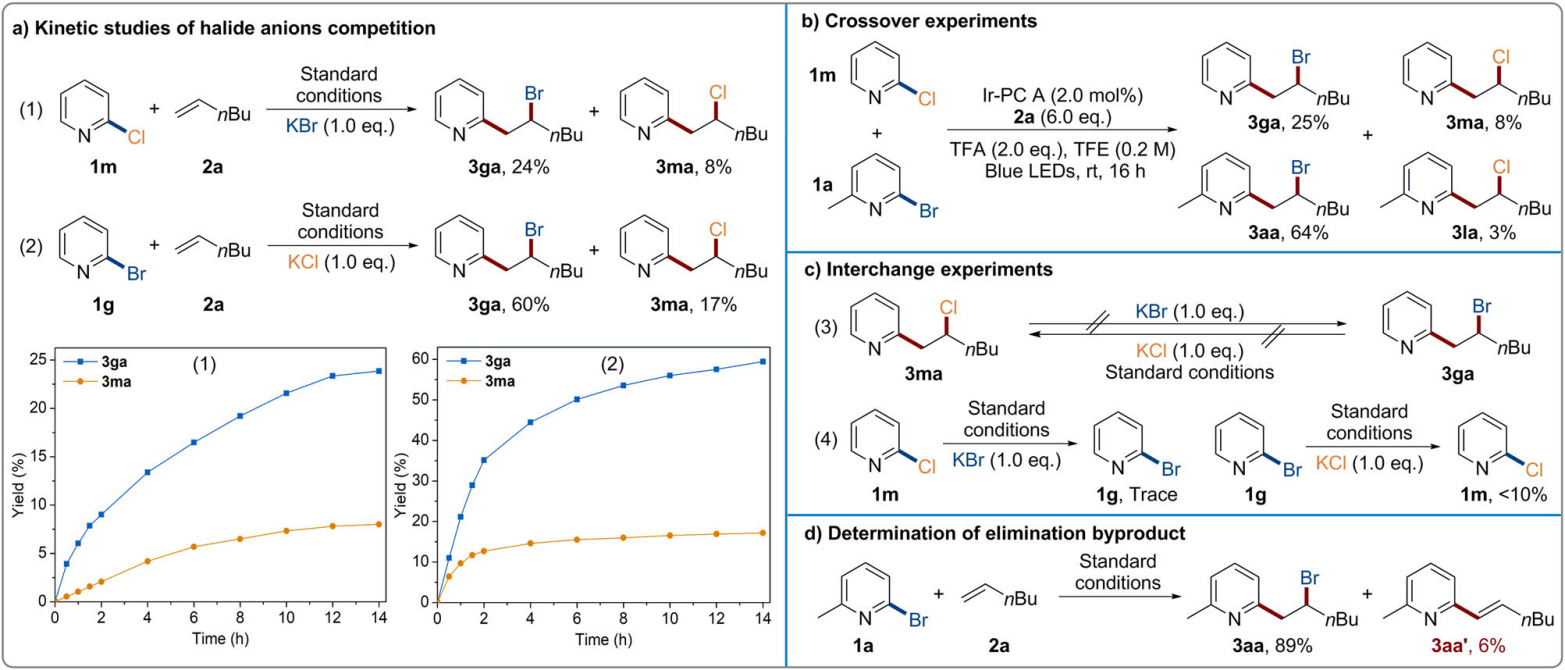
Halide anions competition experiments. **a** Kinetic studies of halide anions competition. **b** Crossover experiments. **c** Interchange experiments. **d** Determination of elimination byproduct.

To probe the mechanism of this photo-induced alkenes halopyridylation, preliminary mechanistic investigations have been conducted (Figs. 3 and 4). The addition of radical scavengers TEMPO (2,2,6,6-Tetramethylpiperidinooxy) or BHT (Butylated Hydroxytoluene) to the reaction mixture almost inhibited this reaction (Fig. 3a).The observation of BHT-pyridyl adduct **6** suggests that the pyridyl radical generation is probably involved in this transformation. Stern−Volmer fluorescence quenching experiments were performed on all reactants of **1a**, **2a**, TFA, and **1a**+TFA mixture, respectively (Fig. 3b, c and Supplementary Figs. 3–6 in Supplementary Information). The results clearly shown that only the combination of **1a**+TFA could dramatically quenched the excited state Ir-PC A* (Fig. 3b, c), which indicates an oxidative quenching activation and the protonated pyridine as the effective quencher in the photocatalytic quenching cycle.

In order to get insight into photoredox process, cyclic voltammetry experiments were further analyzed. As shown in Fig. 3d, a sharp reduction peak of **1a** with *E*_1/2_^red^ = −0.76 V (vs. SCE) was observed in the cathodic reduction scan in TFE, and the **1a**+TFA mixture (1:1 molar ratio) could rise to *E*_1/2_^red^ = -0.69 V (vs. SCE). Moreover, when the solvent was changed to EtOH, reduction potential of the **1a**+TFA mixture fell to *E*_1/2_^red^ = −0.95 V (vs. SCE) (Fig. 3e). These results suggest that the halopyridine substrate is most likely protonated first by the Brønsted acid TFA and gets stronger oxidizing properties. Light on/off control experiments were performed to obtain the reactivity profile of this protocol (Fig. 3f). It is found that the product generation was blocked immediately when the light was turned off and resumed efficiently when turned on, indicating that constant light irradiation is essential for this transformation. The resultant long radical chain progress may be unlikely, considering a relatively low quantum yield (Φ = 0.685, Page S29 in Supplementary Information).

On the other hand, halide anions competition experiments were carried out to verify the radical-polar crossover pathway to give ATRA-type products (Fig. 4). When 2-Cl-pyridine **1** **m** and 1-hexene **2a** were subjected to this standard conditions in the presence of KBr (1.0 equiv.), the expected chloride product **3ma** and crossover bromide product **3ga** were isolated in 8% and 24% yields, respectively (Fig. 4a, eq 1). Based on the kinetic studies results, the formation of crossover bromide product **3ga** dominated during the whole process (Fig. 4a, chart 1). When 2-Br-pyridine **1** **g** reacted with **2a** in the presence of KCl (1.0 equiv.), similar but higher yields for halopyridylation products were obtained (60% **3ga** and 17% **3ma**) (Fig. 4a, eq 2). With the help of these kinetic results, the higher reactivity probably resulted from the easier generation of pyridyl radical from 2-Br-pyridine **1g** than 2-Cl-pyridine **1** **m** (eq 2 vs. eq 1). Additionally, in the absence of extra potassium halide salt, bromide products (**3ga** and **3aa**) also dominated over chloride derivatives (**3ma** and **3la**) in the crossover reaction between 2-Cl-pyridine **1m** and 2-Br-6-Me-pyridine **1a** (Fig. 4b). In general, bromide anion exhibited weaker nucleophilicity than chloride one due to its weaker negative charge density. However, owing to a strong hydrogen bonding interaction in polar protic solvent TFE, the nucleophilic ability was switched and bromide anion turned to be a better nucleophile. Besides, the resubjection of halide product **3ma** and **3ga** to the standard conditions delivered no detectable halide exchange products (Fig. 4c, eq 3). This negative halide interchange result indicates that the current photo-induced protocol will not cleavage alkyl carbon-halide bond which will be common issues for transition metal catalysis. Interestingly, the halogen exchange of halopyridines could slightly occur in the presence of extra potassium halide salt (Fig. 4c, eq 4). Moreover, Heck-type byproduct **3aa’** was obtain in 6% yield under optimized reaction condition, which was formed via *β*-H elimination of carbon cation intermediate. This suggests that a radical-polar crossover mechanism via alkyl carbocation intermediate is operative for the C-X bond formation (Fig. 4d).

**Fig. 5:**
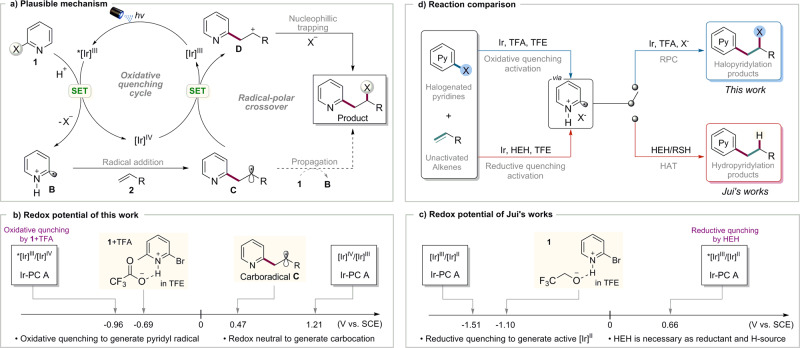
Proposed mechanism and comparison. **a** Plausible mechanism. **b** Redox potential of this work. **c** Redox potential of Jui’s works. **d** Reaction comparison.

Based on the experimental results above and literatures, a possible mechanism of this photo-induced catalytic halopyridylation of alkenes is proposed in Fig. 5a. Initially, irradiation of [Ir]^III^ gives rise to its excited state *[Ir]^III^. Then halopyridine **1** is protonated in the presence of TFA in TFE and reduced by *[Ir]^III^ to generate pyridyl cation radical **B**, along with [Ir]^IV^ and halide anion. The subsequent electrophilic addition of protonated pyridyl radical **B** to nucleophilic alkene **2** produces the secondary alkyl radical **C**. Alkyl radical **C** undergoes polarity-matched SET with [Ir]^IV^ to give carbocation **D**^68,69^, and regenerates the [Ir]^III^ photocatalyst. The resulting carbocation **D** is then trapped by halide anion to produce the final ATRA-type product. An alternative radical-chain propagation cannot be excluded, but is probably not a favored pathway.

Redox potential analysis has been posted to explain the differences between this protocol and Jui’s works^56,59^ in mechanism (Fig. 5b, c). In this work, the desired pyridyl radical is generated via an oxidative quenching activation. The protonated halopyridyl trifluoroacetate possessing higher reduction potential (*E*_1/2_^red^ = -0.69 V) is facial to be reduced by excited state *[Ir]^III^ [*E*_1/2_(*Ir^III^/Ir^IV^) = −0.96 V]. After radical addition, redox potential of the alkyl radical **C** (*E*_1/2_^Oxi^ = 0.47 V)^70^ and [Ir]^IV^ [*E*_1/2_ (Ir^IV^/Ir^III^) = 1.21 V] matches each other, occurring SET to produce carbocation **D** and regenerate [Ir]^III^. Contrarily, Jui’s works utilized the reductive quenching by HEH to give [Ir]^II^ [*E*_1/2_(Ir^II^/Ir^III^) = −1.51 V]. Then pyridyl radical was generated from the reductive activation of halopyridines (*E*_1/2_^red^ = −1.1 V). So hantzsch ester (HEH) is indispensable in Jui’s conditions for the reductive quenching cycle which is obstacle for the halopyridylation of alkenes (Fig. 5d).

**Fig. 6:**
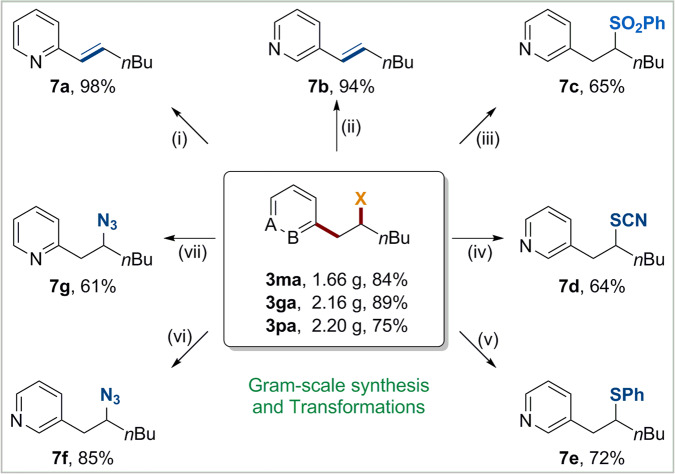
Synthetic transformations. (**i**) **3ga**, K_2_CO_3_, DMF, 80 °C, 6 h. (**ii**) **3pa**, K_2_CO_3_, DMF, 80 °C, 6 h. (**iii**) **3pa**, NaSO_2_Ph, DMF, 60 °C, 3 h. (**iv**) **3pa**, NaSCN, Na_2_CO_3_, MeCN, 40 °C, 3 h. (**v**) **3pa**, thiophenol, Na_2_CO_3_, MeCN, 40 °C, 7 h. (**vi**) **3pa**, NaN_3_, DMF, 60 °C, 10 h. (**vii**) **3ga**, NaN_3_, DMF, 60 °C, 10 h.

Finally, gram-scale reactions and further transformations of this protocol were conducted to demonstrate the synthetic utility. As shown in Fig. 6, 84%, 89%, and 75% yields of chloro-, bromo-, and iodo-pyridylation products could be isolated respectively under standard conditions (**3ma**, **3ga**, and **3pa**). Furthermore, the C−X bonds of these products are beneficial for performing further synthetic elaborations. It have been showed that the halopyridination products **3ga** and **3ma** could undergo many useful transformations, such elimination (**7a** and **7b**), and substitution by sulfinate (**7c**), thiocyanate (**7d**), thiophenol (**7e**), and azidation (**7** **f** and **7** **g**) in moderate to good yields (Fig. 6).

## Discussion

In this work, we have developed a photo-induced catalytic halopyridylation of unactivated alkenes. Using halogenated pyridines as dual pyridyl and halogen source, new C−C bond and C−X bond are simultaneously and regioselectively constructed under mild conditions. It also features an excellent atom and step economy for the late-stage halopyridylation of complex molecules. Tackling the challenging issue from *β*-H elimination and alkyl C−X cleavage, this photo-induced catalysis complements traditional transition metal catalysis in terms of mechanism and product diversity. Moreover, this protocol has good synthetic utility for scale-up preparation, and the C−X bonds of these halopyridination products can serve as versatile handle for further transformations.

## Methods

### General procedure for photo-induced catalytic halopyridylation of alkenes

To an oven-dried 4 mL vial was added Ir(ppy)_2_(dtbbpy)PF_6_ (0.005 mmol, 1.0 mol%), halopyridines **1** (0.5 mmol, 1.0 equiv.), alkenes **2** (1.5 mmol, 3.0 equiv.), TFA (0.5 mmol, 1.0 equiv.), and TFE (2.5 mL, 0.2 M) in the nitrogen glove box. The vial was capped with a septum and wrapped with parafilm. The reaction mixture was stirred for 16 h under visible light irradiation (1 × Kessil PR160, *λ*_max_ = 456 nm, 40 W, irradiation temperature maintained between 25 and 30 °C). Upon completion, the crude product was neutralized with saturated NaHCO_3_ solution or Et_3_N and extracted with ethyl acetate. Organic layer was washed with brine solution and dried over anhydrous Na_2_SO_4_. Removal of the organic solvent in a vacuum rotavapor followed by flash silica gel column chromatographic purification to afford the desired products in moderate to good yields.

## Supplementary information

Supplementary Information

## Data Availability

The authors declare that the data supporting the findings of this study, including experimental details and compound characterization, are available within the article and its Supplementary Information files. All data are available from the corresponding author upon request.
