# Supplementary material for: Photo-induced catalytic halopyridylation of alkenes
Source: Nat Commun. 2021 Nov 11;12:6538. doi: 10.1038/s41467-021-26857-w (PMC8586348; doi:10.1038/s41467-021-26857-w)
Supplement: Supplementary file 1 — Supplementary Information [file 41467_2021_26857_MOESM1_ESM.pdf]

Supplementary Information

Photo-Induced Catalytic Halopyridylation of Alkenes

Shi-Yu Guo,[†] Fan Yang,[†] Ting-Ting Song,[†] Yu-Qing Guan,[†] Xiang-Ting Min,[†] Ding-Wei Ji,[†] Yan-Cheng Hu,[†] and Qing-An Chen[†,‡*]

[†]*Dalian Institute of Chemical Physics, Chinese Academy of Sciences,*
457 Zhongshan Road, Dalian 116023, China
[‡]*University of Chinese Academy of Sciences, Beijing 100049, China*
E-mail: qachen@dicp.ac.cn

Table of Contents

1. Supplementary Note 1 .... S2

2. Supplementary Note 2 .... S2

2.1. Optimization studies of reaction conditions S2

2.2. Synthesis of complex alkene precursors S6

2.3. Unsuccessful halopyridines with strong electron withdrawing substituent S7

2.4. General procedure for photo-induced catalytic halopyridylation of alkenes S8

2.5. Analytical data for the halopyridylation products S8

2.6. Gram-scale reactions S19

2.7. Further transformations S19

2.8. Mechanistic studies S23

2.9. Copies of NMR spectra of products S33

3. Supplementary References S94

1. Supplementary Note 1

Commercially available reagents were used without further purification. Solvents were treated prior to use according to the standard methods. Unless otherwise stated, all reactions were conducted under inert atmosphere using standard Schlenk techniques or in an nitrogen-filled glove-box. **^{1}H NMR** and **^{13}C NMR** spectra were recorded at room temperature in $CDCl_3$ on 400 or 700 MHz instruments with tetramethylsilane (TMS) as internal standard. Flash column chromatography was performed on silica gel (200-300 mesh). All reactions were monitored by TLC, NMR or GC-FID analysis. **HRMS** data was obtained with Micromass HPLC-Q-TOF mass spectrometer (ESI) or Agilent 6540 Accurate-MS spectrometer (Q-TOF).

2. Supplementary Note 2

2.1. Optimization studies of reaction conditions

Supplementary Table 1. Reactions under Jui's conditions[a]

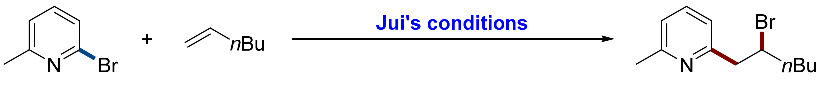

Entry	Reaction conditions	Yield of **3aa** (%)
1	*Ref. 56* (Reductive quenching activation)<br>[Ir (1 mol%), HEH (1.3 eq.), NH_4Cl (2.0 eq.)]	N.D.
2	*Ref. 59* (Reductive quenching activation)<br>[Ir (1 mol%), HEH (1.3 eq.), CySH (5 mol%)]	N.D.
3	*Ref. 56* without HEH<br>[Ir (1 mol%), NH_4Cl (2.0 eq.)]	N.D.
4	*Ref. 59* without HEH<br>[Ir (1 mol%), CySH (5 mol%)]	Trace

[a] Reaction conditions: **1a** (0.2 mmol), **2a** (0.60 mmol), $Ir(ppy)_2(dtbbpy)PF_6$ (1.0 mol%), TFE (2.0 mL), blue LEDs (λ_{max}= 456 nm, 40 W), N_2 atmosphere, room temperature, 16 h; Yields were determined by GC-FID analysis of the crude reaction mixture using mesitylene as internal standard.

Supplementary Table 2. Influence of the photocatalysts[a]

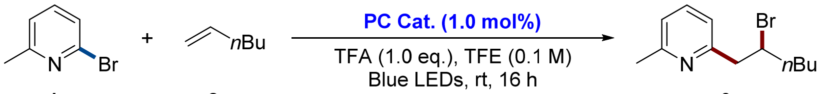

Entry	PC Cat.	Yield of **3aa** (%)
1	Ir(ppy)$_2$(dtbbpy)PF$_6$	89
2	Ir(4-Fppy)$_2$(dtbbpy)PF$_6$	75
3	Ir[dF(CF$_3$)ppy]$_2$(dtbbpy)PF$_6$	0
4	*fac*-Ir(ppy)$_3$	N. D.
5	Eosin Y	N. D.
6	Acid Red 94	N. D.
7	(Acr-Mes)ClO$_4$	N. D.
8	Ru(bpy)$_3$Cl$_2$·6H$_2$O	N. D.
9	Ru(phen)$_3$(PF$_6$)$_2$	N. D.
10	Ru(bpy)$_3$(PF$_6$)$_2$	N. D.
11	Ru(dtbbpy)$_3$(PF$_6$)$_2$	N. D.

[a] Reaction conditions: **1a** (0.2 mmol), **2a** (0.60 mmol), PC Cat. (1.0 mol%), TFA (0.2 mmol), TFE (2.0 mL), blue LEDs (λ_{max}= 456 nm, 40 W), N_2 atmosphere, room temperature, 16 h; Yields were determined by GC-FID analysis of the crude reaction mixture using mesitylene as internal standard.

Supplementary Table 3. Influence of the solvents[a]

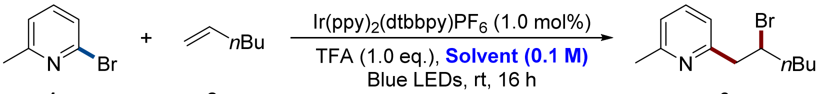

1a **2a** **3aa**

Entry	Solvent	Yield of **3aa** (%)
1	TFE	89
2	HFIP	86
3	EtOH	Trace
4	MeOH	Trace
5	THF	N. D.
6	DMSO	N. D.
7	1,4-Dioxane	N. D.
8	DMF	Trace
9	MeCN	21%
10	DCE	N. D.

[a] Reaction conditions: **1a** (0.2 mmol), **2a** (0.60 mmol), Ir(ppy)$_2$(dtbbpy)PF$_6$ (1.0 mol%), TFA (0.2 mmol), solvent (2.0 mL), blue LEDs (λ_{max}= 456 nm, 40 W), N_2 atmosphere, room temperature, 16 h; Yields were determined by GC-FID analysis of the crude reaction mixture using mesitylene as internal standard.

Supplementary Table 4. Influence of the acid additives[a]

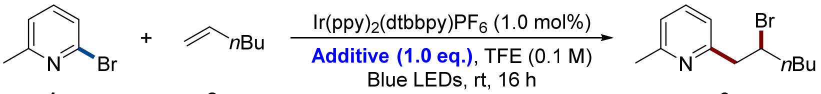

1a **2a** **3aa**

Entry	Additive (1.0 eq.)	Yield of **3aa** (%)
1	TFA	89
2	PivOH	Trace
3	AcOH	N. D.
4	$PhCO_2H$	<10
5	$(C_6H_5O)_2P(O)OH$	76
6	$PhSO_3H$	80
7	HCl	N. D.

[a] Reaction conditions: **1a** (0.2 mmol), **2a** (0.60 mmol), Ir(ppy)$_2$(dtbbpy)PF$_6$ (1.0 mol%), additive (0.2 mmol), TFE (2.0 mL), blue LEDs (λ_{max}= 456 nm, 40 W), N_2 atmosphere, room temperature, 16 h; Yields were determined by GC-FID analysis of the crude reaction mixture using mesitylene as internal standard.

Supplementary Table 5. Influence of the dosage of TFA [a]

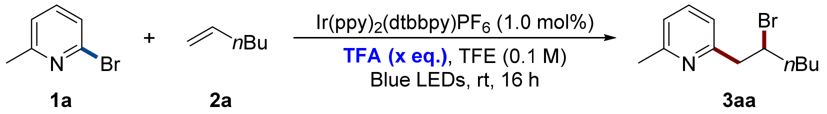

Entry	Equivalents of TFA	Yield of **3aa** (%)
1	0.25 eq.	61
2	0.50 eq	73
3	1.00 eq	89
4	1.50 eq	87

[a] Reaction conditions: **1a** (0.2 mmol), **2a** (0.60 mmol), Ir(ppy)$_2$(dtbbpy)PF$_6$ (1.0 mol%), TFA (x eq.), TFE (2.0 mL), blue LEDs (λ_{max}= 456 nm, 40 W), N_2 atmosphere, room temperature, 16 h; Yields were determined by GC-FID analysis of the crude reaction mixture using mesitylene as internal standard.

Supplementary Table 6. Influence of the dosage of alkenes 2a [a]

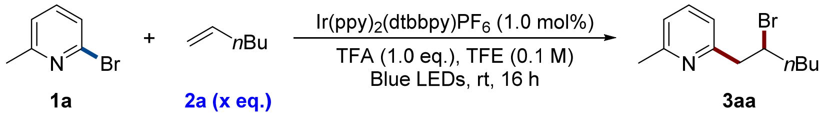

Entry	Equivalents of **2a**	Yield of **3aa** (%)
1	1.0 eq.	79
2	2.0 eq	85
3	3.0 eq	89
4	4.0 eq	88

[a] Reaction conditions: **1a** (0.2 mmol), **2a** (x eq.), Ir(ppy)$_2$(dtbbpy)PF$_6$ (1.0 mol%), TFA (0.2 mmol), TFE (2.0 mL), blue LEDs (λ_{max}= 456 nm, 40 W), N_2 atmosphere, room temperature, 16 h; Yields were determined by GC-FID analysis of the crude reaction mixture using mesitylene as internal standard.

Supplementary Table 7. Influence of the wavelength of Kessil light[a]

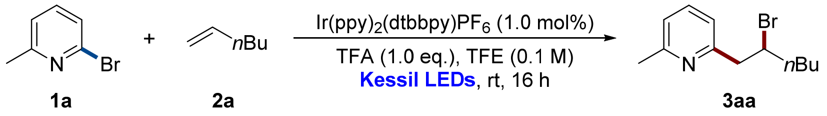

Entry	Wavelength of Kessil light	Yield of **3aa** (%)
1	456 nm	89
2	427 nm	87
3	390 nm	82

[a] Reaction conditions: **1a** (0.2 mmol), **2a** (0.6 mmol), Ir(ppy)$_2$(dtbbpy)PF$_6$ (1.0 mol%), TFA (0.2 mmol), TFE (2.0 mL), Kessil LEDs, N_2 atmosphere, room temperature, 16 h; Yields were determined by GC-FID analysis of the crude reaction mixture using mesitylene as internal standard.

Supplementary Table 8. Control experiments[a]

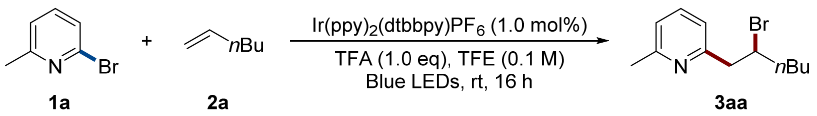

Entry	Deviation from standard conditions	Yield of **3aa** (%)
1	none	89
2	No $Ir(ppy)_2(dtbbpy)PF_6$	N. D.
3	No light	N. D.
4	No TFA	Trace
5	No light at 80 °C oil bath	N. D.
6	Air instead of N_2	Trace
7	12 h instead of 16 h	82
8	20 h instead of 16 h	86

[a] Reaction conditions: **1a** (0.2 mmol), **2a** (0.60 mmol), $Ir(ppy)_2(dtbbpy)PF_6$ (1.0 mol%), TFA (0.2 mmol), TFE (2.0 mL), blue LEDs (λ_{max}= 456 nm, 40 W), N_2 atmosphere, room temperature, 16 h; Yields were determined by GC-FID analysis of the crude reaction mixture using mesitylene as internal standard.

2.2. Synthesis of complex alkene precursors

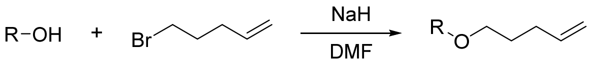

To a solution of ROH (5 mmol, 1.0 equiv.) in DMF (10 mL) was added NaH (10 mmol, 60 wt % in mineral oil, 2.0 equiv.) at 0 ℃. After stirred for 30 min, to the mixture was added 5-bromo-1-pentene (6 mmol, 1.2 equiv.) dropwise at 0 ℃. After stirred for 6 h at room temperature, the mixture was quenched with saturated aq. NH_4Cl and extracted with EtOAc. The combined organic layer was washed with brine, dried over Na_2SO_4, and concentrated in vacuo. The residue was purified by flash column chromatography on silica gel to afford the product.

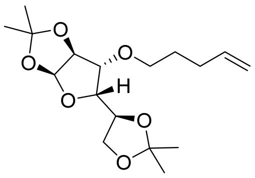

(3aS,5S,6R,6aS)-5-((S)-2,2-Dimethyl-1,3-dioxolan-4-yl)-2,2-dimethyl-6-(pent-4-en-1-yloxy)tetrahydrofuro[2,3-d][1,3]dioxole (Alkene 2p): Colorless oil, 1.36 g, 83% yield. **^{1}H NMR** (400 MHz, $CDCl_3$) δ 5.85 (d, *J* = 3.5 Hz, 1H), 5.84 – 5.72 (m, 1H), 5.01 (d, *J* = 17.1 Hz, 1H), 4.95 (d, *J* = 10.2 Hz, 1H), 4.51 (d, *J* = 3.6 Hz, 1H), 4.29 (q, *J* = 6.5 Hz, 1H), 4.12 – 4.03 (m, 2H), 4.00 – 3.93 (m, 1H), 3.83 (d, *J* = 2.6 Hz, 1H), 3.64 – 3.57 (m, 1H), 3.54 – 3.47 (m, 1H), 2.11 (q, *J* = 7.1 Hz, 2H), 1.69 – 1.60 (m, 3H), 1.48 (s, 3H), 1.41 (s, 3H), 1.33 (s, 3H), 1.30 (s, 3H). **^{13}C NMR** (100 MHz, $CDCl_3$) δ 138.16, 115.07, 111.82, 109.00, 105.39, 82.60, 82.23, 81.33, 72.59, 69.83, 67.38,

30.25, 28.98, 26.96, 26.90, 26.36, 25.51. **HRMS** (m/z) $[M+H]^+$ calcd. for $C_{17}H_{29}O_6$, 329.1964, found 329.1968.

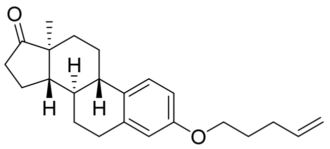

(8R,9S,13S,14S)-13-Methyl-3-(pent-4-en-1-yloxy)-6,7,8,9,11,12,13,14,15,16-decahydro-17H-cyclopenta[a]phenanthren-17-one (Alkene 2q): White solid, 1.29 g, 76% yield, m. p. 82-83 ℃. **^{1}H NMR** (400 MHz, $CDCl_3$) δ 7.19 (d, J = 8.6 Hz, 1H), 6.75 – 6.69 (m, 1H), 6.65 (s, 1H), 5.92 – 5.79 (m, 1H), 5.07 (d, J = 17.1 Hz, 1H), 5.00 (d, J = 10.2 Hz, 1H), 3.95 (t, J = 6.4 Hz, 2H), 2.95 – 2.83 (m, 2H), 2.56 – 2.46 (m, 1H), 2.44 – 2.35 (m, 1H), 2.29 – 2.19 (m, 3H), 2.19 – 1.93 (m, 4H), 1.92 – 1.83 (m, 2H), 1.68 – 1.40 (m, 6H), 0.91 (s, 3H). **^{13}C NMR** (100 MHz, $CDCl_3$) δ 138.01, 132.03, 126.41, 115.24, 114.69, 112.25, 67.20, 50.55, 48.14, 44.12, 38.52, 36.00, 31.72, 30.27, 29.79, 28.64, 26.70, 26.06, 21.72, 13.99. **HRMS** (m/z) $[M+H]^+$ calcd. for $C_{23}H_{31}O_2$, 339.2324, found 339.2323.

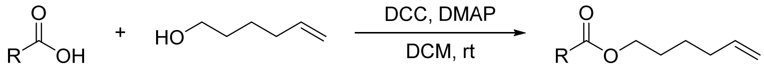

To a stirred solution of carboxylic acid (5 mmol, 1 equiv.) and DCC (dicyclohexylcarbodiimide) (10 mmol, 2 equiv.) in DCM (15 mL) were added DMAP (4-dimethylaminepyridine) (0.75 mmol, 0.15 equiv.) and alcohol (6 mmol, 1.2 equiv.). The reaction mixture was stirred at rt for 24 h. After filtration, the filtrate was concentrated in vacuo and the residue was purified by flash column chromatography on silica gel to afford the product.

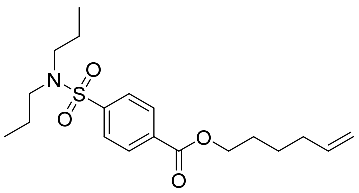

Hex-5-en-1-yl 4-(N,N-dipropylsulfamoyl)benzoate (Alkene 2r): Colorless oil, 1.43 g, 81% yield. **^{1}H NMR** (400 MHz, $CDCl_3$) δ 8.14 (d, J = 8.0 Hz, 2H), 7.86 (d, J = 8.0 Hz, 2H), 5.88 – 5.74 (m, 1H), 5.03 (d, J = 17.2 Hz, 1H), 4.98 (d, J = 10.2 Hz, 1H), 4.35 (t, J = 6.6 Hz, 2H), 3.09 (t, J = 7.6 Hz, 4H), 2.13 (q, J = 7.1 Hz, 2H), 1.85 – 1.75 (m, 2H), 1.60 – 1.49 (m, 6H), 0.86 (t, J = 7.4 Hz, 6H). **^{13}C NMR** (100 MHz, $CDCl_3$) δ 165.41, 144.30, 138.31, 133.85, 130.29, 127.11, 115.13, 65.68, 50.06, 33.40, 28.21, 25.38, 22.07, 11.28. **HRMS** (m/z) $[M+H]^+$ calcd. for $C_{19}H_{30}NO_4S$, 368.1896, found 368.1902.

2.3. Unsuccessful halopyridines with strong electron withdrawing substituent

Supplementary Table 9. Unsuccessful halopyridine substrates

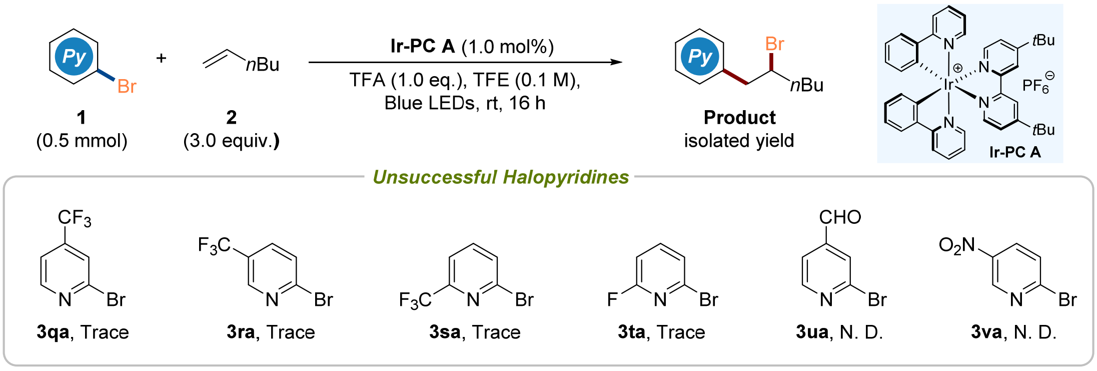

2.4. General procedure for photo-induced catalytic halopyridylation of alkenes

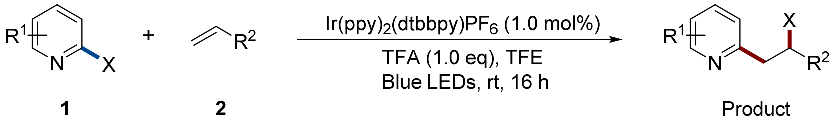

To an oven-dried 4 mL vial was added $Ir(ppy)_2(dtbbpy)PF_6$ (0.005 mmol, 1.0 mol%), halopyridines **1** (0.5 mmol, 1.0 equiv.), alkenes **2** (1.5 mmol, 3.0 equiv.), TFA (0.5 mmol, 1.0 equiv.) and TFE (2.5 mL, 0.2 M) in the nitrogen glove box. The vial was capped with a septum and wrapped with parafilm. The reaction mixture was stirred for 16 h under visible light irradiation (1 x Kessil PR160, λ_{max}= 456 nm, 40 W, irradiation temperature maintained between 25-30 ℃) as shown in Supplementary Figure 1. After reaction completed, the crude product was neutralized with saturated $NaHCO_3$ solution or Et_3N and extracted with ethyl acetate. Organic layer was washed with brine solution and dried over anhydrous Na_2SO_4. Removal of the organic solvent in a vacuum rotavapor followed by flash silica gel column chromatographic purification (hexane/ethyl acetate) to afford the desired products in moderate to good yields.

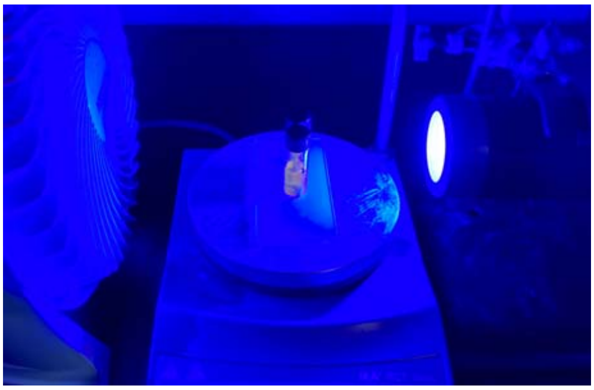

Supplementary Figure 1. Reaction setups

2.5. Analytical data for the halopyridylation products

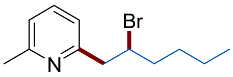

2-(2-Bromohexyl)-6-methylpyridine (Product 3aa):
Colorless oil, 111.4 mg, 87% yield. **^{1}H NMR** (400 MHz, $CDCl_3$) δ 7.51 (t, J = 7.7 Hz, 1H), 7.01 (t, J = 7.9 Hz, 2H), 4.52 – 4.44 (m, 1H), 3.30 (dd, J = 14.2, 5.2 Hz, 1H), 3.22 (dd, J = 14.2, 8.9 Hz, 1H), 2.53 (s, 3H), 1.96 – 1.81 (m, 2H), 1.66 – 1.55 (m, 1H), 1.52 – 1.39 (m, 1H), 1.37 – 1.26 (m, 2H), 0.91 (t, J = 7.3 Hz, 3H). **^{13}C NMR** (100 MHz, $CDCl_3$) δ 158.22, 157.95, 136.62, 121.48, 121.13, 57.20, 48.03, 38.75, 29.81, 24.65, 22.26, 14.11. **HRMS** (m/z) $[M+H]^+$ calcd. for $C_{12}H_{19}BrN$, 256.0695, found 256.0696.

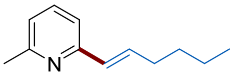

2-(Hex-1-en-1-yl)-6-methylpyridine (Byproduct 3aa'):
Colorless oil, 5.3 mg, 6% yield. **^{1}H NMR** (400 MHz, $CDCl_3$) δ 7.47 (t, J = 7.7

Hz, 1H), 7.07 (d, *J* = 7.8 Hz, 1H), 6.94 (d, *J* = 7.6 Hz, 1H), 6.68 (dt, *J* = 14.4, 6.9 Hz, 1H), 6.46 (d, *J* = 15.7 Hz, 1H), 2.52 (s, 3H), 2.30 – 2.19 (m, 2H), 1.52 – 1.44 (m, 2H), 1.42 – 1.33 (m, 2H), 0.91 (t, *J* = 7.2 Hz, 3H). **^{13}C** NMR (100 MHz, $CDCl_3$) δ 158.02, 155.84, 136.63, 135.75, 130.33, 121.16, 117.78, 32.64, 31.24, 24.73, 22.45, 14.07. **HRMS** (m/z) $[M+H]^+$ calcd. for $C_{12}H_{18}N$, 176.1439, found 176.1441.

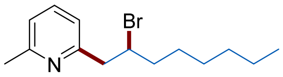

2-(2-Bromooctyl)-6-methylpyridine (Product 3ab): Colorless oil, 126.5 mg, 89% yield. **^{1}H NMR** (400 MHz, $CDCl_3$) δ 7.49 (t, *J* = 7.7 Hz, 1H), 7.00 (t, *J* = 7.9 Hz, 2H), 4.53 – 4.43 (m, 1H), 3.29 (dd, *J* = 14.2, 5.3 Hz, 1H), 3.21 (dd, *J* = 14.2, 8.9 Hz, 1H), 2.53 (s, 3H), 1.95 – 1.79 (m, 2H), 1.69 – 1.55 (m, 1H), 1.51 – 1.40 (m, 1H), 1.33 – 1.24 (m, 6H), 0.87 (t, *J* = 6.8 Hz, 3H). **^{13}C NMR** (100 MHz, $CDCl_3$) δ 158.20, 157.95, 136.54, 121.42, 121.07, 57.18, 48.03, 39.01, 31.78, 28.77, 27.58, 24.65, 22.69, 14.18. **HRMS** (m/z) $[M+H]^+$ calcd. for $C_{14}H_{23}BrN$, 284.1008, found 284.1010.

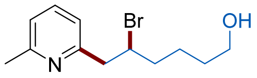

5-Bromo-6-(6-methylpyridin-2-yl)hexan-1-ol (Product 3ac): Colorless oil, 113.0 mg, 83% yield. **^{1}H NMR** (400 MHz, $CDCl_3$) δ 7.51 (t, *J* = 7.6 Hz, 1H), 7.00 (t, *J* = 8.2 Hz, 2H), 4.53 – 4.42 (m, 1H), 3.64 (t, *J* = 5.9 Hz, 2H), 3.30 (dd, *J* = 14.1, 5.3 Hz, 1H), 3.22 (dd, *J* = 14.1, 8.7 Hz, 1H), 2.52 (s, 3H), 2.14 (brs, 1H), 1.99 – 1.83 (m, 2H), 1.77 – 1.66 (m, 1H), 1.65 – 1.51 (m, 3H). **^{13}C NMR** (100 MHz, $CDCl_3$) δ 158.21, 157.75, 136.72, 121.59, 121.23, 62.46, 56.64, 47.68, 38.41, 32.04, 24.52, 23.79. **HRMS** (m/z) $[M+H]^+$ calcd. for $C_{12}H_{19}BrNO$, 272.0645, found 272.0647.

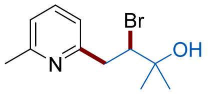

3-Bromo-2-methyl-4-(6-methylpyridin-2-yl)butan-2-ol (Product 3ad): Colorless oil, 92.9 mg, 72% yield. **^{1}H NMR** (400 MHz, $CDCl_3$) δ 7.53 (t, *J* = 7.7 Hz, 1H), 7.02 (t, *J* = 8.0 Hz, 2H), 4.52 (dd, *J* = 9.0, 3.9 Hz, 1H), 3.76 (brs, 1H), 3.53 (dd, *J* = 14.9, 3.9 Hz, 1H), 3.26 (dd, *J* = 14.9, 9.0 Hz, 1H), 2.53 (s, 3H), 1.48 (s, 3H), 1.45 (s, 3H). **^{13}C NMR** (100 MHz, $CDCl_3$) δ 157.87, 157.82, 137.02, 121.59, 121.51, 72.61, 66.50, 42.48, 27.76, 26.81, 24.31. **HRMS** (m/z) $[M+H]^+$ calcd. for $C_{11}H_{17}BrNO$, 258.0488, found 258.0490.

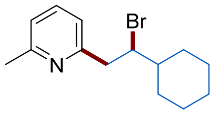

2-(2-Bromo-2-cyclohexylethyl)-6-methylpyridine (Product 3ae): Colorless oil, 118.5 mg, 84% yield. **^{1}H NMR** (400 MHz, $CDCl_3$) δ 7.49 (t, *J* = 7.7 Hz, 1H), 6.99 (t, *J* = 8.4 Hz, 2H), 4.47 (dt, *J* = 10.0, 3.9 Hz, 1H), 3.30 (dd, *J* = 14.3, 4.3 Hz, 1H), 3.19 (dd, *J* = 14.3, 10.0 Hz, 1H), 2.53 (s, 3H), 1.94 – 1.87 (m, 1H), 1.86 – 1.73 (m, 4H), 1.70 – 1.64 (m, 1H), 1.62 – 1.53 (m, 1H), 1.43 – 1.33 (m, 1H), 1.28 – 1.21 (m, 2H), 1.21 – 1.10 (m, 1H). **^{13}C NMR** (100 MHz, $CDCl_3$) δ 158.33, 158.20, 136.54, 121.38, 121.07, 64.21, 45.10, 44.45, 31.54, 28.74, 26.42, 26.31, 26.16, 24.66. **HRMS** (m/z) $[M+H]^+$ calcd. for $C_{14}H_{21}BrN$, 282.0852, found 282.0853.

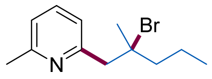

2-(2-Bomo-2-methylpentyl)-6-methylpyridine (Product 3af): Colorless oil, 97.4 mg, 76% yield. **^{1}H NMR** (400 MHz, $CDCl_3$) δ 7.52 (t, *J* = 7.7 Hz, 1H), 7.16 (d, *J* = 7.6 Hz, 1H), 7.03 (d, *J* = 7.6 Hz, 1H), 3.38 (d, *J* = 13.6 Hz, 1H), 3.31 (d, *J* = 13.6 Hz, 1H), 2.53 (s, 3H), 1.92 – 1.79 (m, 2H), 1.75 (s, 3H), 1.70 – 1.57 (m, 2H), 0.95 (t, *J* = 7.3 Hz, 3H). **^{13}C NMR** (100 MHz, $CDCl_3$) δ 157.56, 156.98, 136.23, 122.43, 121.43, 72.29,

53.21, 47.87, 31.61, 24.64, 19.51, 14.16. **HRMS** (m/z) $[M+H]^+$ calcd. for $C_{12}H_{19}BrN$, 256.0695, found 256.0698.

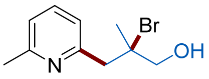

2-Bromo-2-methyl-3-(6-methylpyridin-2-yl)propan-1-ol (Product 3ag): Colorless oil, 87.9 mg, 72% yield. **^{1}H NMR** (400 MHz, $CDCl_3$) δ 7.57 (t, J = 7.7 Hz, 1H), 7.10 – 7.03 (m, 2H), 5.73 (brs, 1H), 3.65 (d, J = 12.1 Hz, 1H), 3.52 (d, J = 12.1 Hz, 1H), 3.40 (d, J = 14.1 Hz, 1H), 3.30 (d, J = 14.1 Hz, 1H), 2.51 (s, 3H), 1.87 (s, 3H). **^{13}C NMR** (100 MHz, $CDCl_3$) δ 157.64, 156.18, 137.40, 122.30, 121.95, 70.15, 68.80, 49.75, 30.30, 24.33. **HRMS** (m/z) $[M+H]^+$ calcd. for $C_{10}H_{15}BrNO$, 244.0332, found 244.0333.

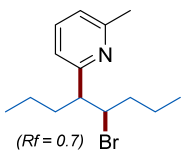

2-(5-Bromooctan-4-yl)-6-methylpyridine (Product 3ah): Colorless oil, 79.6 mg, 56% yield, single isomer, R_f = 0.7 (petroleum ether/ethyl acetate = 10/1). **^{1}H NMR** (400 MHz, $CDCl_3$) δ 7.51 (t, J = 7.7 Hz, 1H), 7.00 (d, J = 7.7 Hz, 1H), 6.97 (d, J = 7.7 Hz, 1H), 4.42 – 4.33 (m, 1H), 3.12 – 2.94 (m, 1H), 2.53 (s, 3H), 2.05 – 1.96 (m, 1H), 1.91 – 1.81 (m, 1H), 1.76 – 1.67 (m, 1H), 1.66 – 1.54 (m, 2H), 1.44 – 1.36 (m, 1H), 1.18 – 1.07 (m, 2H), 0.89 – 0.81 (m, 6H). **^{13}C NMR** (100 MHz, $CDCl_3$) δ 161.18, 158.12, 136.51, 121.50, 120.47, 63.79, 54.42, 39.43, 34.56, 24.57, 21.02, 20.60, 14.26, 13.49. **HRMS** (m/z) $[M+H]^+$ calcd. for $C_{14}H_{23}BrN$, 284.1008, found 284.1010.

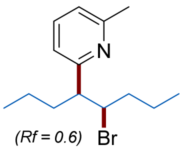

2-(5-Bromooctan-4-yl)-6-methylpyridine (Product 3ah): Colorless oil, 39.8 mg, 28% yield, single isomer, R_f = 0.6 (petroleum ether/ethyl acetate = 10/1). **^{1}H NMR** (400 MHz, $CDCl_3$) δ 7.49 (t, J = 7.7 Hz, 1H), 7.04 (d, J = 7.7 Hz, 1H), 7.00 (d, J = 7.6 Hz, 1H), 4.50 – 4.41 (m, 1H), 3.18 – 3.06 (m, 1H), 2.52 (s, 3H), 1.94 – 1.77 (m, 3H), 1.75 – 1.57 (m, 2H), 1.52 – 1.38 (m, 1H), 1.23 – 1.05 (m, 2H), 0.93 – 0.83 (m, 6H). **^{13}C NMR** (100 MHz, $CDCl_3$) δ 161.02, 157.75, 136.08, 121.40, 120.60, 62.86, 54.27, 38.11, 34.61, 24.74, 20.99, 20.84, 14.23, 13.61. **HRMS** (m/z) $[M+H]^+$ calcd. for $C_{14}H_{23}BrN$, 284.1008, found 284.1011.

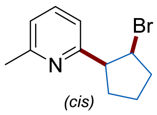

***cis*-2-(2-Bromocyclopentyl)-6-methylpyridine (Product *cis*-3ai):** Colorless oil, 81.6 mg, 68% yield, single isomer. **^{1}H NMR** (400 MHz, $CDCl_3$) δ 7.48 (t, J = 7.7 Hz, 1H), 6.98 (dd, J = 7.6, 5.2 Hz, 2H), 4.57 – 4.47 (m, 1H), 3.49 – 3.35 (m, 1H), 2.52 (s, 3H), 2.50 – 2.42 (m, 1H), 2.22 – 2.09 (m, 2H), 1.99 – 1.82 (m, 3H). **^{13}C NMR** (100 MHz, $CDCl_3$) δ 160.70, 158.37, 136.50, 121.40, 119.88, 57.94, 55.79, 37.61, 32.24, 24.78, 23.71. **HRMS** (m/z) $[M+H]^+$ calcd. for $C_{11}H_{15}BrN$, 240.0382, found 240.0381.

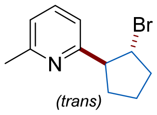

***trans*-2-(2-Bromocyclopentyl)-6-methylpyridine (Product *trans*-3ai):** Colorless oil, 15.6 mg, 13% yield, single isomer. **^{1}H NMR** (400 MHz, $CDCl_3$) δ 7.53 (t, J = 7.7 Hz, 1H), 7.06 (d, J = 7.8 Hz, 1H), 7.02 (d, J = 7.6 Hz, 1H), 4.91 (t, J = 3.5 Hz, 1H), 3.42 – 3.32 (m, 1H), 2.54 (s, 3H), 2.48 – 2.24 (m, 3H), 2.20 – 2.10 (m, 1H), 2.10 – 2.03 (m, 1H), 1.93 – 1.82 (m, 1H). **^{13}C NMR** (100 MHz, $CDCl_3$) δ 159.79, 157.64, 136.30, 121.48, 118.89, 61.79, 54.32, 37.13, 25.89, 24.67, 21.70. **HRMS** (m/z) $[M+H]^+$ calcd. for $C_{11}H_{15}BrN$, 240.0382, found 240.0385.

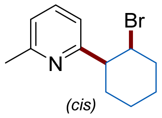

cis-2-(2-Bromocyclohexyl)-6-methylpyridine (Product cis-3aj): Colorless oil, 54.6 mg, 43% yield, single isomer. **^{1}H NMR** (700 MHz, $CDCl_3$) δ 7.49 (t, J = 7.6 Hz, 1H), 6.99 (d, J = 7.6 Hz, 1H), 6.92 (d, J = 7.6 Hz, 1H), 4.48 (td, J = 11.7, 4.3 Hz, 1H), 2.98 (td, J = 11.7, 3.9 Hz, 1H), 2.53 (s, 3H), 2.52 – 2.49 (m, 1H), 2.01 – 1.93 (m, 2H), 1.85 – 1.78 (m, 2H), 1.70 – 1.64 (m, 1H), 1.51 – 1.42 (m, 2H). **^{13}C NMR** (175 MHz, $CDCl_3$) δ 162.61, 158.03, 136.52, 121.39, 119.41, 56.81, 55.75, 38.71, 35.09, 27.54, 25.70, 24.78. **HRMS** (m/z) $[M+H]^+$ calcd. for $C_{12}H_{17}BrN$, 254.0539, found 254.0541.

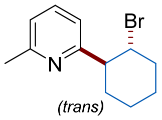

trans-2-(2-Bromocyclohexyl)-6-methylpyridine (Product trans-3aj): Colorless oil, 53.4 mg, 42% yield, single isomer. **^{1}H NMR** (700 MHz, $CDCl_3$) δ 7.53 (t, J = 7.7 Hz, 1H), 7.01 (d, J = 7.6 Hz, 1H), 6.95 (d, J = 7.8 Hz, 1H), 5.04 – 5.00 (m, 1H), 2.95 (dt, J = 12.4, 2.9 Hz, 1H), 2.53 (s, 3H), 2.29 – 2.25 (m, 1H), 2.11 – 2.04 (m, 2H), 1.97 – 1.92 (m, 1H), 1.92 – 1.86 (m, 1H), 1.86 – 1.82 (m, 1H), 1.65 – 1.62 (m, 1H), 1.49 – 1.42 (m, 1H). **^{13}C NMR** (175 MHz, $CDCl_3$) δ 161.91, 157.59, 136.46, 121.49, 118.26, 61.72, 49.99, 35.47, 25.83, 24.65, 20.66. **HRMS** (m/z) $[M+H]^+$ calcd. for $C_{12}H_{17}BrN$, 254.0539, found 254.0542.

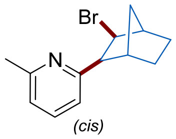

cis-2-(3-Bromobicyclo[2.2.1]heptan-2-yl)-6-methylpyridine (Product cis-3ak): Colorless oil, 95.8 mg, 72% yield, single isomer. **^{1}H NMR** (400 MHz, $CDCl_3$) δ 7.51 (t, J = 7.7 Hz, 1H), 7.01 (d, J = 7.8 Hz, 1H), 6.97 (d, J = 7.6 Hz, 1H), 4.55 (dd, J = 7.7, 1.7 Hz, 1H), 3.29 (d, J = 7.8 Hz, 1H), 2.69 – 2.64 (m, 2H), 2.52 (s, 3H), 2.34 – 2.28 (m, 1H), 1.82 – 1.70 (m, 1H), 1.67 – 1.55 (m, 1H), 1.49 – 1.44 (m, 1H), 1.41 – 1.32 (m, 2H). **^{13}C NMR** (100 MHz, $CDCl_3$) δ 162.24, 157.11, 136.17, 120.99, 119.52, 61.02, 55.90, 47.54, 41.76, 36.05, 30.52, 27.41, 24.70. **HRMS** (m/z) $[M+H]^+$ calcd. for $C_{13}H_{17}BrN$, 266.0539, found 266.0537.

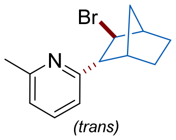

tranas-2-(3-Bromobicyclo[2.2.1]heptan-2-yl)-6-methylpyridine (Product trans-3ak):

Colorless oil, 24.0 mg, 18% yield, single isomer. **^{1}H NMR** (400 MHz, $CDCl_3$) δ 7.46 (t, J = 7.7 Hz, 1H), 6.97 (dd, J = 7.6, 4.1 Hz, 2H), 4.93 – 4.86 (m, 1H), 2.89 (dd, J = 5.2, 2.2 Hz, 1H), 2.59 – 2.55 (m, 1H), 2.51 (s, 3H), 2.32 – 2.28 (m, 1H), 2.12 – 2.01 (m, 2H), 1.71 – 1.64 (m, 1H), 1.61 – 1.55 (m, 1H), 1.55 – 1.49 (m, 1H), 1.35 – 1.30 (m, 1H). **^{13}C NMR** (100 MHz, $CDCl_3$) δ 161.51, 157.84, 136.45, 120.92, 119.62, 60.17, 58.84, 44.85, 44.57, 35.36, 30.61, 24.83, 24.24. **HRMS** (m/z) $[M+H]^+$ calcd. for $C_{13}H_{17}BrN$, 266.0539, found 266.0543.

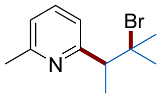

2-(3-Bromo-3-methylbutan-2-yl)-6-methylpyridine (Product 3al): Colorless oil, 83.5 mg, 69% yield. **^{1}H NMR** (400 MHz, $CDCl_3$) δ 7.51 (t, J = 7.7 Hz, 1H), 7.16 (d, J = 7.7 Hz, 1H), 7.01 (d, J = 7.6 Hz, 1H), 3.26 – 3.14 (m, 1H), 2.52 (s, 3H), 1.78 (s, 3H), 1.77 (s, 3H), 1.52 (d, J = 7.0 Hz, 3H). **^{13}C NMR** (100 MHz, $CDCl_3$) δ 161.63, 157.19, 136.29, 121.50, 120.35, 72.67, 54.88, 34.24, 32.09, 24.66, 17.97. **HRMS** (m/z) $[M+H]^+$ calcd. for $C_{11}H_{17}BrN$, 242.0539, found 242.0538.

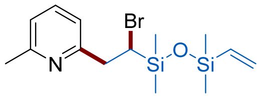

2-(2-Bromo-2-(1,1,3,3-tetramethyl-3-vinyldisiloxaneyl)ethyl)-6-methylpyridine (Product 3an): Colorless oil, 118.3 mg, 66% yield. **^{1}H NMR** (400 MHz, $CDCl_3$) δ 7.50 (t, J = 7.6 Hz, 1H), 7.00 (d, J = 7.8 Hz, 2H), 6.15 (dd, J = 20.3, 14.8 Hz, 1H), 5.96 (dd, J = 14.8, 3.9 Hz,

1H), 5.75 (dd, J = 20.3, 3.9 Hz, 1H), 3.76 (dd, J = 12.2, 3.5 Hz, 1H), 3.40 (dd, J = 14.9, 3.5 Hz, 1H), 3.03 (dd, J = 14.9, 12.2 Hz, 1H), 2.54 (s, 3H), 0.27 (s, 3H), 0.25 (s, 3H), 0.19 (s, 6H). **^{13}C NMR** (100 MHz, $CDCl_3$) δ 158.92, 158.15, 139.27, 136.46, 132.15, 121.31, 120.67, 42.33, 41.11, 24.63, 0.50. -0.99, -1.57. **HRMS** (m/z) $[M+H]^+$ calcd. for $C_{14}H_{25}BrNOSi_2$, 358.0653, found 358.0655.

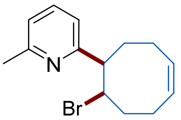

2-(8-Bromocyclooct-4-en-1-yl)-6-methylpyridine (Product 3ao): Colorless oil, 98.1 mg, 70% yield, mixture of diastereomers (dr = 12:1). **^{1}H NMR** (400 MHz, $CDCl_3$) δ 7.46 (t, J = 7.2 Hz, 1H), 6.96 (d, J = 7.4 Hz, 1H), 6.88 (d, J = 7.5 Hz, 1H), 5.86 – 5.68 (m, 1H), 5.68 – 5.46 (m, 1H), 5.13 – 5.04 (m, 0.92H), 4.32 – 4.14 (m, 0.08H), 3.72 – 3.34 (m, 1H), 3.05 – 2.89 (m, 1H), 2.82 – 2.68 (m, 2H), 2.52 (s, 3H), 2.41 – 2.24 (m, 2H), 2.16 – 2.07 (m, 1H), 2.06 – 1.97 (m, 1H), 1.95 – 1.84 (m, 1H). **^{13}C NMR** (100 MHz, $CDCl_3$) δ 164.22, 157.77, 136.43, 131.21, 126.35, 121.15, 119.63, 62.66, 52.09, 35.49, 33.51, 26.85, 24.95, 24.77. **HRMS** (m/z) $[M+H]^+$ calcd. for $C_{14}H_{19}BrN$, 280.0965, found 280.0967.

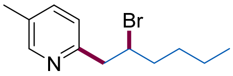

2-(2-Bromohexyl)-5-methylpyridine (Product 3ba): Colorless oil, 114.0 mg, 89% yield. **^{1}H NMR** (400 MHz, $CDCl_3$) δ 8.37 (s, 1H), 7.42 (dd, J = 7.8, 1.8 Hz, 1H), 7.08 (d, J = 7.8 Hz, 1H), 4.49 – 4.42 (m, 1H), 3.30 (dd, J = 14.2, 5.1 Hz, 1H), 3.20 (dd, J = 14.2, 8.9 Hz, 1H), 2.30 (s, 3H), 1.94 – 1.81 (m, 2H), 1.65 – 1.52 (m, 1H), 1.51 – 1.40 (m, 1H), 1.38 – 1.23 (m, 2H), 0.89 (t, J = 7.3 Hz, 3H). **^{13}C NMR** (100 MHz, $CDCl_3$) δ 155.65, 149.94, 136.99, 131.23, 123.61, 57.06, 47.46, 38.73, 29.83, 22.22, 18.25, 14.08. **HRMS** (m/z) $[M+H]^+$ calcd. for $C_{12}H_{19}BrN$, 256.0695, found 256.0698.

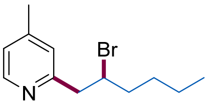

2-(2-Bromohexyl)-4-methylpyridine (Product 3ca): Colorless oil, 99.9 mg, 78% yield. **^{1}H NMR** (400 MHz, $CDCl_3$) δ 8.40 (d, J = 5.0 Hz, 1H), 7.01 (s, 1H), 6.98 (d, J = 5.0 Hz, 1H), 4.53 – 4.44 (m, 1H), 3.31 (dd, J = 14.2, 5.1 Hz, 1H), 3.19 (dd, J = 14.2, 9.0 Hz, 1H), 2.34 (s, 3H), 1.94 – 1.83 (m, 2H), 1.65 – 1.53 (m, 1H), 1.51 – 1.42 (m, 1H), 1.41 – 1.27 (m, 2H), 0.90 (t, J = 7.3 Hz, 3H). **^{13}C NMR** (100 MHz, $CDCl_3$) δ 158.34, 149.20, 147.68, 125.11, 122.95, 56.81, 47.78, 38.80, 29.85, 22.23, 21.18, 14.09. **HRMS** (m/z) $[M+H]^+$ calcd. for $C_{12}H_{19}BrN$, 256.0695, found 256.0695.

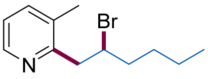

2-(2-Bromohexyl)-3-methylpyridine (Product 3da): Colorless oil, 117.8 mg, 92% yield. **^{1}H NMR** (400 MHz, $CDCl_3$) δ 8.40 (d, J = 4.6 Hz, 1H), 7.44 (d, J = 7.6 Hz, 1H), 7.07 (dd, J = 7.6, 4.8 Hz, 1H), 4.66 – 4.57 (m, 1H), 3.40 (dd, J = 14.6, 8.8 Hz, 1H), 3.25 (dd, J = 14.6, 5.4 Hz, 1H), 2.36 (s, 3H), 1.96 – 1.89 (m, 2H), 1.65 – 1.55 (m, 1H), 1.52 – 1.42 (m, 1H), 1.40 – 1.28 (m, 2H), 0.91 (t, J = 7.3 Hz, 3H). **^{13}C NMR** (100 MHz, $CDCl_3$) δ 157.24, 146.90, 138.07, 131.82, 121.84, 56.93, 44.10, 38.92, 29.96, 22.26, 19.26, 14.10. **HRMS** (m/z) $[M+H]^+$ calcd. for $C_{12}H_{19}BrN$, 256.0695, found 256.0693.

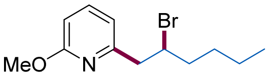

2-(2-Bromohexyl)-6-methoxypyridine (Product 3ea): Colorless oil, 102.1 mg, 75% yield. **^{1}H NMR** (400 MHz, $CDCl_3$) δ 7.50 (t, J = 7.8 Hz, 1H), 6.74 (d, J = 7.2 Hz, 1H), 6.60 (d, J = 8.3 Hz, 1H), 4.60 – 4.52 (m, 1H), 3.91 (s, 3H), 3.27 – 3.15 (m, 2H), 1.95 – 1.80 (m, 2H), 1.51 – 1.44 (m, 1H), 1.43 – 1.30 (m, 3H), 0.91 (t, J = 7.3 Hz, 3H). **^{13}C NMR** (175 MHz, $CDCl_3$) δ 154.53, 150.64, 136.91, 124.37,

121.11, 57.36, 55.75, 46.86, 38.69, 29.86, 22.25, 14.11. **HRMS** (m/z) $[M+H]^+$ calcd. for $C_{12}H_{19}BrNO$, 272.0645, found 272.0641.

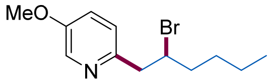

2-(2-Bromohexyl)-5-methoxypyridine (Product 3fa):

Colorless oil, 98.0 mg, 72% yield. **^{1}H NMR** (400 MHz, $CDCl_3$) δ 8.25 (d, *J* = 2.3 Hz, 1H), 7.19 – 7.09 (m, 2H), 4.47 – 4.40 (m, 1H), 3.85 (s, 3H), 3.29 (dd, *J* = 14.3, 5.1 Hz, 1H), 3.19 (dd, *J* = 14.3, 8.8 Hz, 1H), 1.94 – 1.81 (m, 2H), 1.59 – 1.53 (m, 1H), 1.51 – 1.42 (m, 1H), 1.41 – 1.30 (m, 2H), 0.91 (t, *J* = 7.3 Hz, 3H). **^{13}C NMR** (100 MHz, $CDCl_3$) δ 154.54, 150.67, 136.94, 124.36, 121.09, 57.35, 55.75, 46.88, 38.70, 29.87, 22.26, 14.11. **HRMS** (m/z) $[M+H]^+$ calcd. for $C_{12}H_{19}BrNO$, 272.0645, found 272.0646.

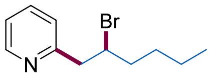

2-(2-Bromohexyl)pyridine (Product 3ga):

Colorless oil, 111.4 mg, 92% yield. **^{1}H NMR** (400 MHz, $CDCl_3$) δ 8.55 (d, *J* = 4.7 Hz, 1H), 7.62 (td, *J* = 7.7, 1.7 Hz, 1H), 7.19 (d, *J* = 7.7 Hz, 1H), 7.16 (dd, *J* = 7.3, 5.0 Hz, 1H), 4.53 – 4.45 (m, 1H), 3.35 (dd, *J* = 14.2, 5.0 Hz, 1H), 3.24 (dd, *J* = 14.2, 9.0 Hz, 1H), 1.93 – 1.86 (m, 2H), 1.66 – 1.53 (m, 1H), 1.52 – 1.41 (m, 1H), 1.40 – 1.28 (m, 2H), 0.90 (t, *J* = 7.3 Hz, 3H). **^{13}C NMR** (100 MHz, $CDCl_3$) δ 158.66, 149.57, 136.44, 124.20, 121.92, 56.71, 47.91, 38.79, 29.83, 22.22, 14.08. **HRMS** (m/z) $[M+H]^+$ calcd. for $C_{11}H_{17}BrN$, 242.0539, found 242.0543.

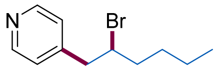

4-(2-Bromohexyl)pyridine (Product 3ha):

Colorless oil, 92.0 mg, 76% yield. **^{1}H NMR** (400 MHz, $CDCl_3$) δ 8.60 (s, 2H), 7.21 (d, *J* = 5.1 Hz, 2H), 4.22 – 4.12 (m, 1H), 3.21 (dd, *J* = 14.4, 5.4 Hz, 1H), 3.13 (dd, *J* = 14.4, 8.4 Hz, 1H), 1.90 – 1.80 (m, 2H), 1.64 – 1.52 (m, 1H), 1.50 – 1.41 (m, 1H), 1.41 – 1.31 (m, 2H), 0.91 (t, *J* = 7.3 Hz, 3H). **^{13}C NMR** (100 MHz, $CDCl_3$) δ 149.65, 125.02, 100.13, 55.36, 44.87, 38.55, 29.81, 22.18, 14.07. **HRMS** (m/z) $[M+H]^+$ calcd. for $C_{11}H_{17}BrN$, 242.0539, found 242.0540.

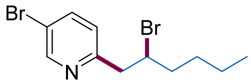

5-Bromo-2-(2-bromohexyl)pyridine (Product 3ia):

Pale brown oil, 96.3 mg, 60% yield. **^{1}H NMR** (400 MHz, $CDCl_3$) δ 8.61 (d, *J* = 2.1 Hz, 1H), 7.75 (dd, *J* = 8.2, 2.3 Hz, 1H), 7.11 (d, *J* = 8.2 Hz, 1H), 4.47 – 4.39 (m, 1H), 3.31 (dd, *J* = 14.3, 4.7 Hz, 1H), 3.19 (dd, *J* = 14.3, 9.1 Hz, 1H), 1.95 – 1.84 (m, 2H), 1.66 – 1.57 (m, 1H), 1.54 – 1.43 (m, 1H), 1.42 – 1.32 (m, 2H), 0.92 (t, *J* = 7.3 Hz, 3H). **^{13}C NMR** (100 MHz, $CDCl_3$) δ 157.24, 150.67, 138.99, 125.50, 118.95, 56.19, 47.14, 38.82, 29.83, 22.23, 14.10. **HRMS** (m/z) $[M+H]^+$ calcd. for $C_{11}H_{16}Br_2N$, 319.9644, found 319.9648.

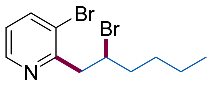

3-Bromo-2-(2-bromohexyl)pyridine (Product 3ja):

Pale brown oil, 104.3 mg, 65% yield. **^{1}H NMR** (400 MHz, $CDCl_3$) δ 8.51 (dd, *J* = 4.6, 1.3 Hz, 1H), 7.84 (dd, *J* = 8.0, 1.4 Hz, 1H), 7.06 (dd, *J* = 8.0, 4.6 Hz, 1H), 4.68 – 4.58 (m, 1H), 3.64 (dd, *J* = 14.8, 8.6 Hz, 1H), 3.44 (dd, *J* = 14.8, 5.6 Hz, 1H), 1.98 – 1.86 (m, 2H), 1.67 – 1.55 (m, 1H), 1.52 – 1.42 (m, 1H), 1.41 – 1.28 (m, 2H), 0.91 (t, *J* = 7.3 Hz, 3H). **^{13}C NMR** (100 MHz, $CDCl_3$) δ 157.19, 147.98, 140.52, 123.20, 122.00, 54.92, 46.21, 38.63, 29.84, 22.23, 14.08. **HRMS** (m/z) $[M+H]^+$ calcd. for $C_{11}H_{16}Br_2N$, 319.9644, found 319.9645.

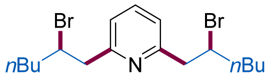

2,6-Bis(2-bromohexyl)pyridine (Product 3ka):

Colorless oil, 141.8 mg, 70% yield, mixture of diastereomers (*dr* = 1:1). **^{1}H NMR** (700 MHz, $CDCl_3$) δ 7.58 – 7.52 (m, 1H), 7.10 – 6.98 (m, 2H), 4.57 – 4.45 (m, 2H), 3.35 – 3.28 (m, 2H), 3.28 – 3.22 (m, 2H), 1.92 – 1.82 (m, 4H), 1.63 – 1.56 (m, 2H), 1.49 – 1.42 (m, 2H), 1.38 – 1.34 (m, 2H), 1.33 – 1.29 (m, 2H), 0.91 (t, J = 7.4 Hz, 6H). **^{13}C NMR** (175 MHz, $CDCl_3$) δ 158.32, 158.29, 136.68, 136.61, 122.11, 122.03, 56.72, 56.61, 47.76, 47.72, 38.56, 38.54, 29.86, 29.80, 22.28, 22.25, 14.13, 14.12. **HRMS** (m/z) $[M+H]^+$ calcd. for $C_{17}H_{28}Br_2N$, 404.0583, found 404.0586.

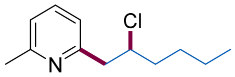

2-(2-Chlorohexyl)-6-methylpyridine (Product 3la):

Colorless oil, 87.9 mg, 83% yield. **^{1}H NMR** (400 MHz, $CDCl_3$) δ 7.50 (t, J = 7.7 Hz, 1H), 7.01 (dd, J = 7.6, 2.8 Hz, 2H), 4.43 – 4.31 (m, 1H), 3.18 (dd, J = 14.0, 5.0 Hz, 1H), 3.08 (dd, J = 14.0, 8.9 Hz, 1H), 2.53 (s, 3H), 1.87 – 1.73 (m, 2H), 1.64 – 1.52 (m, 1H), 1.50 – 1.40 (m, 1H), 1.39 – 1.29 (m, 2H), 0.90 (t, J = 7.3 Hz, 3H). **^{13}C NMR** (100 MHz, $CDCl_3$) δ 158.19, 157.59, 136.59, 121.43, 121.23, 63.31, 47.44, 38.14, 28.68, 24.65, 22.36, 14.11. **HRMS** (m/z) $[M+H]^+$ calcd. for $C_{12}H_{19}ClN$, 212.1201, found 212.1202.

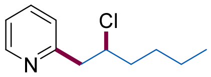

2-(2-Chlorohexyl)pyridine (Product 3ma):

Colorless oil, 85.0 mg, 86% yield. **^{1}H NMR** (400 MHz, $CDCl_3$) δ 8.55 (d, J = 4.7 Hz, 1H), 7.62 (td, J = 7.7, 1.6 Hz, 1H), 7.20 (d, J = 7.7 Hz, 1H), 7.15 (dd, J = 7.3, 5.1 Hz, 1H), 4.43 – 4.33 (m, 1H), 3.22 (dd, J = 14.0, 4.8 Hz, 1H), 3.11 (dd, J = 14.0, 8.9 Hz, 1H), 1.88 – 1.71 (m, 2H), 1.64 – 1.51 (m, 1H), 1.50 – 1.40 (m, 1H), 1.39 – 1.29 (m, 2H), 0.90 (t, J = 7.3 Hz, 3H). **^{13}C NMR** (100 MHz, $CDCl_3$) δ 158.29, 149.54, 136.43, 124.33, 121.88, 63.00, 47.31, 38.16, 28.71, 22.33, 14.09. **HRMS** (m/z) $[M+H]^+$ calcd. for $C_{11}H_{17}ClN$, 198.1044, found 198.1046.

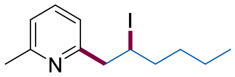

2-(2-Iodohexyl)-6-methylpyridine (Product 3na):

Colorless oil, 94.0 mg, 62% yield. **^{1}H NMR** (400 MHz, $CDCl_3$) δ 7.51 (t, J = 7.6 Hz, 1H), 7.03 (d, J = 7.7 Hz, 1H), 6.97 (d, J = 7.6 Hz, 1H), 4.58 – 4.48 (m, 1H), 3.31 (d, J = 7.3 Hz, 2H), 2.54 (s, 3H), 1.93 – 1.83 (m, 1H), 1.81 – 1.72 (m, 1H), 1.64 – 1.56 (m, 1H), 1.48 – 1.41 (m, 1H), 1.38 – 1.29 (m, 2H), 0.91 (t, J = 7.2 Hz, 3H). **^{13}C NMR** (100 MHz, $CDCl_3$) δ 158.80, 158.25, 136.64, 121.50, 120.92, 49.52, 40.10, 38.22, 31.87, 24.64, 22.07, 14.11. **HRMS** (m/z) $[M+H]^+$ calcd. for $C_{12}H_{19}IN$, 304.0557, found 304.0560.

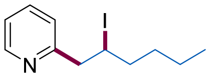

2-(2-Iodohexyl)pyridine (Product 3oa):

Colorless oil, 96.9 mg, 67% yield. **^{1}H NMR** (400 MHz, $CDCl_3$) δ 8.61 – 8.51 (m, 1H), 7.63 (td, J = 7.7, 1.9 Hz, 1H), 7.20 – 7.15 (m, 2H), 4.59 – 4.51 (m, 1H), 3.40 – 3.29 (m, 2H), 1.96 – 1.86 (m, 1H), 1.83 – 1.74 (m, 1H), 1.64 – 1.59 (m, 1H), 1.50 – 1.41 (m, 1H), 1.40 – 1.30 (m, 2H), 0.91 (t, J = 7.2 Hz, 3H). **^{13}C NMR** (100 MHz, $CDCl_3$) δ 159.54, 149.62, 136.51, 124.02, 121.97, 49.45, 40.24, 37.53, 31.90, 22.06, 14.11. **HRMS** (m/z) $[M+H]^+$ calcd. for $C_{11}H_{17}IN$, 290.0400, found 290.0407.

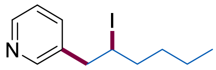

3-(2-Iodohexyl)pyridine (Product 3pa):

Colorless oil, 118.6 mg, 82% yield. **^{1}H NMR** (400 MHz, $CDCl_3$) δ 8.51 (dd, J = 4.8, 1.6 Hz, 1H), 8.46 (d, J = 1.7 Hz, 1H), 7.53 (dt, J = 7.8, 1.8 Hz, 1H), 7.26 –

7.21 (m, 1H), 4.23 – 4.14 (m, 1H), 3.24 – 3.13 (m, 2H), 1.89 – 1.78 (m, 1H), 1.76 – 1.66 (m, 1H), 1.62 – 1.50 (m, 1H), 1.45 – 1.26 (m, 3H), 0.89 (t, *J* = 7.2 Hz, 3H). **^{13}C NMR** (100 MHz, $CDCl_3$) δ 150.38, 148.28, 136.73, 135.28, 123.45, 44.29, 39.63, 37.61, 31.83, 21.96, 14.04. **HRMS** (m/z) $[M+H]^+$ calcd. for $C_{11}H_{17}IN$, 290.0400, found 290.0405.

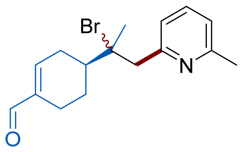

(4S)-4-(2-Bromo-1-(6-methylpyridin-2-yl)propan-2-yl)cyclohex-1-ene-1-carbaldehyde (Product 4a):

Colorless oil, 120.8 mg, 75% yield, mixture of diastereomers (*dr* = 1:1). **^{1}H NMR** (400 MHz, $CDCl_3$) δ 9.47 – 9.41 (m, 1H), 7.54 – 7.48 (m, 1H), 7.16 (d, *J* = 7.7 Hz, 0.5H), 7.11 (d, *J* = 7.6 Hz, 0.5H), 7.03 (d, *J* = 7.5 Hz, 1H), 6.85 – 6.81 (m, 0.5H), 6.81 – 6.76 (m, 0.5H), 3.57 (d, *J* = 13.6 Hz, 0.5H), 3.46 (d, *J* = 13.7 Hz, 1H), 3.40 (d, *J* = 13.8 Hz, 0.5H), 2.68 – 2.56 (m, 1H), 2.56 – 2.51 (m, 1H), 2.50 (s, 3H), 2.47 – 2.40 (m, 1H), 2.30 – 2.24 (m, 0.5H), 2.11 – 2.05 (m, 0.5H), 2.05 – 1.96 (m, 1H), 1.81 (s, 1.5H), 1.78 (s, 1.5H), 1.76 – 1.64 (m, 1H), 1.52 – 1.35 (m, 1H). **^{13}C NMR** (100 MHz, $CDCl_3$) δ 193.77, 157.81, 157.69, 156.62, 156.54, 150.61, 150.27, 141.61, 141.30, 136.36, 136.30, 122.50, 122.28, 121.56, 121.55, 75.82, 75.55, 51.47, 51.19, 44.70, 44.31, 30.35, 29.68, 29.60, 29.26, 25.00, 24.68, 24.66, 24.33, 21.97, 21.88. **HRMS** (m/z) $[M+H]^+$ calcd. for $C_{16}H_{21}BrNO$, 322.0807, found 322.0816.

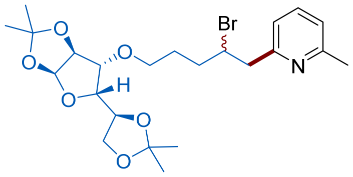

2-(2-Bromo-5-(((3aS,5S,6R,6aS)-5-((S)-2,2-dimethyl-1,3-dioxolan-4-yl)-2,2-dimethyltetrahydrofuro[2,3-d][1,3]dioxol-6-yl)oxy)pentyl)-6-methylpyridine (Product 4b):

Colorless oil, 200.2 mg, 80% yield, mixture of diastereomers (*dr* = 1:1). **^{1}H NMR** (400 MHz, $CDCl_3$) δ 7.46 (t, *J* = 7.7 Hz, 1H), 7.01 – 6.92 (m, 2H), 5.81 (t, *J* = 3.1 Hz, 1H), 4.50 – 4.42 (m, 2H), 4.26 – 4.19 (m, 1H), 4.08 – 4.05 (m, 1H), 4.04 – 3.99 (m, 1H), 3.95 – 3.90 (m, 1H), 3.81 (d, *J* = 3.0 Hz, 1H), 3.65 – 3.57 (m, 1H), 3.56 – 3.48 (m, 1H), 3.29 – 3.15 (m, 2H), 2.49 (s, 3H), 2.03 – 1.95 (m, 1H), 1.94 – 1.82 (m, 2H), 1.77 – 1.66 (m, 1H), 1.45 (s, 3H), 1.38 (s, 3H), 1.30 (s, 3H), 1.27 (s, 3H). **^{13}C NMR** (100 MHz, $CDCl_3$) δ 158.15, 158.14, 157.58, 157.56, 136.51, 121.42, 120.94, 111.75, 108.94, 105.29, 82.51, 82.23, 82.14, 81.24, 72.51, 69.88, 69.73, 67.28, 56.38, 56.26, 47.86, 35.53, 35.42, 27.91, 27.83, 26.90, 26.84, 26.31, 25.47, 24.53. **HRMS** (m/z) $[M+H]^+$ calcd. for $C_{23}H_{35}BrNO_6$, 500.1648, found 500.1672.

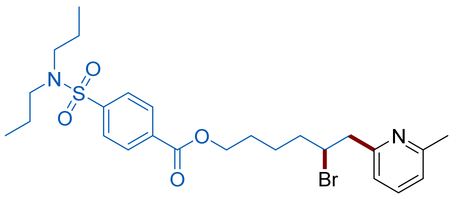

5-Bromo-6-(6-methylpyridin-2-yl)hexyl 4-(N,N-Dipropylsulfamoyl)benzoate (Product 4c):

Colorless oil, 229.3 mg, 85% yield. **^{1}H NMR** (400 MHz, $CDCl_3$) δ 8.12 (d, *J* = 8.2 Hz, 2H), 7.84 (d, *J* = 8.2 Hz, 2H), 7.47 (t, *J* = 7.6 Hz, 1H), 6.99 (d, *J* = 7.8 Hz, 1H), 6.96 (d, *J* = 7.7 Hz, 1H), 4.53 – 4.43 (m, 1H), 4.33 (t, *J* = 5.7 Hz, 2H), 3.27 (dd, *J* = 14.2, 5.6 Hz, 1H), 3.21 (dd, *J* = 14.2, 8.6 Hz, 1H), 3.07 (t, *J* = 7.6 Hz, 4H), 2.49 (s, 3H), 2.01 – 1.86 (m, 2H), 1.86 – 1.72 (m, 3H), 1.68 – 1.59 (m, 1H), 1.56 – 1.46 (m, 4H), 0.83 (t, *J* = 7.3 Hz, 6H). **^{13}C NMR** (100 MHz, $CDCl_3$) δ 165.25, 158.16, 157.52, 144.21, 136.58, 133.68, 130.23, 127.02, 121.48, 121.01, 65.35, 56.30, 49.97, 47.86, 38.28, 28.02, 24.55, 24.15, 21.97, 11.21. **HRMS** (m/z) $[M+H]^+$ calcd. for $C_{25}H_{36}BrN_2O_4S$, 539.1579, found 539.1586.

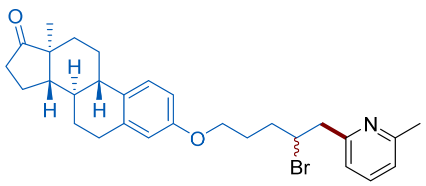

(8R,9S,13S,14S)-3-((4-Bromo-5-(6-methylpyridin-2-yl)pentyl)oxy)-13-methyl-6,7,8,9,11,12,13,14,15,16-decahydro-17H-cyclopenta[a]phenanthren-17-one (Product 4d): White solid, m. p. 98-99 ℃, 171.0 mg, 67% yield, mixture of diastereomers (*dr* = 1:1). **^{1}H NMR** (400 MHz, $CDCl_3$) δ 7.52 (t, *J* = 7.6 Hz, 1H), 7.19 (d, *J* = 8.6 Hz, 1H), 7.02 (t, *J* = 8.4 Hz, 3H), 6.73 – 6.65 (m, 1H), 6.66 – 6.57 (m, 1H), 4.61 – 4.48 (m, 1H), 4.03 – 3.92 (m, 2H), 3.34 (dd, *J* = 14.2, 5.3 Hz, 1H), 3.26 (dd, *J* = 14.2, 8.8 Hz, 1H), 2.94 – 2.83 (m, 2H), 2.53 (s, 3H), 2.54 – 2.44 (m, 1H), 2.44 – 2.35 (m, 1H), 2.31 – 2.19 (m, 1H), 2.21 – 1.90 (m, 7H), 1.70 – 1.36 (m, 7H), 0.91 (s, 3H). **^{13}C NMR** (100 MHz, $CDCl_3$) δ 221.03, 158.12, 157.51, 157.03, 137.81, 136.90, 132.13, 126.41, 121.69, 121.27, 114.65, 112.25, 67.12, 56.35, 50.52, 48.12, 47.75, 44.09, 38.49, 35.99, 35.59, 31.70, 29.77, 27.64, 26.67, 26.04, 24.43, 21.70, 13.98. **HRMS** (m/z) $[M+H]^+$ calcd. for $C_{29}H_{37}BrNO_2$, 510.2008, found 510.1944.

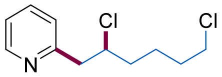

2-(2,6-Dichlorohexyl)pyridine (Product 5a): Faint yellow oil, 88.2 mg, 76% yield. **^{1}H NMR** (400 MHz, $CDCl_3$) δ 8.58 – 8.51 (m, 1H), 7.62 (td, *J* = 7.7, 1.8 Hz, 1H), 7.22 – 7.13 (m, 2H), 4.44 – 4.35 (m, 1H), 3.52 (t, *J* = 6.4 Hz, 2H), 3.22 (dd, *J* = 14.0, 5.2 Hz, 1H), 3.13 (dd, *J* = 14.0, 8.6 Hz, 1H), 1.90 – 1.71 (m, 5H), 1.68 – 1.55 (m, 1H). **^{13}C NMR** (100 MHz, $CDCl_3$) δ 157.97, 149.57, 136.49, 124.32, 121.97, 62.38, 47.18, 44.82, 37.49, 32.12, 23.94. **HRMS** (m/z) $[M+H]^+$ calcd. for $C_{11}H_{16}Cl_2N$, 232.0654, found 232.0655.

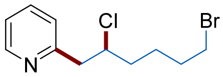

2-(6-Bromo-2-chlorohexyl)pyridine (Product 5b): Faint yellow oil, 107.9 mg, 78% yield. **^{1}H NMR** (400 MHz, $CDCl_3$) δ 8.57 – 8.52 (m, 1H), 7.62 (td, *J* = 7.7, 1.8 Hz, 1H), 7.20 (d, *J* = 7.8 Hz, 1H), 7.18 – 7.13 (m, 1H), 4.44 – 4.34 (m, 1H), 3.39 (t, *J* = 6.7 Hz, 2H), 3.21 (dd, *J* = 14.0, 5.2 Hz, 1H), 3.13 (dd, *J* = 14.0, 8.5 Hz, 1H), 1.93 – 1.72 (m, 5H), 1.67 – 1.55 (m, 1H). **^{13}C NMR** (100 MHz, $CDCl_3$) δ 157.95, 149.57, 136.48, 124.31, 121.96, 62.33, 47.18, 37.35, 33.46, 32.27, 25.21. **HRMS** (m/z) $[M+H]^+$ calcd. for $C_{11}H_{16}BrClN$, 276.0149, found 276.0147.

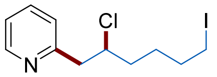

2-(2-Chloro-6-iodohexyl)pyridine (Product 5c): Pale brown oil, 103.6 mg, 64% yield. **^{1}H NMR** (400 MHz, $CDCl_3$) δ 8.63 – 8.49 (m, 1H), 7.63 (td, *J* = 7.6, 1.9 Hz, 1H), 7.24 – 7.11 (m, 2H), 4.44 – 4.34 (m, 1H), 3.26 – 3.10 (m, 4H), 1.93 – 1.79 (m, 4H), 1.75 – 1.67 (m, 1H), 1.65 – 1.53 (m, 1H). **^{13}C NMR** (100 MHz, $CDCl_3$) δ 157.96, 149.56, 136.56, 124.38, 122.01, 62.34, 47.19, 37.16, 33.03, 27.54, 6.42. **HRMS** (m/z) $[M+H]^+$ calcd. for $C_{11}H_{16}ClIN$, 324.0010, found 324.0014.

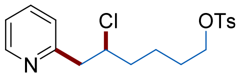

5-Chloro-6-(pyridin-2-yl)hexyl 4-methylbenzenesulfonate (Product 5d): Faint yellow oil, 147.2 mg, 80% yield. **^{1}H NMR** (400 MHz, $CDCl_3$) δ 8.53 – 8.48 (m, 1H), 7.77 – 7.73 (m, 2H), 7.60 (td, *J* = 7.7, 1.8 Hz, 1H), 7.31 (d, *J* = 8.0 Hz, 2H), 7.18 – 7.11 (m, 2H), 4.46 – 4.34 (m, 1H), 3.99 (t, *J* = 6.2 Hz, 2H), 3.26 (dd, *J* = 14.3, 5.5 Hz, 1H), 3.19 (dd, *J* = 14.3, 8.6 Hz, 1H), 2.40 (s, 3H), 1.86 – 1.72 (m, 2H), 1.71 – 1.54 (m, 3H), 1.53 – 1.38 (m, 1H). **^{13}C NMR** (100 MHz, $CDCl_3$) δ 158.11, 149.38, 144.79, 136.50, 133.05, 129.90, 127.87,

124.08, 121.93, 70.23, 55.52, 47.53, 37.96, 28.17, 23.55, 21.66. **HRMS** (m/z) $[M+H]^+$ calcd. for $C_{18}H_{23}ClNO_3S$, 368.1082, found 368.1084.

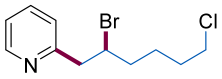

2-(2-Bromo-6-chlorohexyl)pyridine (Product 5e):

Faint yellow oil, 124.5 mg, 90% yield. **^{1}H NMR** (400 MHz, $CDCl_3$) δ 8.56 – 8.51 (m, 1H), 7.61 (td, *J* = 7.7, 1.8 Hz, 1H), 7.20 – 7.13 (m, 2H), 4.52 – 4.44 (m, 1H), 3.51 (t, *J* = 6.4 Hz, 2H), 3.33 (dd, *J* = 14.3, 5.4 Hz, 1H), 3.25 (dd, *J* = 14.3, 8.7 Hz, 1H), 1.94 – 1.85 (m, 2H), 1.84 – 1.71 (m, 3H), 1.67 – 1.57 (m, 1H). **^{13}C NMR** (100 MHz, $CDCl_3$) δ 158.31, 149.53, 136.48, 124.16, 121.97, 55.77, 47.73, 44.76, 38.05, 31.95, 25.03. **HRMS** (m/z) $[M+H]^+$ calcd. for $C_{11}H_{16}BrClN$, 276.0149, found 276.0153.

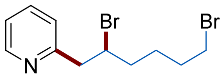

2-(2,6-Dibromohexyl)pyridine (Product 5f):

Pale brown oil, 149.3 mg, 93% yield. **^{1}H NMR** (400 MHz, $CDCl_3$) δ 8.57 – 8.48 (m, 1H), 7.60 (td, *J* = 7.7, 1.8 Hz, 1H), 7.20 – 7.10 (m, 2H), 4.52 – 4.42 (m, 1H), 3.37 (t, *J* = 6.7 Hz, 2H), 3.32 (dd, *J* = 14.2, 5.4 Hz, 1H), 3.24 (dd, *J* = 14.3, 8.6 Hz, 1H), 1.95 – 1.69 (m, 5H), 1.66 – 1.54 (m, 1H). **^{13}C NMR** (100 MHz, $CDCl_3$) δ 158.26, 149.51, 136.45, 124.12, 121.94, 55.69, 47.70, 37.88, 33.39, 32.08, 26.27. **HRMS** (m/z) $[M+H]^+$ calcd. for $C_{11}H_{16}Br_2N$, 319.9644, found 319.9647.

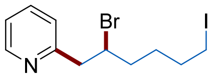

2-(2-Bromo-6-iodohexyl)pyridine (Product 5g):

Pale brown oil, 125.1 mg, 68% yield. **^{1}H NMR** (400 MHz, $CDCl_3$) δ 8.55 (d, *J* = 4.9 Hz, 1H), 7.63 (td, *J* = 7.7, 1.9 Hz, 1H), 7.24 – 7.12 (m, 2H), 4.54 – 4.43 (m, 1H), 3.35 (dd, *J* = 14.4, 5.2 Hz, 1H), 3.26 (dd, *J* = 14.3, 8.7 Hz, 1H), 3.18 (t, *J* = 6.9 Hz, 2H), 1.96 – 1.80 (m, 4H), 1.80 – 1.68 (m, 1H), 1.66 – 1.54 (m, 1H). **^{13}C NMR** (100 MHz, $CDCl_3$) δ 158.34, 149.57, 136.58, 124.25, 122.04, 55.74, 47.77, 37.76, 32.90, 28.65, 6.38. **HRMS** (m/z) $[M+H]^+$ calcd. for $C_{11}H_{16}BrIN$ 367.9505, found 367.9508.

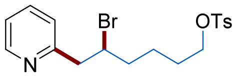

5-Bromo-6-(pyridin-2-yl)hexyl 4-methylbenzenesulfonate (Product 5h):

Faint yellow oil, 179.4 mg, 87% yield. **^{1}H NMR** (400 MHz, $CDCl_3$) δ 8.60 – 8.47 (m, 1H), 7.77 (d, *J* = 8.3 Hz, 2H), 7.62 (td, *J* = 7.7, 1.8 Hz, 1H), 7.33 (d, *J* = 8.1 Hz, 2H), 7.22 – 7.11 (m, 2H), 4.38 – 4.27 (m, 1H), 4.02 (t, *J* = 6.2 Hz, 2H), 3.16 (dd, *J* = 14.1, 5.3 Hz, 1H), 3.08 (dd, *J* = 14.1, 8.5 Hz, 1H), 2.43 (s, 3H), 1.82 – 1.57 (m, 5H), 1.52 – 1.39 (m, 1H). **^{13}C NMR** (100 MHz, $CDCl_3$) δ 157.85, 149.51, 144.85, 136.53, 133.19, 129.96, 127.98, 124.29, 121.98, 70.32, 62.20, 47.08, 37.48, 28.40, 22.52, 21.74. **HRMS** (m/z) $[M+H]^+$ calcd. for $C_{18}H_{23}BrNO_3S$, 412.0577, found 412.0581.

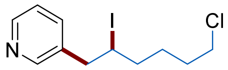

3-(6-Chloro-2-iodohexyl)pyridine (Product 5i):

Pale brown oil, 144.0 mg, 89% yield. **^{1}H NMR** (400 MHz, $CDCl_3$) δ 8.51 (dd, *J* = 4.8, 1.5 Hz, 1H), 8.46 (d, *J* = 2.0 Hz, 1H), 7.53 (dt, *J* = 7.8, 1.8 Hz, 1H), 7.27 – 7.22 (m, 1H), 4.23 – 4.15 (m, 1H), 3.52 (t, *J* = 6.3 Hz, 2H), 3.27 – 3.14 (m, 2H), 1.90 – 1.68 (m, 5H), 1.65 – 1.51 (m, 1H). **^{13}C NMR** (100 MHz, $CDCl_3$) δ 150.33, 148.36, 136.64, 135.00, 123.47, 44.72, 44.27, 39.04, 36.55, 31.73, 27.14. **HRMS** (m/z) $[M+H]^+$ calcd. for $C_{11}H_{16}ClIN$, 324.0010, found 324.0015.

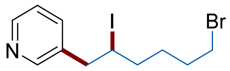

3-(6-Bromo-2-iodohexyl)pyridine (Product 5j):

Pale brown oil, 169.3 mg, 92% yield. **^{1}H NMR** (400 MHz, $CDCl_3$) δ 8.53 – 8.48 (m, 1H), 8.48 – 8.42 (m, 1H), 7.52 (dt, *J* = 7.8, 2.0 Hz, 1H), 7.26 – 7.21 (m, 1H), 4.24 – 4.12 (m, 1H), 3.38 (t, *J* = 6.6 Hz, 2H), 3.26 – 3.14 (m, 2H), 2.01 – 1.76 (m, 4H), 1.75 – 1.68 (m, 1H), 1.66 – 1.51 (m, 1H). **^{13}C NMR** (100 MHz, $CDCl_3$) δ 150.35, 148.39, 136.57, 134.93, 123.44, 44.24, 38.87, 36.48, 33.33, 31.85, 28.36. **HRMS** (m/z) $[M+H]^+$ calcd. for $C_{11}H_{16}BrIN$, 367.9505, found 367.9503.

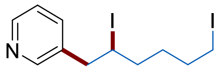

3-(2,6-Diiodohexyl)pyridine (Product 5k):

Pale brown oil, 134.9 mg, 65% yield. **^{1}H NMR** (400 MHz, $CDCl_3$) δ 8.54 (d, *J* = 4.6 Hz, 1H), 8.48 (s, 1H), 7.56 (d, *J* = 7.8 Hz, 1H), 7.30 – 7.26 (m, 1H), 4.24 – 4.14 (m, 1H), 3.31 – 3.13 (m, 4H), 1.93 – 1.79 (m, 3H), 1.79 – 1.67 (m, 2H), 1.61 – 1.49 (m, 1H). **^{13}C NMR** (100 MHz, $CDCl_3$) δ 150.38, 148.42, 136.70, 135.03, 123.53, 44.33, 38.71, 36.42, 32.63, 30.72, 6.23. **HRMS** (m/z) $[M+H]^+$ calcd. for $C_{11}H_{16}I_2N$, 415.9367, found 415.9370.

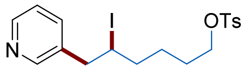

5-Iodo-6-(pyridin-3-yl)hexyl 4-methylbenzenesulfonate (Product 5l):

Pale brown oil, 197.5 mg, 86% yield. **^{1}H NMR** (400 MHz, $CDCl_3$) δ 8.53 (d, *J* = 4.2 Hz, 1H), 8.44 (s, 1H), 7.79 (d, *J* = 8.2 Hz, 2H), 7.52 (d, *J* = 7.7 Hz, 1H), 7.35 (d, *J* = 8.0 Hz, 2H), 7.26 – 7.25 (m, 1H), 4.16 – 4.08 (m, 1H), 4.03 (t, *J* = 5.7 Hz, 2H), 3.25 – 3.07 (m, 2H), 2.44 (s, 3H), 1.84 – 1.75 (m, 1H), 1.74 – 1.67 (m, 2H), 1.66 – 1.52 (m, 2H), 1.51 – 1.38 (m, 1H). **^{13}C NMR** (100 MHz, $CDCl_3$) δ 150.36, 148.44, 144.94, 136.65, 134.97, 133.20, 130.02, 128.03, 123.52, 70.19, 44.30, 39.14, 36.44, 28.14, 25.90, 21.80. **HRMS** (m/z) $[M+H]^+$ calcd. for $C_{18}H_{23}INO_3S$, 460.0438, found 460.0439.

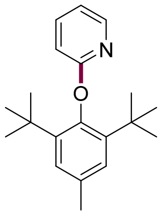

2-(2,6-Di-tert-butyl-4-methylphenoxy)pyridine (Product 6):

Colorless oil, 68.4 mg, 46% yield. **^{1}H NMR** (400 MHz, $CDCl_3$) δ 8.60 (ddd, *J* = 4.9, 1.9, 0.9 Hz, 1H), 7.56 (td, *J* = 7.7, 1.9 Hz, 1H), 7.15 (ddd, *J* = 7.5, 4.8, 1.1 Hz, 1H), 7.06 (d, *J* = 7.9 Hz, 1H), 6.76 (s, 2H), 1.67 (s, 3H), 1.25 (s, 18H). **^{13}C NMR** (100 MHz, $CDCl_3$) δ 186.39, 161.74, 149.54, 145.75, 145.70, 136.78, 122.11, 121.24, 46.41, 34.97, 29.64, 25.60. **HRMS** (m/z) $[M+H]^+$ calcd. for $C_{20}H_{28}NO$, 298.2165, found 298.2168.

2.6. Gram-scale reactions

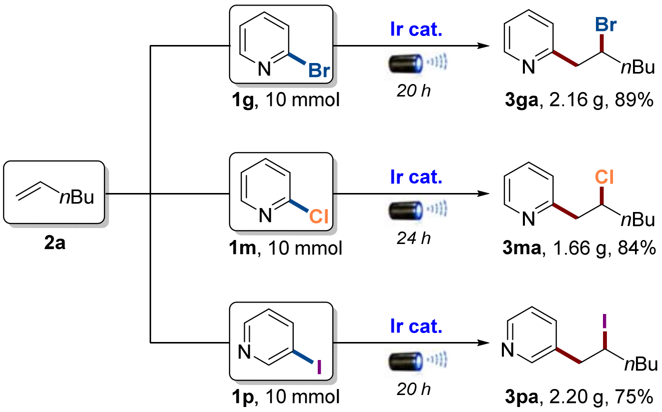

To an oven-dried 100 mL vial was added Ir(ppy)$_2$(dtbbpy)PF$_6$ (0.1 mmol, 1.0 mol%), halopyridines **1g**, **1m** or **1p** (10.0 mmol, 1.0 equiv.), alkenes **2a** (30.0 mmol, 3.0 equiv.), TFA (10.0 mmol, 1.0 equiv.) and TFE (50 mL, 0.2 M) in the nitrogen glove box. The vial was capped with a septum and wrapped with parafilm. The reaction mixture was stirred for 20-24 h under visible light irradiation (2 x Kessil PR160, λ_{max}= 456 nm, 40 W, irradiation temperature maintained between 25-30 ℃). After reaction completed, the crude product was neutralized with saturated $NaHCO_3$ solution or Et_3N and extracted with ethyl acetate. Organic layer was washed with brine solution and dried over anhydrous Na_2SO_4. Removal of the organic solvent in a vacuum rotavapor followed by flash silica gel column chromatographic purification (hexane/ethyl acetate) afforded the desired products **3ga**, **3ma** and **3pa** in 89% (2.16 g), 84% (1.66 g) and 75% (2.20 g) yields, respectively. The reaction products need to be stored in the refrigerator to prevent deterioration.

2.7. Further transformations

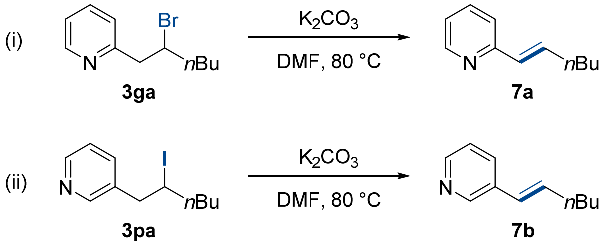

A flame-dried Schlenk flask was charged with **3ga** or **3pa** (0.2 mmol, 1.0 equiv) and K_2CO_3 (0.4 mmol, 2.0 equiv), dissolved in anhydrous DMF (2.0 mL, 0.1 M) and magnetically stirred under atmosphere of nitrogen at 80 °C for 8 h. The reaction was monitored by TLC. Afterwards, the resulting solution was washed by water and brine, dried over Na_2SO_4. And removal of the organic solvent in a vacuum rotavapor followed by flash silica gel column chromatographic purification (hexane/ethyl acetate) afforded the desired product **7a** or **7b**.

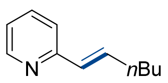

2-(Hex-1-en-1-yl)pyridine (Product 7a):

Colorless oil, 31.6 mg, 98% yield. **^{1}H NMR** (400 MHz, CDCl3) δ 8.52 (d, *J* = 4.1 Hz, 1H), 7.60 (td, *J* = 7.8, 1.8 Hz, 1H), 7.26 – 7.22 (m, 1H), 7.08 (dd, *J* = 7.5, 5.0 Hz, 1H), 6.74 (dt, *J* = 15.7, 7.0 Hz, 1H), 6.48 (d, *J* = 15.7 Hz, 1H), 2.32 – 2.20 (m, 2H), 1.54 – 1.45 (m, 2H), 1.43 – 1.35 (m, 2H), 0.92 (t, *J* = 7.2 Hz, 3H). **^{13}C NMR** (100 MHz, CDCl3) δ 156.29, 149.47, 136.57, 136.32, 129.92, 121.65, 121.08, 32.68, 31.26, 22.45, 14.09. **HRMS** (m/z) calcd. for $C_{11}H_{16}N$ $[M+H]^+$ 162.1277, found 162.1278.

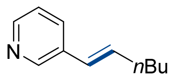

3-(Hex-1-en-1-yl)pyridine (Product 7b):

Colorless oil, 30.3 mg, 94% yield. **^{1}H NMR** (400 MHz, CDCl3) δ 8.95 – 8.03 (m, 2H), 7.64 (d, *J* = 7.9 Hz, 1H), 7.25 – 7.12 (m, 1H), 6.39 – 6.23 (m, 2H), 2.26 – 2.19 (m, 2H), 1.50 – 1.41 (m, 2H), 1.41 – 1.31 (m, 2H), 0.92 (t, *J* = 7.2 Hz, 3H). **^{13}C NMR** (100 MHz, CDCl3) δ 148.00, 147.91, 133.84, 132.48, 126.31, 32.94, 31.41, 22.39, 14.05. **HRMS** (m/z) calcd. for $C_{11}H_{16}N$ $[M+H]^+$162.1277, found 162.1278.

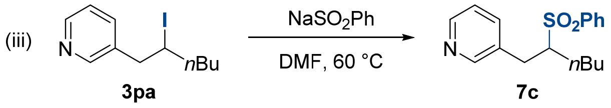

A flame-dried Schlenk flask was charged with **3pa** (0.2 mmol, 1.0 equiv) and $NaSO_2Ph$ (0.4 mmol, 2.0 equiv), dissolved in anhydrous DMF (2.0 mL, 0.1 M) and magnetically stirred under atmosphere of nitrogen at 60 °C for 3 h. The reaction was monitored by TLC. Afterwards, the resulting solution was washed by water and brine, dried over Na_2SO_4. And removal of the organic solvent in a vacuum rotavapor followed by flash silica gel column chromatographic purification (hexane/ethyl acetate) afforded the desired product **7c**.

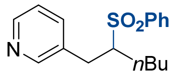

3-(2-(Phenylsulfonyl)hexyl)pyridine (Product 7c):

Colorless oil, 39.4 mg, 65% yield. **^{1}H NMR** (400 MHz, CDCl3) δ 8.46 (s, 1H), 8.36 (s, 1H), 7.89 (d, *J* = 7.5 Hz, 2H), 7.66 (t, *J* = 7.4 Hz, 1H), 7.57 (t, *J* = 7.6 Hz, 2H), 7.46 (d, *J* = 7.6 Hz, 1H), 7.22 – 7.16 (m, 1H), 3.29 (dd, *J* = 14.3, 4.3 Hz, 1H), 3.24 – 3.16 (m, 1H), 2.80 (dd, *J* = 14.3, 8.6 Hz, 1H), 1.89 – 1.78 (m, 1H), 1.62 – 1.51 (m, 1H), 1.35 – 1.27 (m, 1H), 1.25 – 1.20 (m, 1H), 1.19 – 1.07 (m, 2H), 0.75 (t, *J* = 7.2 Hz, 3H). **^{13}C NMR** (100 MHz, CDCl3) δ 150.31, 148.40, 137.99, 136.61, 133.97, 129.42, 128.94, 65.67, 31.87, 28.99, 27.66, 22.50, 13.70. **HRMS** (m/z) calcd. for $C_{17}H_{22}NSO_2$ $[M+H]^+$ 304.1366, found 304.1370.

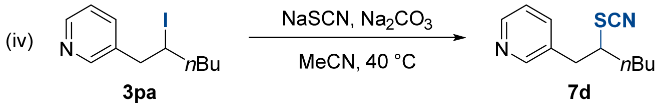

A flame-dried Schlenk flask was charged with **3pa** (0.2 mmol, 1.0 equiv), NaSCN (0.4 mmol, 2.0 equiv) and Na_2CO_3 (0.3 mmol, 1.5 equiv), dissolved in anhydrous MeCN (2.0 mL, 0.1 M) and magnetically stirred under atmosphere of nitrogen at 40 °C for 3 h. The reaction was monitored by TLC. Afterwards, the resulting solution was washed by water and brine, dried over Na_2SO_4. And removal of

the organic solvent in a vacuum rotavapor followed by flash silica gel column chromatographic purification (hexane/ethyl acetate) afforded the desired product **7d**.

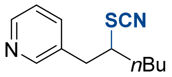

3-(2-Thiocyanatohexyl)pyridine (Product 7d):

Colorless oil, 28.2 mg, 64% yield. **^{1}H NMR** (400 MHz, CDCl3) δ 8.64 – 8.39 (m, 2H), 7.62 – 7.52 (m, 1H), 7.31 – 7.26 (m, 1H), 3.26 – 3.17 (m, 1H), 3.12 (dd, *J* = 14.4, 6.0 Hz, 1H), 3.03 (dd, *J* = 14.4, 8.3 Hz, 1H), 1.89 – 1.72 (m, 2H), 1.63 – 1.52 (m, 1H), 1.46 – 1.29 (m, 3H), 0.92 (t, *J* = 7.2 Hz, 3H). **^{13}C NMR** (100 MHz, CDCl3) δ 150.52, 148.83, 136.92, 123.70, 110.79, 52.36, 39.16, 34.54, 29.23, 22.27, 13.97. **HRMS** (m/z) calcd. for $C_{12}H_{17}N_2S$ $[M+H]^+$221.1107, found 221.1109.

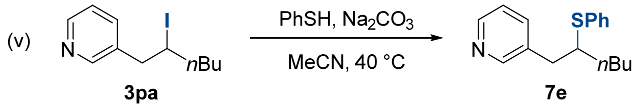

A flame-dried Schlenk flask was charged with **3pa** (0.2 mmol, 1.0 equiv), thiophenol (0.4 mmol, 2.0 equiv) and Na_2CO_3 (0.3 mmol, 1.5 equiv), dissolved in anhydrous MeCN (2.0 mL, 0.1 M) and magnetically stirred under atmosphere of nitrogen at 40 °C for 7 h. The reaction was monitored by TLC. Afterwards, the resulting solution was washed by water and brine, dried over Na_2SO_4. And removal of the organic solvent in a vacuum rotavapor followed by flash silica gel column chromatographic purification (hexane/ethyl acetate) afforded the desired product **7e**.

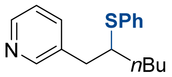

3-(2-(Phenylthio)hexyl)pyridine (Product 7e):

Colorless oil, 39.1 mg, 72% yield. **^{1}H NMR** (400 MHz, CDCl3) δ 8.57 – 8.32 (m, 2H), 7.52 – 7.45 (m, 1H), 7.40 – 7.32 (m, 2H), 7.30 – 7.26 (m, 2H), 7.25 – 7.13 (m, 2H), 3.35 – 3.23 (m, 1H), 2.90 (dd, *J* = 14.2, 6.5 Hz, 1H), 2.82 (dd, *J* = 14.1, 7.3 Hz, 1H), 1.64 – 1.39 (m, 4H), 1.32 – 1.24 (m, 2H), 0.86 (t, *J* = 7.3 Hz, 3H). **^{13}C NMR** (100 MHz, CDCl3) δ 150.72, 147.90, 136.88, 135.17, 132.25, 129.06, 127.10, 123.29, 50.52, 38.66, 33.55, 29.09, 22.62, 14.09. **HRMS** (m/z) calcd. for $C_{17}H_{22}NS$ $[M+H]^+$ 272.1467, found 272.1464.

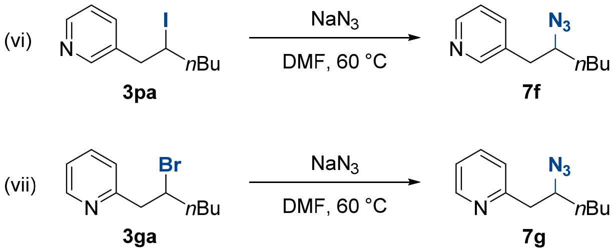

A flame-dried Schlenk flask was charged with **3ga** or **3pa** (0.2 mmol, 1.0 equiv) and NaN_3 (0.4 mmol, 2.0 equiv), dissolved in anhydrous DMF (2.0 mL, 0.1 M) and magnetically stirred under atmosphere of nitrogen at 60 °C for 10 h. The reaction was monitored by TLC. Afterwards, the resulting solution was washed by water and brine, dried over Na_2SO_4. And removal of the organic

solvent in a vacuum rotavapor followed by flash silica gel column chromatographic purification (hexane/ethyl acetate) afforded the desired product **7f** or **7g**.

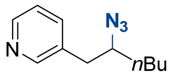

3-(2-Azidohexyl)pyridine (Product 7f):

Colorless oil, 34.7 mg, 85% yield. **^{1}H NMR** (400 MHz, CDCl3) δ 8.44 (s, 2H), 7.49 (d, *J* = 7.8 Hz, 1H), 7.22 – 7.16 (m, 1H), 3.48 – 3.37 (m, 1H), 2.77 (dd, *J* = 14.1, 5.2 Hz, 1H), 2.69 (dd, *J* = 14.1, 8.1 Hz, 1H), 1.55 – 1.45 (m, 2H), 1.45 – 1.35 (m, 1H), 1.34 – 1.17 (m, 3H), 0.85 (t, *J* = 7.0 Hz, 3H). **^{13}C NMR** (101 MHz, CDCl3) δ 150.65, 148.32, 136.87, 63.93, 38.22, 33.97, 28.36, 22.57, 14.08. **HRMS** (m/z) calcd. for $C_{11}H_{17}N_4$ $[M+H]^+$ 205.1448, found 205.1451.

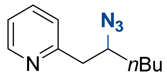

2-(2-Azidohexyl)pyridine (Product 7g):

Colorless oil, 24.9 mg, 61% yield. **^{1}H NMR** (400 MHz, CDCl3) δ 8.60 – 8.51 (m, 1H), 7.67 – 7.55 (m, 1H), 7.23 – 7.09 (m, 2H), 3.90 – 3.80 (m, 1H), 3.00 (dd, *J* = 13.8, 5.1 Hz, 1H), 2.91 (dd, *J* = 13.8, 8.7 Hz, 1H), 1.64 – 1.55 (m, 2H), 1.54 – 1.44 (m, 1H), 1.43 – 1.27 (m, 3H), 0.90 (t, *J* = 7.1 Hz, 3H). **^{13}C NMR** (100 MHz, CDCl3) δ 158.33, 149.64, 136.59, 124.11, 121.86, 63.07, 43.48, 34.34, 28.39, 22.58, 14.09. **HRMS** (m/z) calcd. for $C_{11}H_{17}N_4$ $[M+H]^+$ 205.1448, found 205.1451.

2.8. Mechanistic studies

2.8.1 Radical trapping experiments

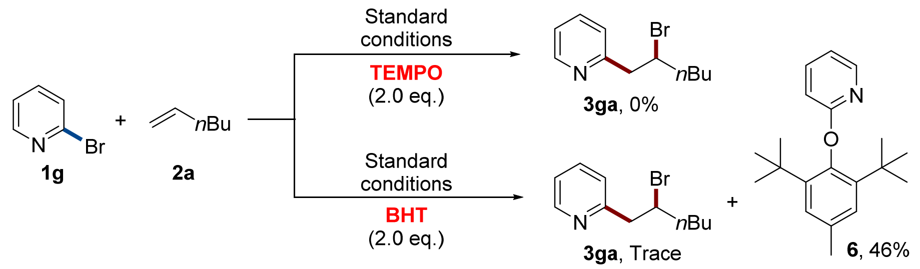

To an oven-dried 4 mL vial was added Ir(ppy)$_2$(dtbbpy)PF$_6$ (0.005 mmol, 1.0 mol%), halopyridines **1g** (0.5 mmol, 1.0 equiv.), alkenes **2a** (1.5 mmol, 3.0 equiv.), TFA (0.5 mmol, 1.0 equiv.), TFE (2.5 mL, 0.2 M) and radical scavenger **TEMPO** or **BHT** (1.0 mmol, 2.0 equiv.) in the nitrogen glove box. The vial was capped with a septum and wrapped with parafilm. The reaction mixture was stirred for 16 h under visible light irradiation (1 x Kessil PR160, λ_{max}= 456 nm, 40 W, irradiation temperature maintained between 25-30 ℃). After reaction completed, the crude product was neutralized with saturated $NaHCO_3$ solution or Et_3N and extracted with ethyl acetate. Organic layer was washed with brine solution and dried over anhydrous Na_2SO_4. Removal of the organic solvent in a vacuum rotavapor followed by flash silica gel column chromatographic purification (hexane/ethyl acetate) failed to afford the desired product **3ga** in acceptable yield, but with 46% yield of BHT-Pyridine adduct **6** isolated. The BHT-Pyridine adduct was confirmed by NMR and HRMS (m/z) analysis.

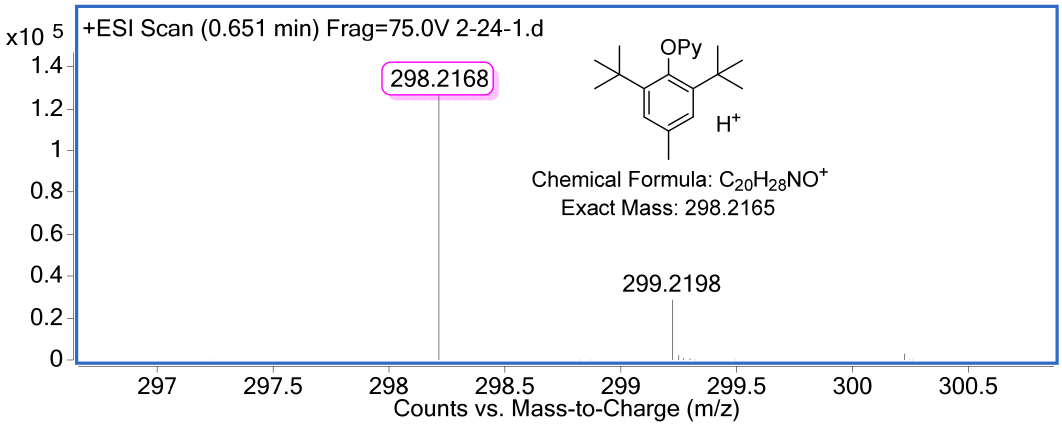

Supplementary Figure 2. HRMS (m/z) (ESI) spectra of BHT-Pyridine adduct

2.8.2 Stern-Volmer fluorescence quenching studies

Stern-Volmer experiments for all the components of the reaction mixture were carried out to monitor the emission intensity of solutions of Ir(ppy)$_2$(dtbbpy)PF$_6$ (0.01 mM) containing variable amounts of the quencher in trifluoroethanol (TFE). The emmision intensity at 555 nm was collected with excited wavelength of 365 nm in TFE using a PTI QM-400 Spectrofluorophotometer. After degassing the sample with a stream of argon for 10 minutes, the emission intensity of the sample was collected and plots were constructed according to the Stern-Volmer equation $I_0/I = 1 + Kqt_0[Q]$.

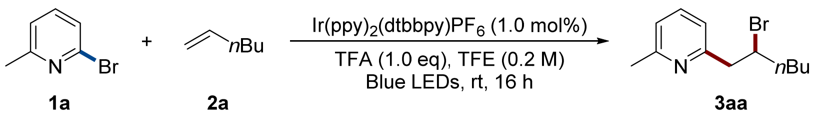

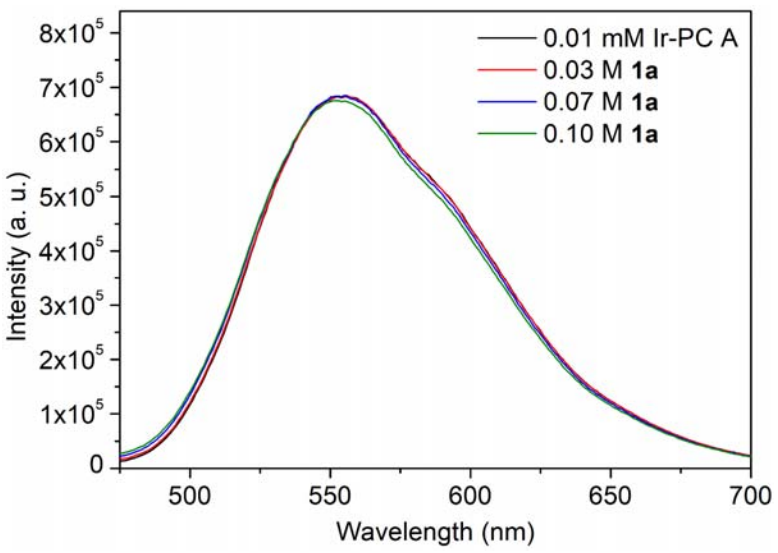

Supplementary Figure 3. Ir(ppy)$_2$(dtbbpy)PF$_6$ emission quenching with 1a

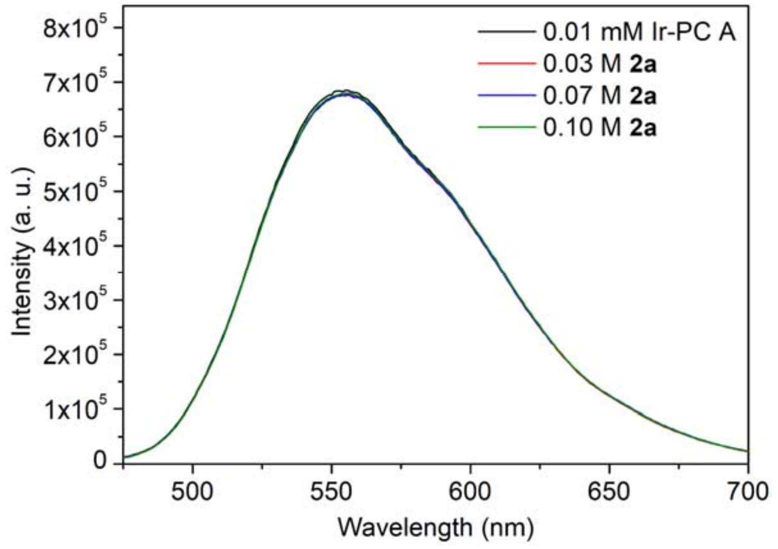

Supplementary Figure 4. Ir(ppy)$_2$(dtbbpy)PF$_6$ emission quenching with 2a

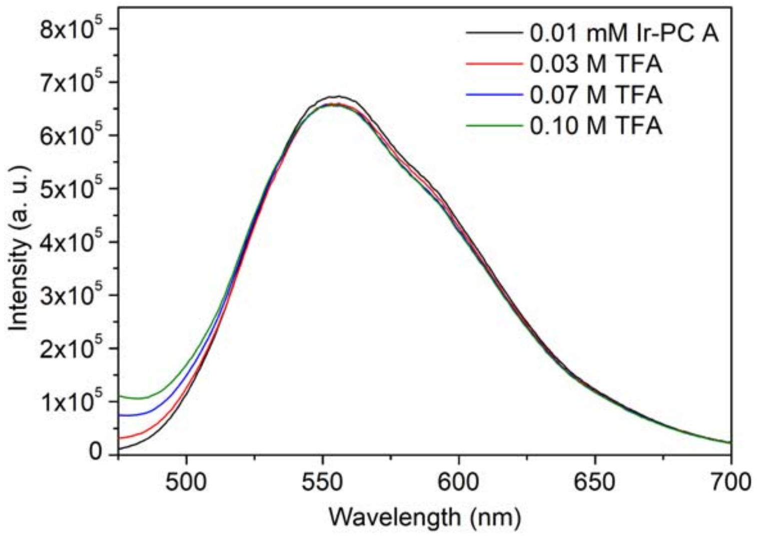

Supplementary Figure 5. Ir(ppy)$_2$(dtbbpy)PF$_6$ emission quenching with TFA

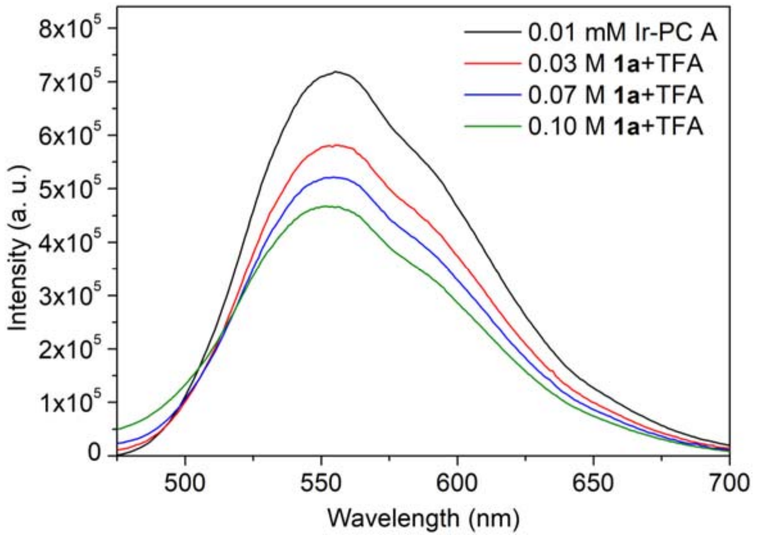

Supplementary Figure 6. Ir(ppy)$_2$(dtbbpy)PF$_6$ emission quenching with combine of 1a and TFA

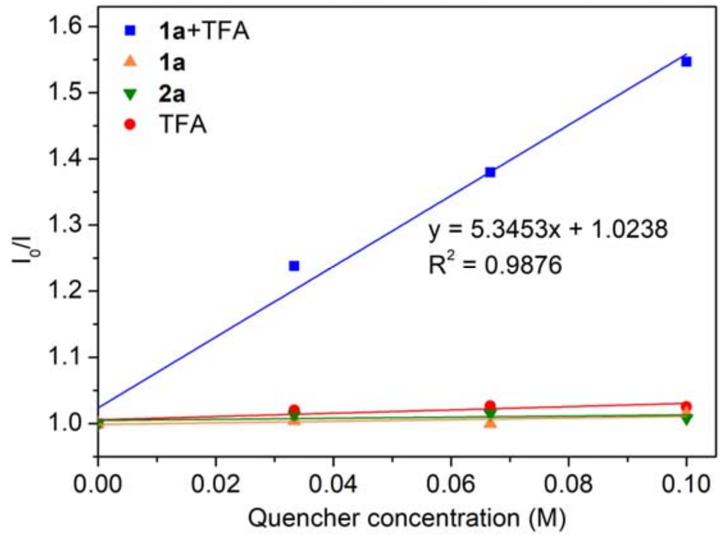

Supplementary Figure 7. Stern-Volmer quenching studies

2.8.3 Cyclic Voltammetry

A standard three-electrode cell configuration was used to collect cyclic voltammograms at room temperature with a CHI660e electrochemical workstation. A 3 mm glassy carbon disc electrode, a Ag/AgCl (3 M KCl) electrode, and a platinum wire were used as the working electrode, reference electrode, and counter electrode, respectively. The electrolyte solution contains 0.1 M tetrabutylammonium hexafluorophosphate ($TBAPF_6$). Measurement of the reduction potential was tested by cathodic reduction scan. For comparison with reported redox potentials of the photosensitizers, the reference electrode potential was converted to the saturated calomel electrode (SCE) scale.

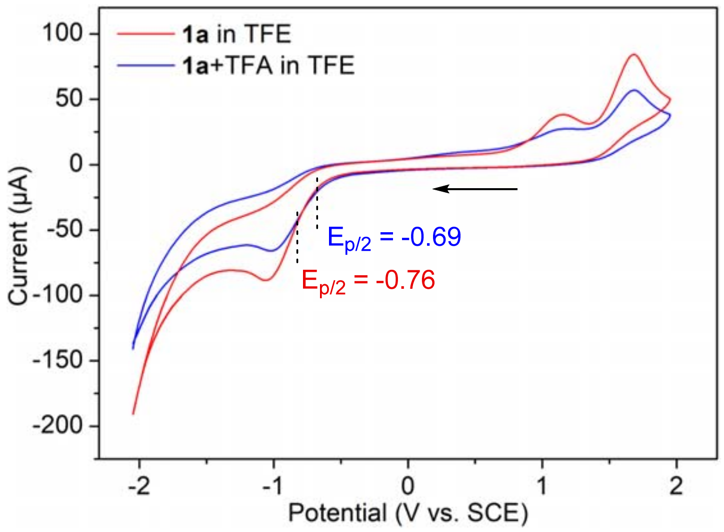

Supplementary Figure 8. Cyclic voltammograms of 1a (1 mM) and 1a+TFA (1:1 molar ratio, 1 mM) in TFE solution

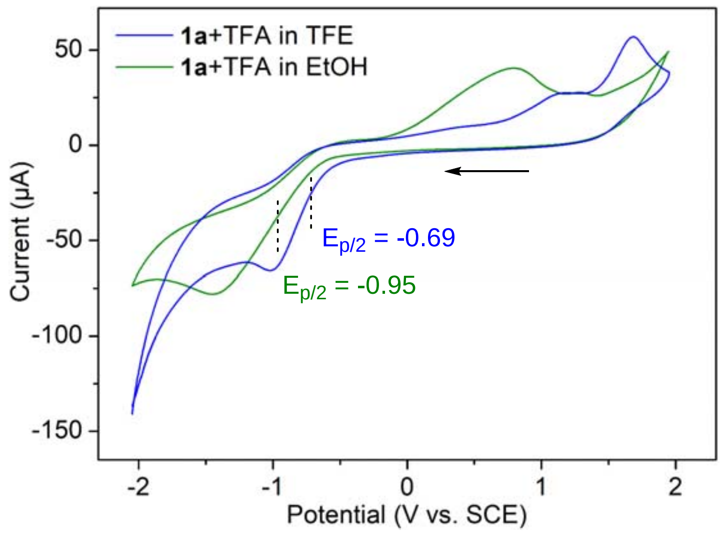

Supplementary Figure 9. Cyclic voltammograms of 1a+TFA (1:1 molar ratio, 1 mM) in TFE solution and EtOH solution

2.8.4 Light on/off experiments

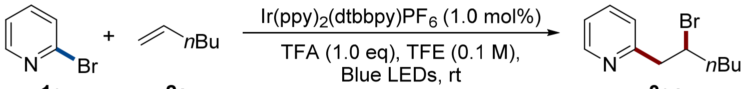

To an oven-dried 4 mL vial was added Ir(ppy)$_2$(dtbbpy)PF$_6$ (0.002 mmol, 1.0 mol%), halopyridines **1g** (0.2 mmol, 1.0 equiv.), alkenes **2a** (0.6 mmol, 3.0 equiv.), TFA (0.2 mmol, 1.0 equiv.), dimethyl terephthalate as internal standard (0.1 mmol) and TFE (2.0 mL, 0.1 M) in the nitrogen glove box. The vial was capped with a septum and wrapped with parafilm. The reaction was sequentially stirred under visible light irradiation (1 x Kessil PR160, λ_{max}= 456 nm, 40 W, irradiation temperature maintained between 25-30 ℃) and under the dark atmosphere. Every one hours an aliquot of 10 µL was removed *via* syringe and analyzed by GC-FID. After a total of 8 h the determined yields were plotted against the reaction time.

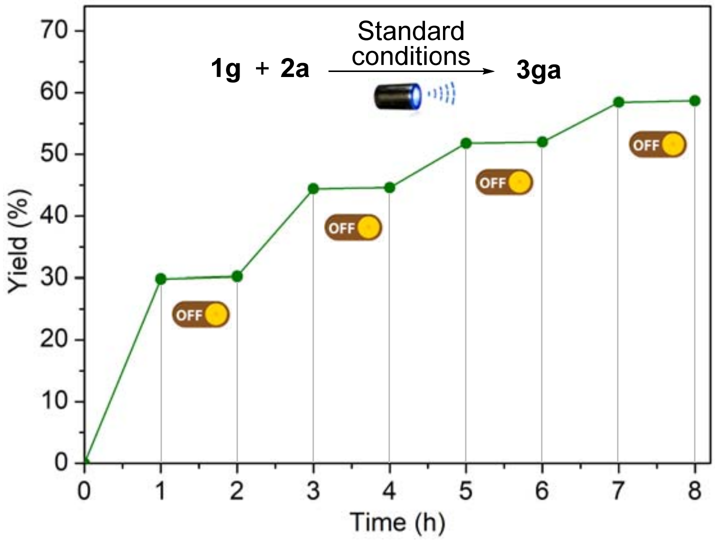

Supplementary Figure 10. Effect of Blue LEDs irradiation

2.8.5 Quantum yield measurements (Blue LEDs)

(a) Determination of the photon flux of Kessil Blue LEDs (456 nm):

Kessil LEDs (λ_{max} = 456 nm) was used with 25% intensity for measurement of quantum yield.

The photon flux of the LED (λ_{max} = 456 nm) was determined by standard ferrioxalate actinometry.[1] A 0.15 M solution of ferrioxalate was prepared by dissolving potassium ferrioxalate hydrate (0.737 g) in H_2SO_4 (10 mL of a 0.05 M solution). A buffered solution of 1,10-phenanthroline was prepared by dissolving 1,10-phenanthroline (5.0 mg) and sodium acetate (1.13 g) in H_2SO_4 (5.0 mL of a 0.5 M solution). Both solutions were stored in the dark. To determine the photon flux of Kessil LEDs, the ferrioxalate solution (2.0 mL) was placed in a cuvette and irradiated for 90 seconds at λ_{max} = 456 nm. After irradiation, the phenanthroline solution (0.35 mL) was added to the cuvette and the mixture was allowed to stir in the dark for 1 h to allow the ferrous ions to completely coordinate to the phenanthroline. The absorbance of the solution was measured at 510 nm. Three groups of parallel experiments were carried out to calculate the average value. A non-irradiated sample was also prepared and the absorbance at 510 nm was measured. And the results were summarized in Supplementary Table 10. Conversion was calculated using eq 1.

Supplementary Table 10. Absorbance of ferrioxalate samples at $A_{510\ nm}$

Irradiation-1	Irradiation-2	Irradiation-3	Non-Irradiation	$\Delta A_{510\ nm}$
2.5201	2.4832	2.5304	0.3388	2.1724
	Average value = 2.5112			

$$mol\,of\,Fe^{2+} = \frac{V \cdot \Delta A_{510nm}}{l \cdot \varepsilon} = \frac{(0.00235\,L) \cdot 2.1724}{(1.0\,cm) \cdot (11{,}100\,\frac{L}{mol \cdot \mathrm{cm}})} = 4.60 \times 10^{-7} \quad (1)$$

"V" is the total volume (0.00235 L) of the solution after addition of phenanthroline, "ΔA" is the difference in absorbance at 510 nm between the irradiated and non-irradiated solutions, "l" is the path length (1.00 cm), and "ε" is the molar absorptivity of the ferrioxalate actinometer at 510 nm (11,100 $L \cdot mol^{-1}cm^{-1}$).[2] The photon flux can be calculated using eq 2 and 3.

$$f = 1 - 10^{-A_{456nm}} = 1 - 10^{-1.2319} = 0.9414 \quad (2)$$

$$Photo\ flux_{Kessil\,456nm} = \frac{mol\,of\,Fe^{+2}}{\phi \cdot t \cdot f} = \frac{4.6 \times 10^{-7}}{(0.84) \cdot (90s) \cdot (0.9414)} = 6.46 \times 10^{-9}\ einstein/s \quad (3)$$

"Φ" is the quantum yield for the ferrioxalate actinometer (0.84 at λ = 456 nm),[3] "t" is the irradiation time (90 s), and "f" is the fraction of light absorbed at 456 nm by the ferrioxalate actinometer, which is calculated using eq 2 where $A_{456\ nm}$ is the absorbance of the ferrioxalate solution at 456 nm. An absorption spectrum gave an $A_{456\ nm}$ value of 1.2319 (Supplementary Figure 11, black curve), indicating that the fraction of absorbed light (f) is 0.9414. The photon flux was thus calculated (average of three experiments) to be 6.46 × 10^{-9} einsteins·s^{-1}.

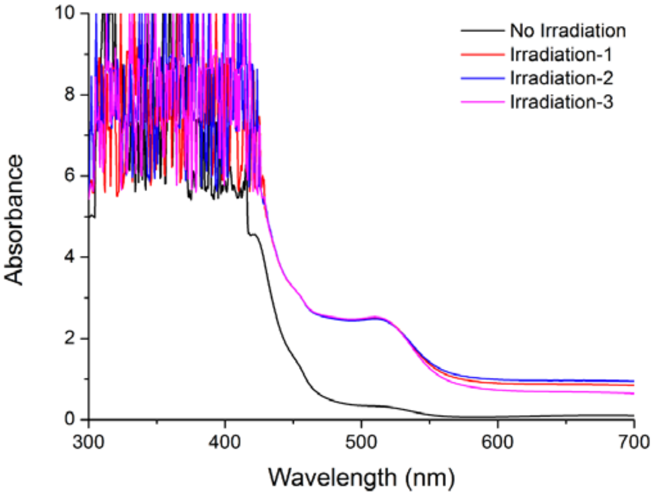

Supplementary Figure 11. Absorption spectra of irradiation and non-irradiation experiments

(b) Determination of the reaction quantum yield:

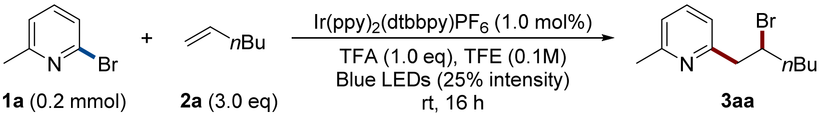

The reaction mixture was stirred and irradiated by Kessil LEDs (λ = 456 nm, 25% intensity) for 3600 s. The yield of product **3aa** was determined by GC-FID analysis of the crude reaction mixture using mesitylene as internal standard. The yield of **3aa** was determined to be 7.5% (0.15 × 10^{-3} mol). The reaction quantum yield "Φ" was determined using eq 5 where the photon flux is 6.46 × 10^{-9} einsteins·s^{-1} (determined by actinometry as described above), "t" is the reaction time (3600 s) and "f" is the fraction of incident light absorbed by the catalyst, determined using eq 4. An absorption spectrum of the catalyst $Ir(ppy)_2(dtbbpy)PF_6$ (0.0025 M in TFE) gave an absorbance value of 1.2382 at 456 nm (Supplementary Figure 12), indicating that the fraction of light absorbed by the photocatalyst (f) is 0.9422. The reaction quantum yield (Φ) was calculated to be 0.6846.

$$f = 1 - 10^{-A_{456nm}} = 1 - 10^{-1.2382} = 0.9422 \quad (4)$$

$$\Phi = \frac{mol\ of\ 3aa}{photo\ flux_{Kessil\,456nm} \cdot t \cdot f} = \frac{0.015 \times 10^{-3}\, mol}{6.46 \times 10^{-9}\, einstein \cdot s^{-1} \cdot 3600\, s \cdot 0.9422} = 0.6846 \quad (5)$$

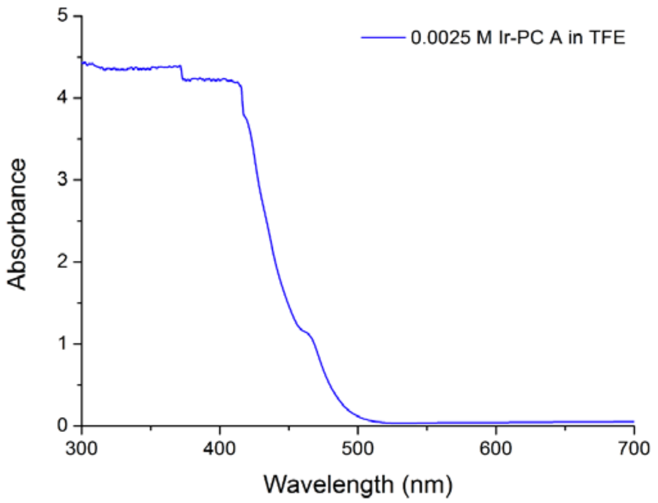

Supplementary Figure 12. Absorption spectra of 0.0025 M solution of $Ir(ppy)_2(dtbbpy)PF_6$ in TFE

2.8.6 Halide anions competition experiments

(a) Kinetic studies of halide anions competition

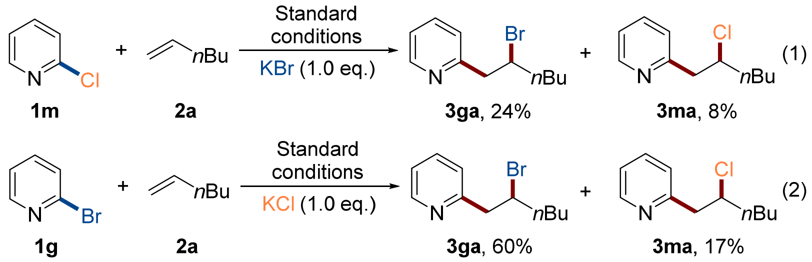

Reaction (1): To an oven-dried 4 mL vial was added $Ir(ppy)_2(dtbbpy)PF_6$ (0.002 mmol, 1.0 mol%), halopyridines **1m** (0.2 mmol, 1.0 equiv.), alkenes **2a** (0.6 mmol, 3.0 equiv.), TFA (0.2 mmol, 1.0 equiv.), KBr (0.2 mmol, 1.0 equiv.), dimethyl terephthalate as internal standard (0.1 mmol) and TFE (2.0 mL, 0.1 M) in the nitrogen glove box. The vial was capped with a septum and wrapped with parafilm. The reaction mixture was stirred under visible light irradiation. Every half an hour (0 h - 2 h) and every two hours (2 h - 14 h) an aliquot of 10 μL was removed *via* syringe and analyzed by GC-FID. After a total of 14 h the determined yields were plotted against the reaction time.

Reaction (2): To an oven-dried 4 mL vial was added $Ir(ppy)_2(dtbbpy)PF_6$ (0.002 mmol, 1.0 mol%), halopyridines **1g** (0.2 mmol, 1.0 equiv.), alkenes **2a** (0.6 mmol, 3.0 equiv.), TFA (0.2 mmol, 1.0 equiv.), KCl (0.2 mmol, 1.0 equiv.), dimethyl terephthalate as internal standard (0.1 mmol) and TFE (2.0 mL, 0.1 M) in the nitrogen glove box. The vial was capped with a septum and wrapped with parafilm. The reaction mixture was stirred under visible light irradiation. Every half an hour (0 h - 2 h) and every two hours (2 h - 14 h) an aliquot of 10 μL was removed *via* syringe and analyzed by GC-FID. After a total of 14 h the determined yields were plotted against the reaction time.

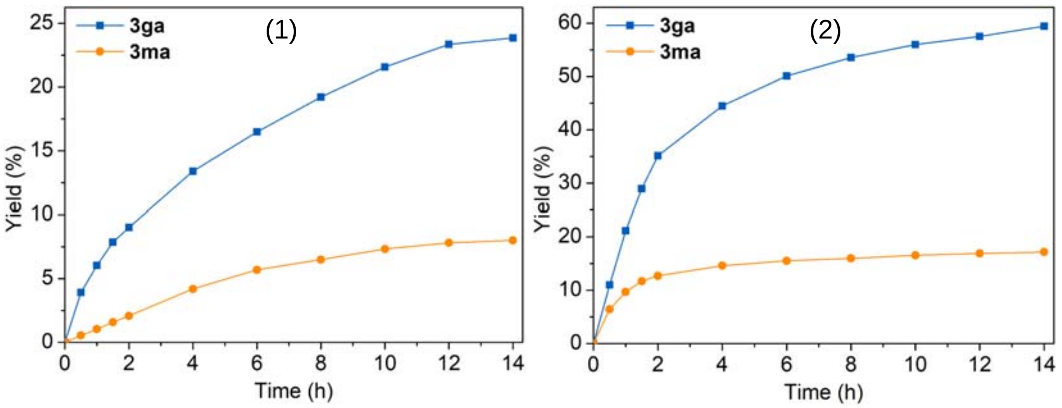

Supplementary Figure 13. Kinetic studies

(b) Crossover experiments

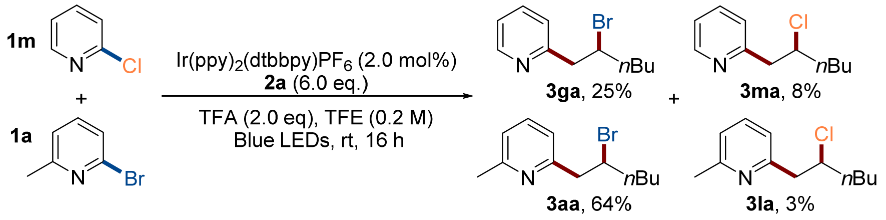

To an oven-dried 4 mL vial was added Ir(ppy)$_2$(dtbbpy)PF$_6$ (0.004 mmol, 2.0 mol%), halopyridines **1m** (0.2 mmol, 1.0 equiv.) and **1a** (0.2 mmol, 1.0 equiv.), alkenes **2a** (1.2 mmol, 6.0 equiv.), TFA (0.4 mmol, 2.0 equiv.) and TFE (2.0 mL, 0.1 M) in the nitrogen glove box. The vial was capped with a septum and wrapped with parafilm. The reaction mixture was stirred under visible light irradiation for 16 h and the yields determined by GC-FID analysis with dimethyl terephthalate as internal standard.

(c) Interchange experiments

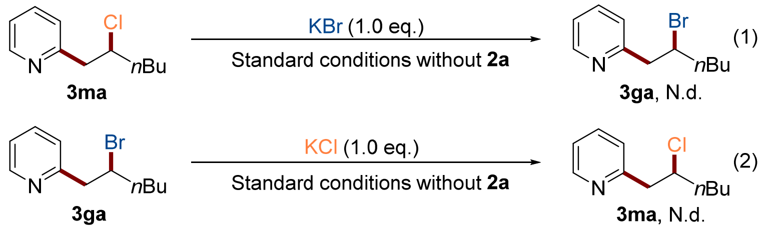

Reaction (1): To an oven-dried 4 mL vial was added Ir(ppy)$_2$(dtbbpy)PF$_6$ (0.002 mmol, 1.0 mol%), **3ma** (0.2 mmol, 1.0 equiv.), TFA (0.2 mmol, 1.0 equiv.) and TFE (2.0 mL, 0.1 M) in the nitrogen glove box. The vial was capped with a septum and wrapped with parafilm. The reaction mixture was stirred under visible light irradiation for 16 h and the yields determined by GC-FID analysis with dimethyl terephthalate as internal standard.

Reaction (2): To an oven-dried 4 mL vial was added Ir(ppy)$_2$(dtbbpy)PF$_6$ (0.002 mmol, 1.0 mol%), **3ga** (0.2 mmol, 1.0 equiv.), TFA (0.2 mmol, 1.0 equiv.) and TFE (2.0 mL, 0.1 M) in the nitrogen glove box. The vial was capped with a septum and wrapped with parafilm. The reaction mixture was stirred under visible light irradiation for 16 h and the yields determined by GC-FID analysis with dimethyl terephthalate as internal standard.

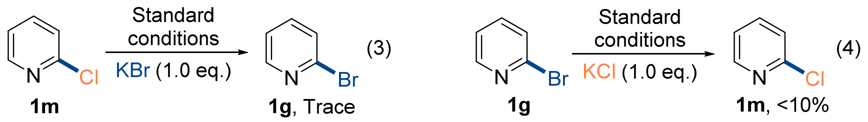

Reaction (3): To an oven-dried 4 mL vial was added Ir(ppy)$_2$(dtbbpy)PF$_6$ (0.002 mmol, 1.0 mol%), **1m** (0.2 mmol, 1.0 equiv.), TFA (0.2 mmol, 1.0 equiv.) KBr (0.2 mmol, 1.0 equiv.) and TFE (2.0 mL, 0.1 M) in the nitrogen glove box. The vial was capped with a septum and wrapped with parafilm. The reaction mixture was stirred under visible light irradiation for 16 h and the yields determined by GC-FID analysis with dimethyl terephthalate as internal standard.

Reaction (4): To an oven-dried 4 mL vial was added Ir(ppy)$_2$(dtbbpy)PF$_6$ (0.002 mmol, 1.0 mol%), **1g** (0.2 mmol, 1.0 equiv.), TFA (0.2 mmol, 1.0 equiv.) KCl (0.2 mmol, 1.0 equiv.) and TFE (2.0 mL, 0.1 M) in the nitrogen glove box. The vial was capped with a septum and wrapped with parafilm. The reaction mixture was stirred under visible light irradiation for 16 h and the yields determined by GC-FID analysis with dimethyl terephthalate as internal standard.

2.9. Copies of NMR spectra of products

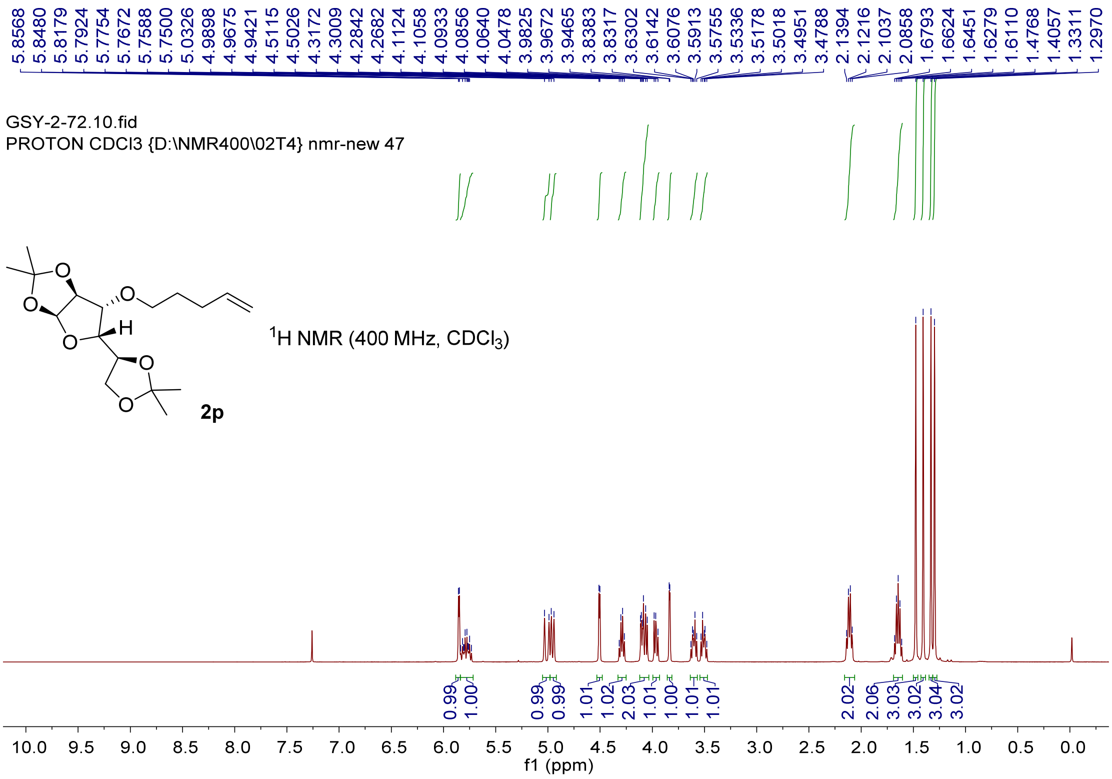

Supplementary Figure 14. ^{1}H NMR spectra of compound 2p

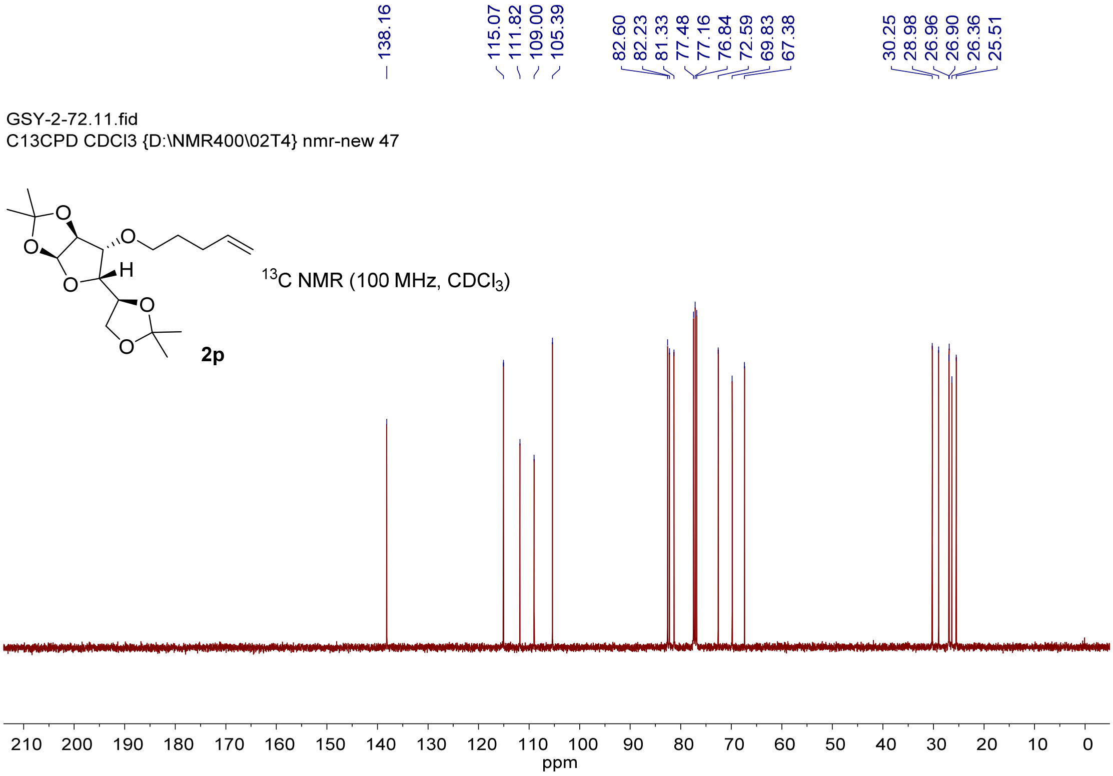

Supplementary Figure 15. ^{13}C NMR spectra of compound 2p

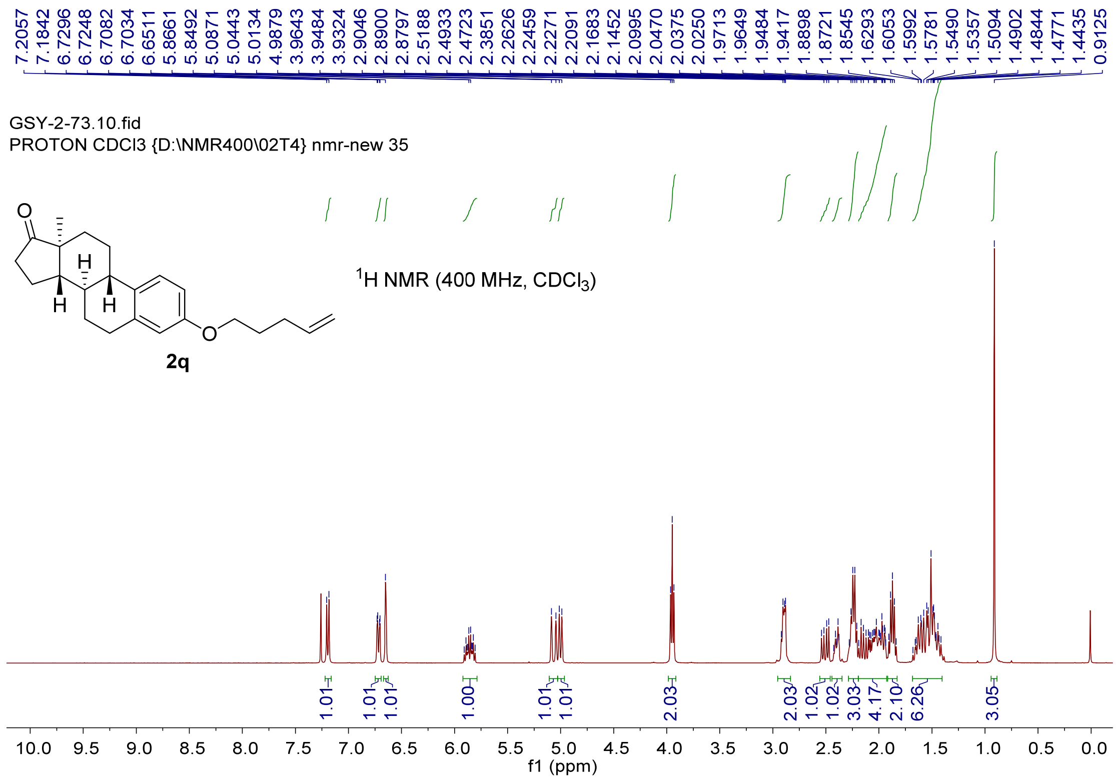

Supplementary Figure 16. ^{1}H NMR spectra of compound 2q

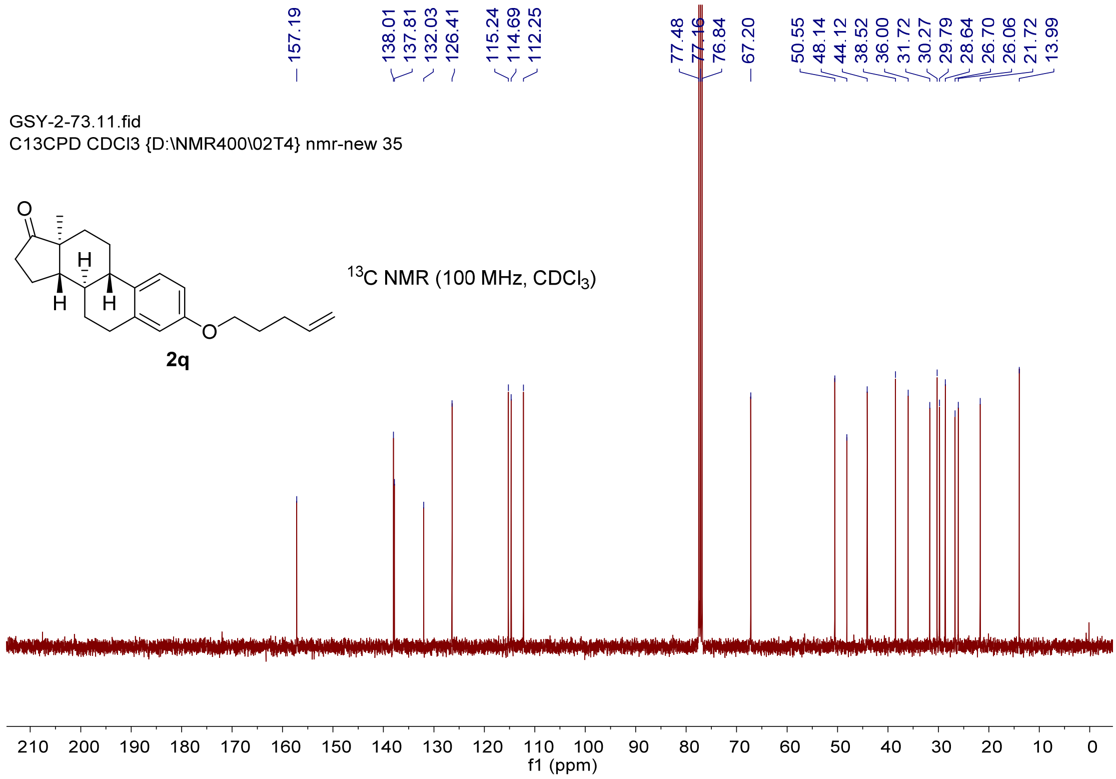

Supplementary Figure 17. ^{13}C NMR spectra of compound 2p

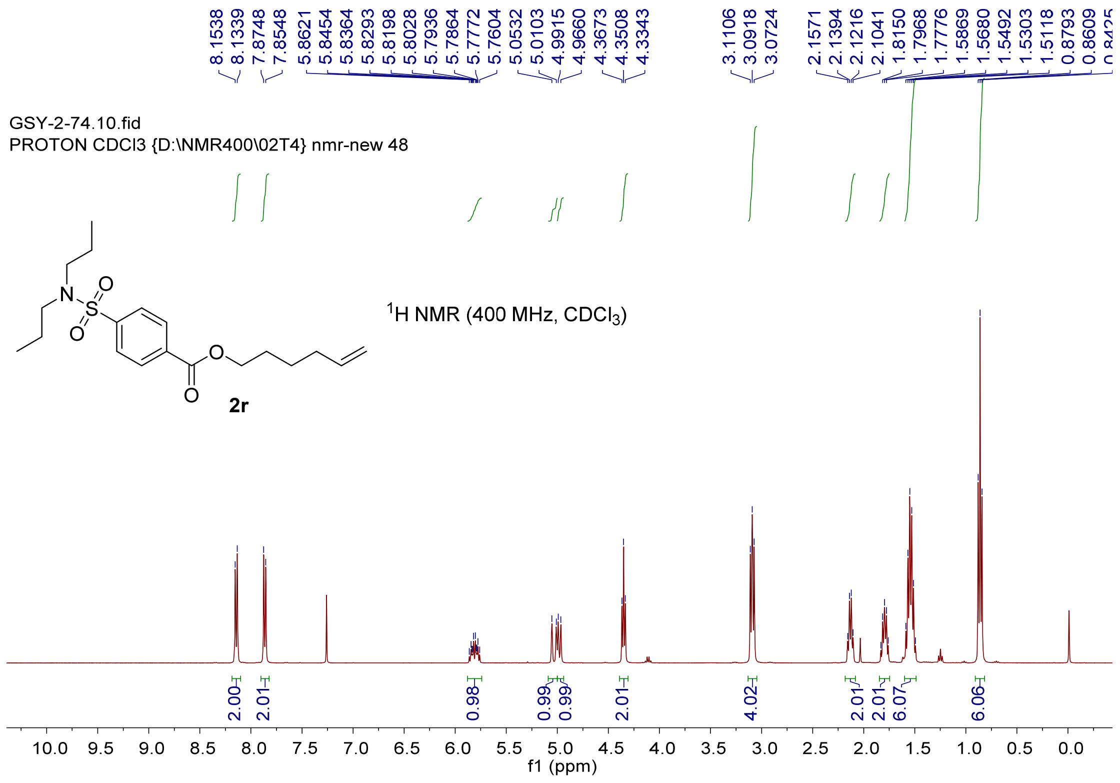

Supplementary Figure 18. ^{1}H NMR spectra of compound 2r

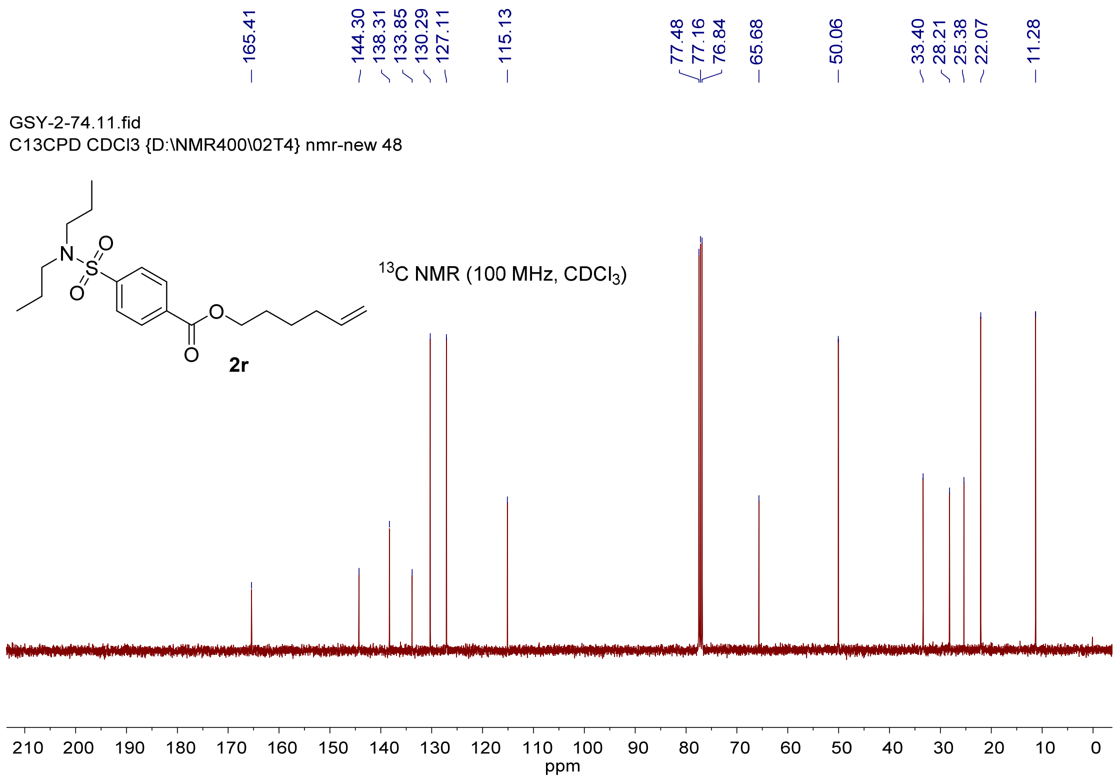

Supplementary Figure 19. ^{13}C NMR spectra of compound 2r

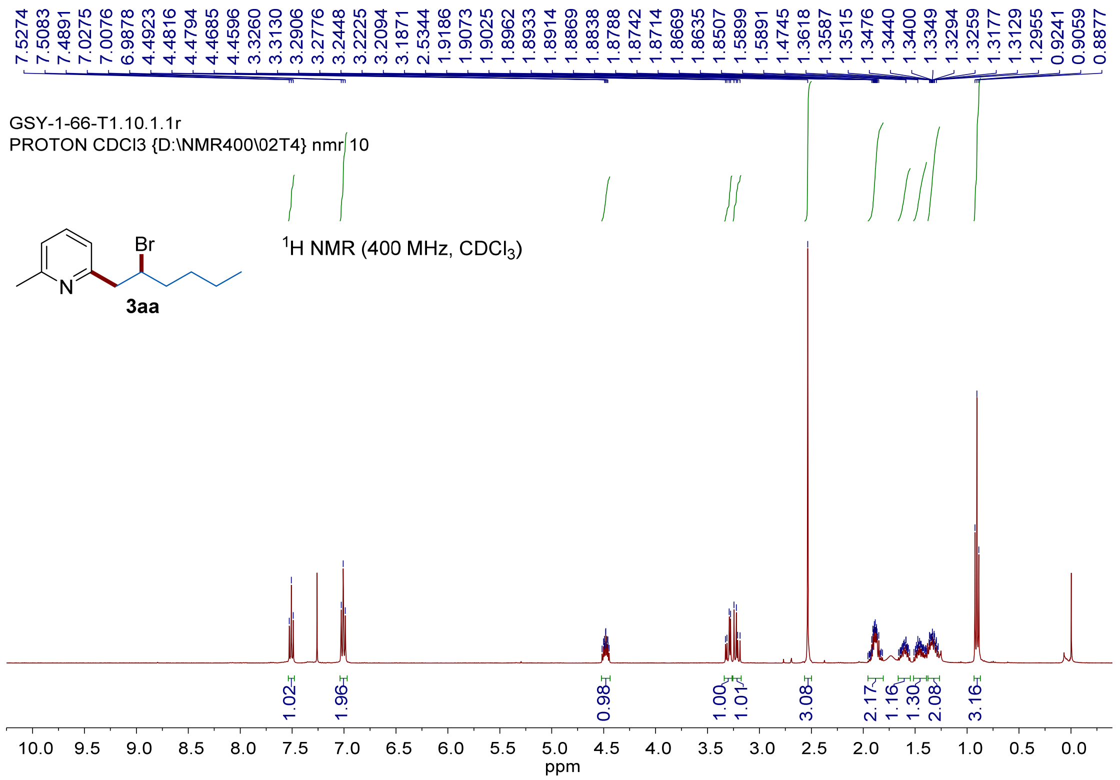

Supplementary Figure 20. ^{1}H NMR spectra of compound 3aa

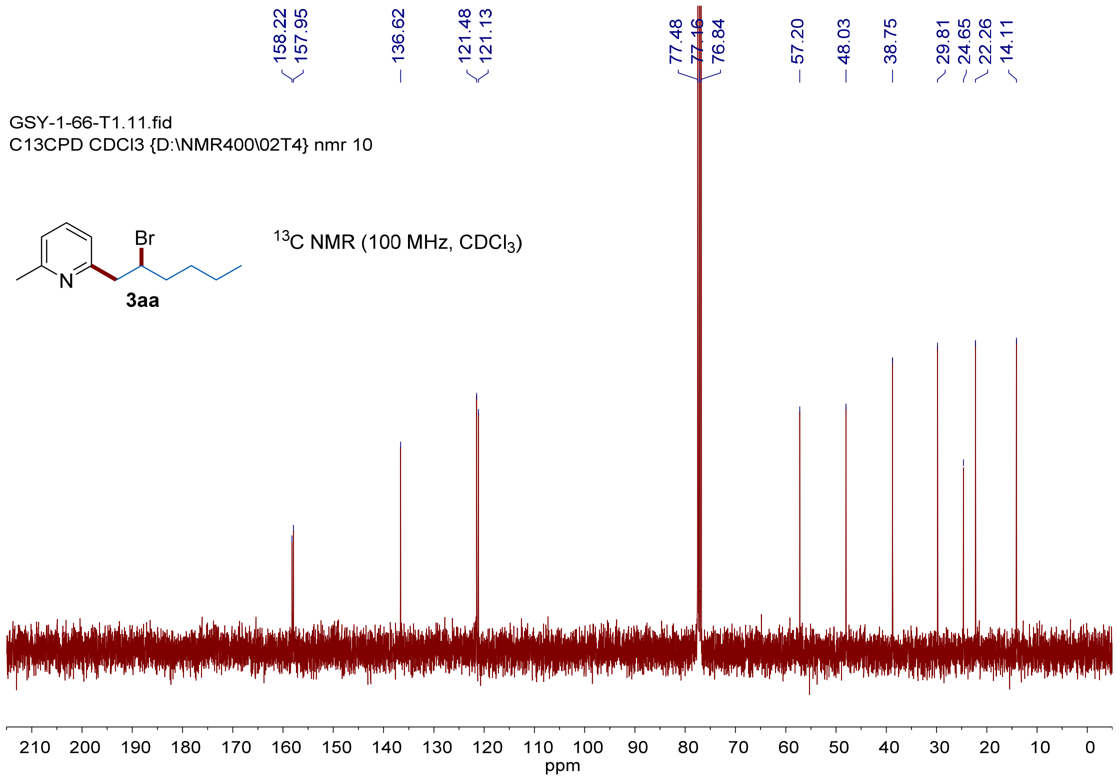

Supplementary Figure 21. ^{13}C NMR spectra of compound 3aa

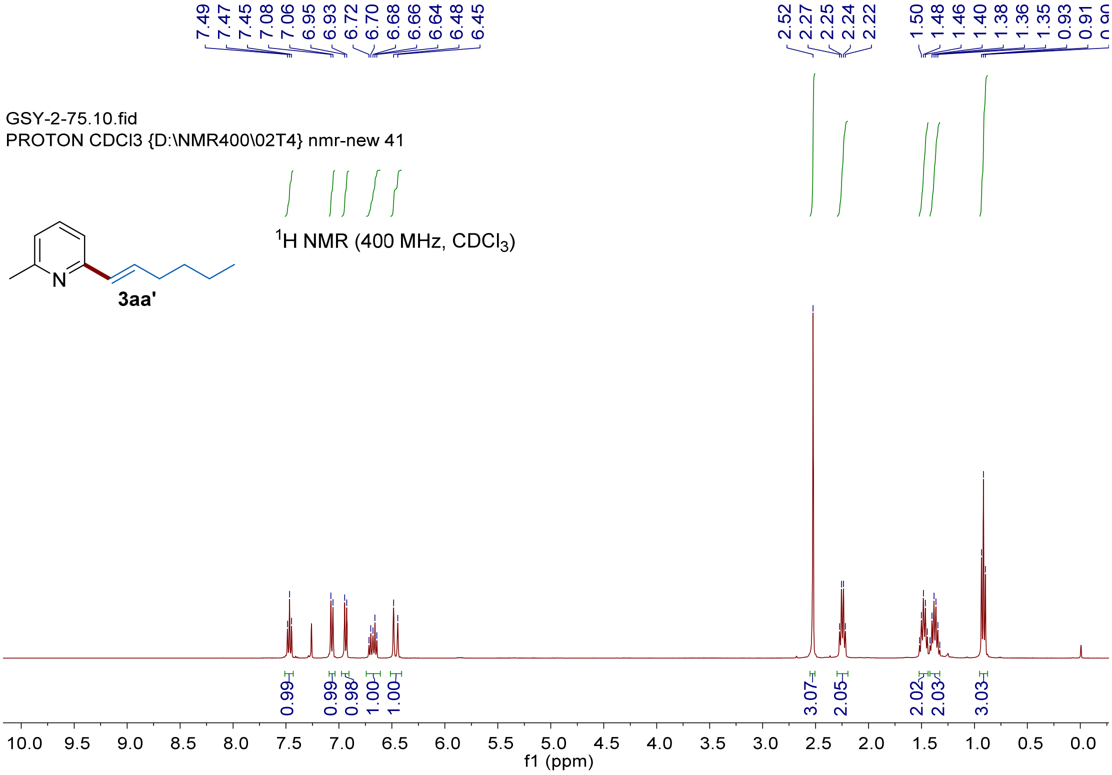

Supplementary Figure 22. ^{1}H NMR spectra of compound 3aa'

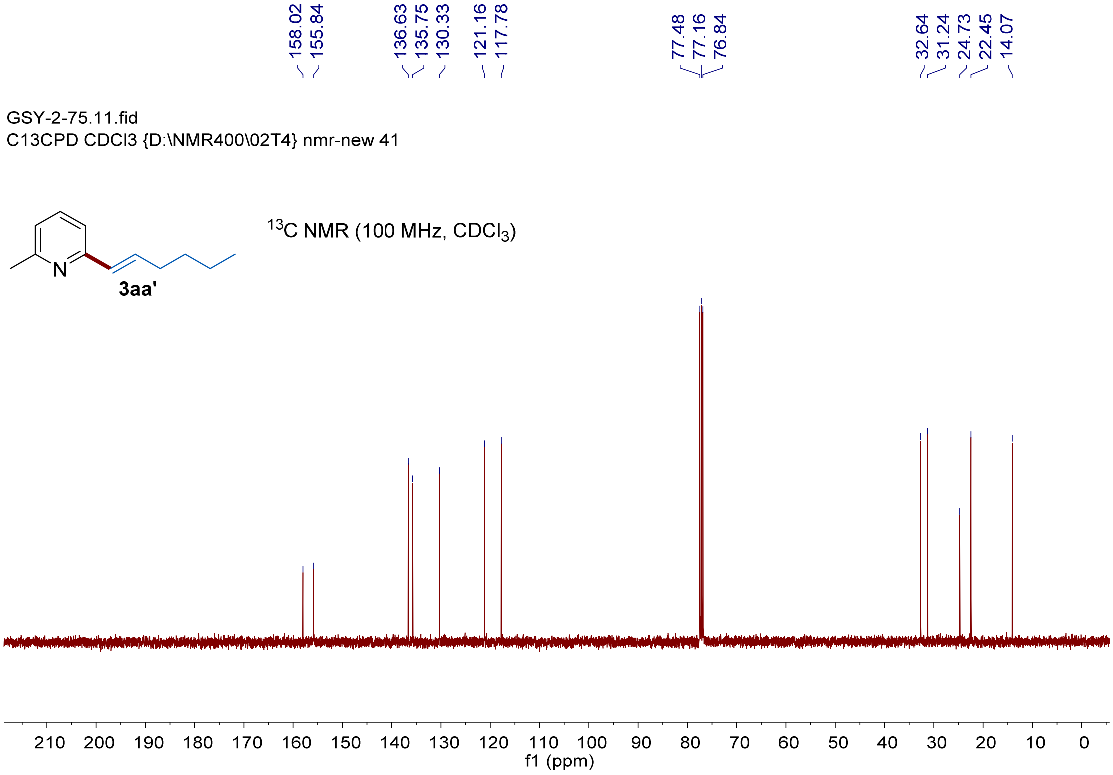

Supplementary Figure 23. ^{13}C NMR spectra of compound 3aa'

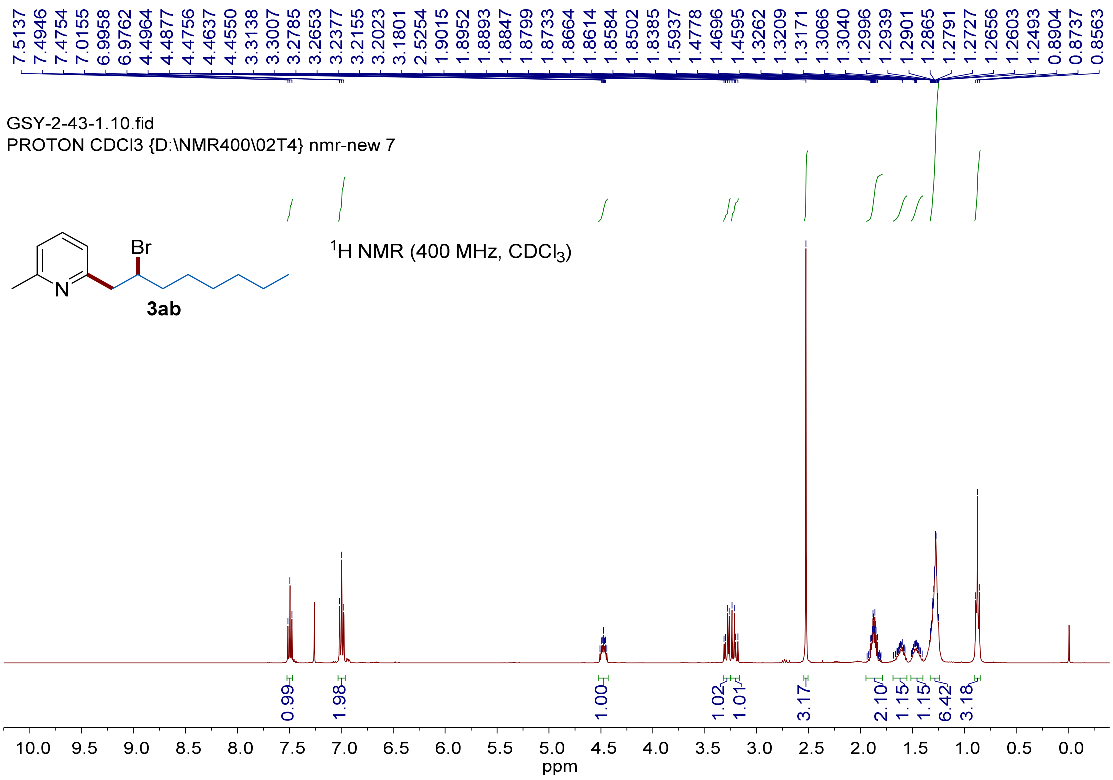

Supplementary Figure 24. ^{1}H NMR spectra of compound 3ab

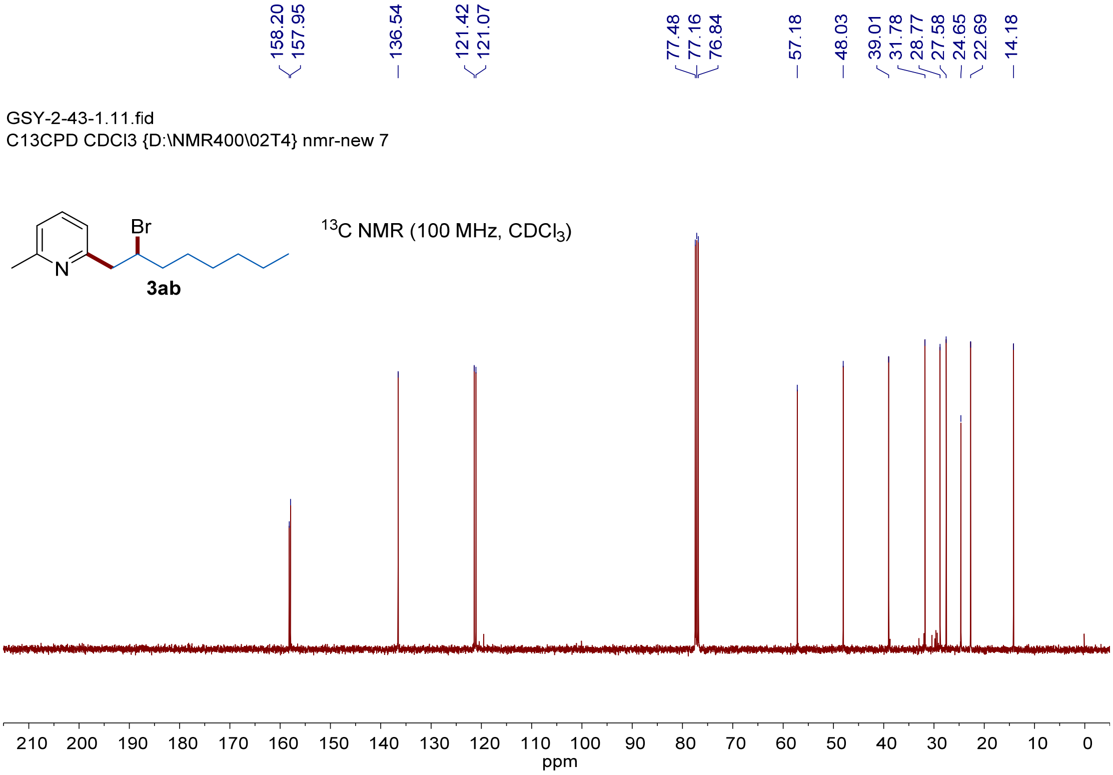

Supplementary Figure 25. ^{13}C NMR spectra of compound 3ab

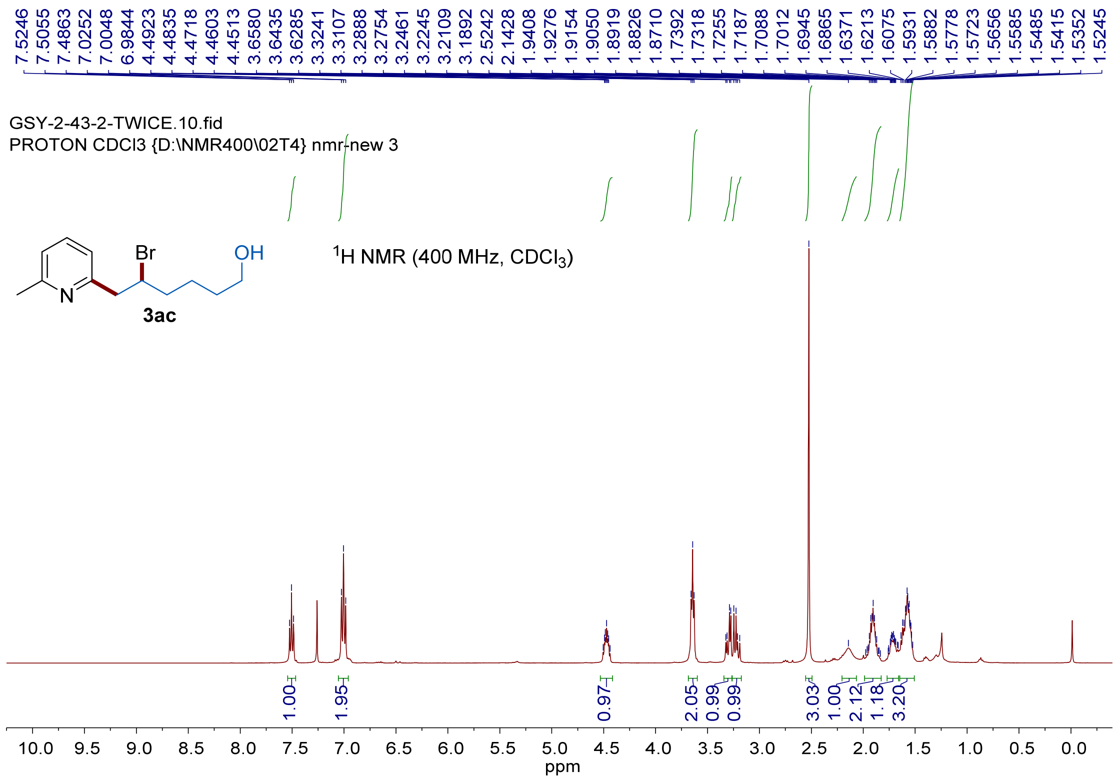

Supplementary Figure 26. ^{1}H NMR spectra of compound 3ac

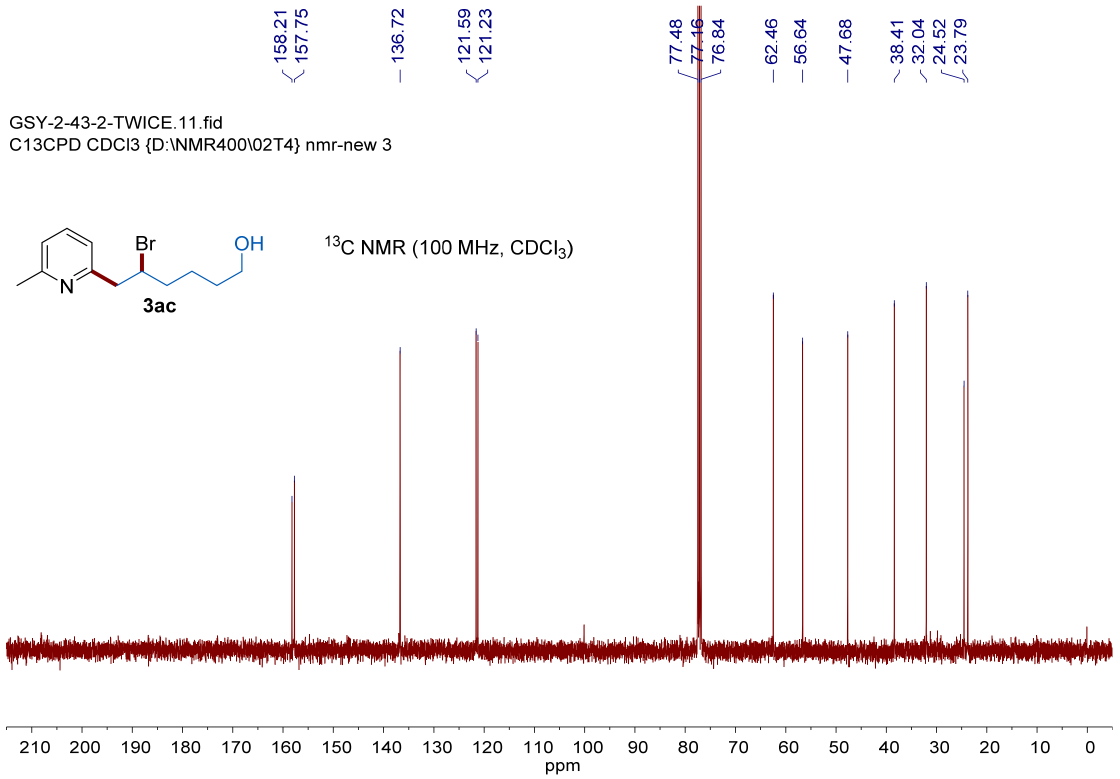

Supplementary Figure 27. ^{13}C NMR spectra of compound 3ac

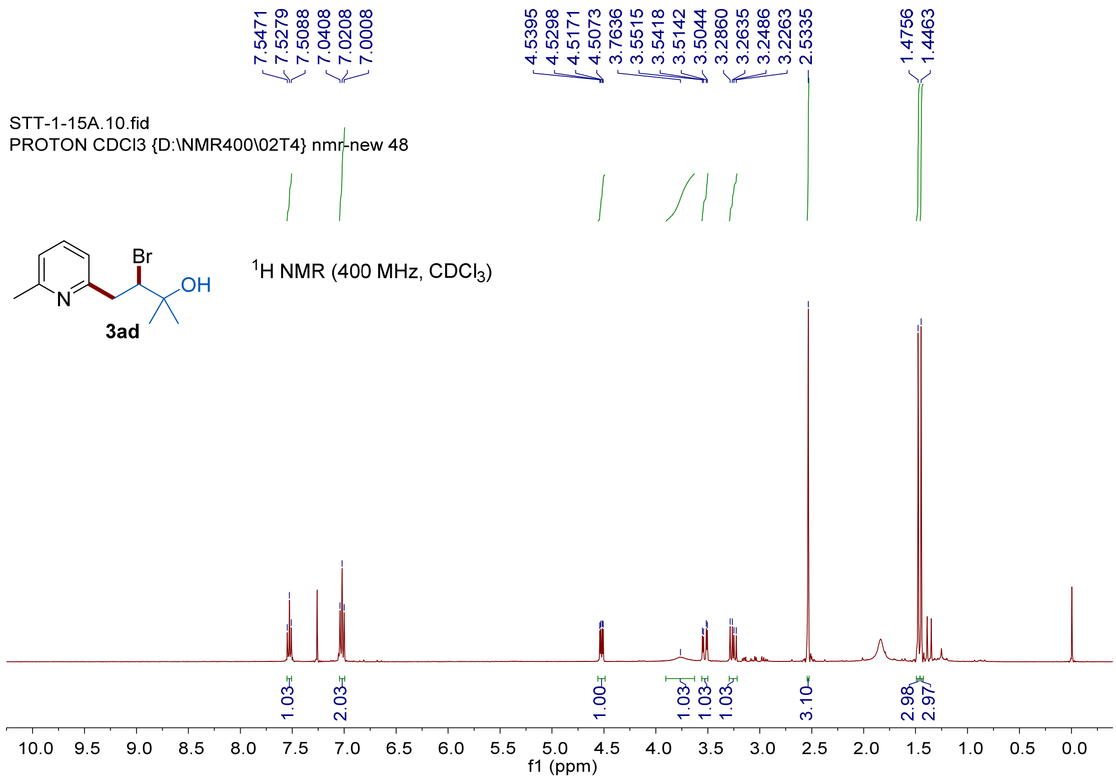

Supplementary Figure 28. ^{1}H NMR spectra of compound 3ad

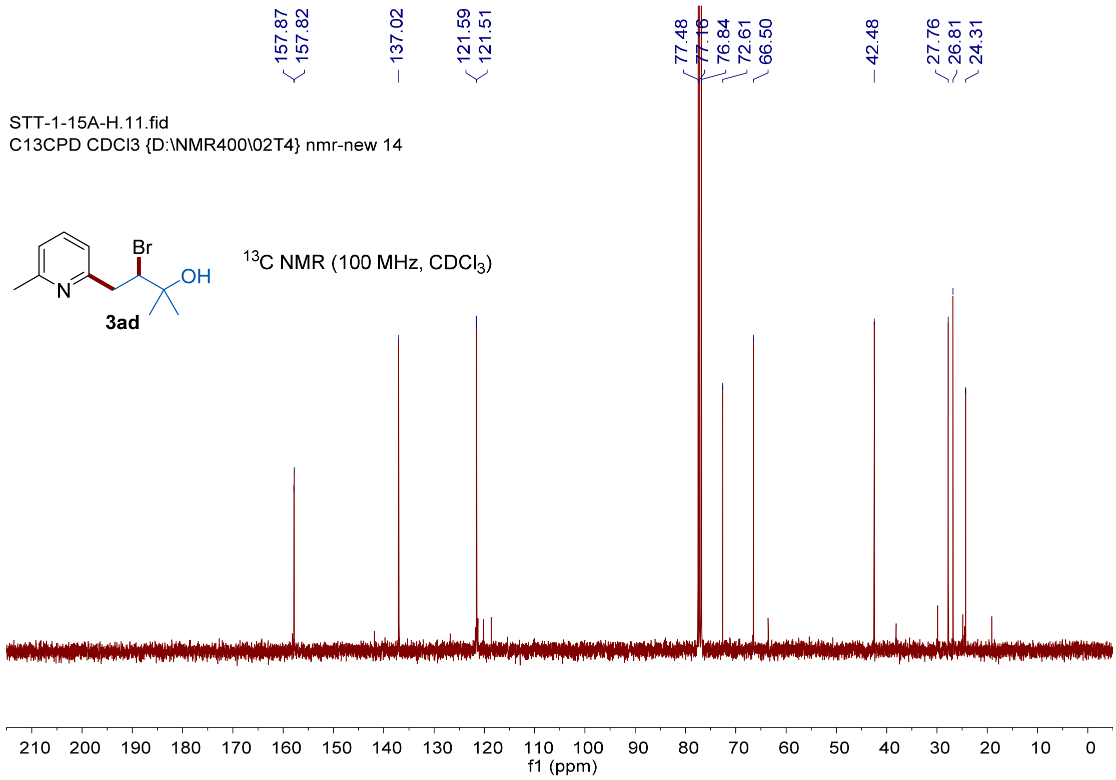

Supplementary Figure 29. ^{13}C NMR spectra of compound 3ad

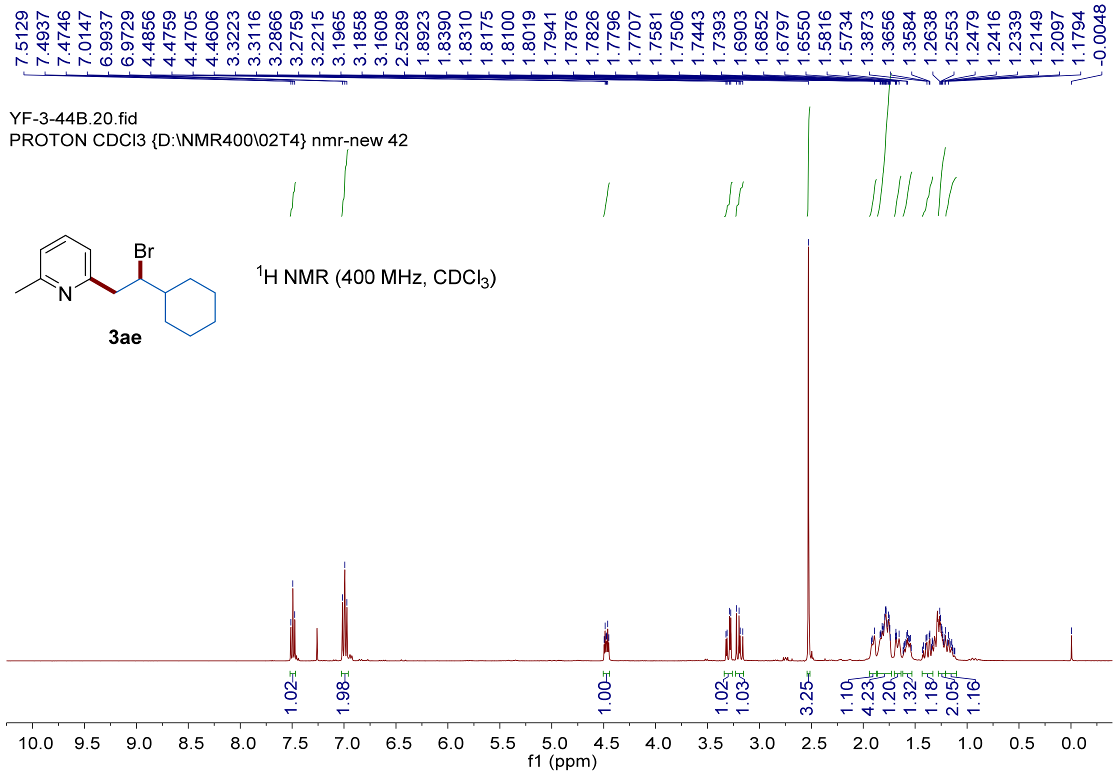

Supplementary Figure 30. ^{1}H NMR spectra of compound 3ae

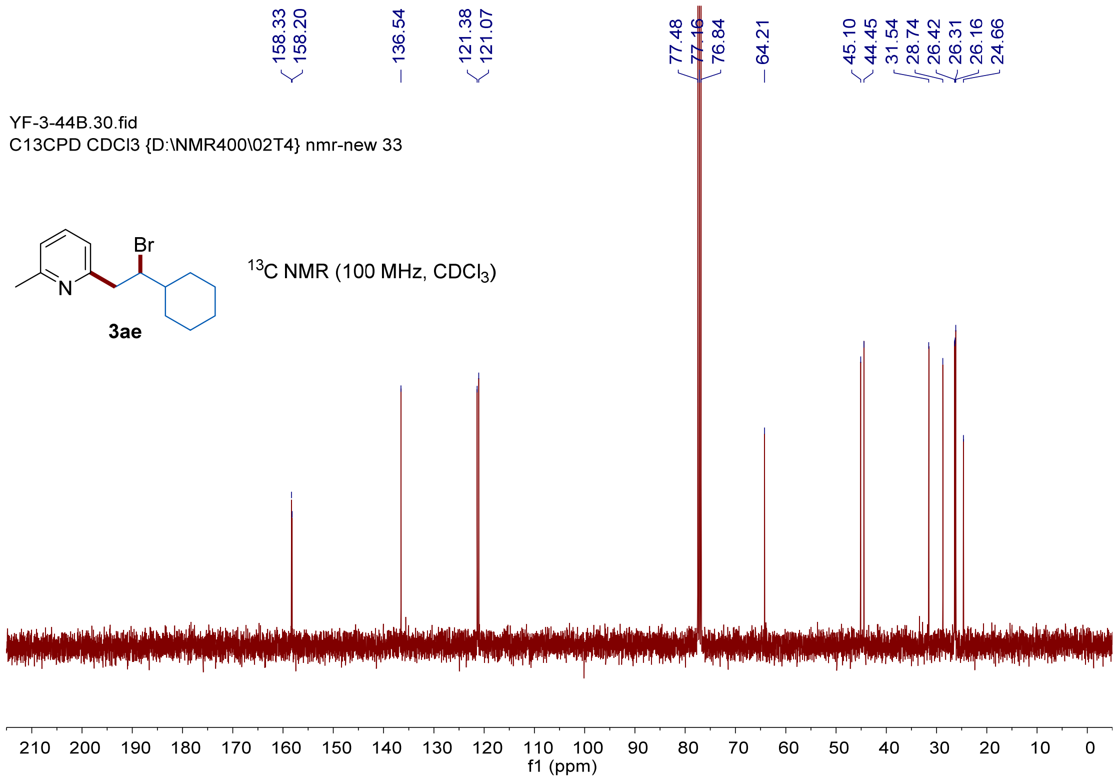

Supplementary Figure 31. ^{13}C NMR spectra of compound 3ae

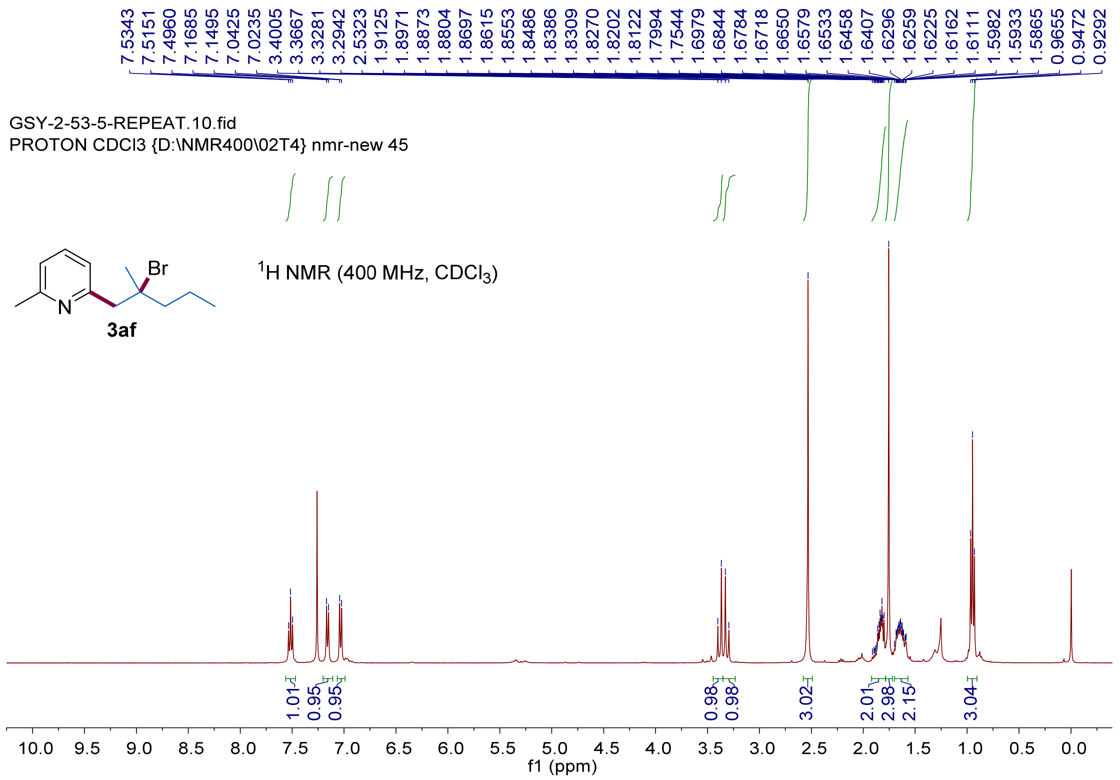

Supplementary Figure 32. ^{1}H NMR spectra of compound 3af

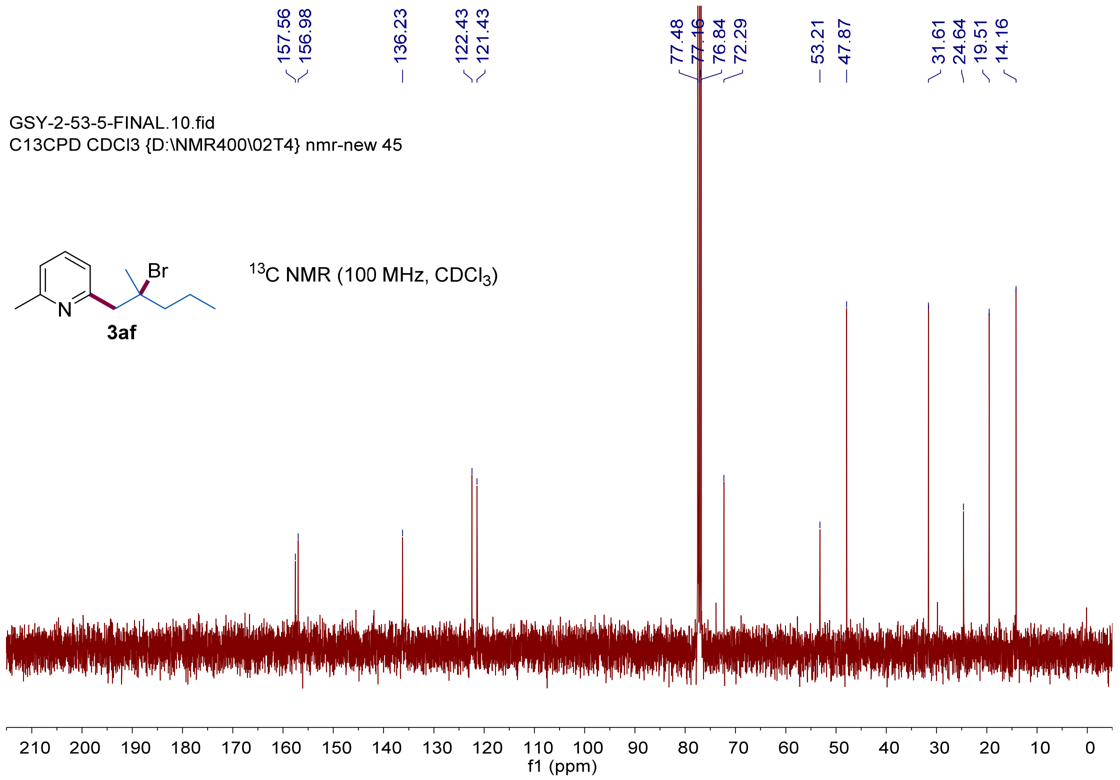

Supplementary Figure 33. ^{13}C NMR spectra of compound 3af

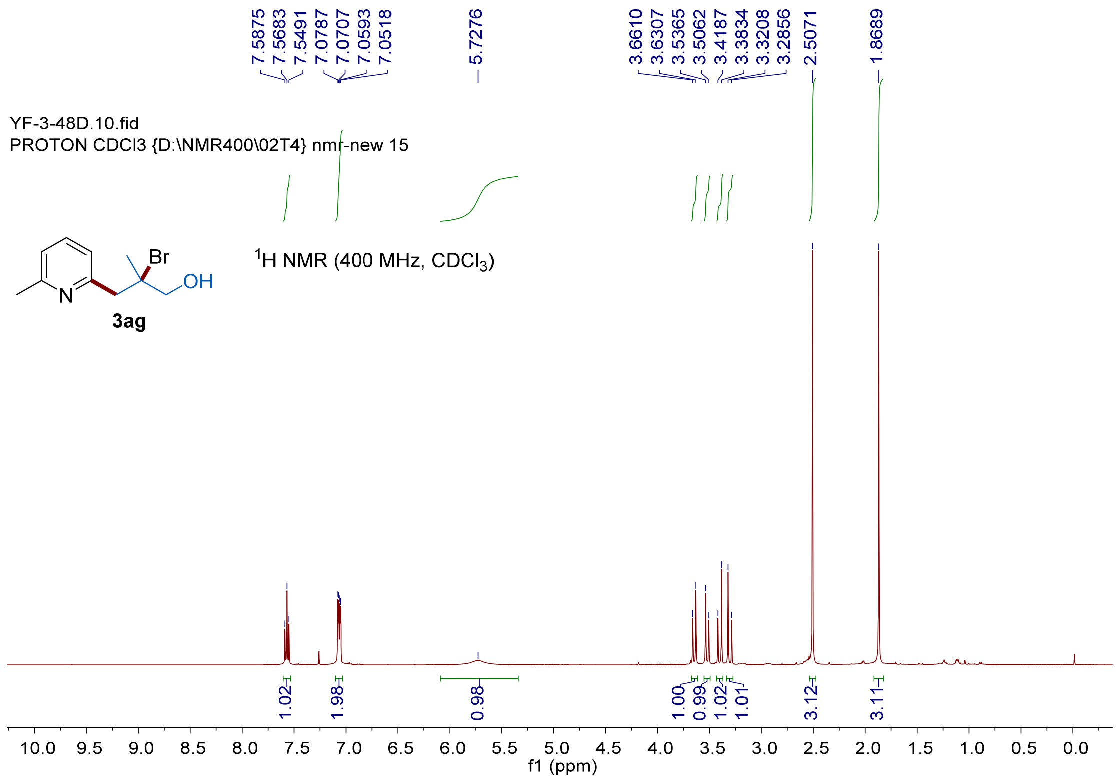

Supplementary Figure 34. ^{1}H NMR spectra of compound 3ag

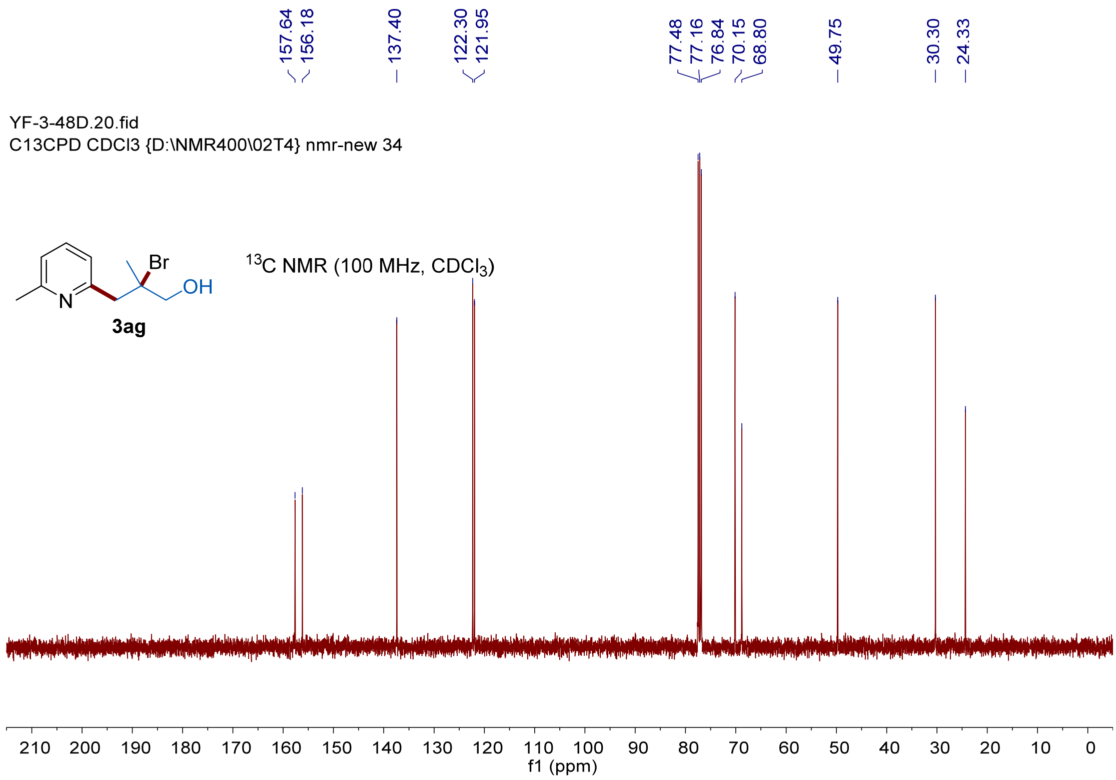

Supplementary Figure 35. ^{13}C NMR spectra of compound 3ag

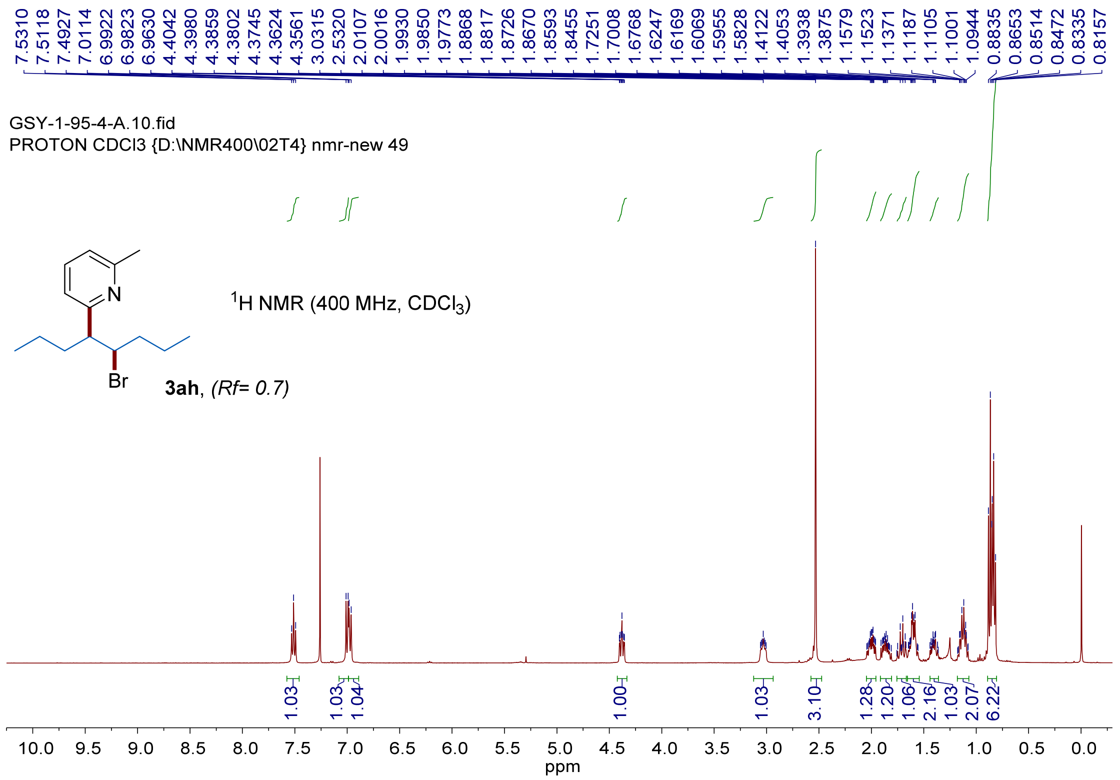

Supplementary Figure 36. ^{1}H NMR spectra of compound 3ah (*Rf* = *0.7*)

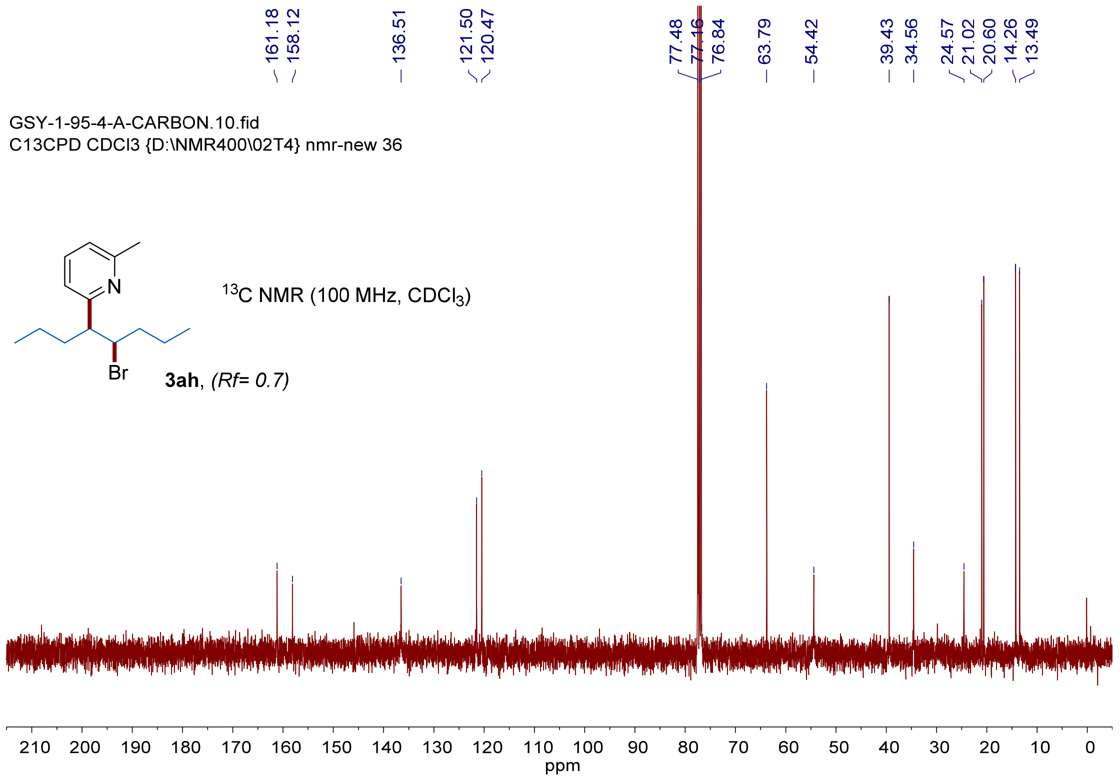

Supplementary Figure 37. ^{13}C NMR spectra of compound 3ah (*Rf* = *0.7*)

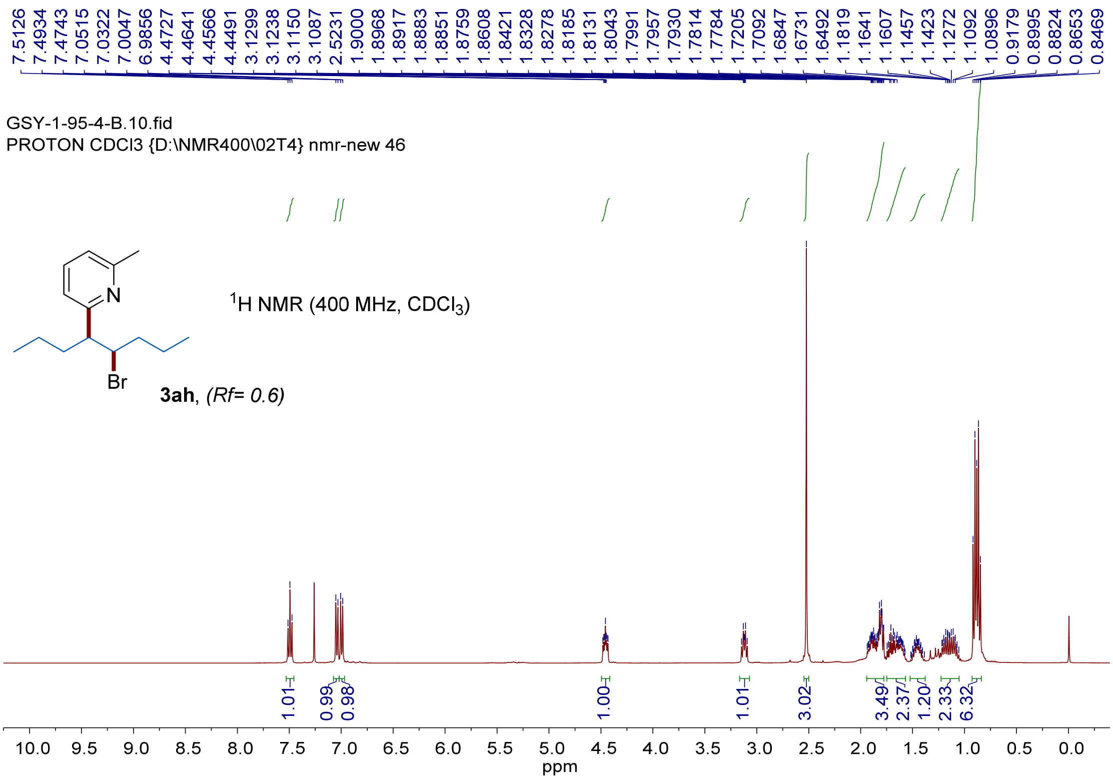

Supplementary Figure 38. ^{1}H NMR spectra of compound 3ah (***Rf* = *0.6***)

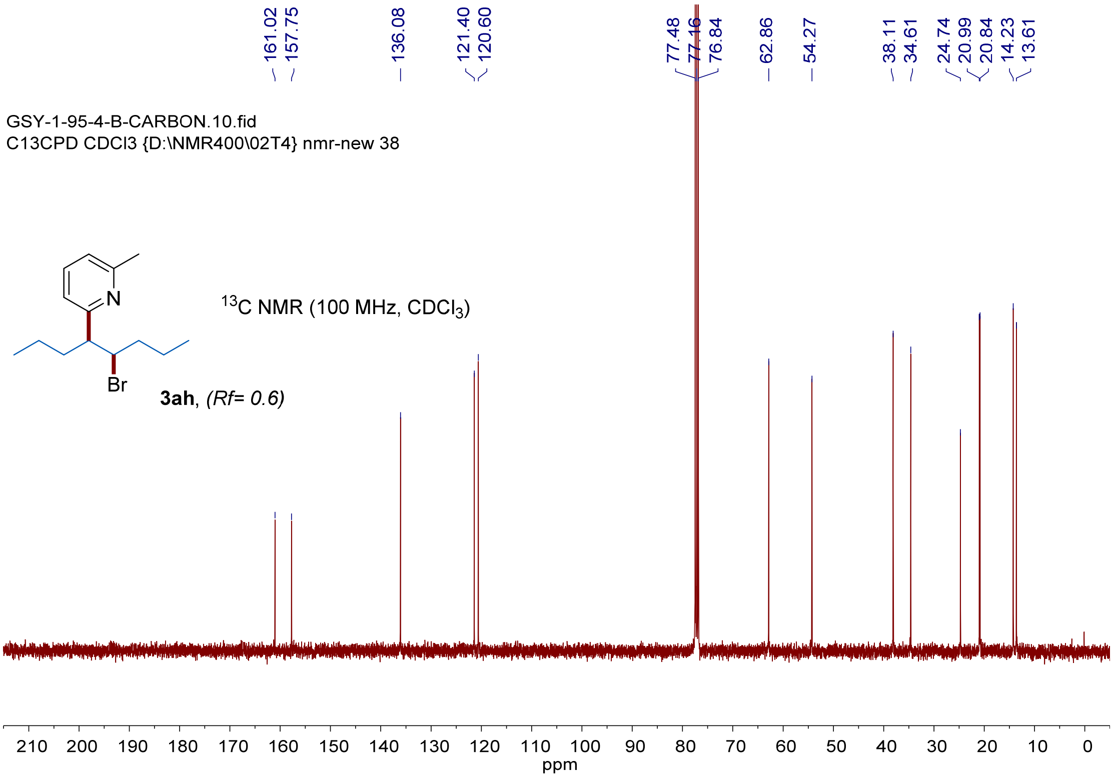

Supplementary Figure 39. ^{13}C NMR spectra of compound 3ah (***Rf* = *0.6***)

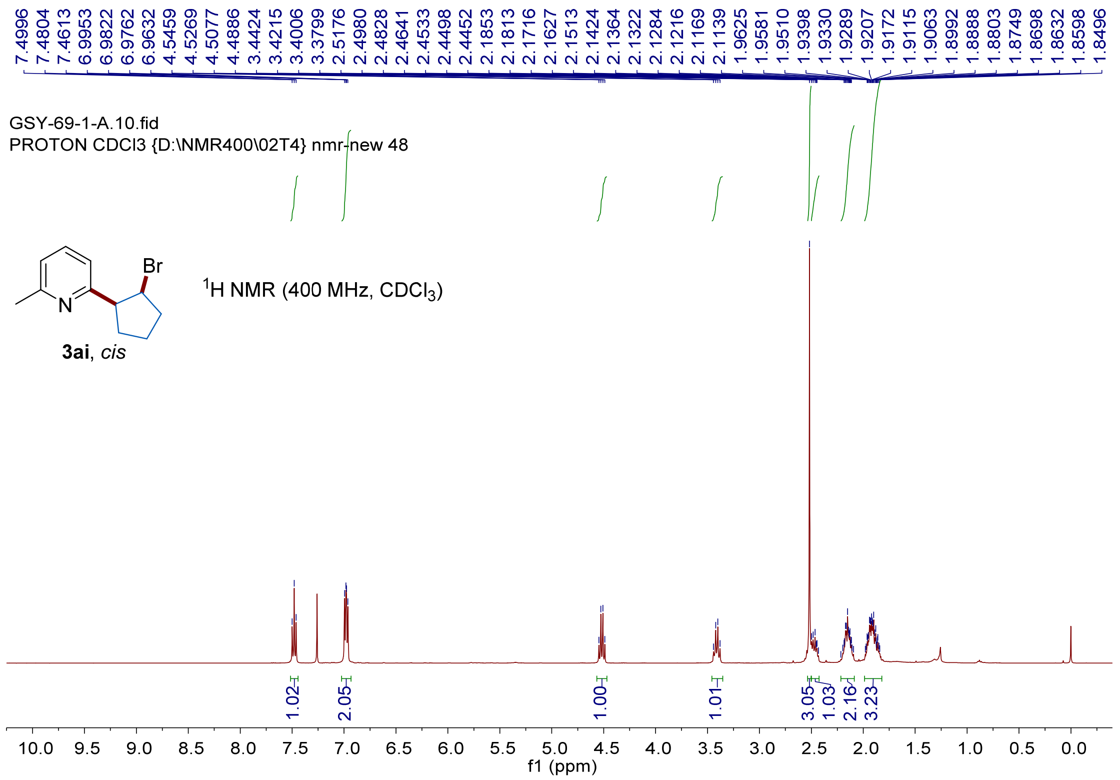

Supplementary Figure 40. ^{1}**H NMR spectra of compound 3ai (*cis*)**

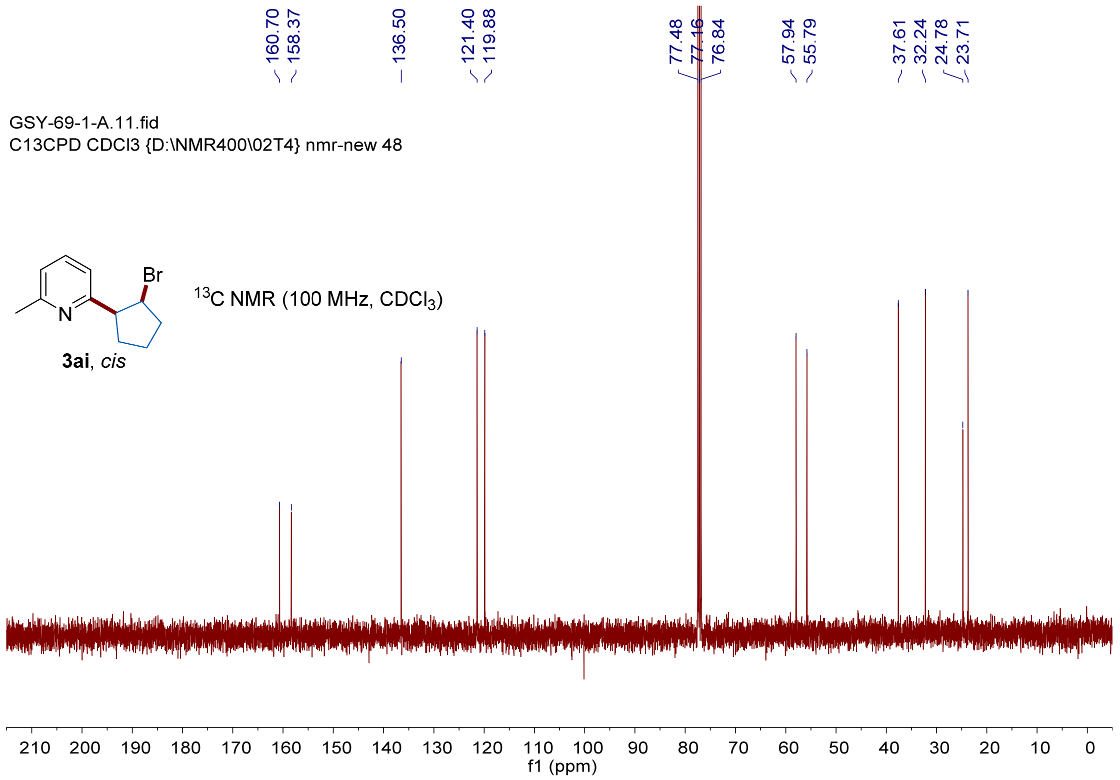

Supplementary Figure 41. ^{13}**C NMR spectra of compound 3ai (*cis*)**

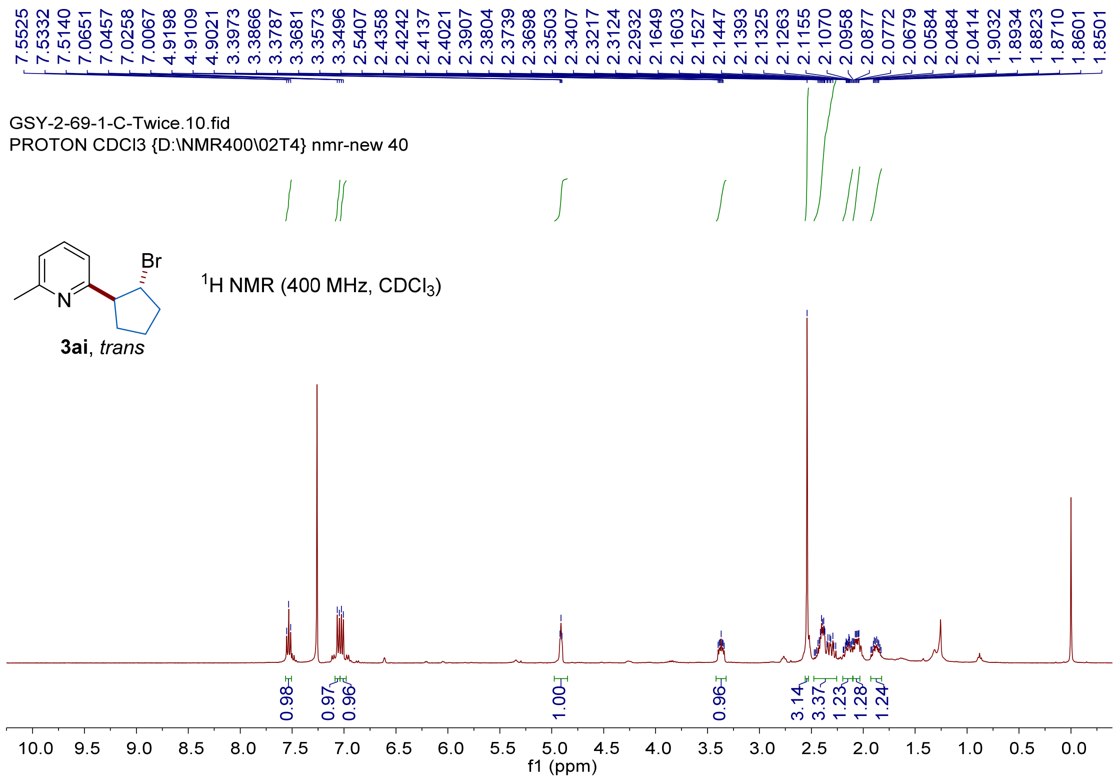

Supplementary Figure 42. ^{1}H NMR spectra of compound 3ai (*trans*)

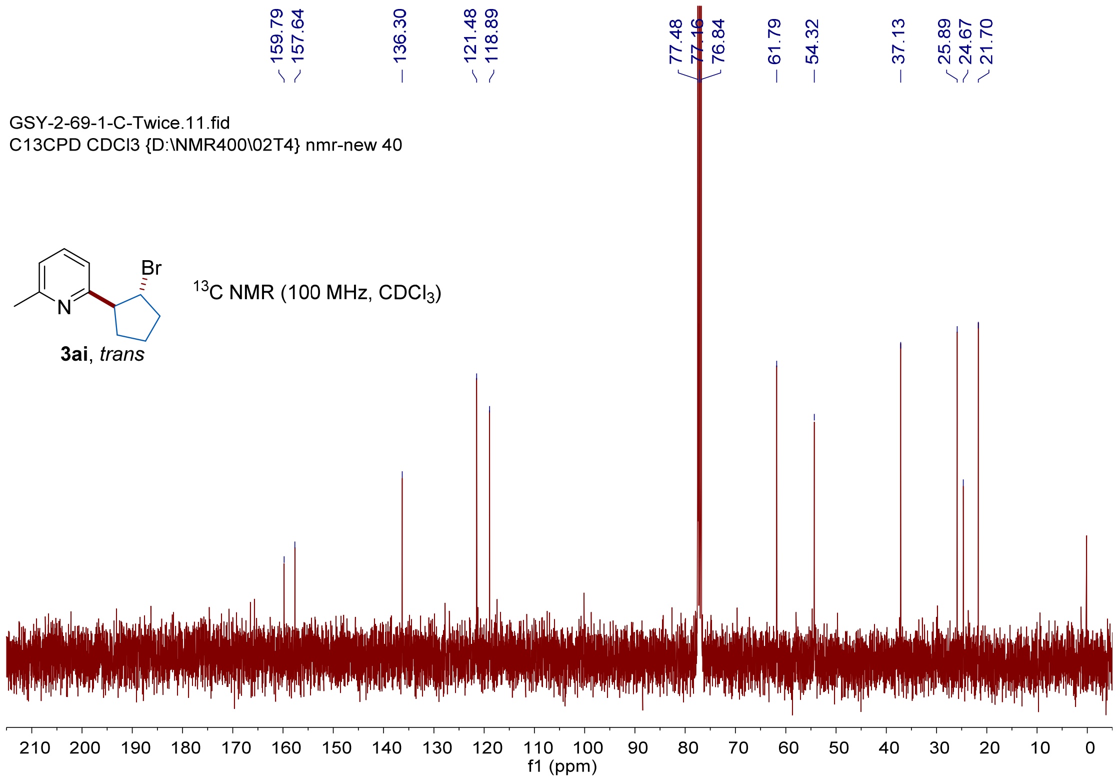

Supplementary Figure 43. ^{13}C NMR spectra of compound 3ai (*trans*)

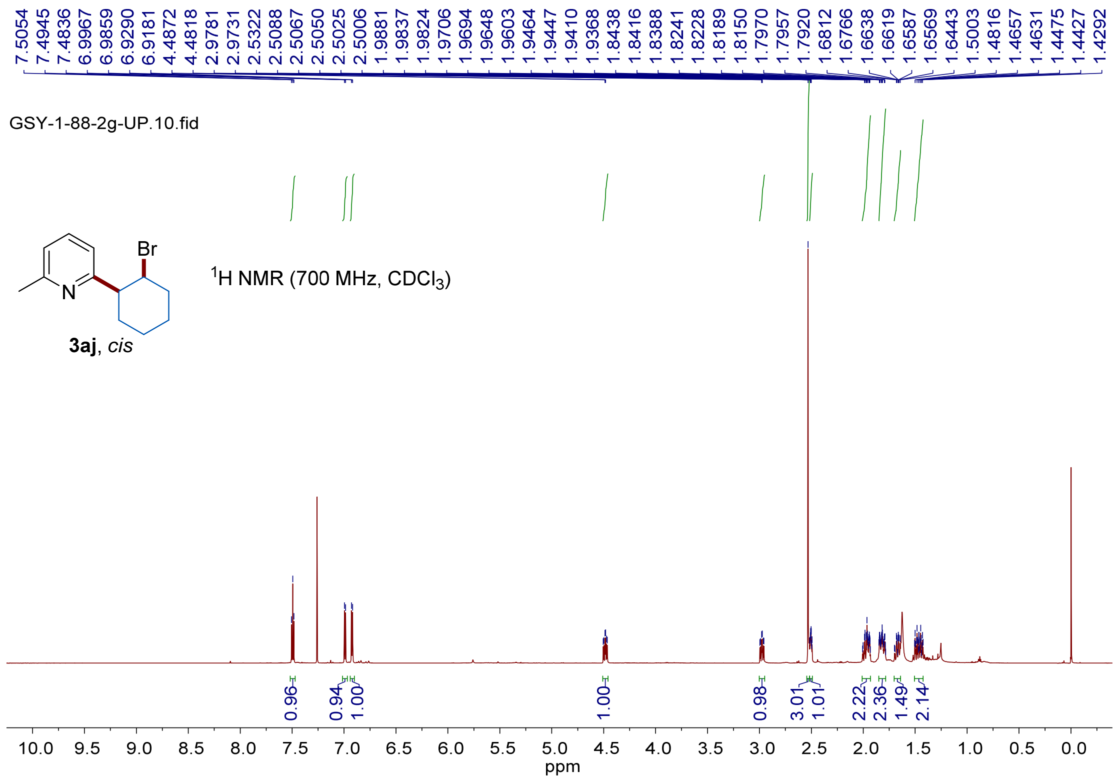

Supplementary Figure 44. ^{1}H NMR spectra of compound 3aj (*cis*)

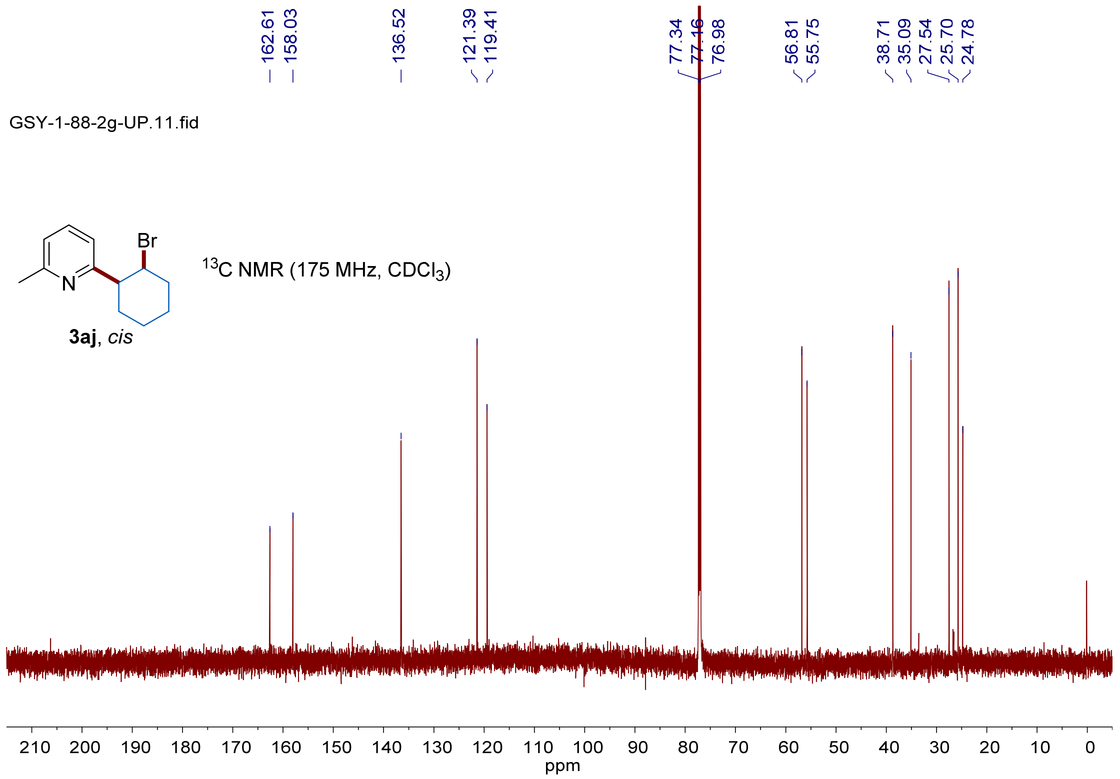

Supplementary Figure 45. ^{13}C NMR spectra of compound 3aj (*cis*)

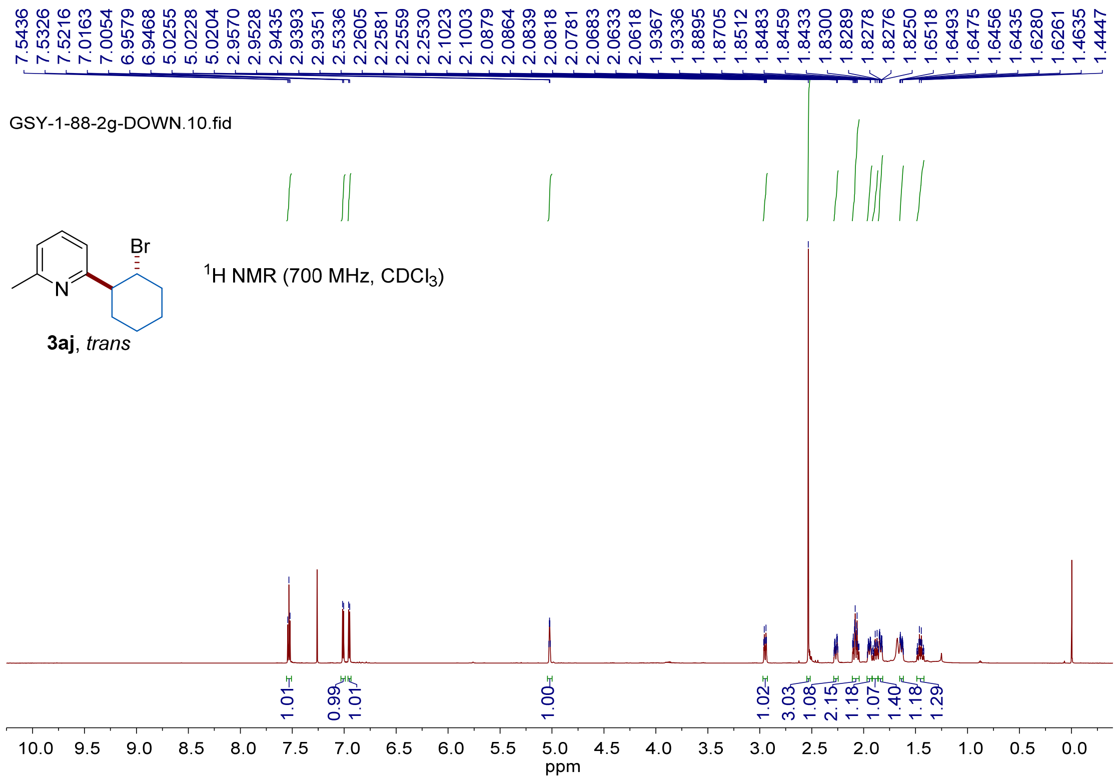

Supplementary Figure 46. ^{1}H NMR spectra of compound 3aj (*trans*)

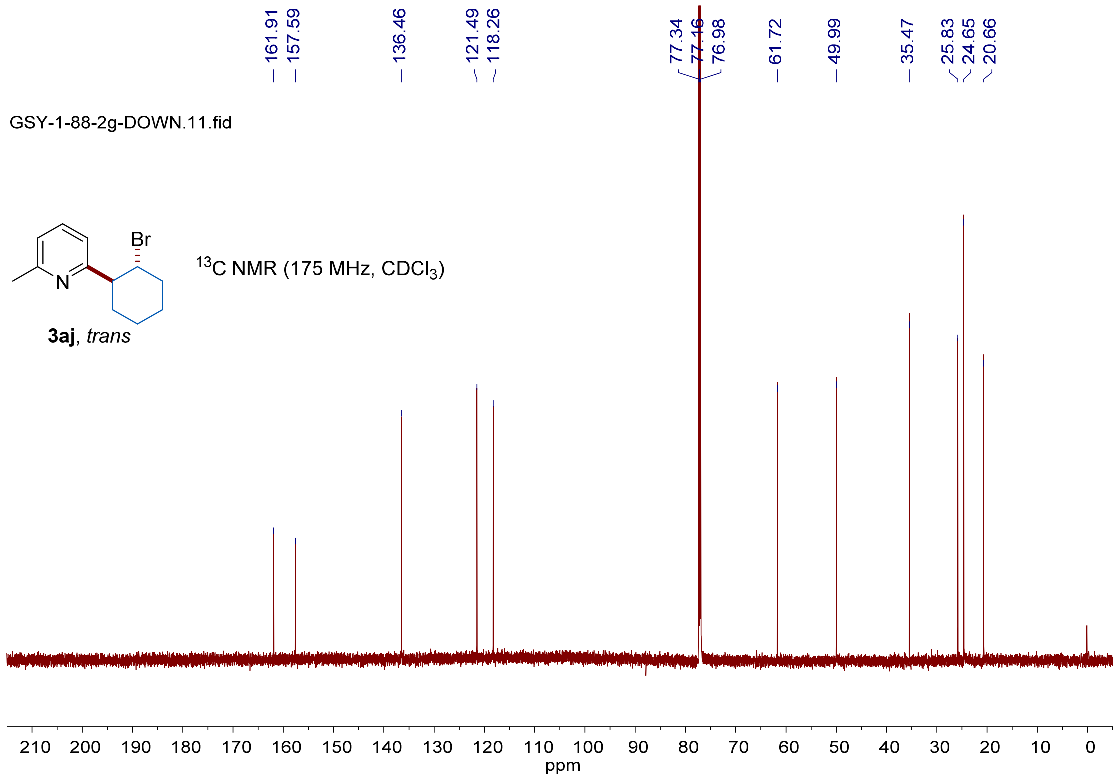

Supplementary Figure 47. ^{13}C NMR spectra of compound 3aj (*trans*)

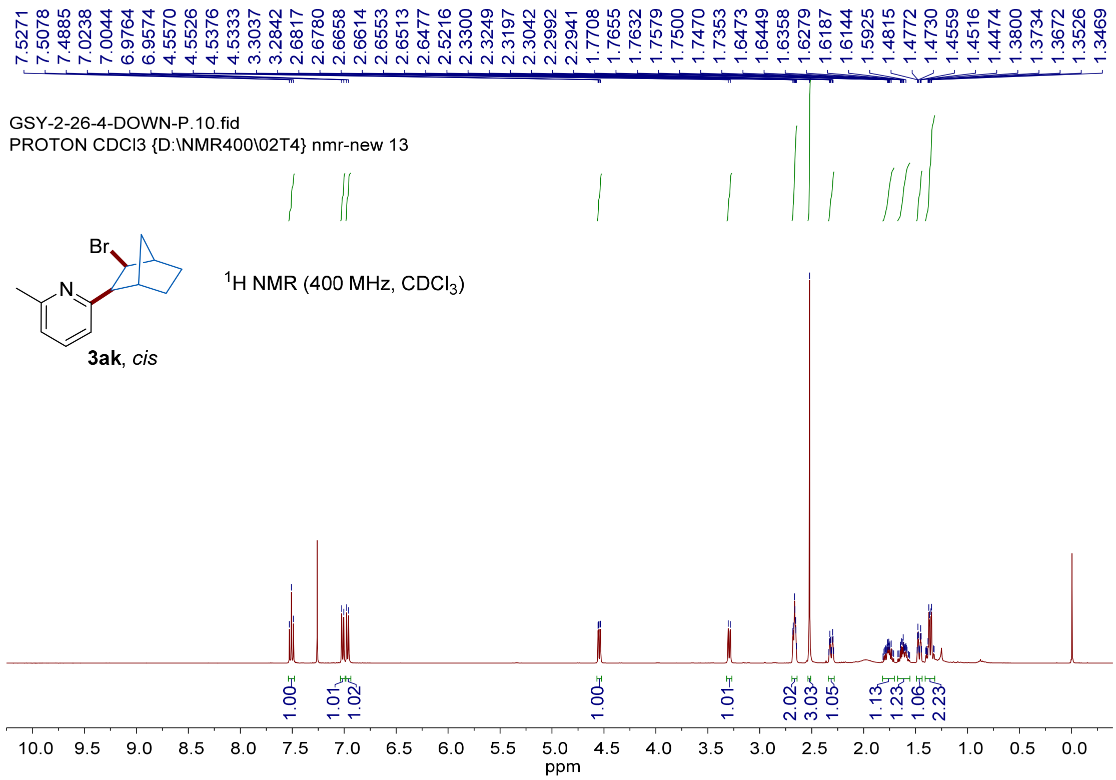

Supplementary Figure 48. ^{1}H NMR spectra of compound 3ak (*cis*)

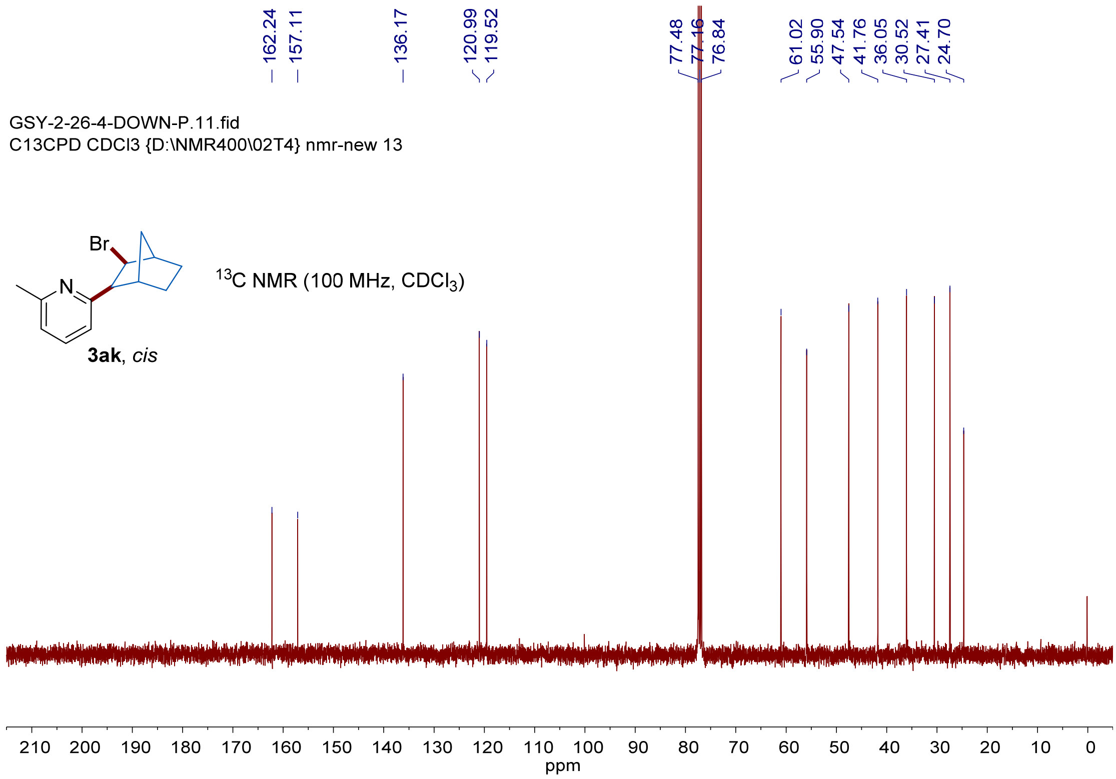

Supplementary Figure 49. ^{13}C NMR spectra of compound 3ak (*cis*)

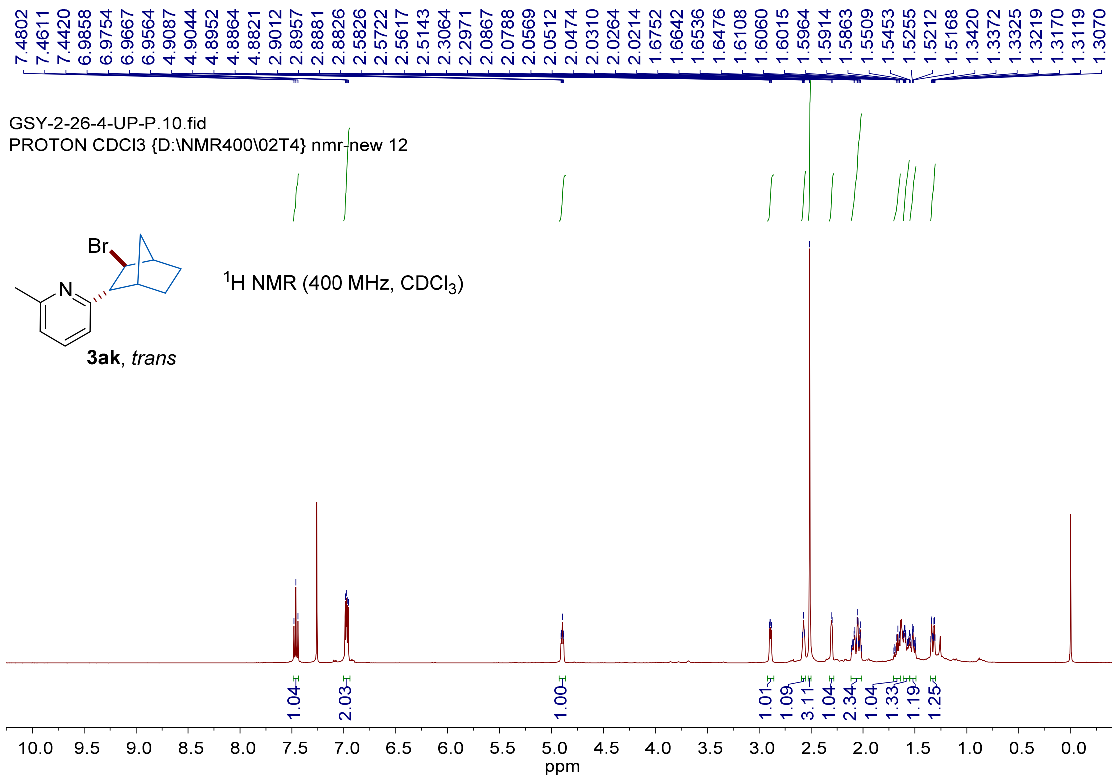

Supplementary Figure 50. ^{1}H NMR spectra of compound 3ak (*trans*)

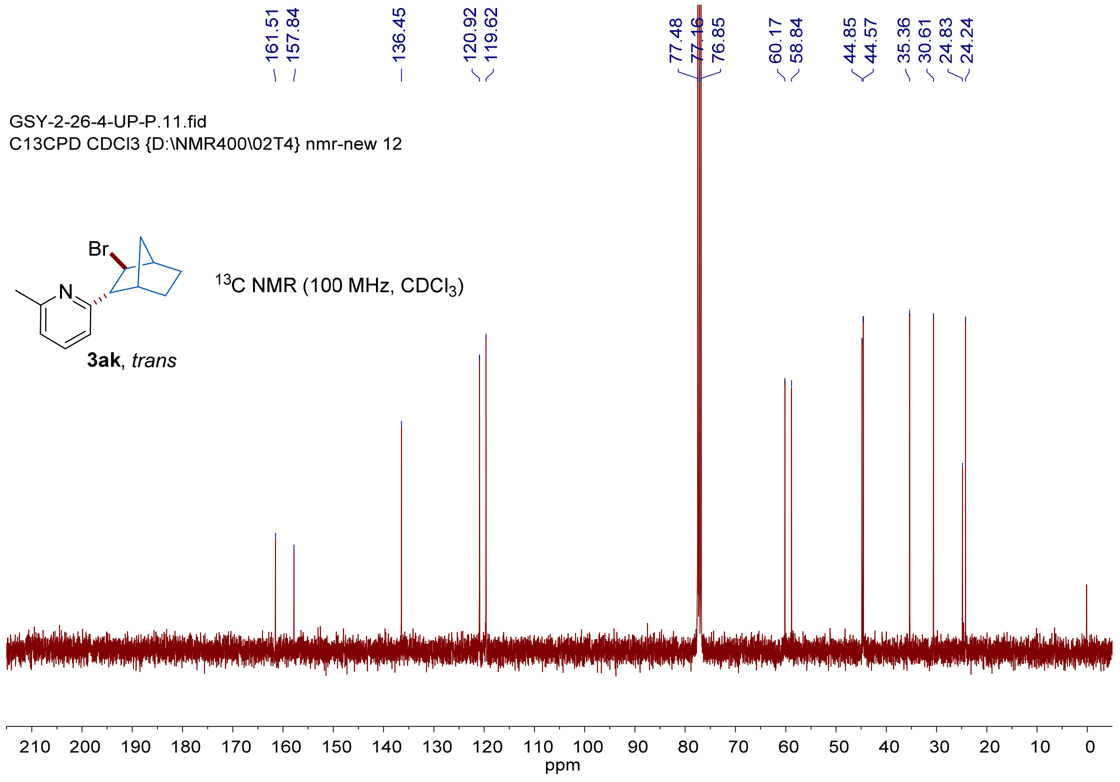

Supplementary Figure 51. ^{13}C NMR spectra of compound 3ak (*trans*)

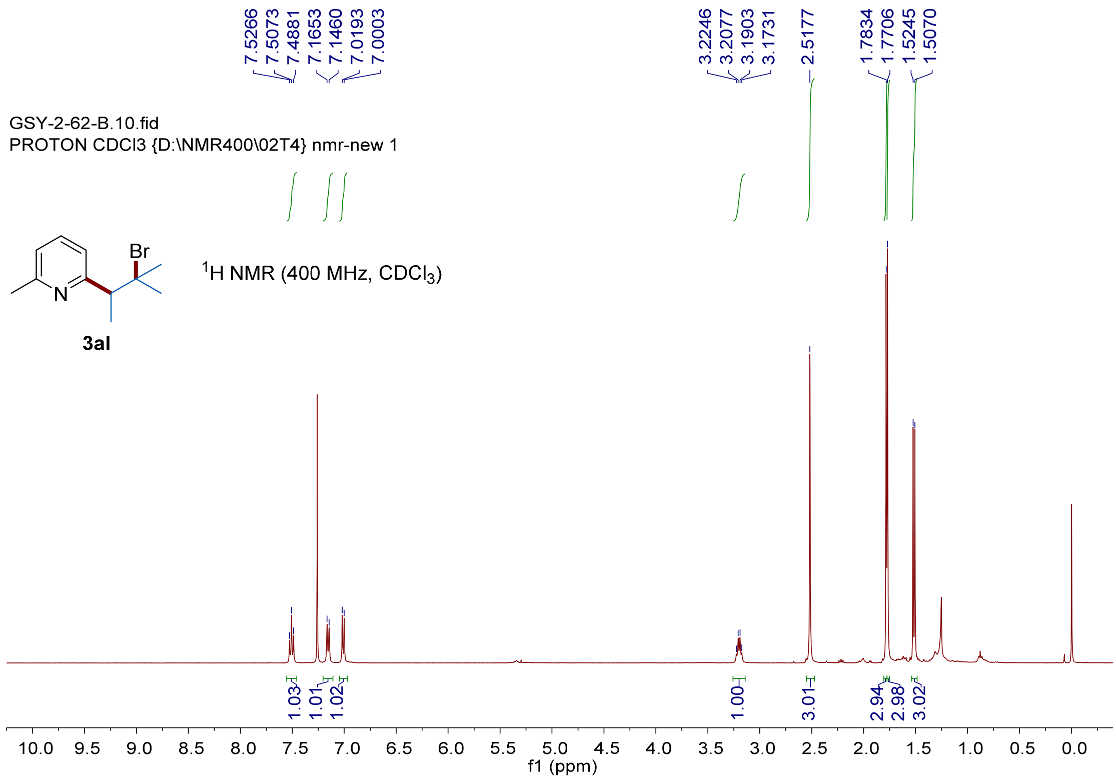

Supplementary Figure 52. ^{1}H NMR spectra of compound 3al

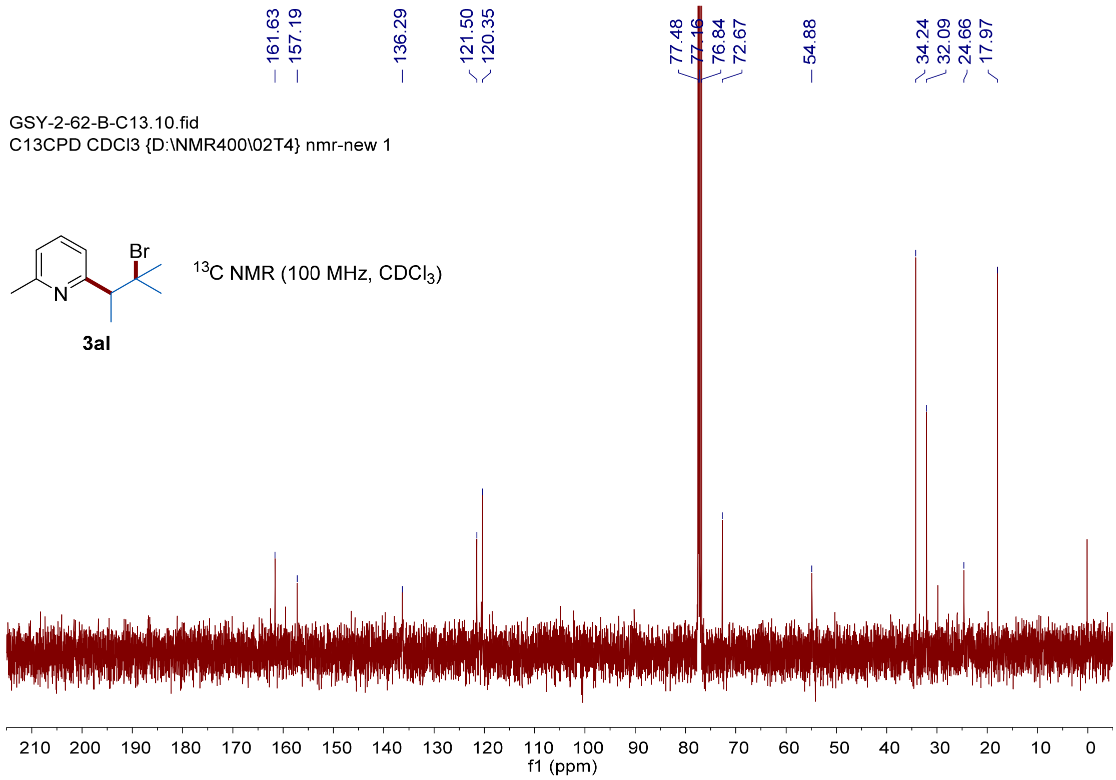

Supplementary Figure 53. ^{13}C NMR spectra of compound 3al

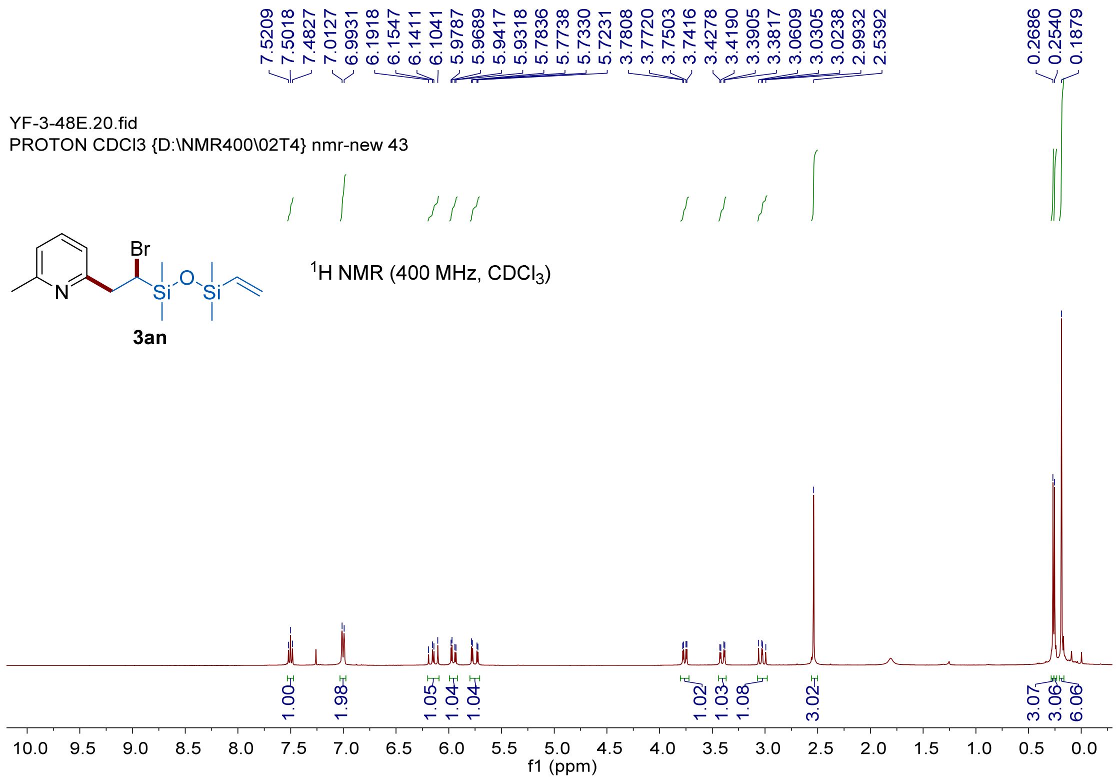

Supplementary Figure 54. ^{1}**H NMR spectra of compound 3an**

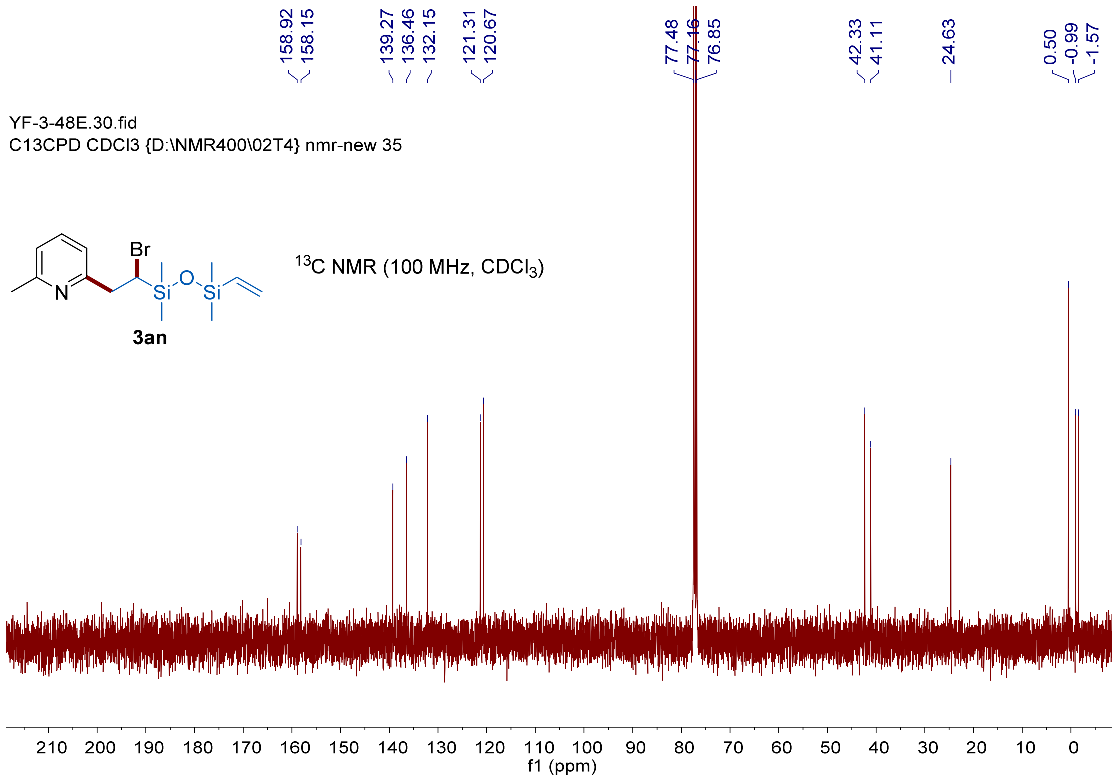

Supplementary Figure 55. ^{13}**C NMR spectra of compound 3an**

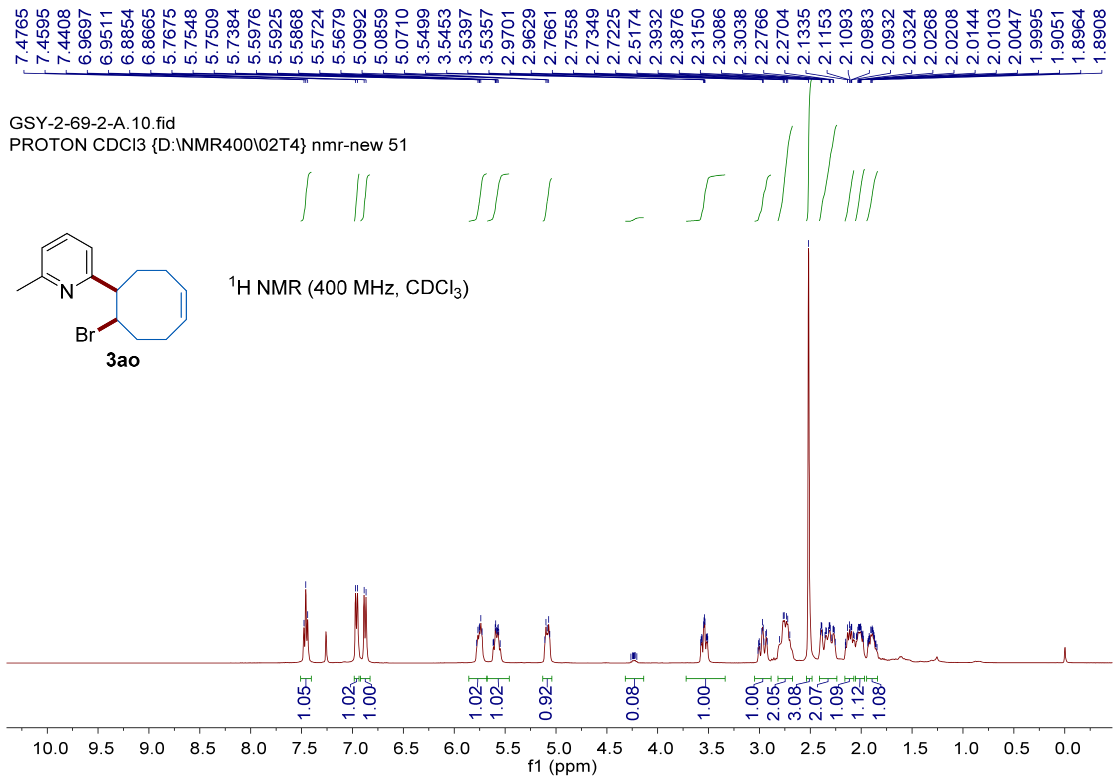

Supplementary Figure 56. ^{1}H NMR spectra of compound 3ao

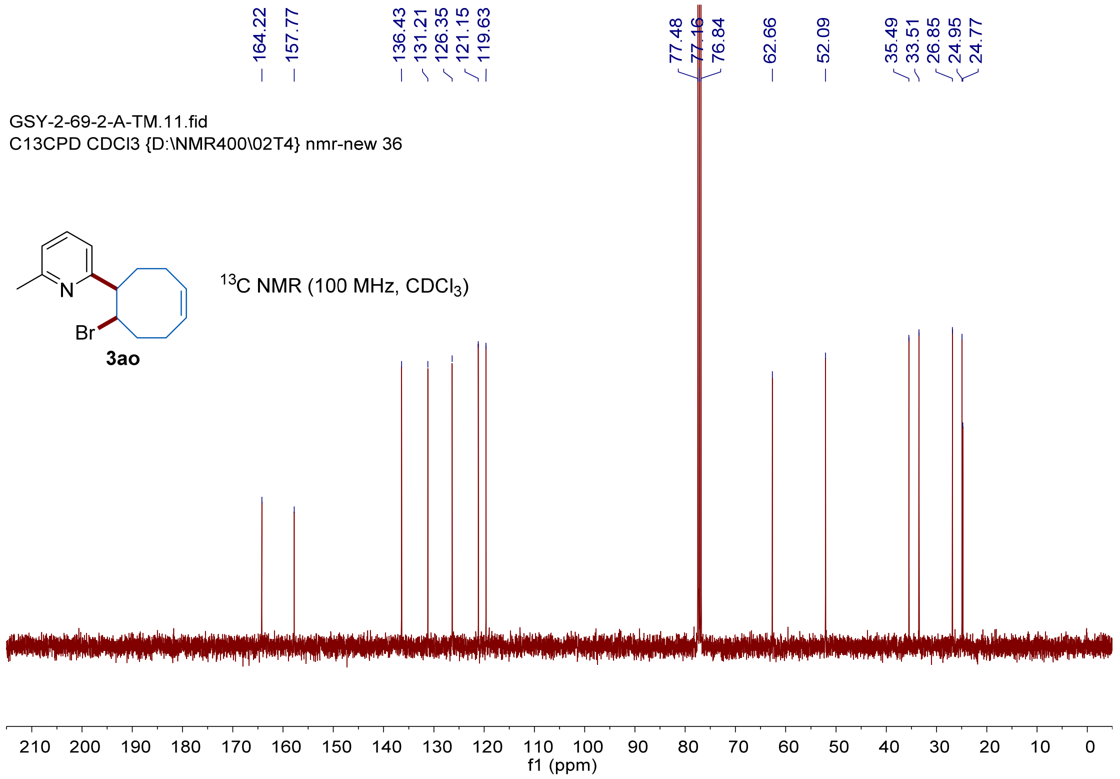

Supplementary Figure 57. ^{13}C NMR spectra of compound 3ao

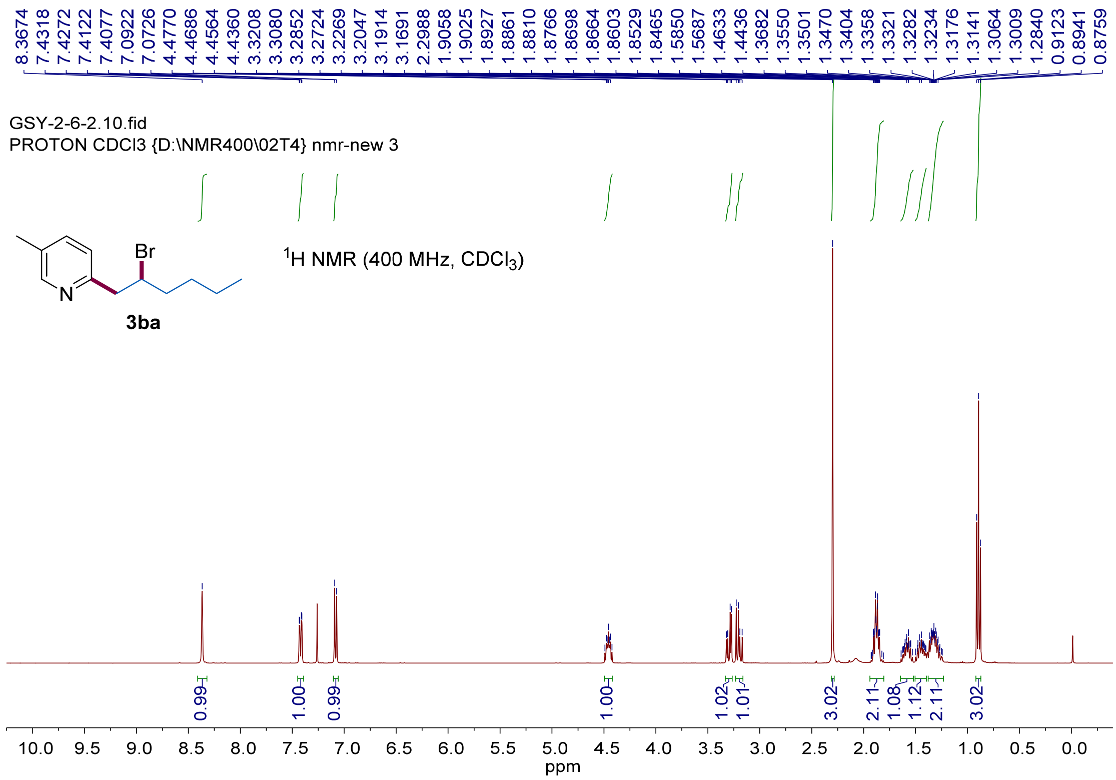

Supplementary Figure 58. ^{1}H NMR spectra of compound 3ba

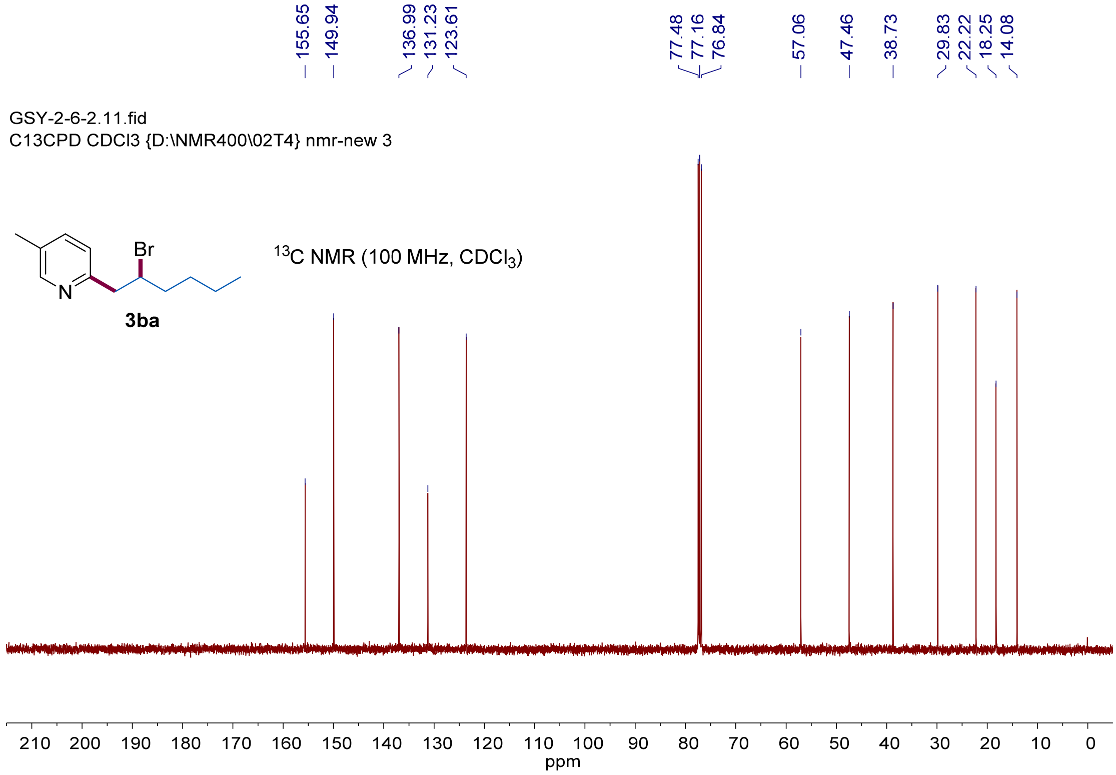

Supplementary Figure 59. ^{13}C NMR spectra of compound 3ba

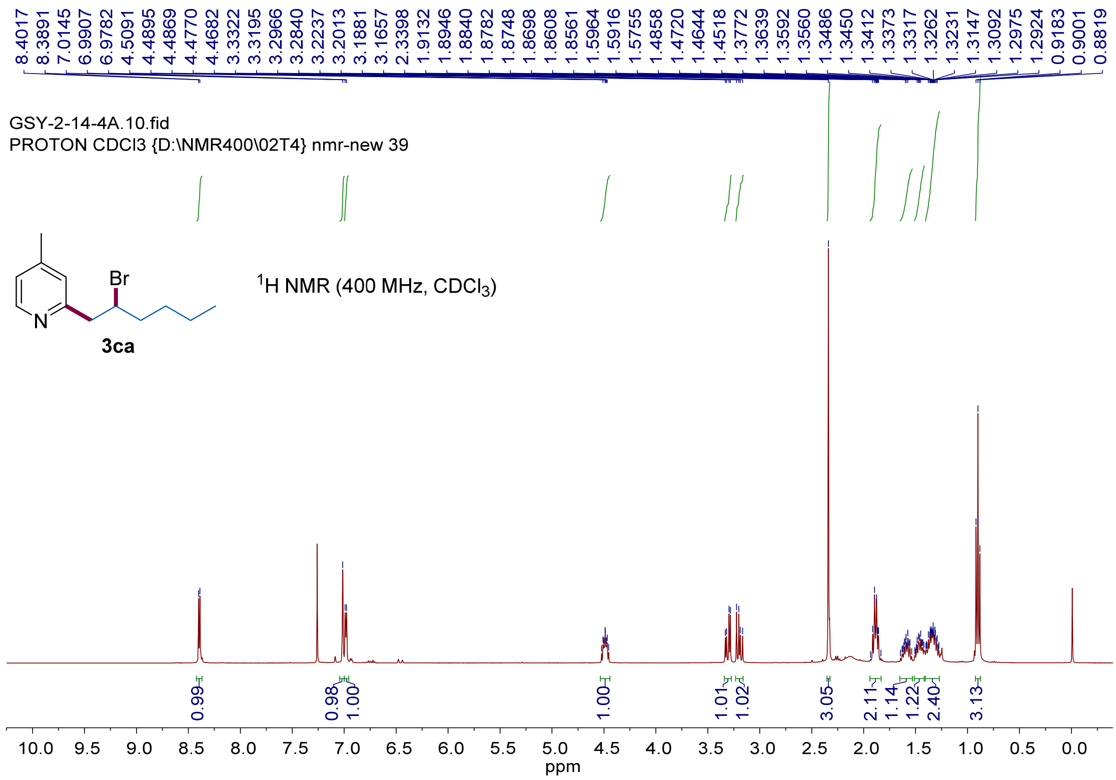

Supplementary Figure 60. ^{1}H NMR spectra of compound 3ca

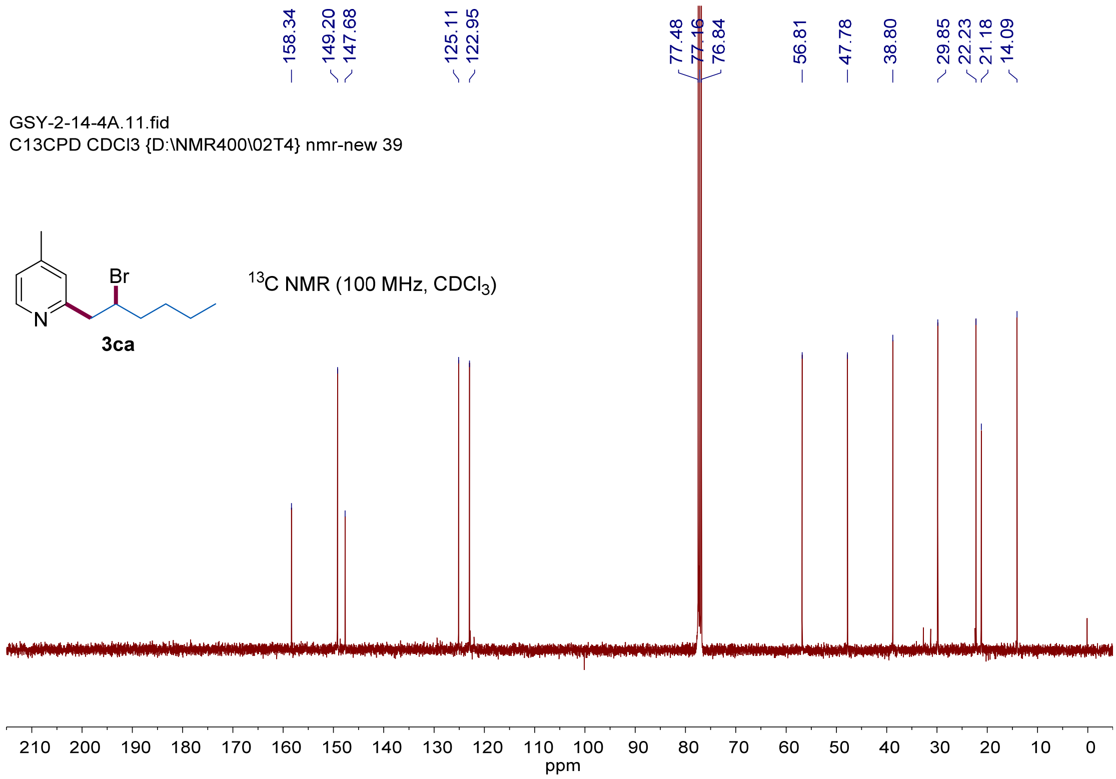

Supplementary Figure 61. ^{13}C NMR spectra of compound 3ca

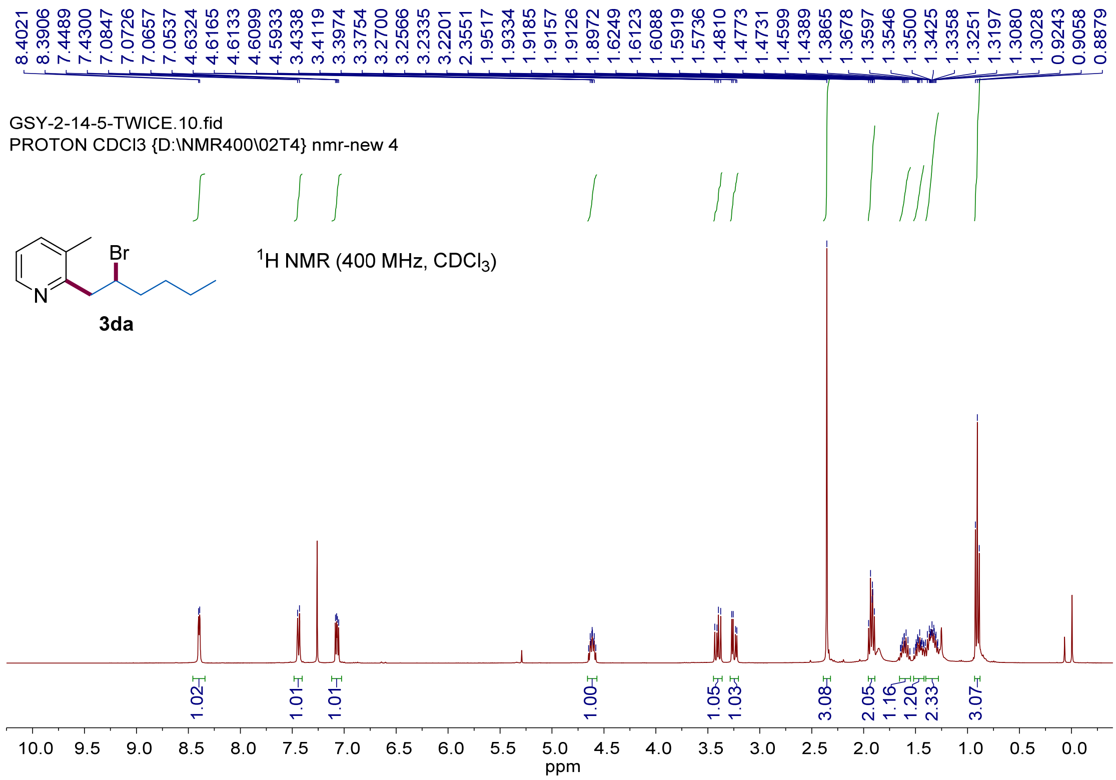

Supplementary Figure 62. ^{1}H NMR spectra of compound 3da

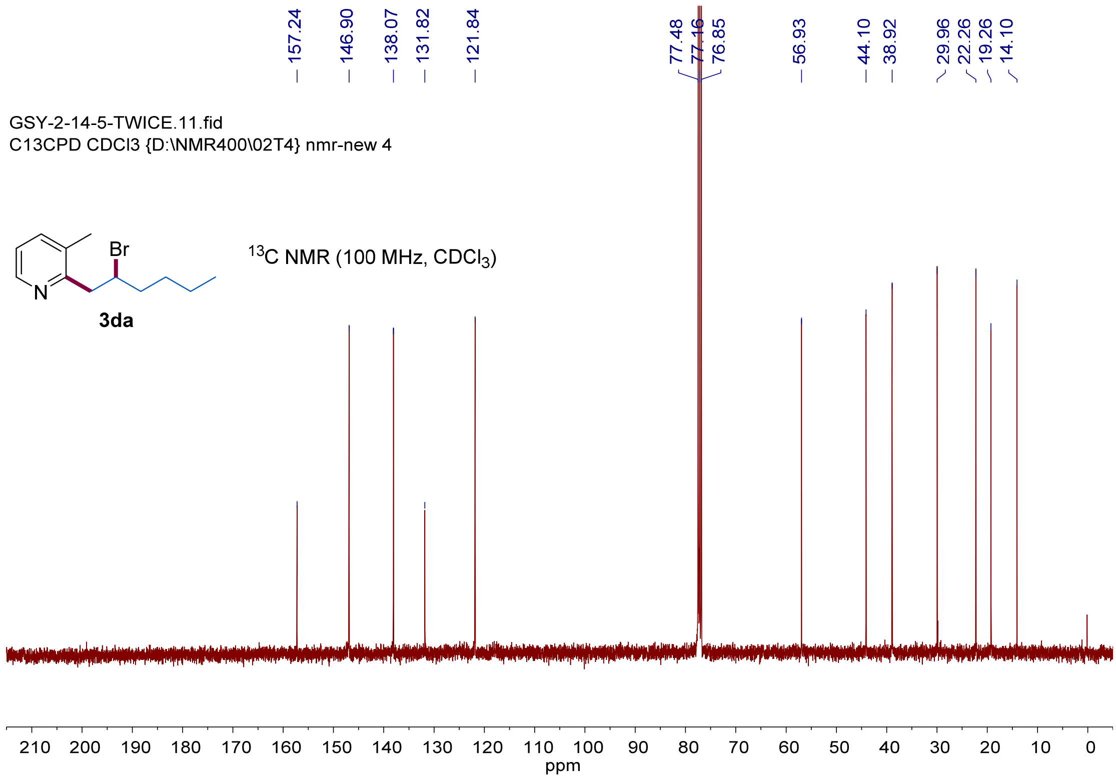

Supplementary Figure 63. ^{13}C NMR spectra of compound 3da

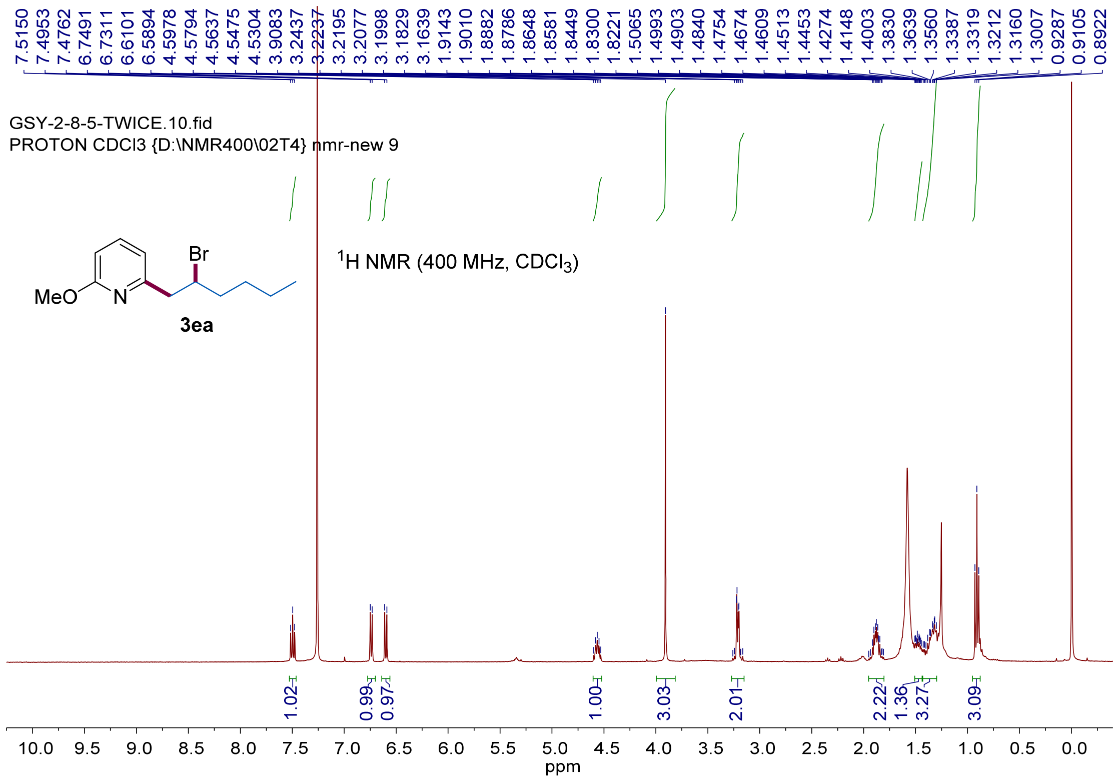

Supplementary Figure 64. ^{1}H NMR spectra of compound 3ea

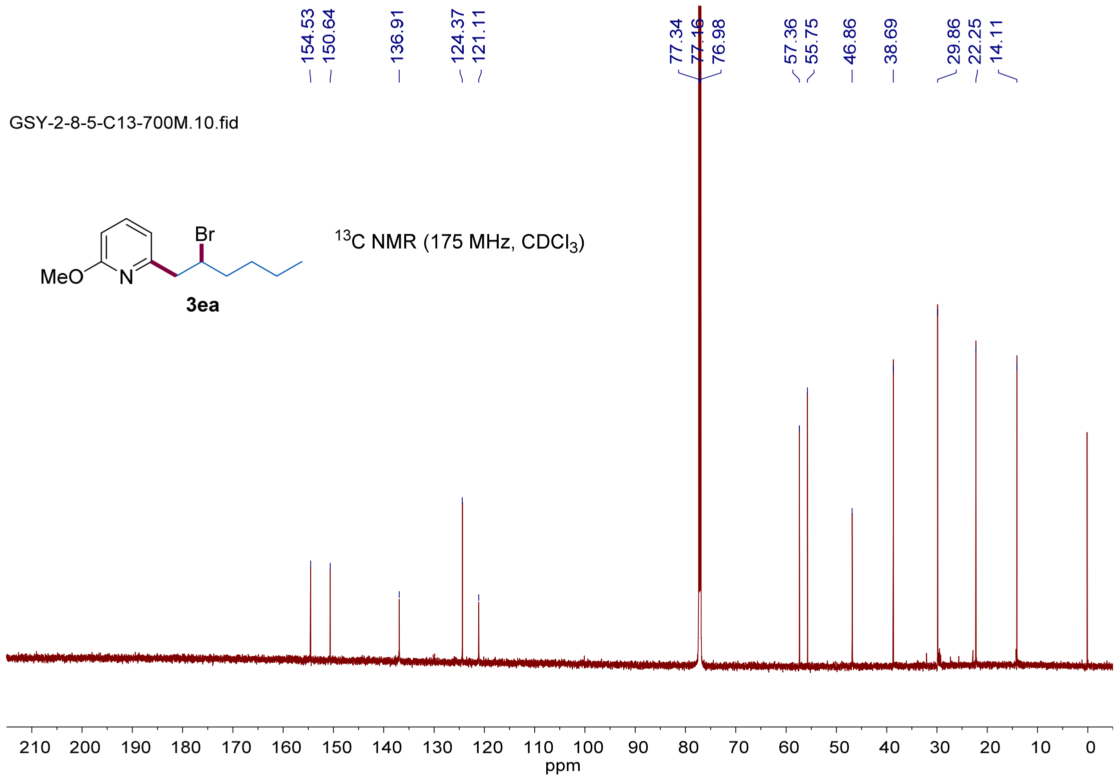

Supplementary Figure 65. ^{13}C NMR spectra of compound 3ea

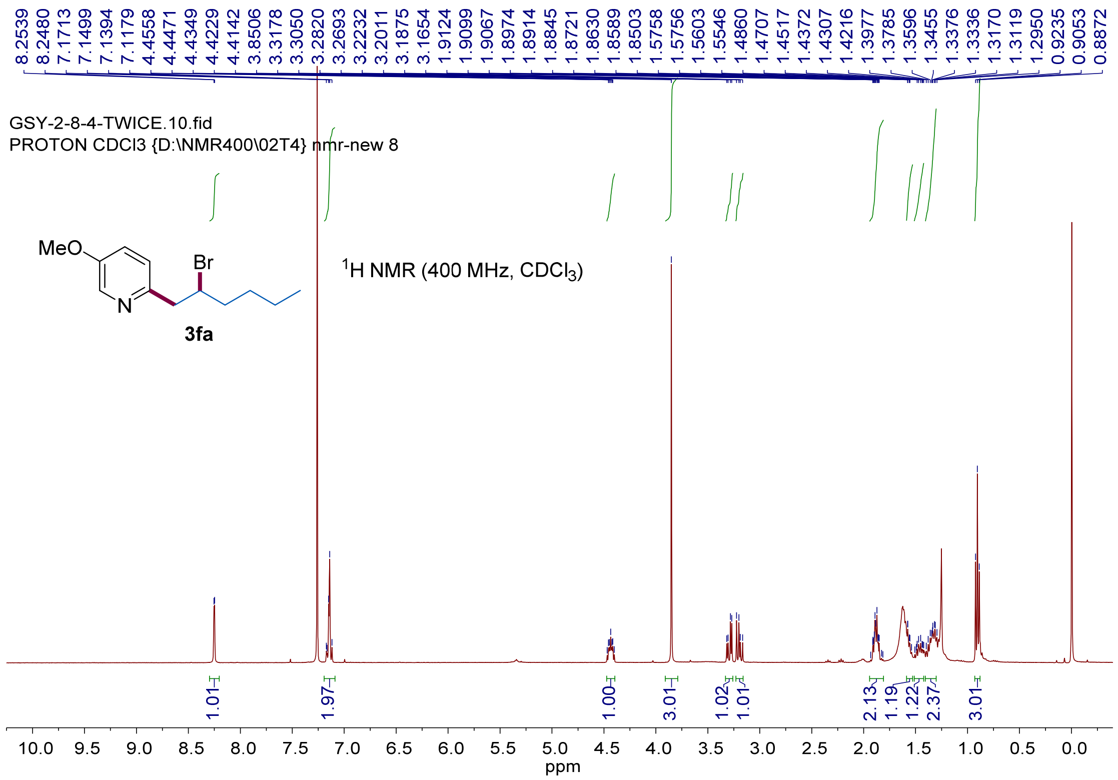

Supplementary Figure 66. ^{1}H NMR spectra of compound 3fa

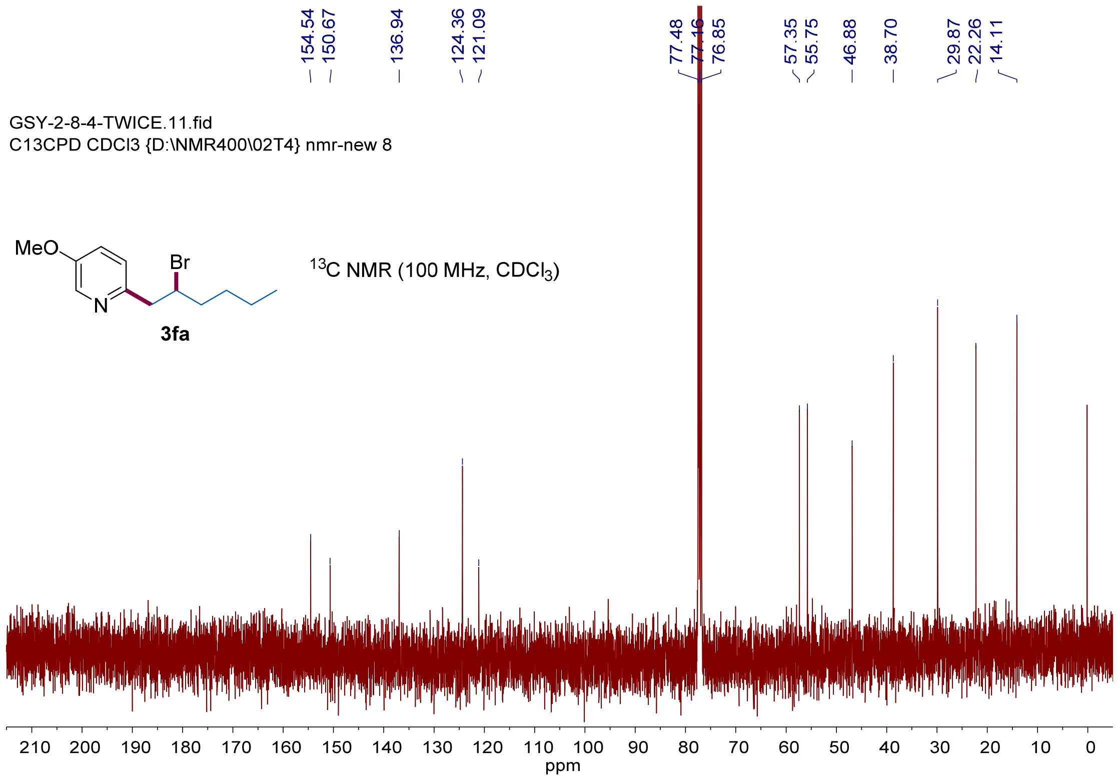

Supplementary Figure 67. ^{13}C NMR spectra of compound 3fa

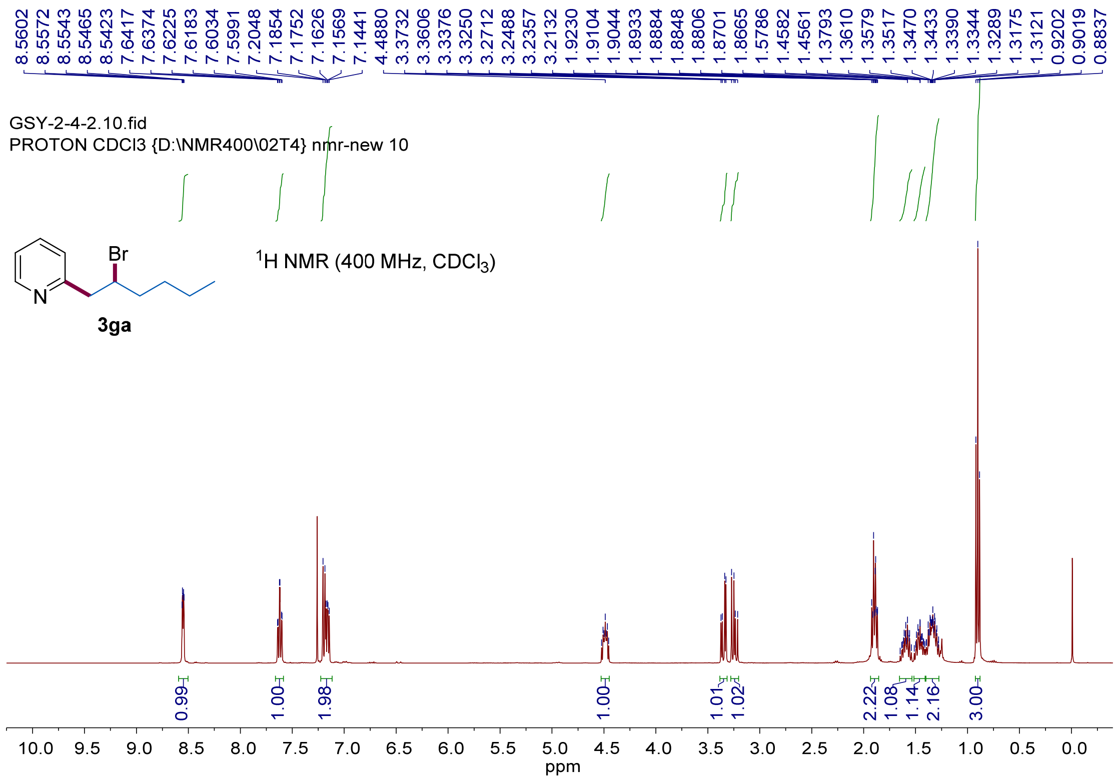

Supplementary Figure 68. ^{1}H NMR spectra of compound 3ga

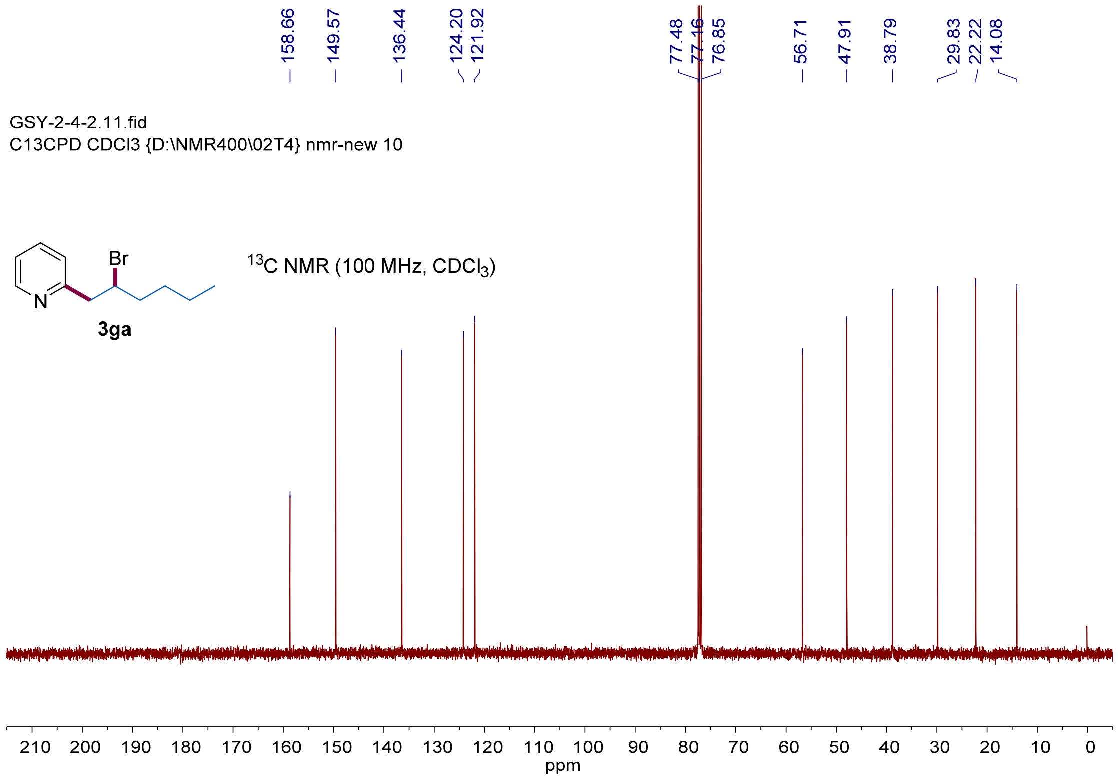

Supplementary Figure 69. ^{13}C NMR spectra of compound 3ga

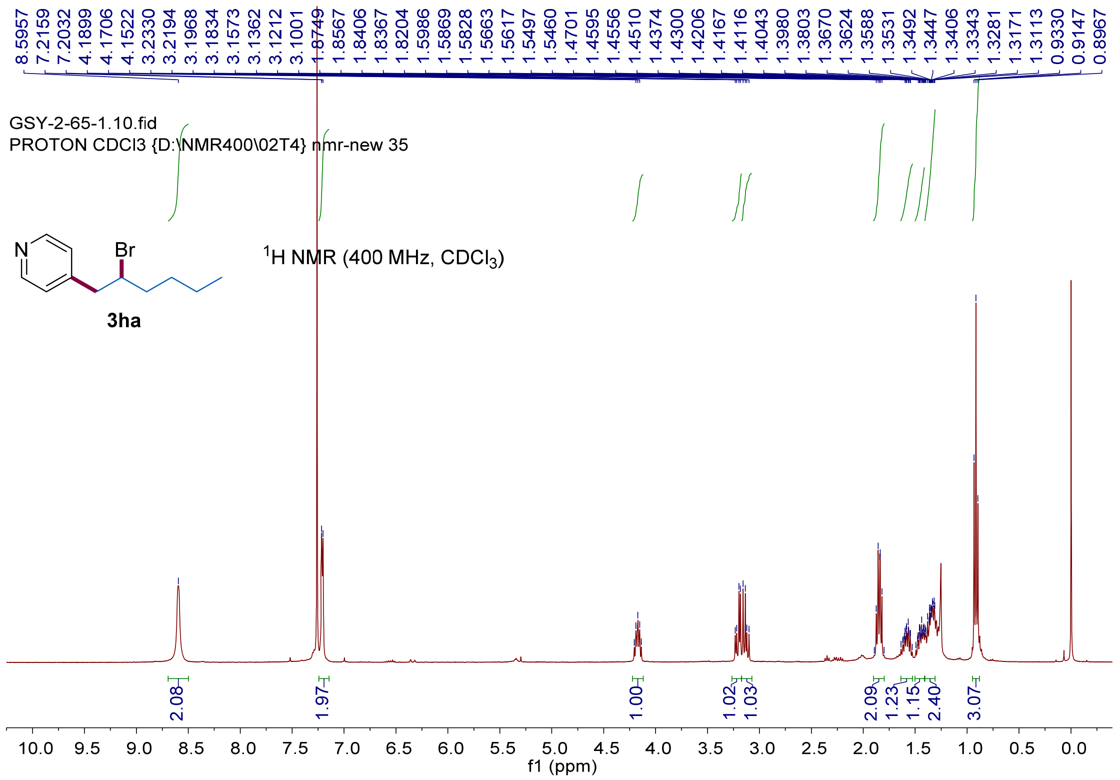

Supplementary Figure 70. ^{1}H NMR spectra of compound 3ha

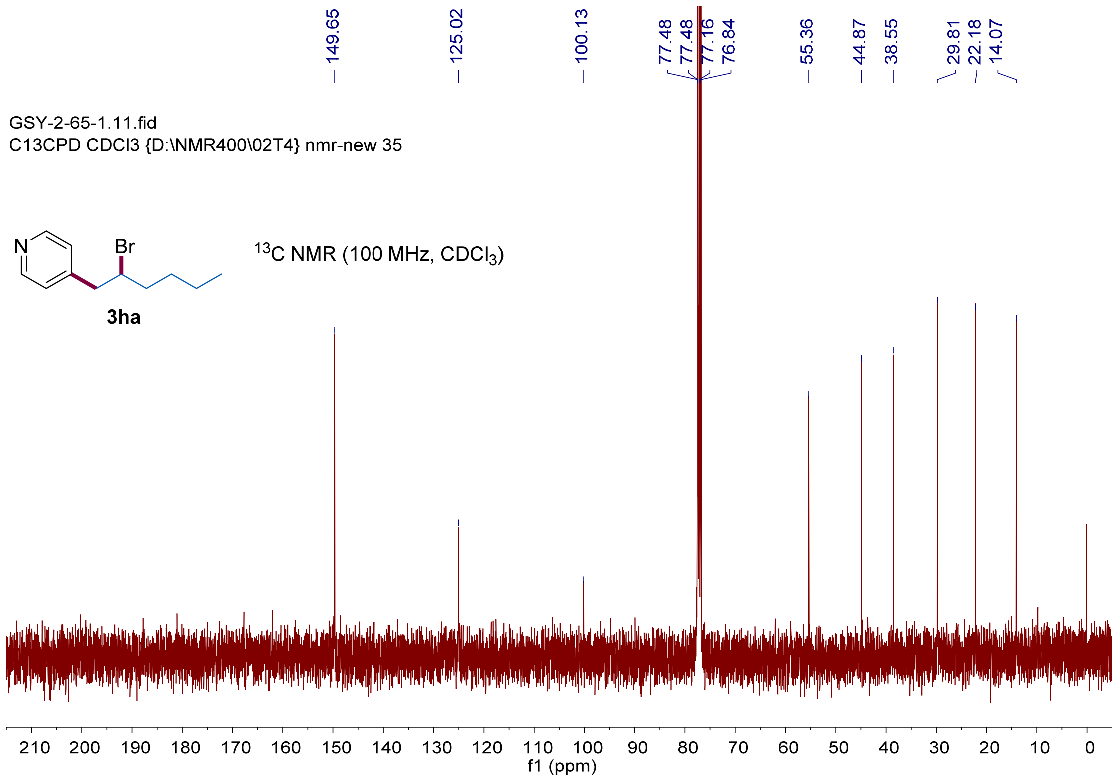

Supplementary Figure 71. ^{13}C NMR spectra of compound 3ha

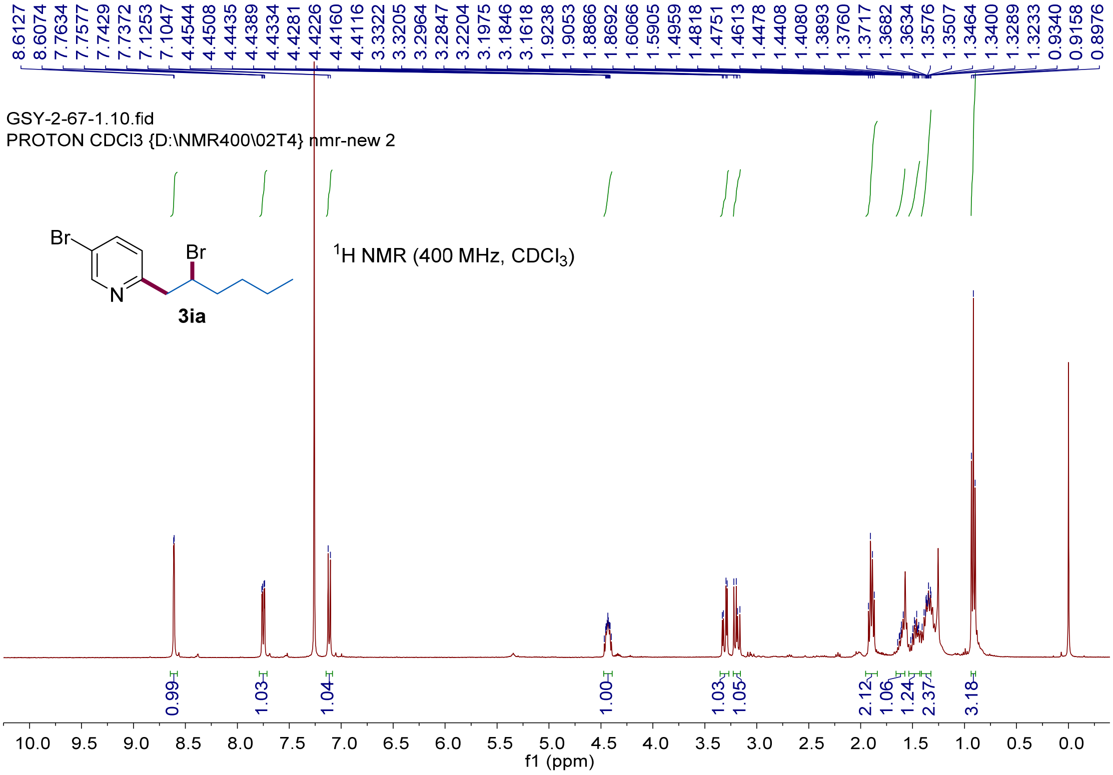

Supplementary Figure 72. ^{1}H NMR spectra of compound 3ia

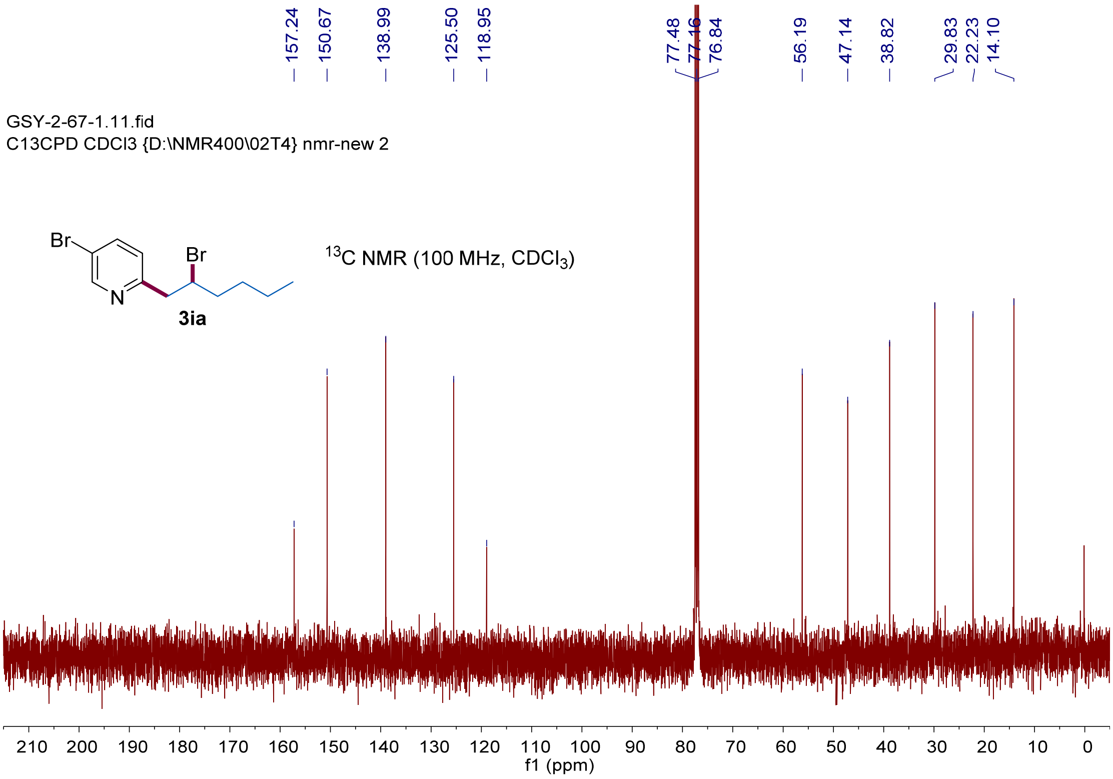

Supplementary Figure 73. ^{13}C NMR spectra of compound 3ia

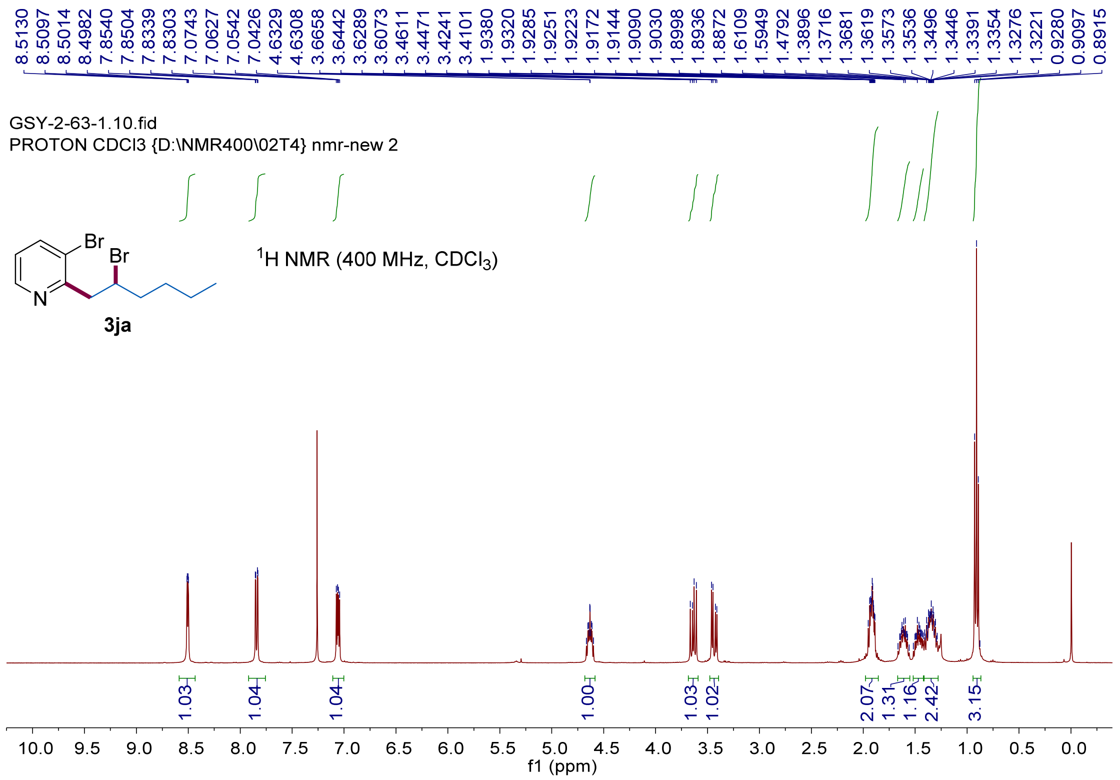

Supplementary Figure 74. ^{1}H NMR spectra of compound 3ja

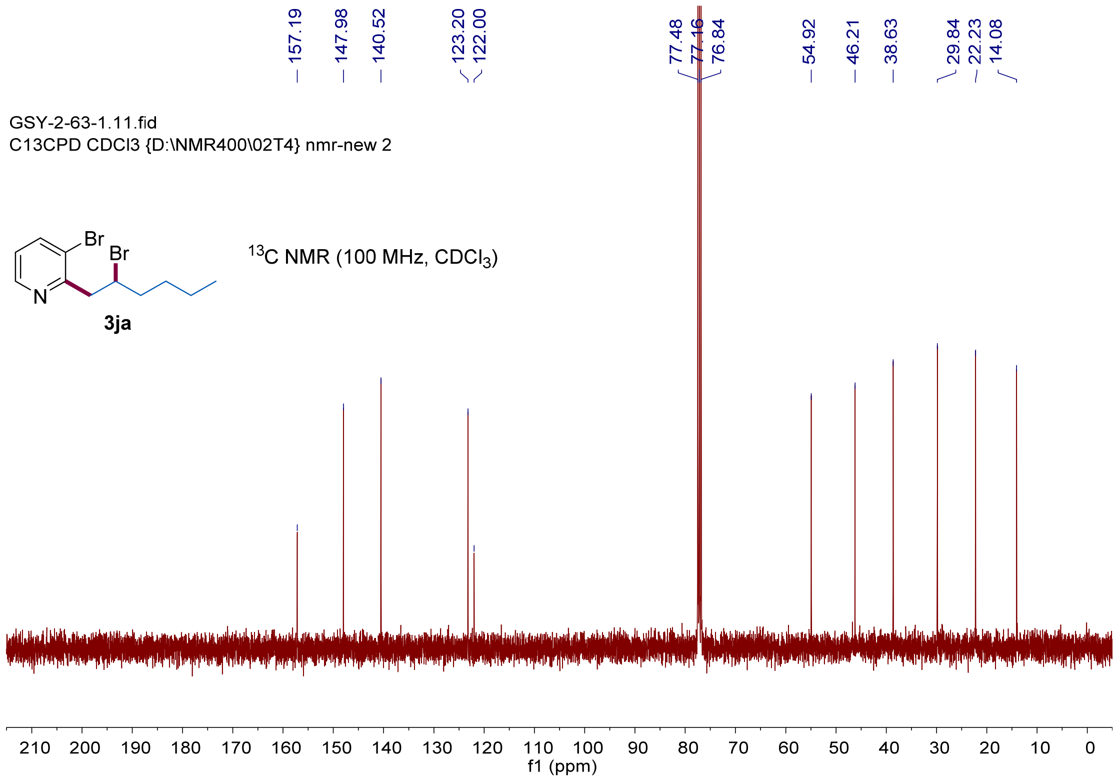

Supplementary Figure 75. ^{13}C NMR spectra of compound 3ja

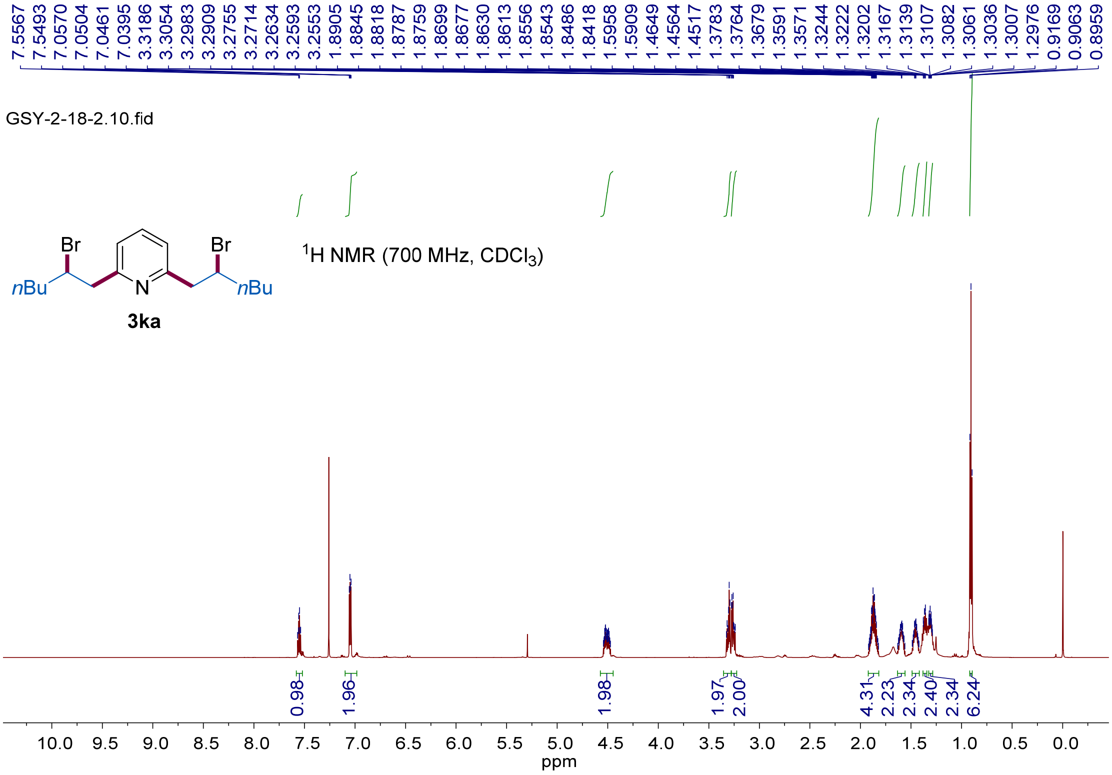

Supplementary Figure 76. ^{1}H NMR spectra of compound 3ka

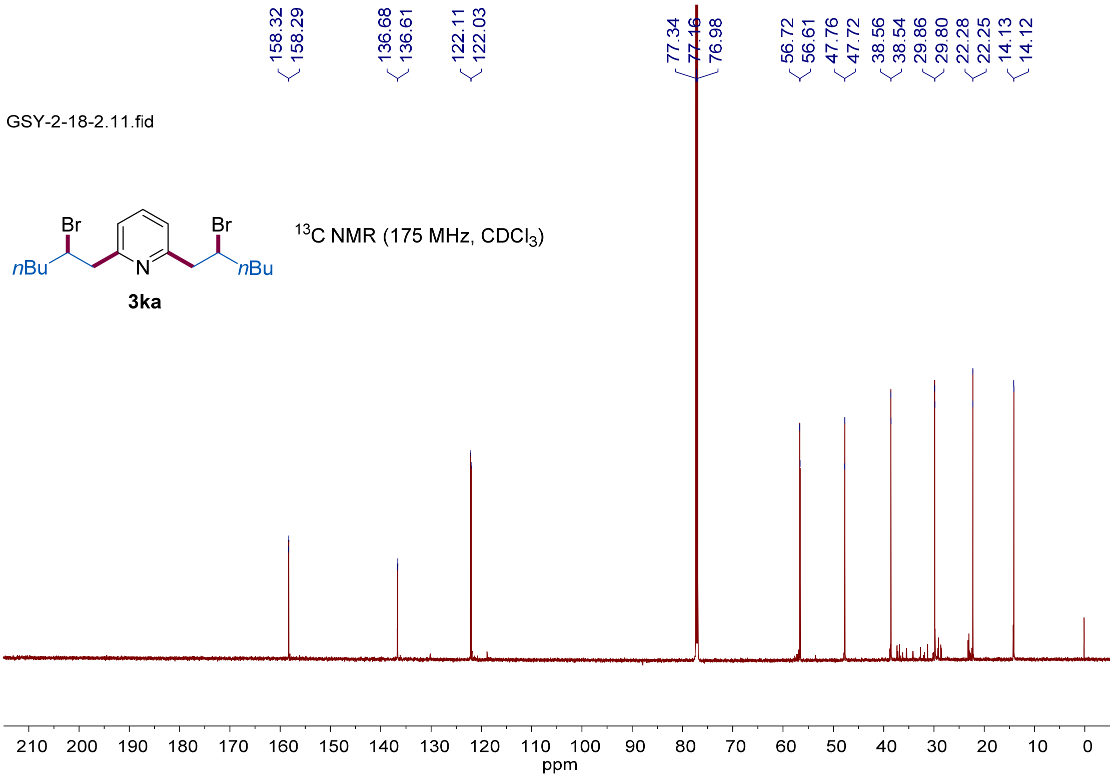

Supplementary Figure 77. ^{13}C NMR spectra of compound 3ka

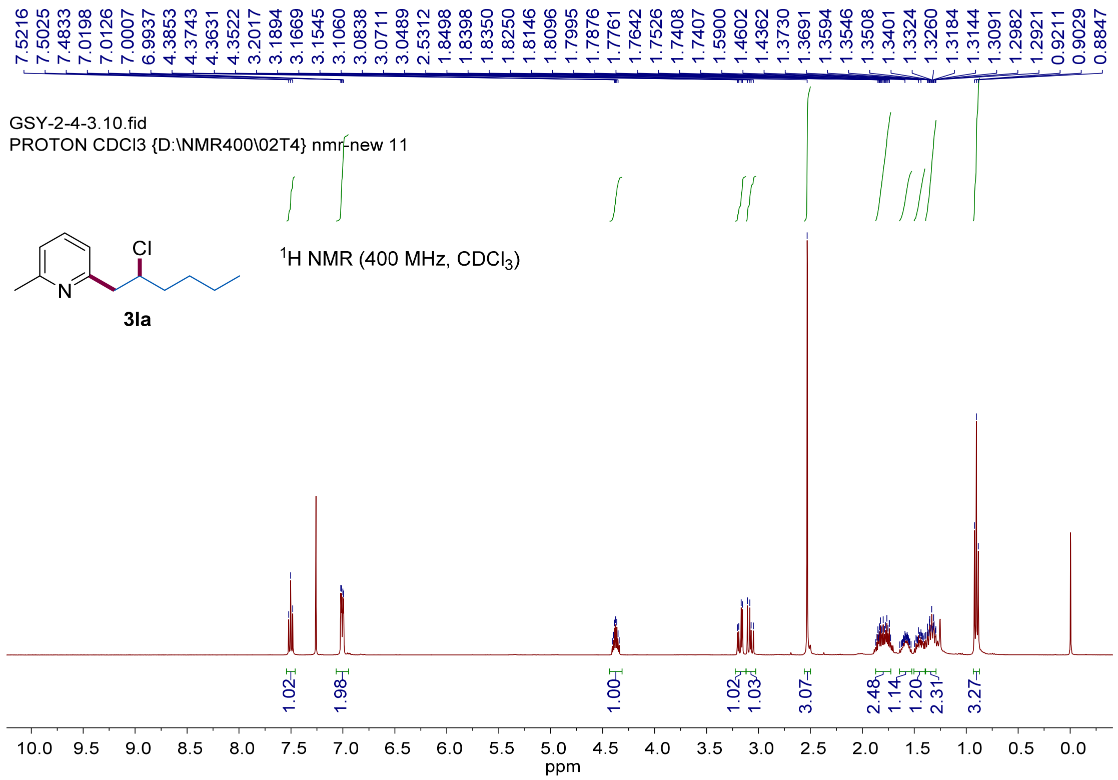

Supplementary Figure 78. ^{1}H NMR spectra of compound 3la

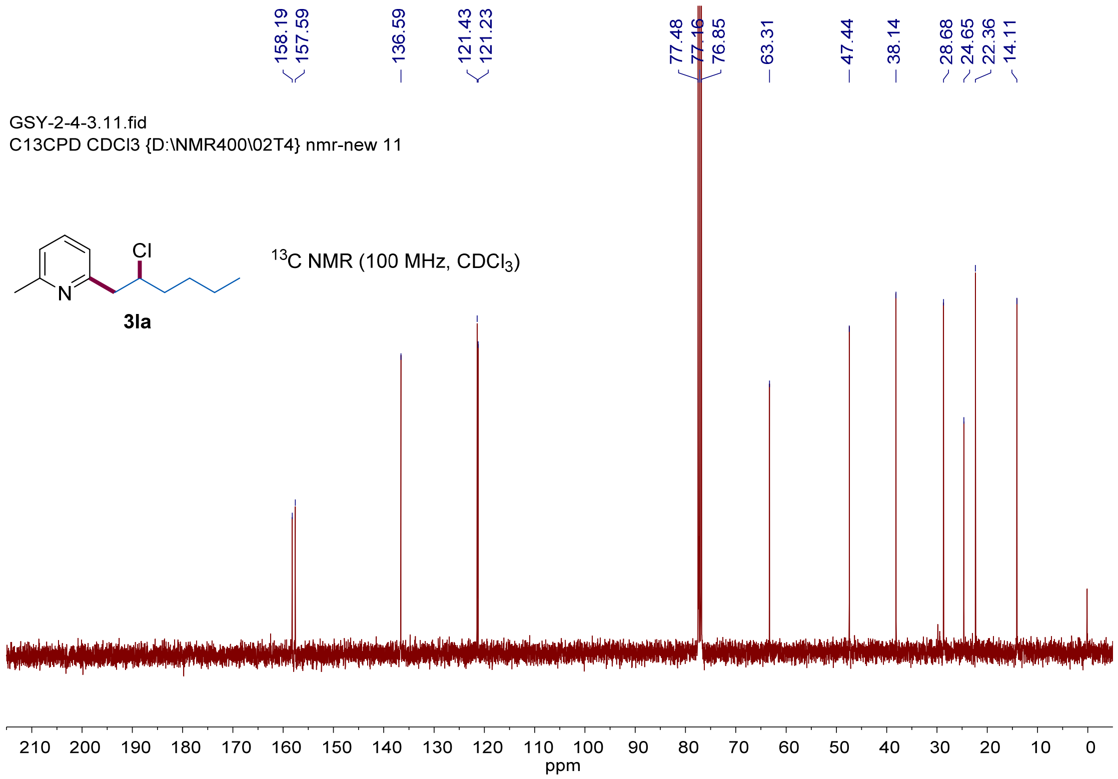

Supplementary Figure 79. ^{13}C NMR spectra of compound 3la

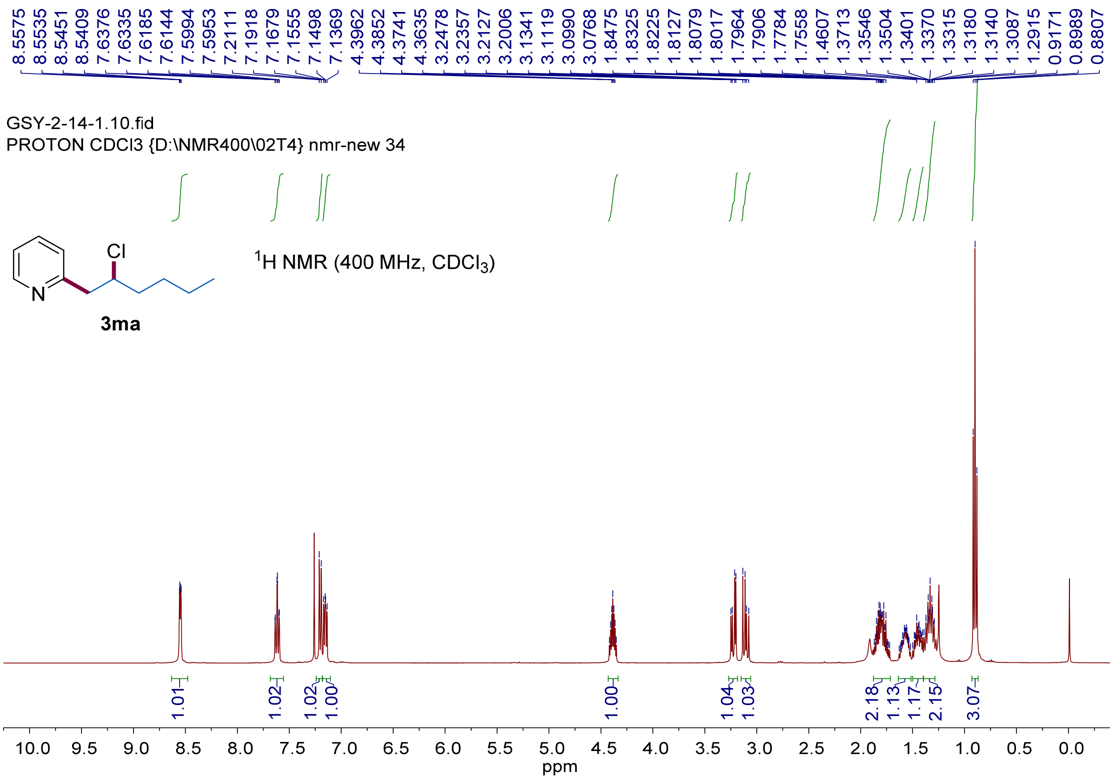

Supplementary Figure 80. ^{1}H NMR spectra of compound 3ma

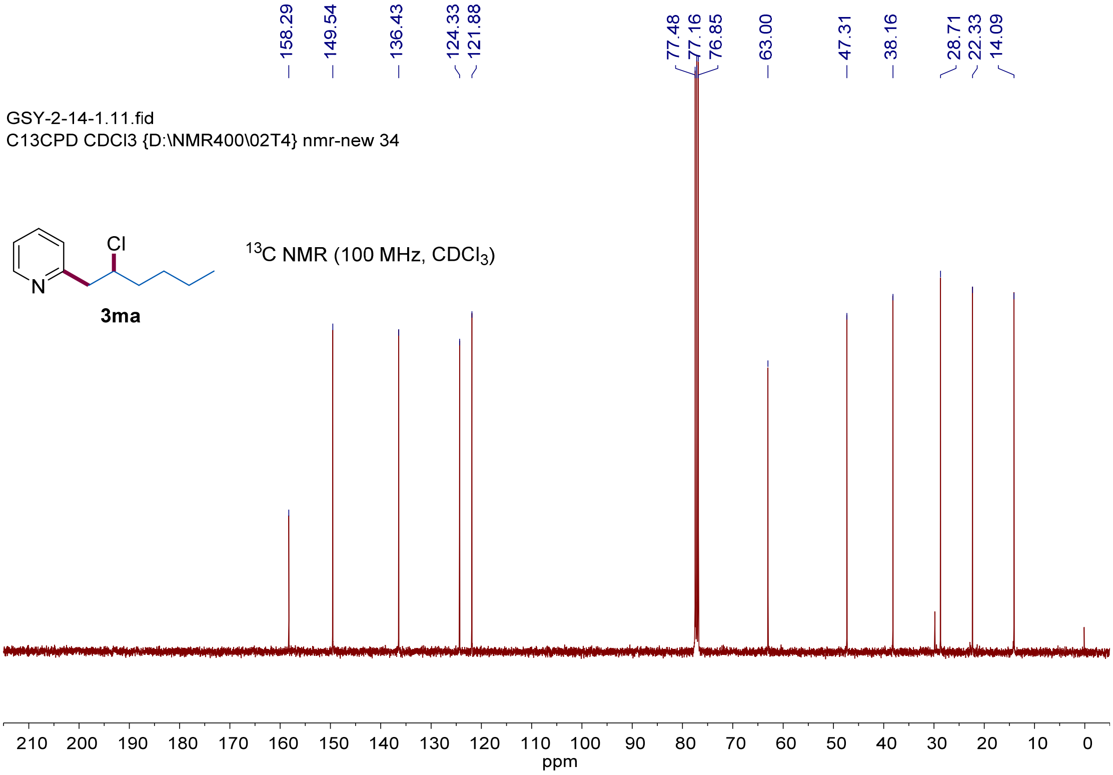

Supplementary Figure 81. ^{13}C NMR spectra of compound 3ma

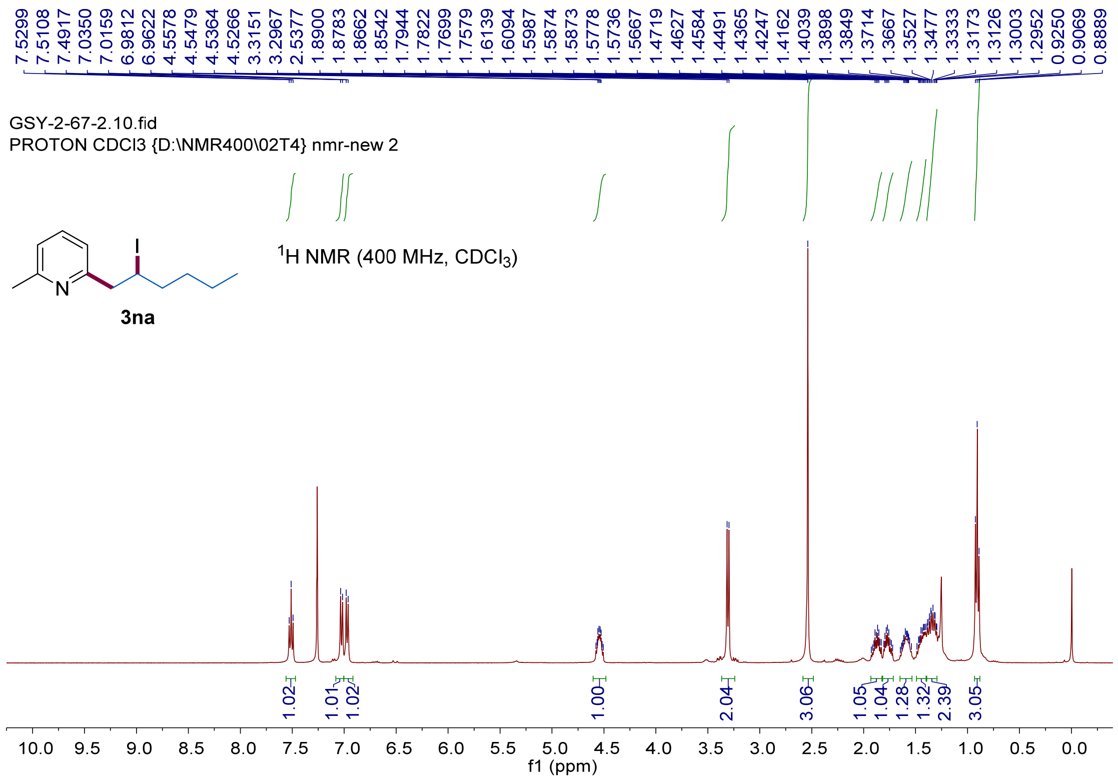

Supplementary Figure 82. ^{1}**H NMR spectra of compound 3na**

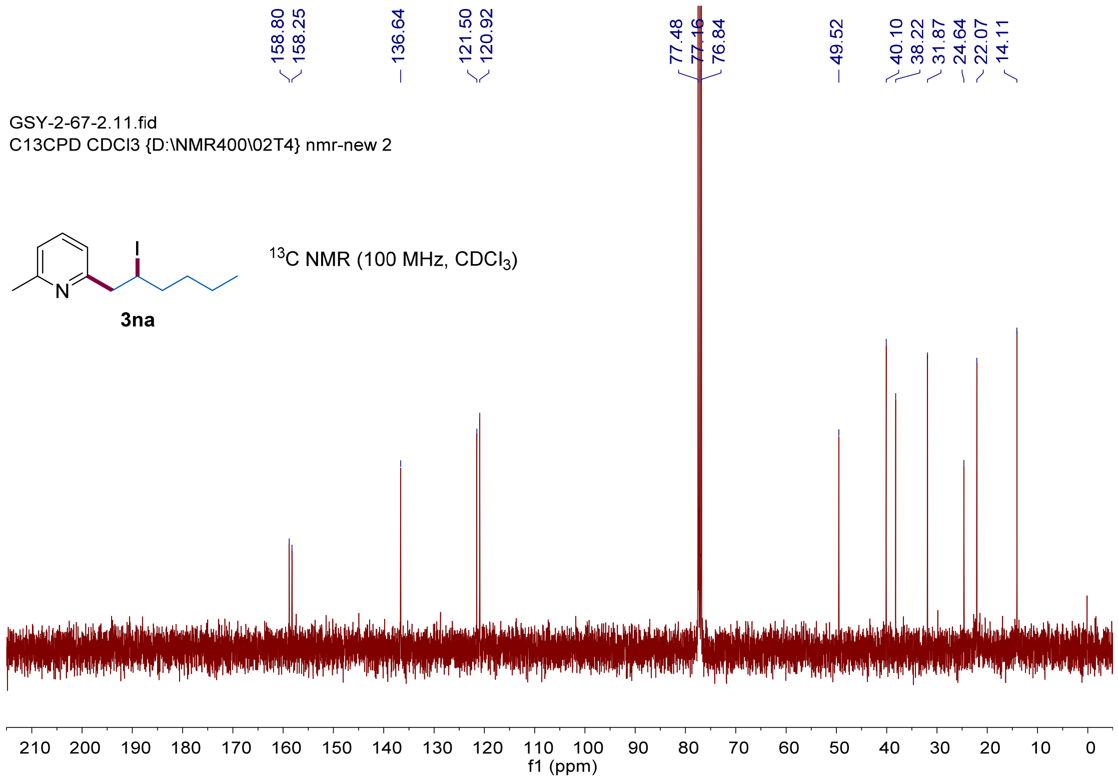

Supplementary Figure 83. ^{13}**C NMR spectra of compound 3na**

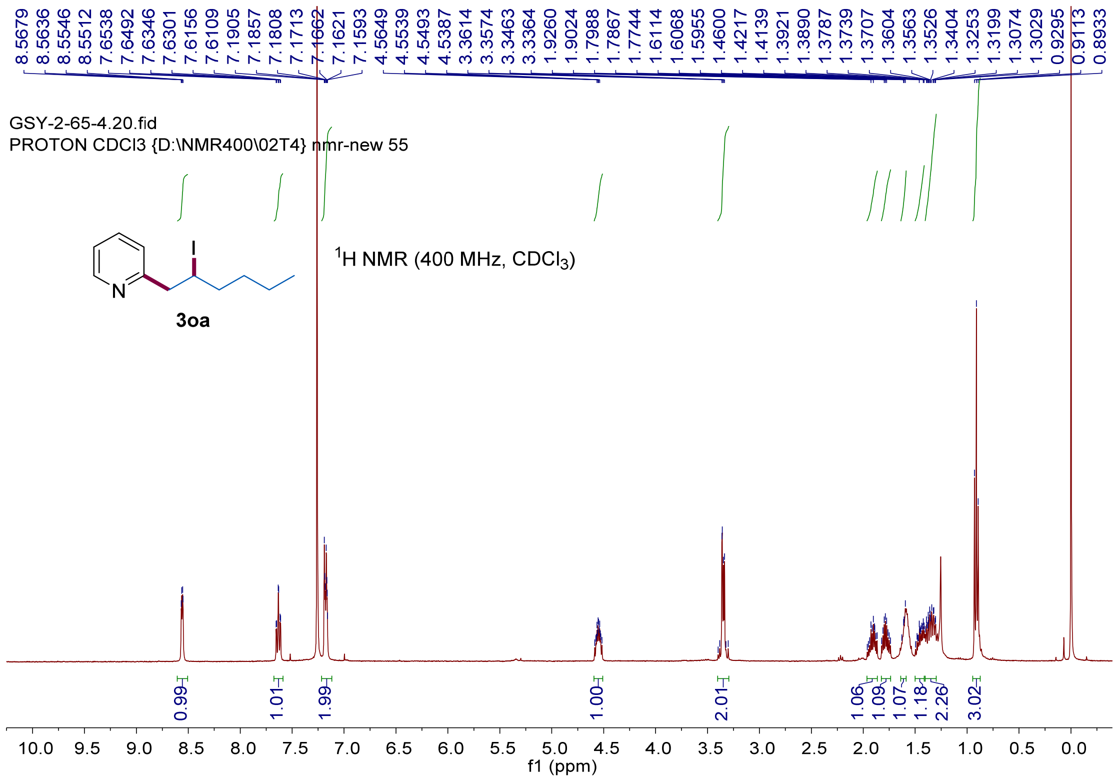

Supplementary Figure 84. ^{1}H NMR spectra of compound 3oa

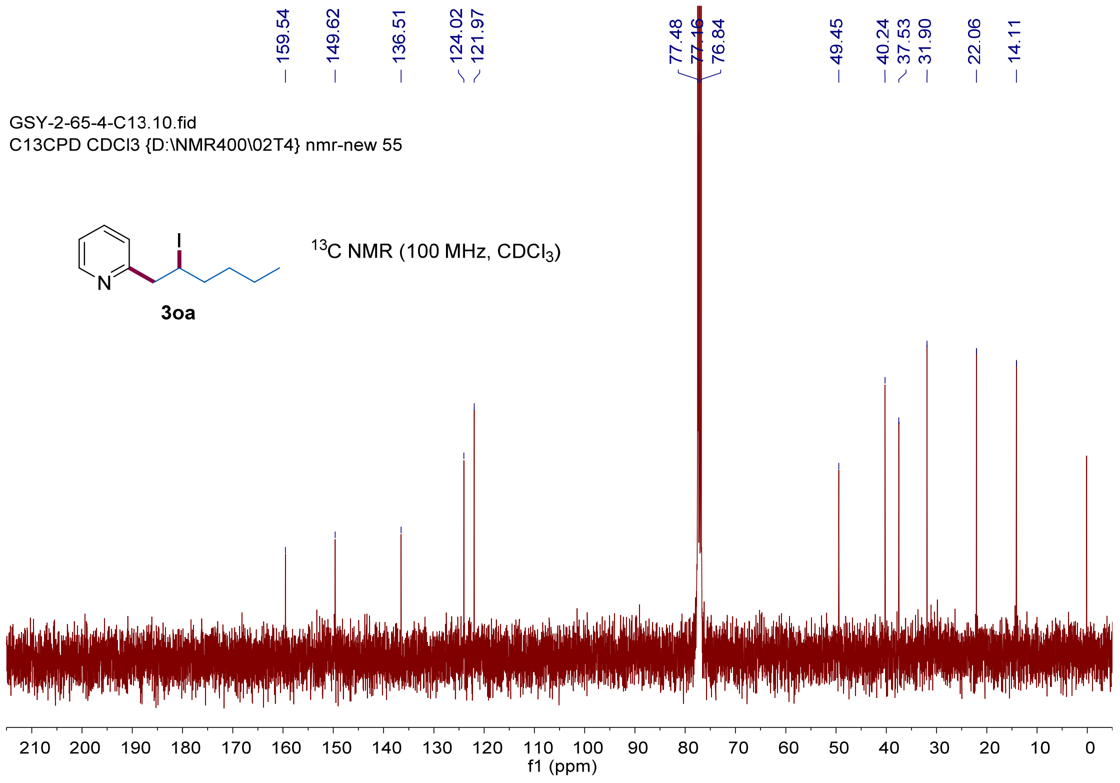

Supplementary Figure 85. ^{13}C NMR spectra of compound 3oa

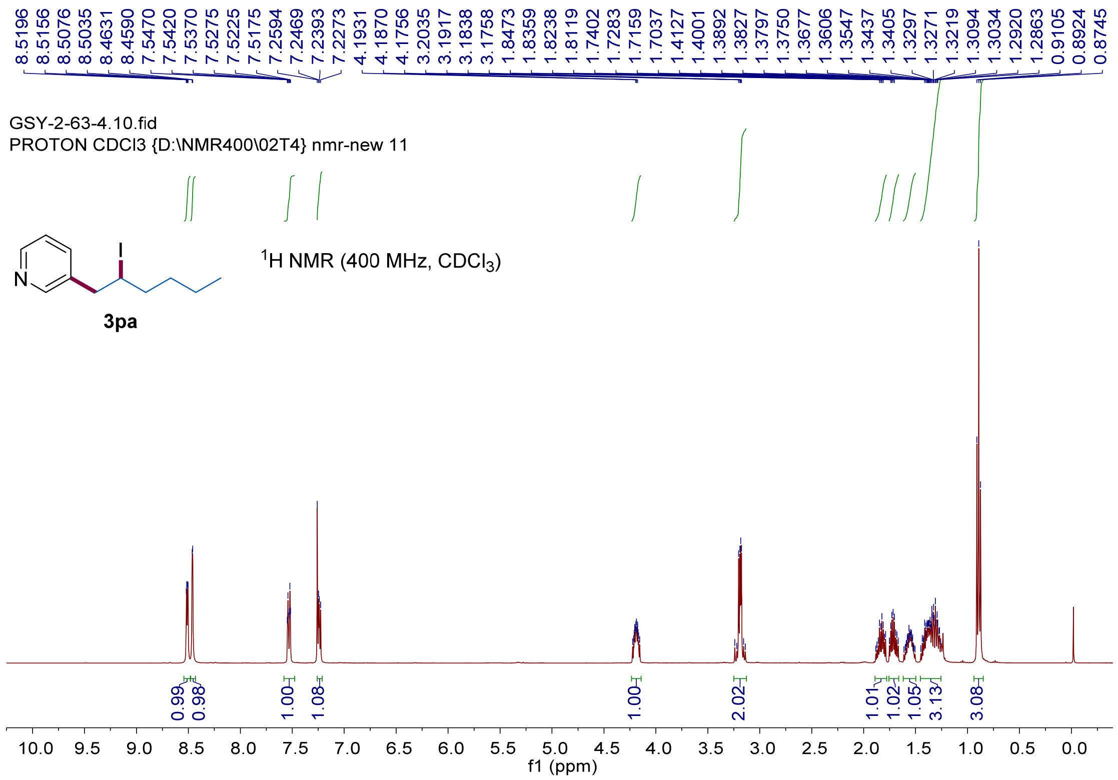

Supplementary Figure 86. ^{1}H NMR spectra of compound 3pa

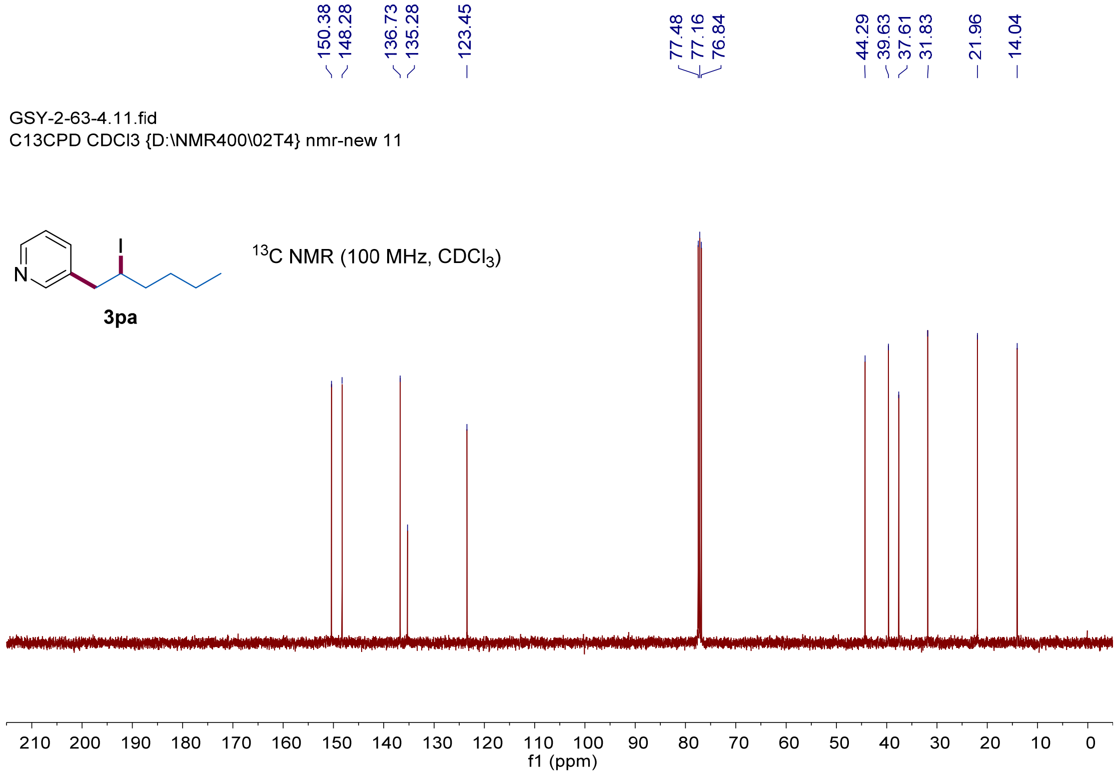

Supplementary Figure 87. ^{13}C NMR spectra of compound 3pa

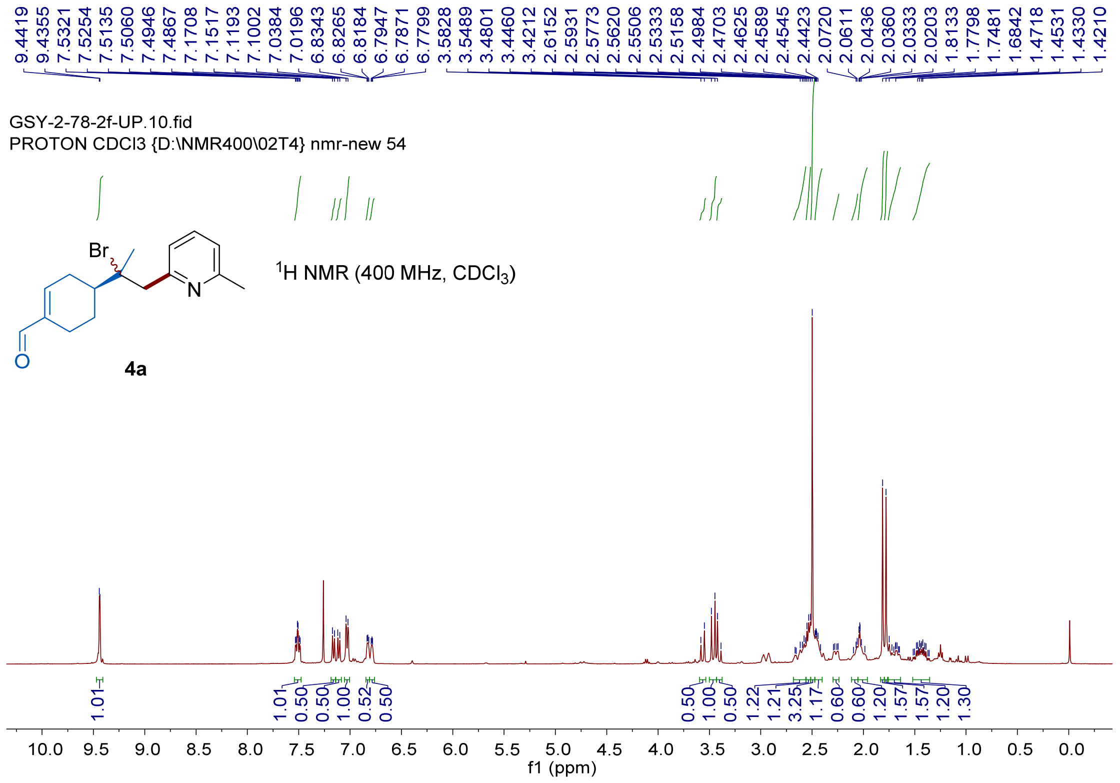

Supplementary Figure 88. ^{1}H NMR spectra of compound 4a

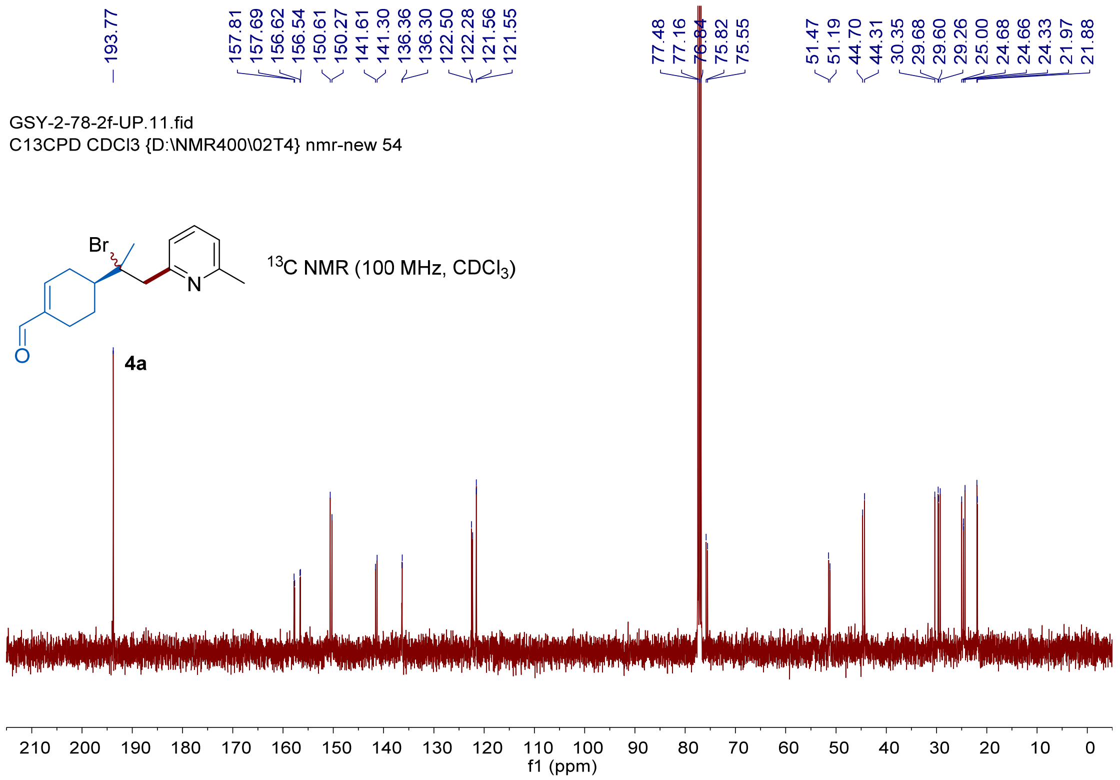

Supplementary Figure 89. ^{13}C NMR spectra of compound 4a

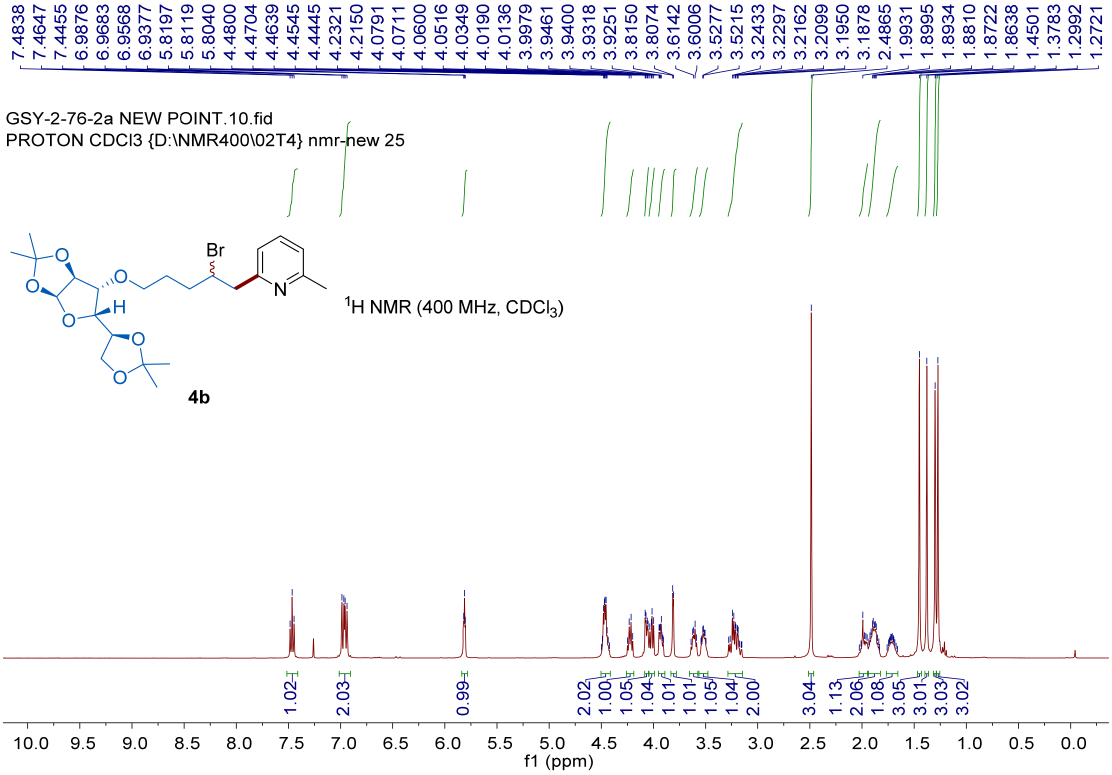

Supplementary Figure 90. ^{1}H NMR spectra of compound 4b

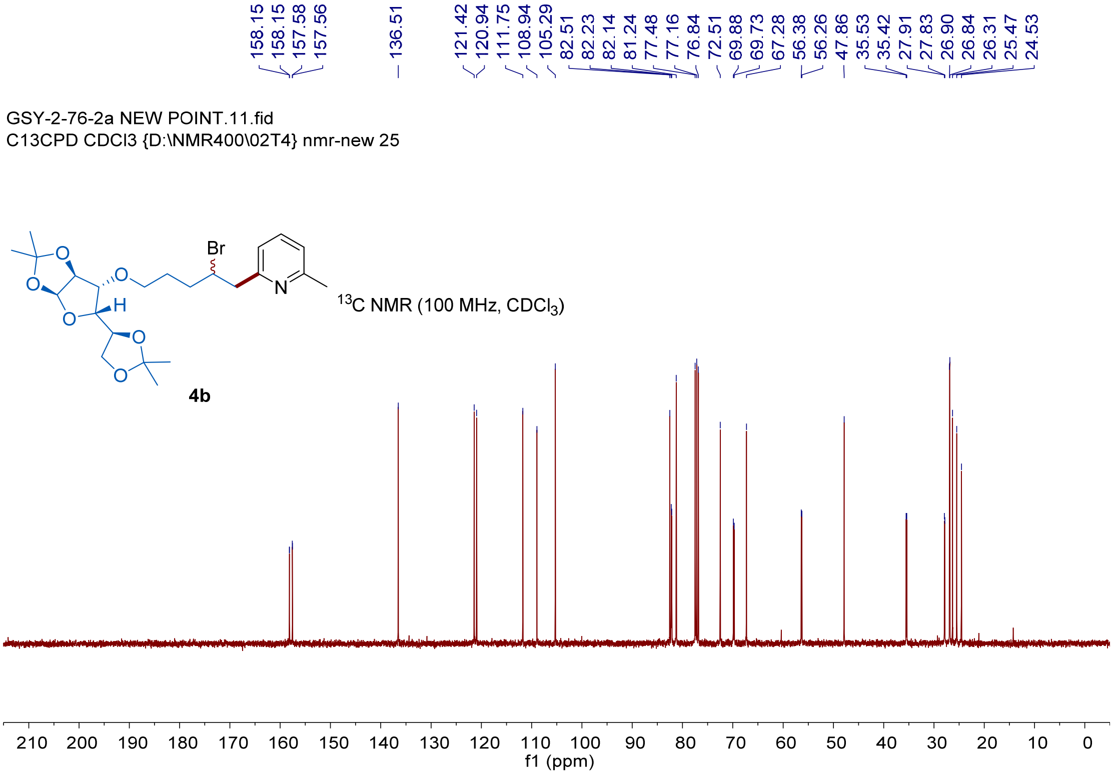

Supplementary Figure 91. ^{13}C NMR spectra of compound 4b

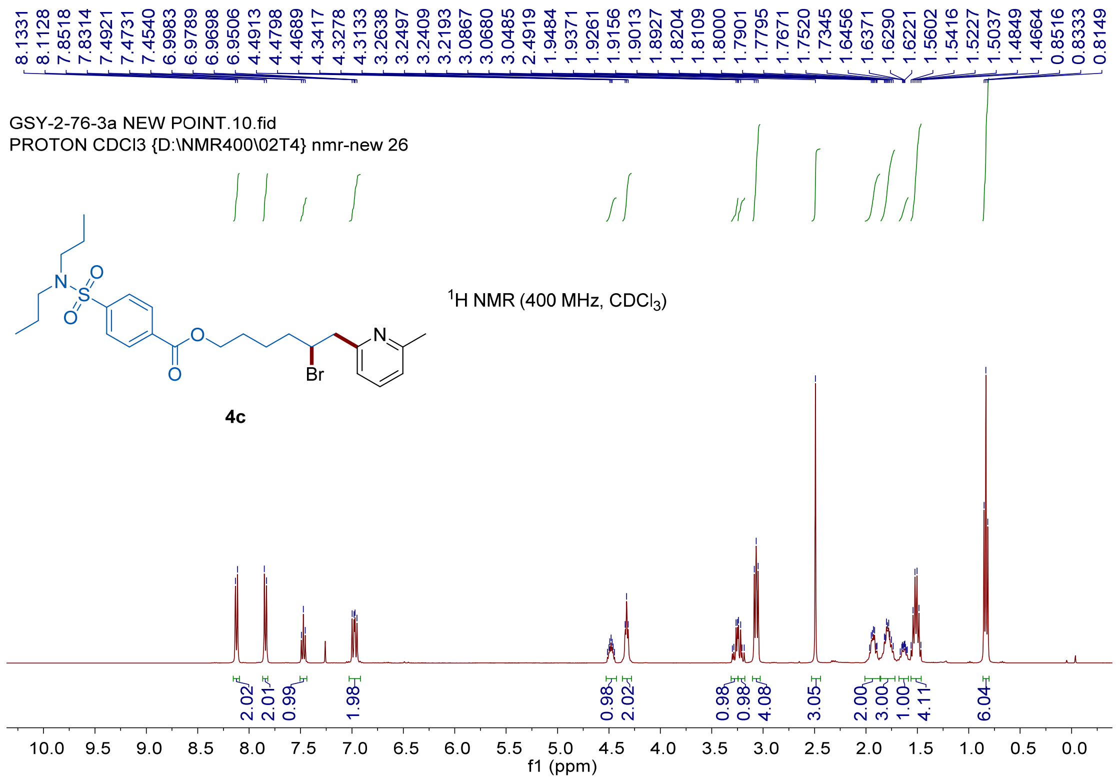

Supplementary Figure 92. ^{1}H NMR spectra of compound 4c

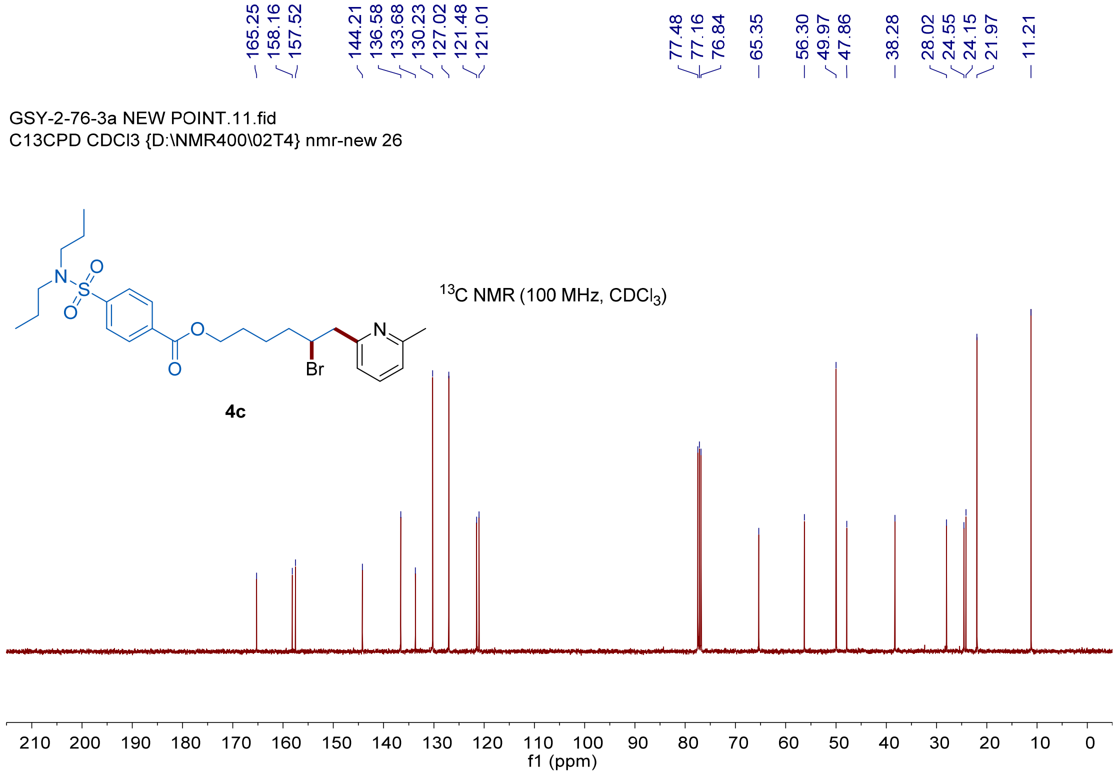

Supplementary Figure 93. ^{13}C NMR spectra of compound 4c

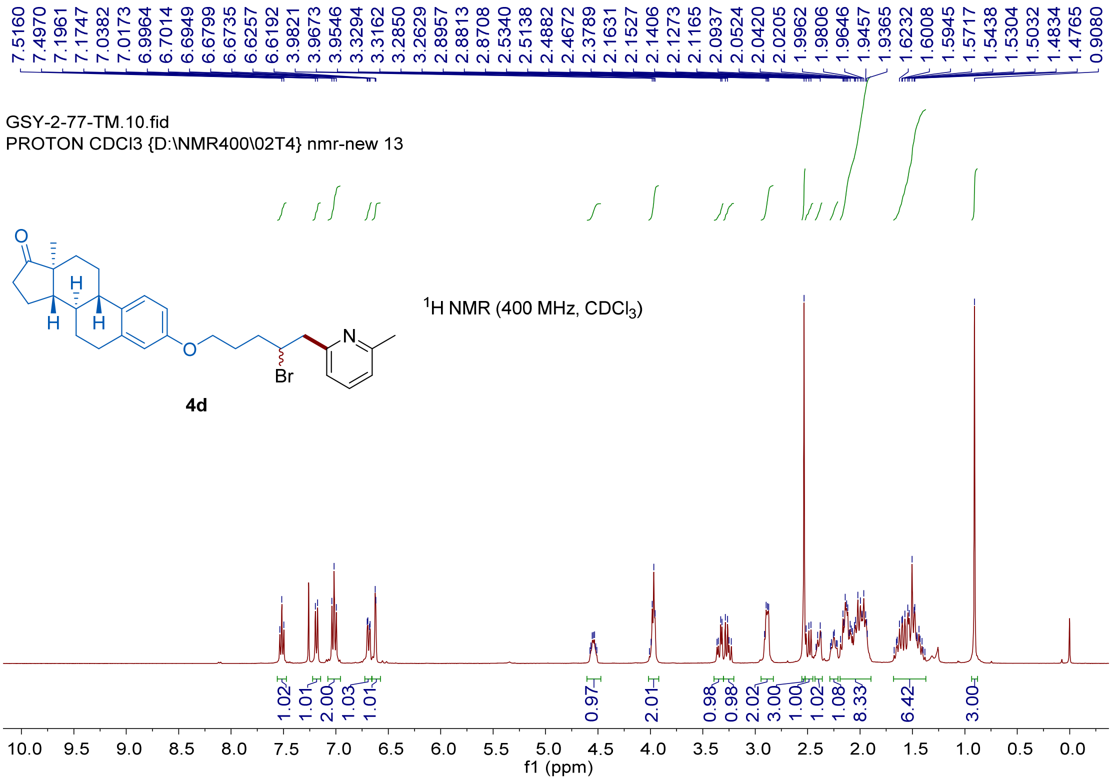

Supplementary Figure 94. ^{1}**H NMR spectra of compound 4d**

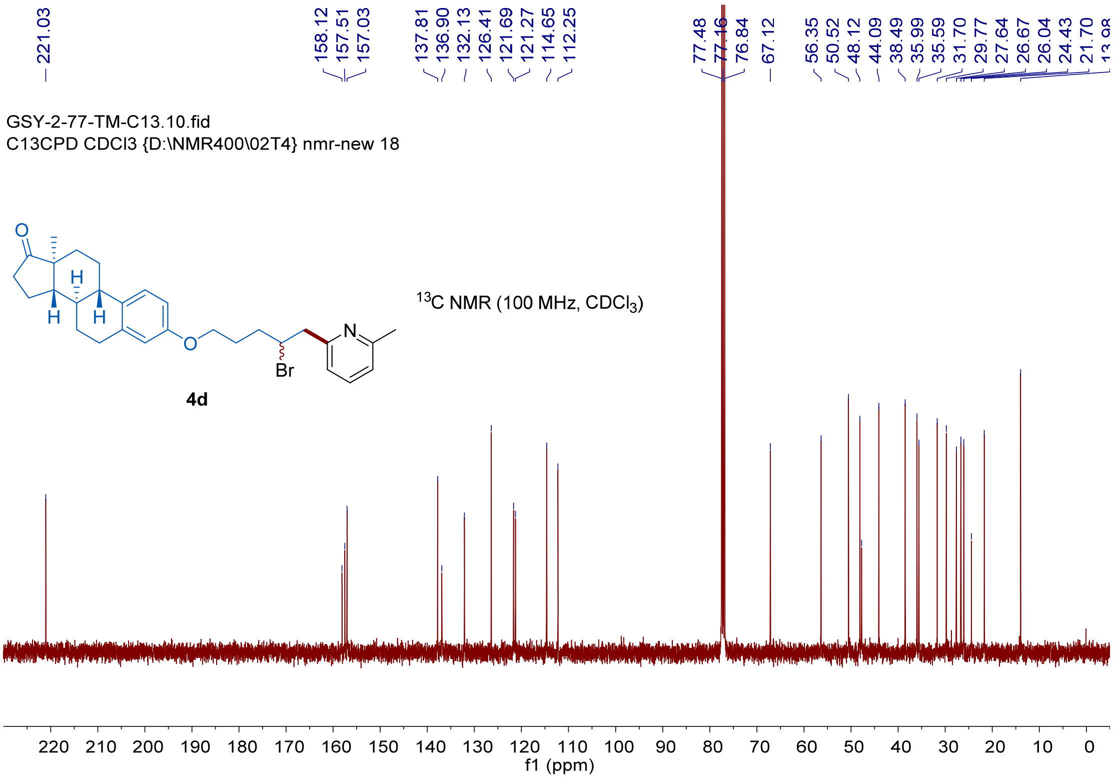

Supplementary Figure 95. ^{13}**C NMR spectra of compound 4d**

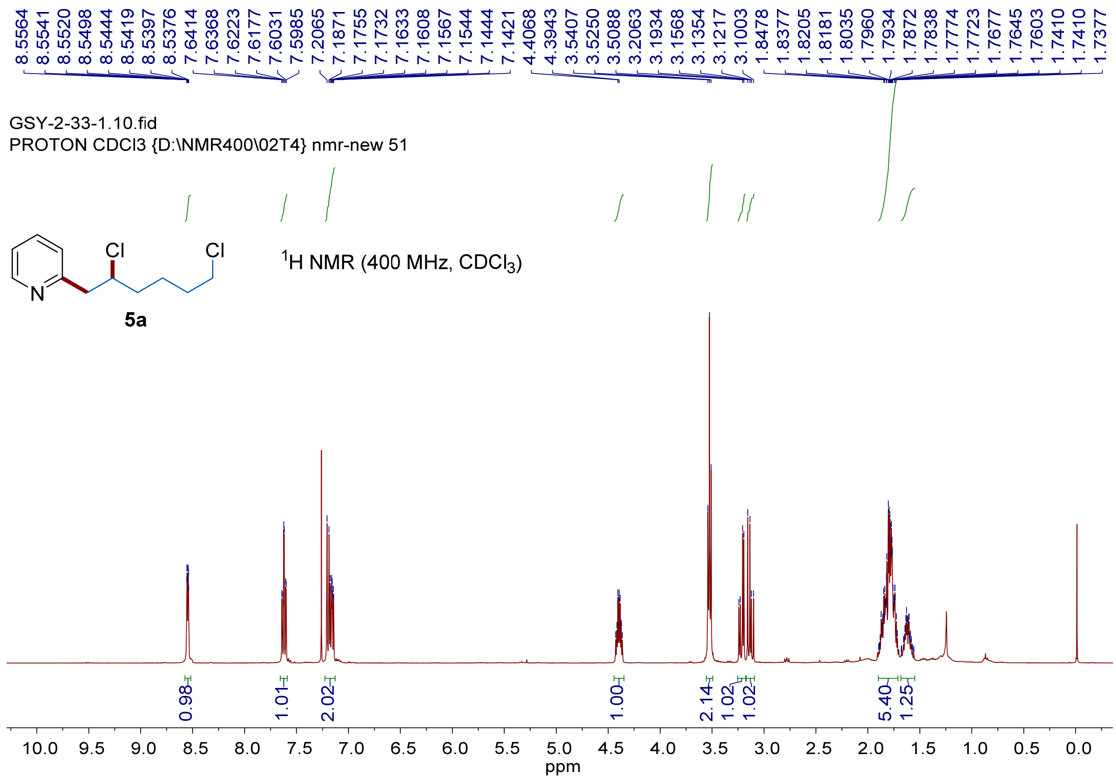

Supplementary Figure 96. ^{1}H NMR spectra of compound 5a

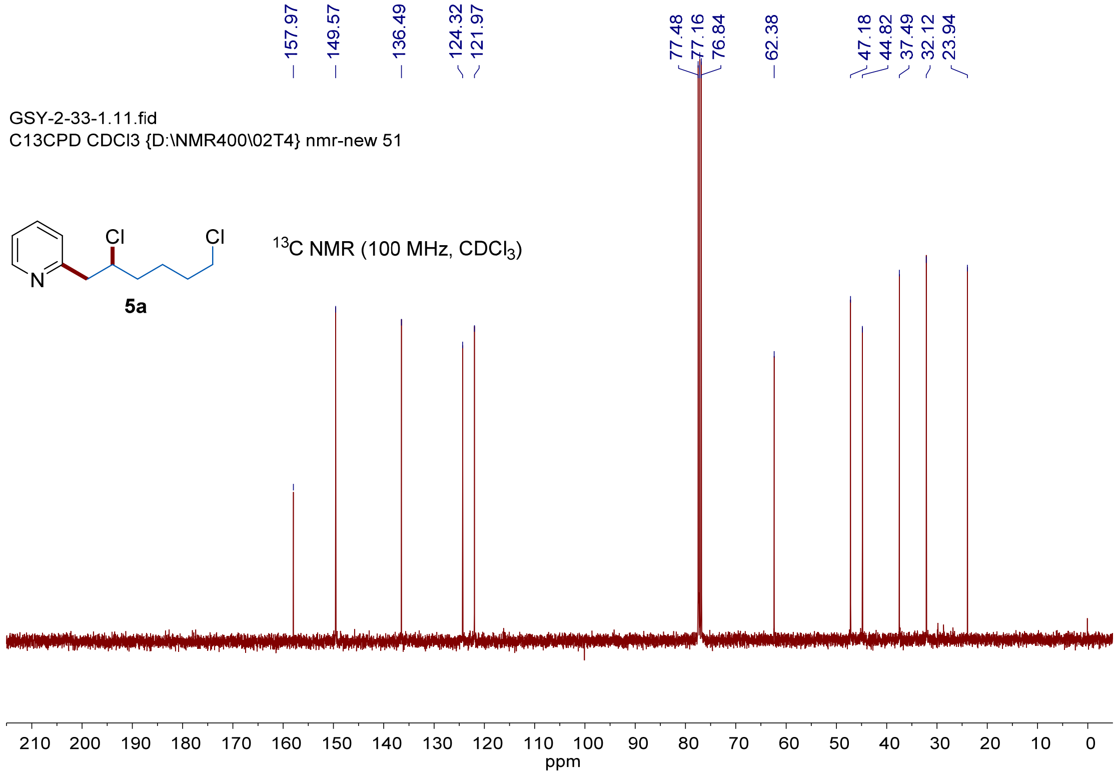

Supplementary Figure 97. ^{13}C NMR spectra of compound 5a

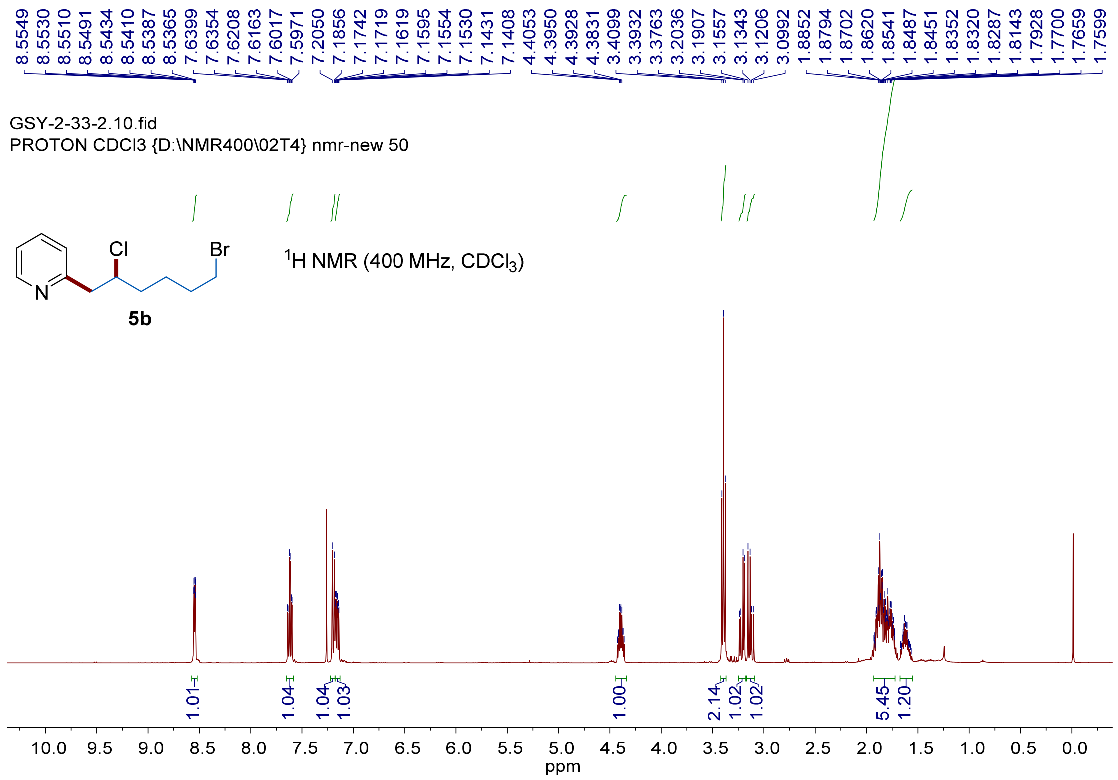

Supplementary Figure 98. ^{1}**H NMR spectra of compound 5b**

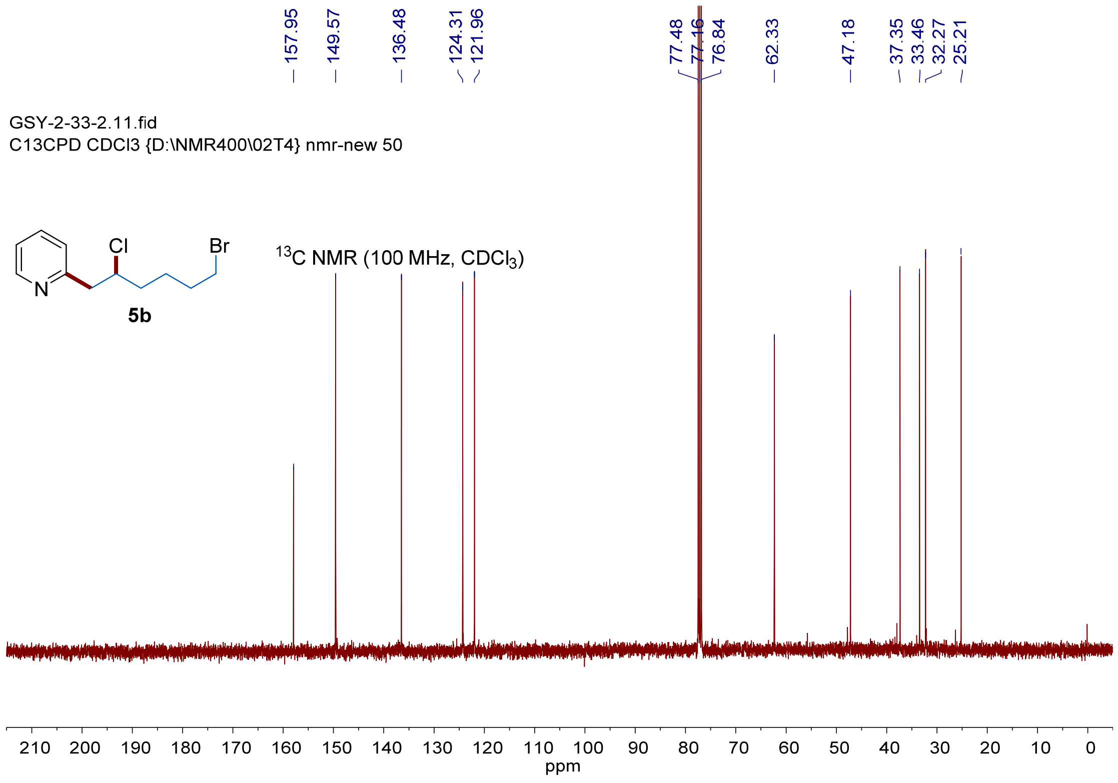

Supplementary Figure 99. ^{13}**C NMR spectra of compound 5b**

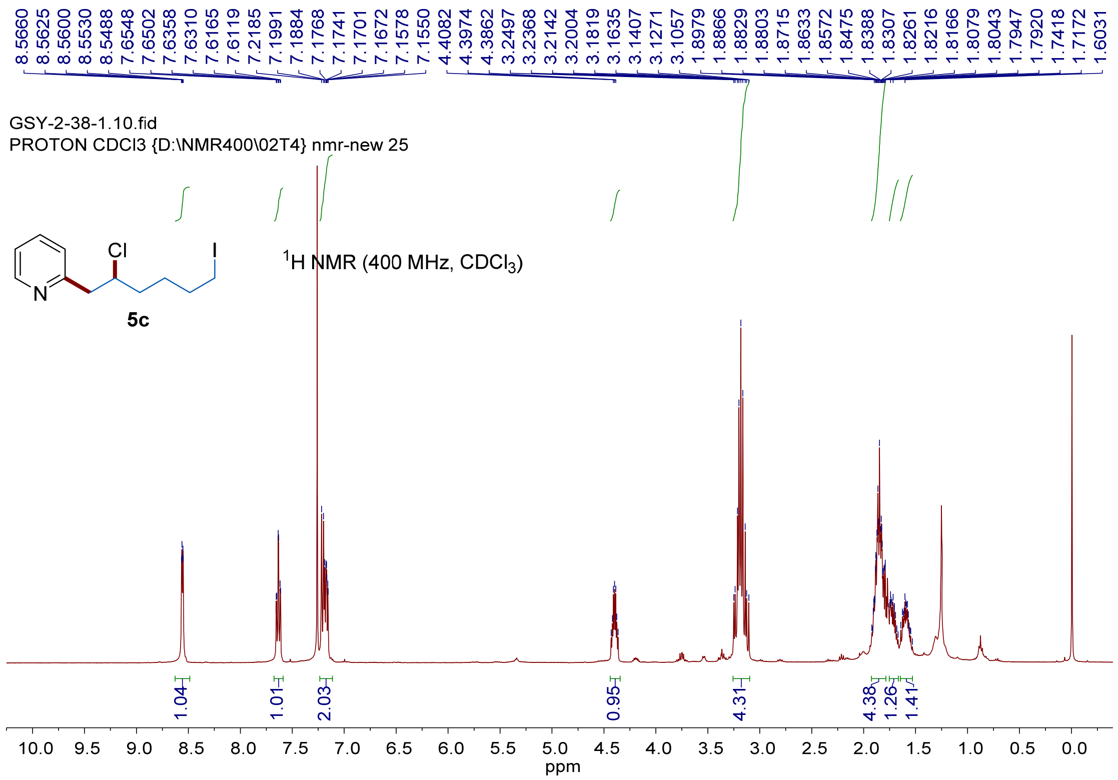

Supplementary Figure 100. ^{1}H NMR spectra of compound 5c

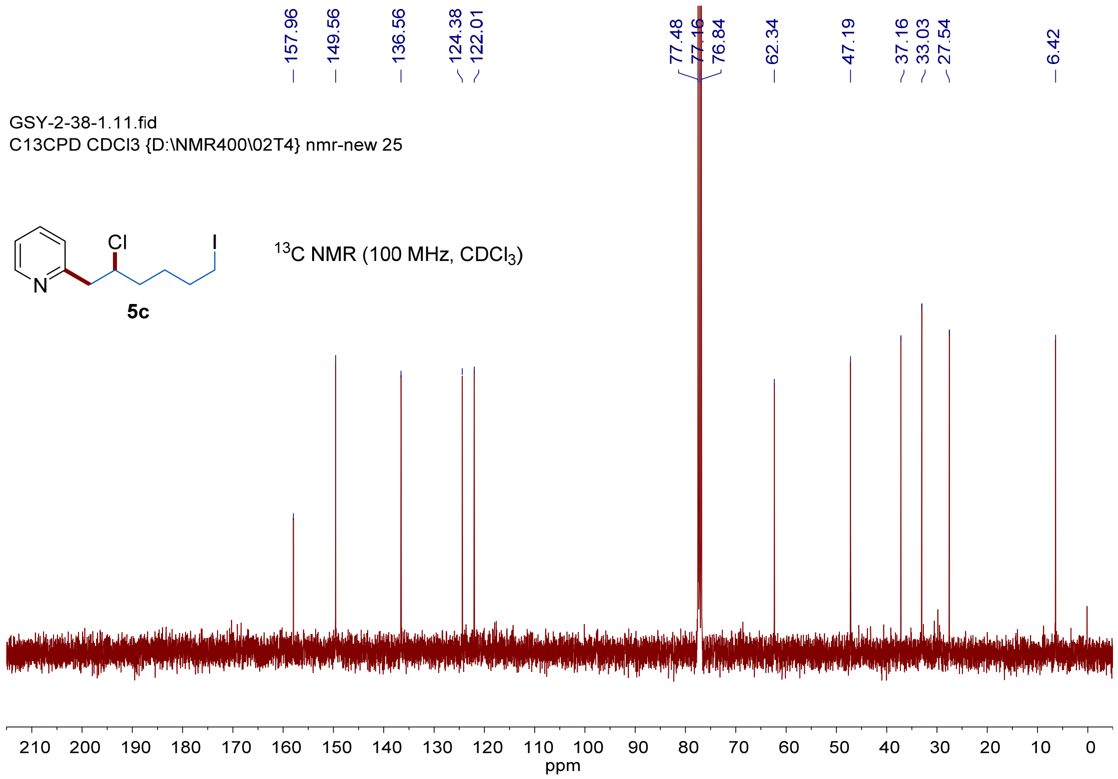

Supplementary Figure 101. ^{13}C NMR spectra of compound 5c

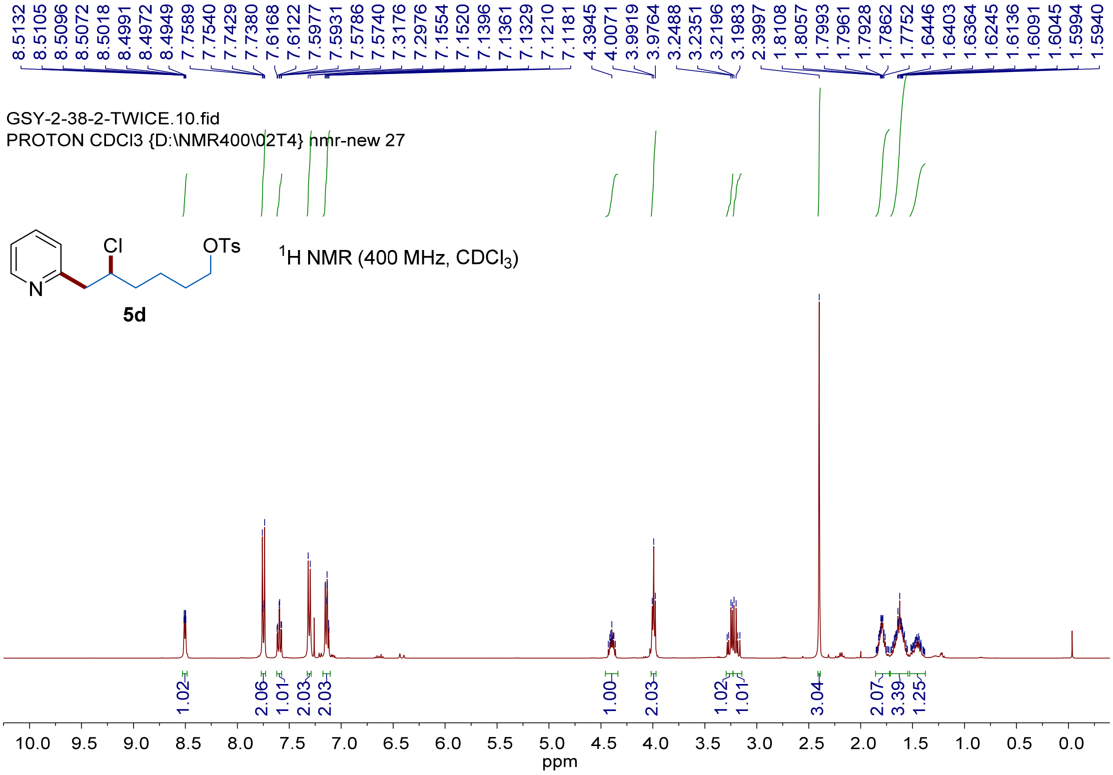

Supplementary Figure 102. ^{1}**H NMR spectra of compound 5d**

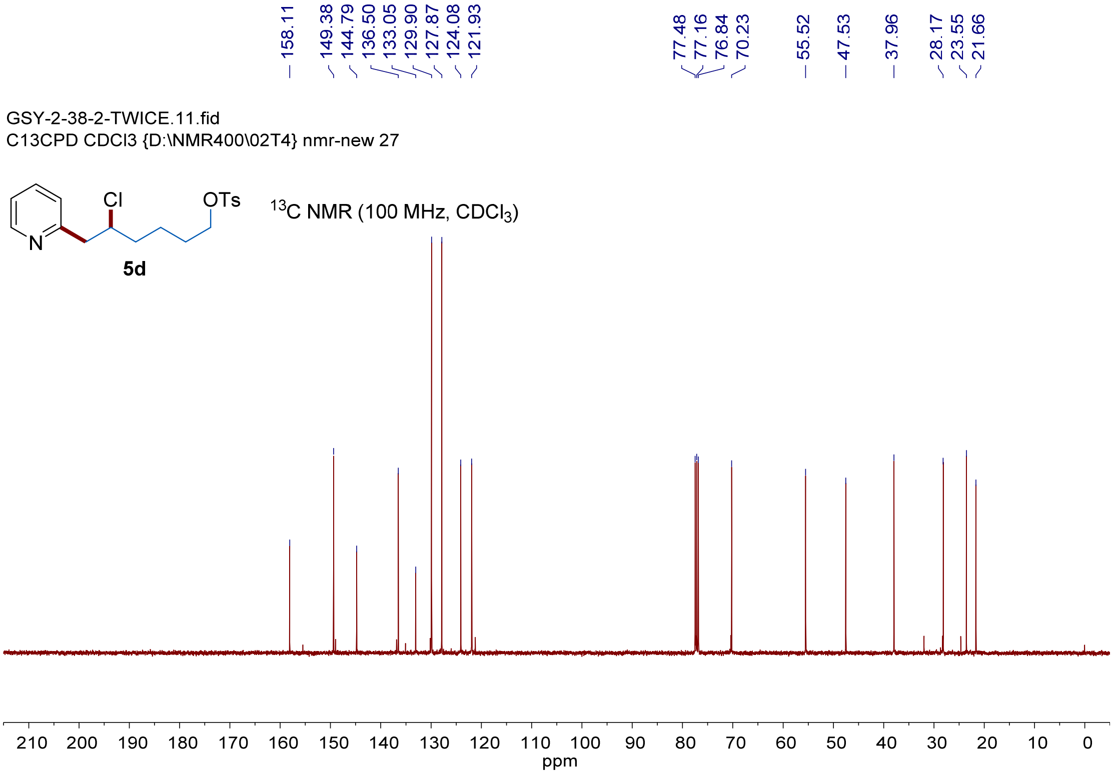

Supplementary Figure 103. ^{13}**C NMR spectra of compound 5d**

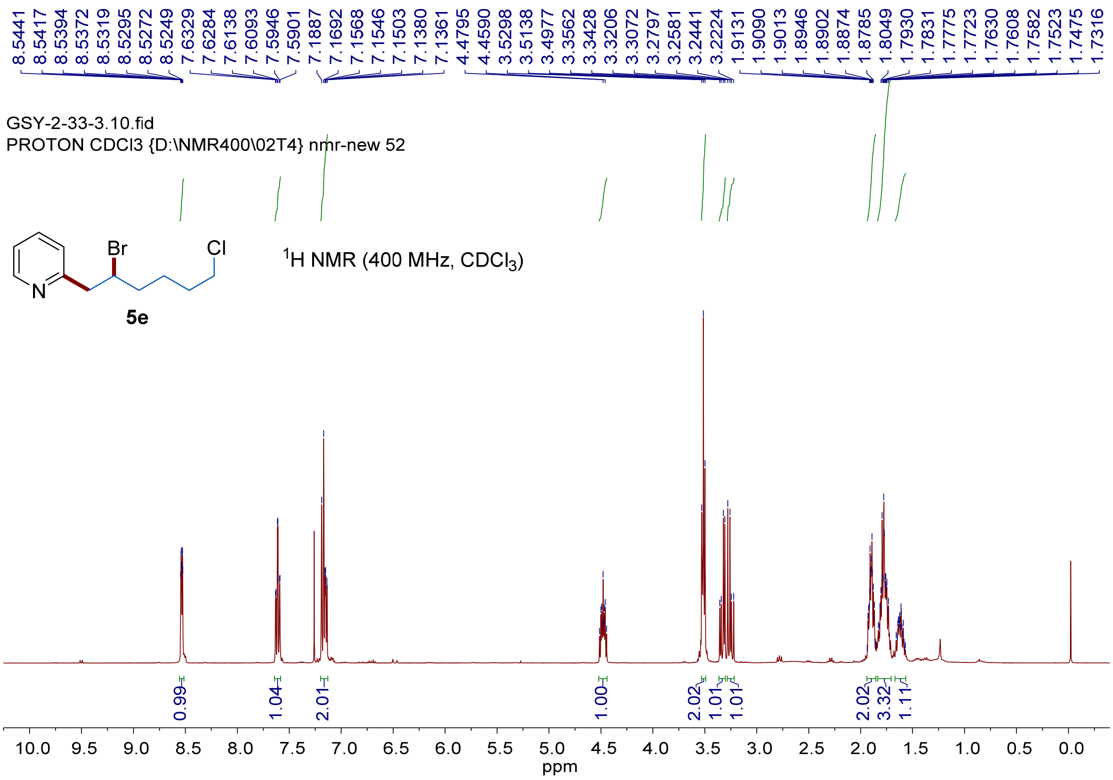

Supplementary Figure 104. ^{1}H NMR spectra of compound 5e

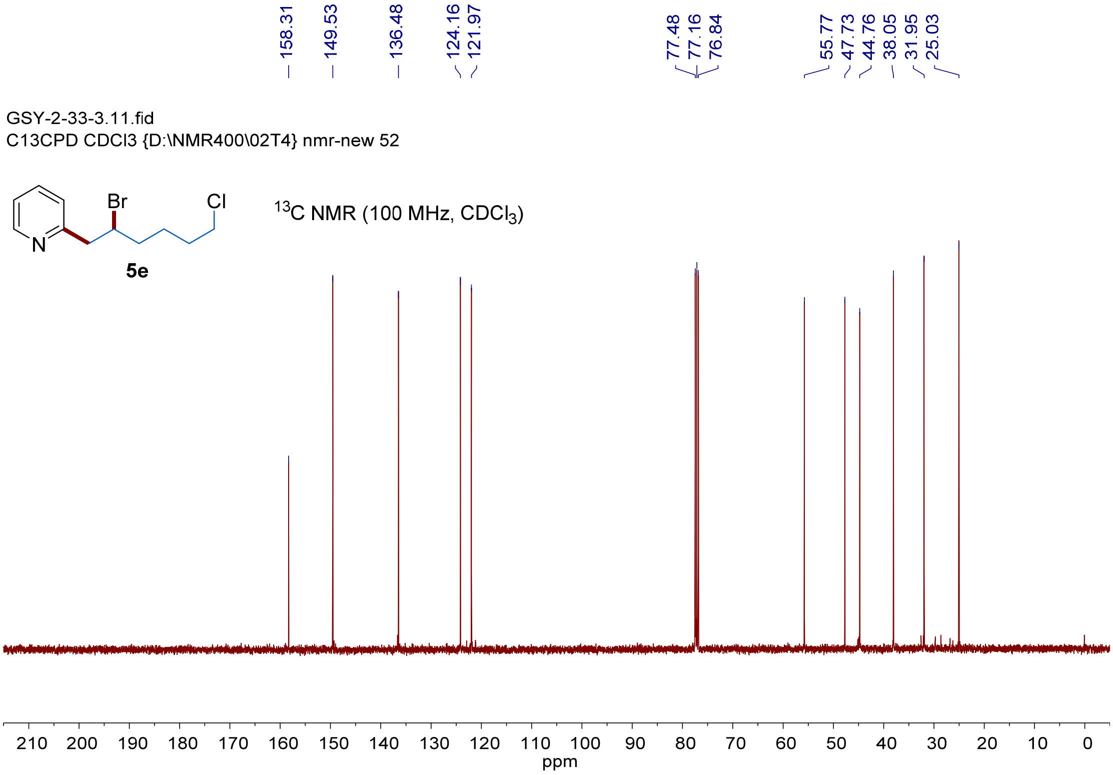

Supplementary Figure 105. ^{13}C NMR spectra of compound 5e

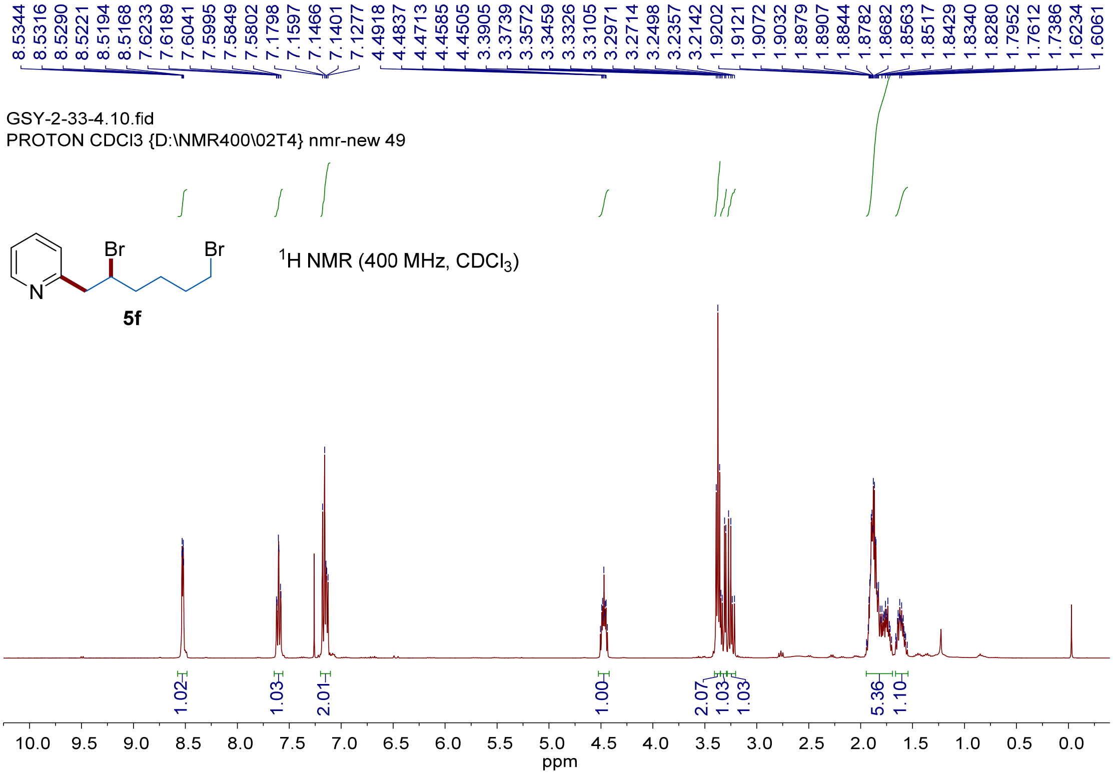

Supplementary Figure 106. ^{1}H NMR spectra of compound 5f

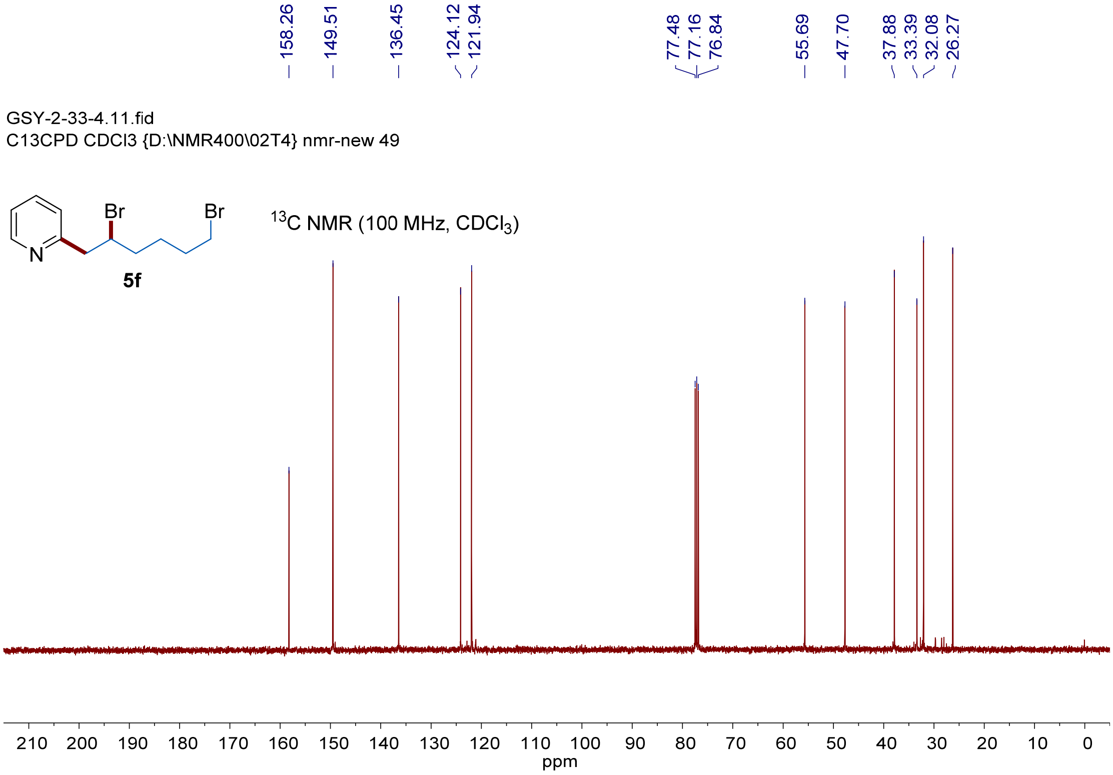

Supplementary Figure 107. ^{13}C NMR spectra of compound 5f

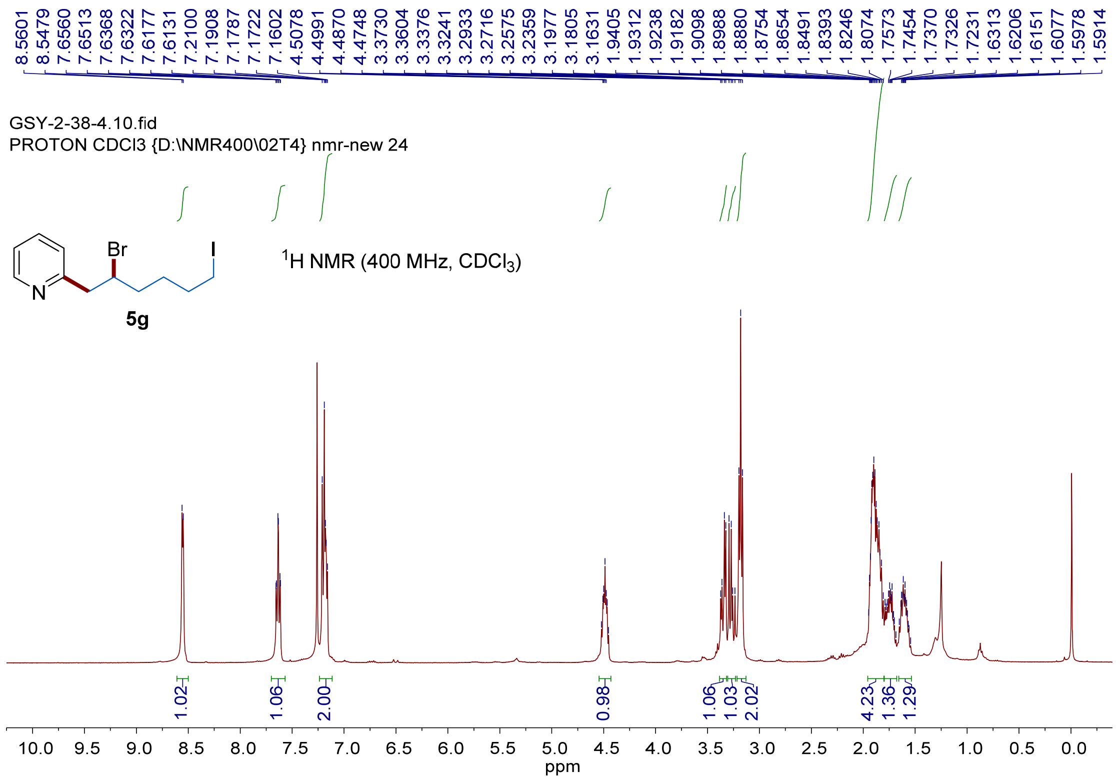

Supplementary Figure 108. ^{1}H NMR spectra of compound 5g

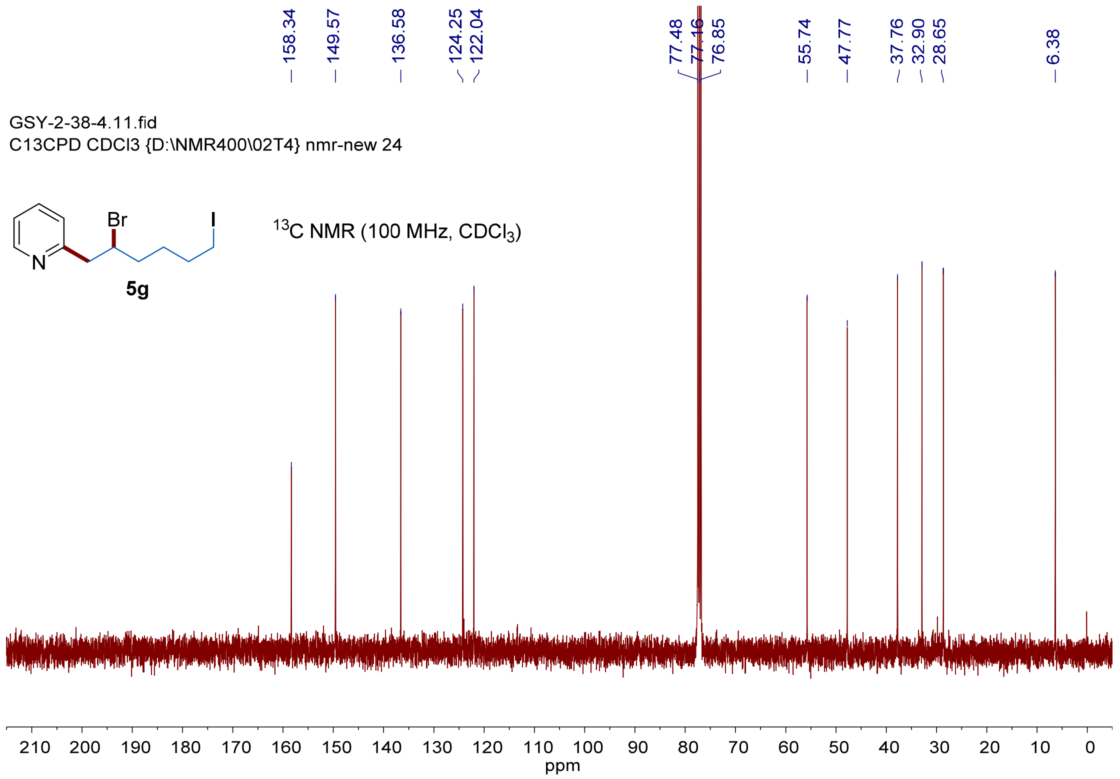

Supplementary Figure 109. ^{13}C NMR spectra of compound 5g

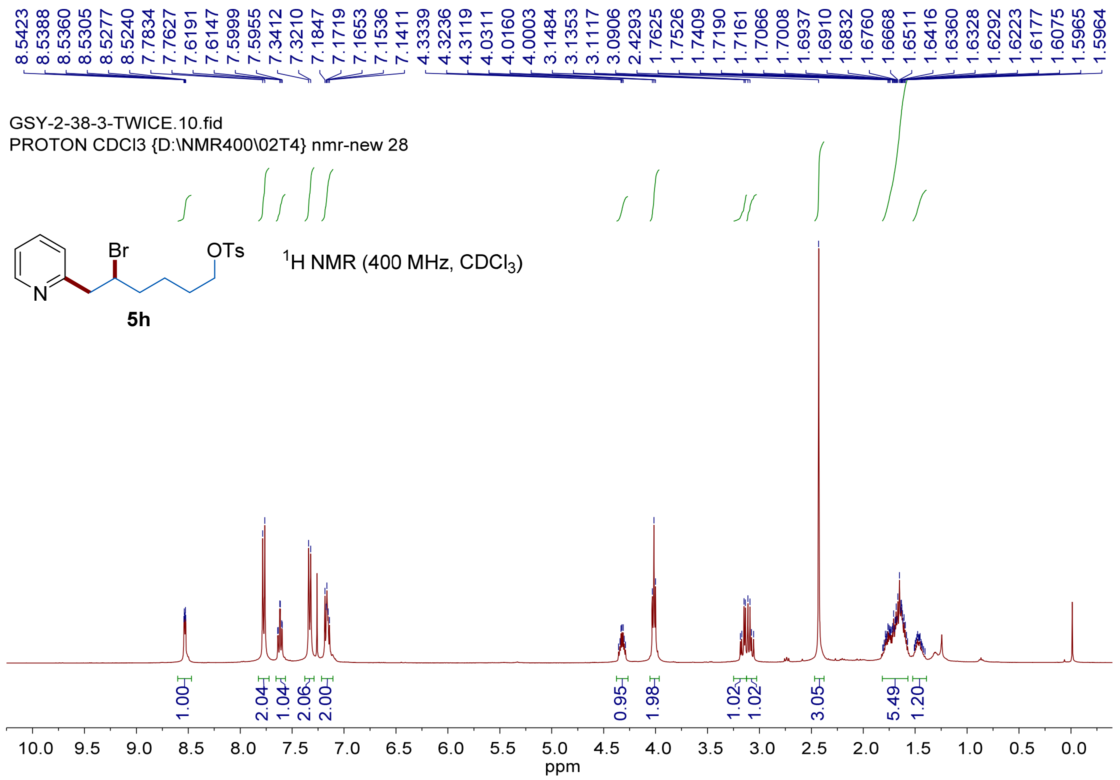

Supplementary Figure 110. ^{1}H NMR spectra of compound 5h

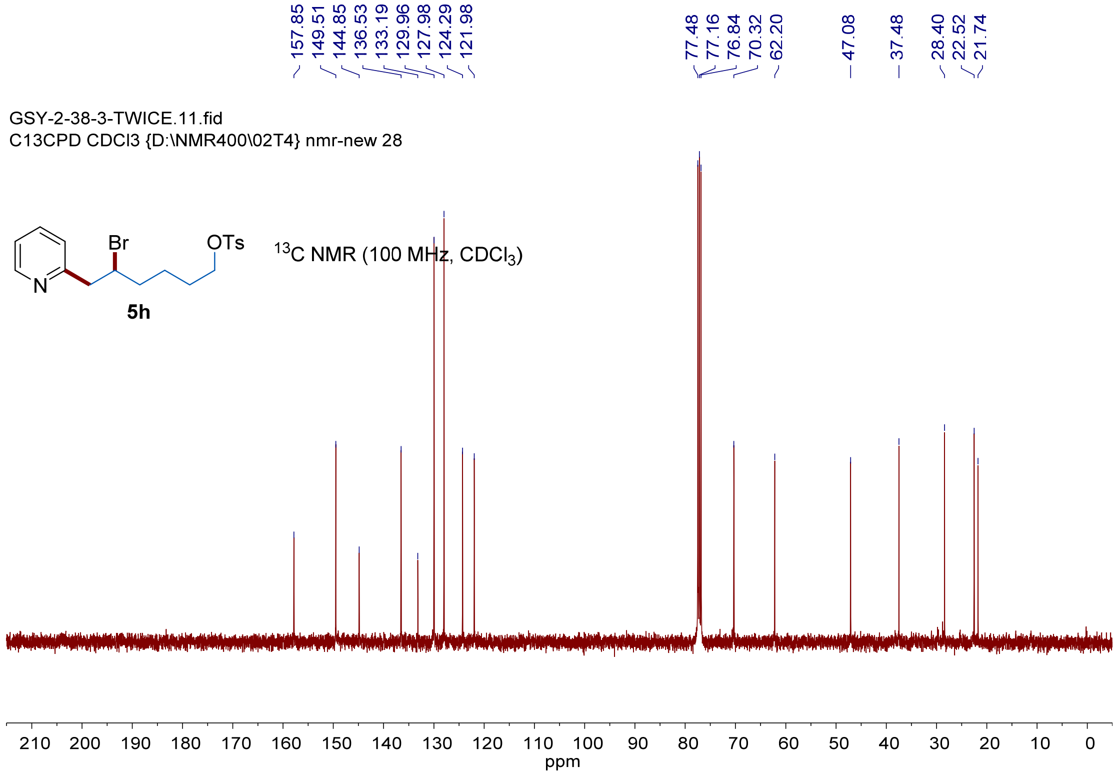

Supplementary Figure 111. ^{13}C NMR spectra of compound 5h

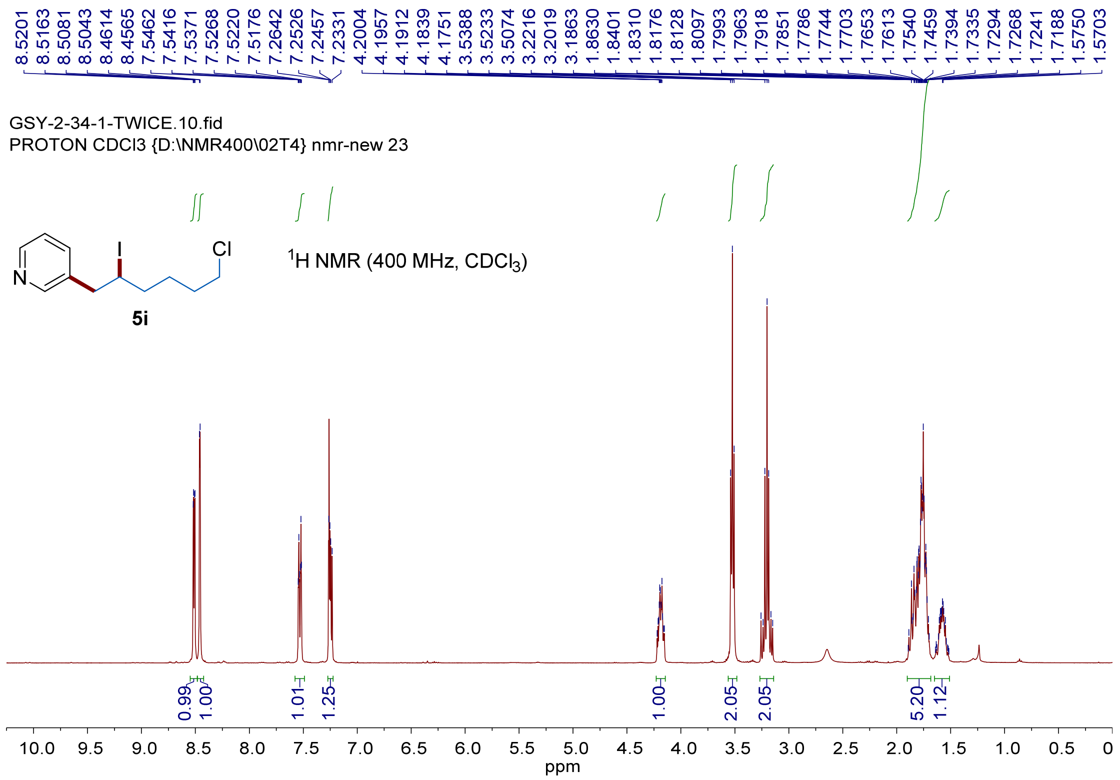

Supplementary Figure 112. ^{1}**H NMR spectra of compound 5i**

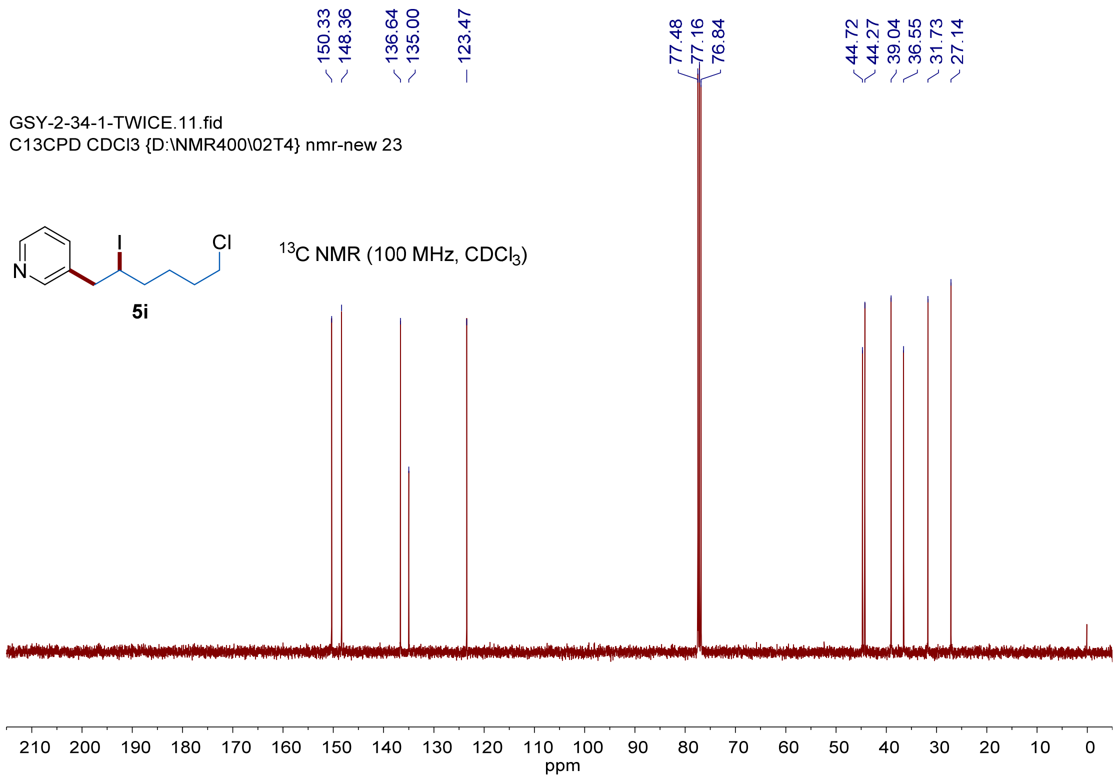

Supplementary Figure 113. ^{13}**C NMR spectra of compound 5i**

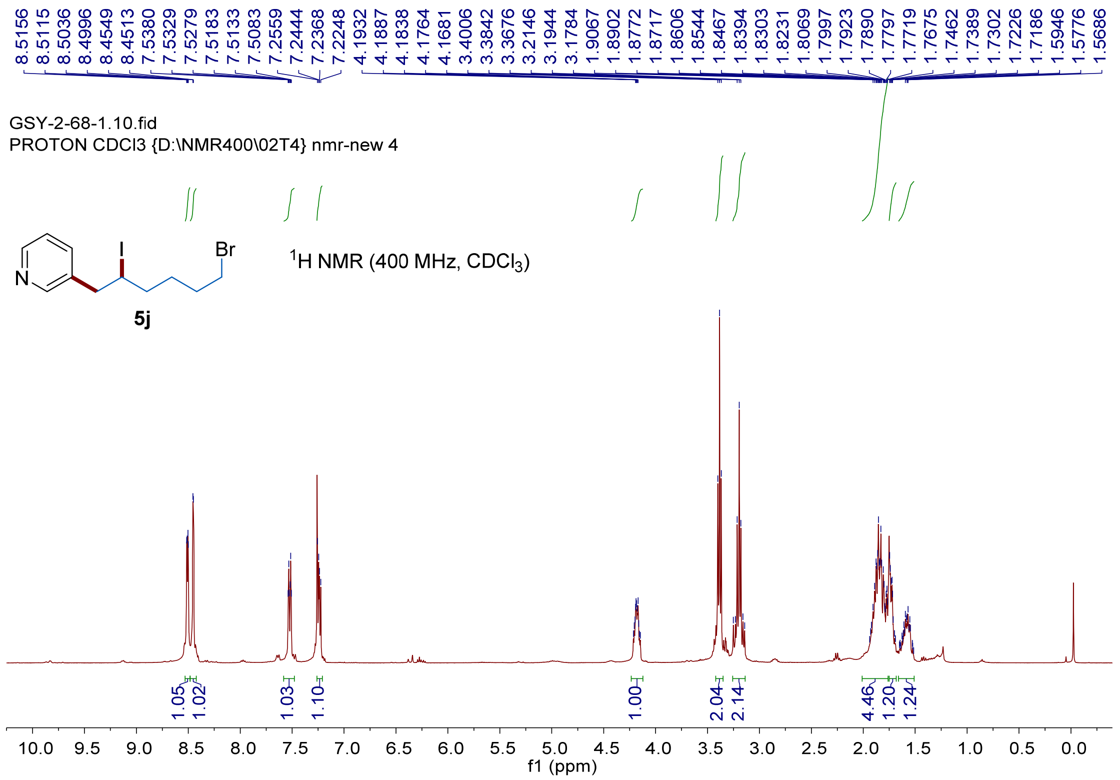

Supplementary Figure 114. ^{1}H NMR spectra of compound 5j

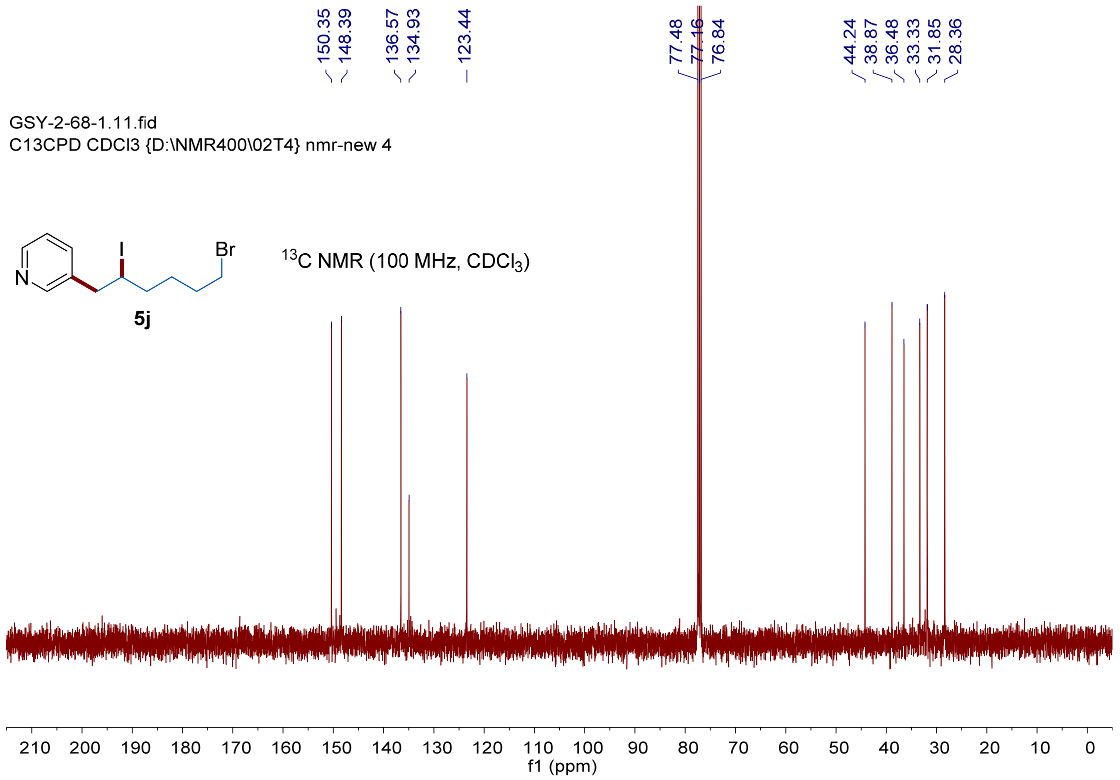

Supplementary Figure 115. ^{13}C NMR spectra of compound 5j

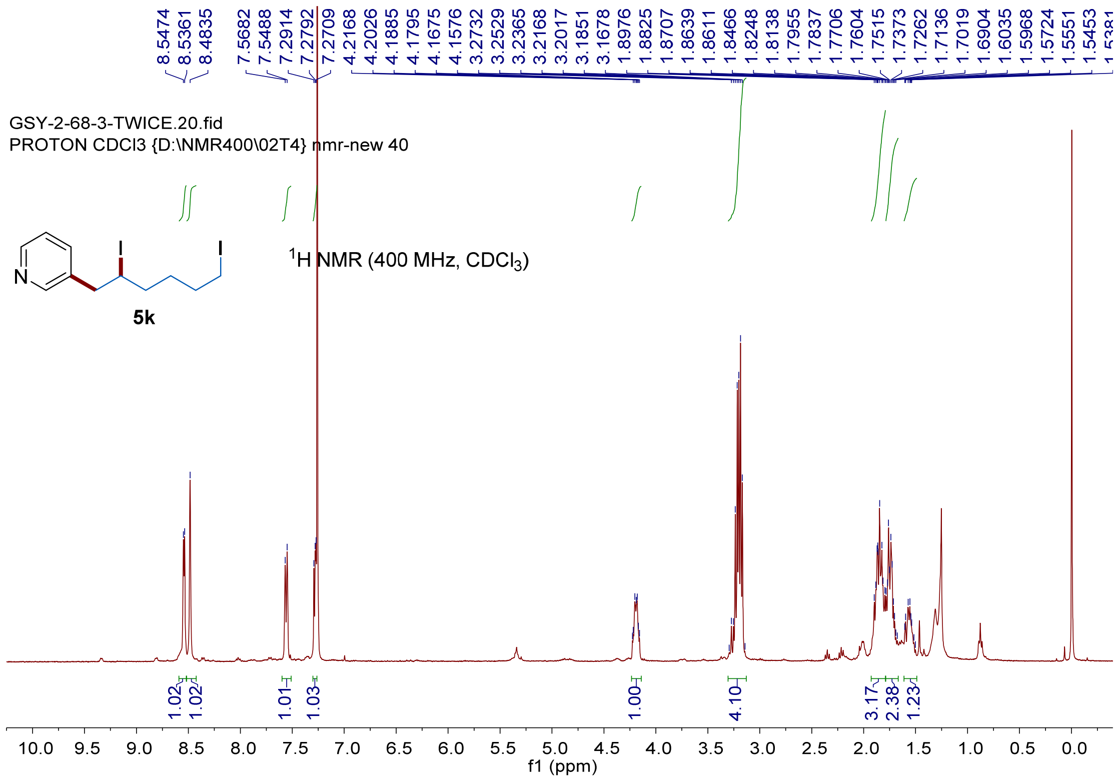

Supplementary Figure 116. ^{1}H NMR spectra of compound 5k

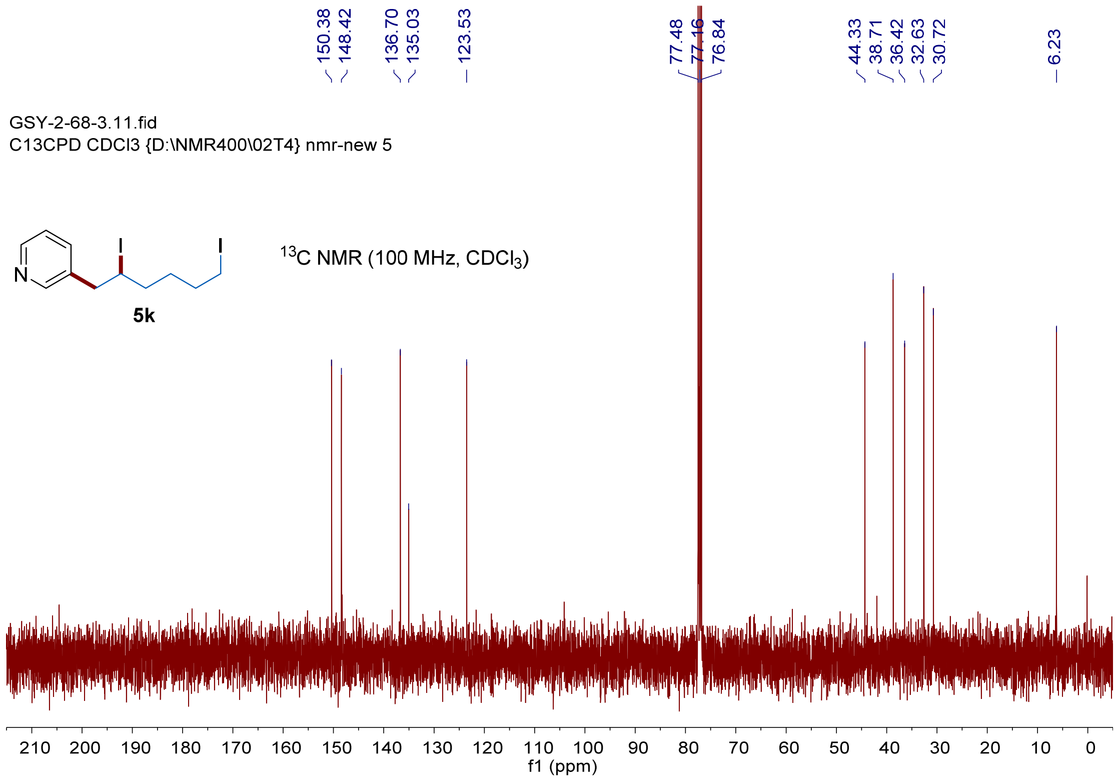

Supplementary Figure 117. ^{13}C NMR spectra of compound 5k

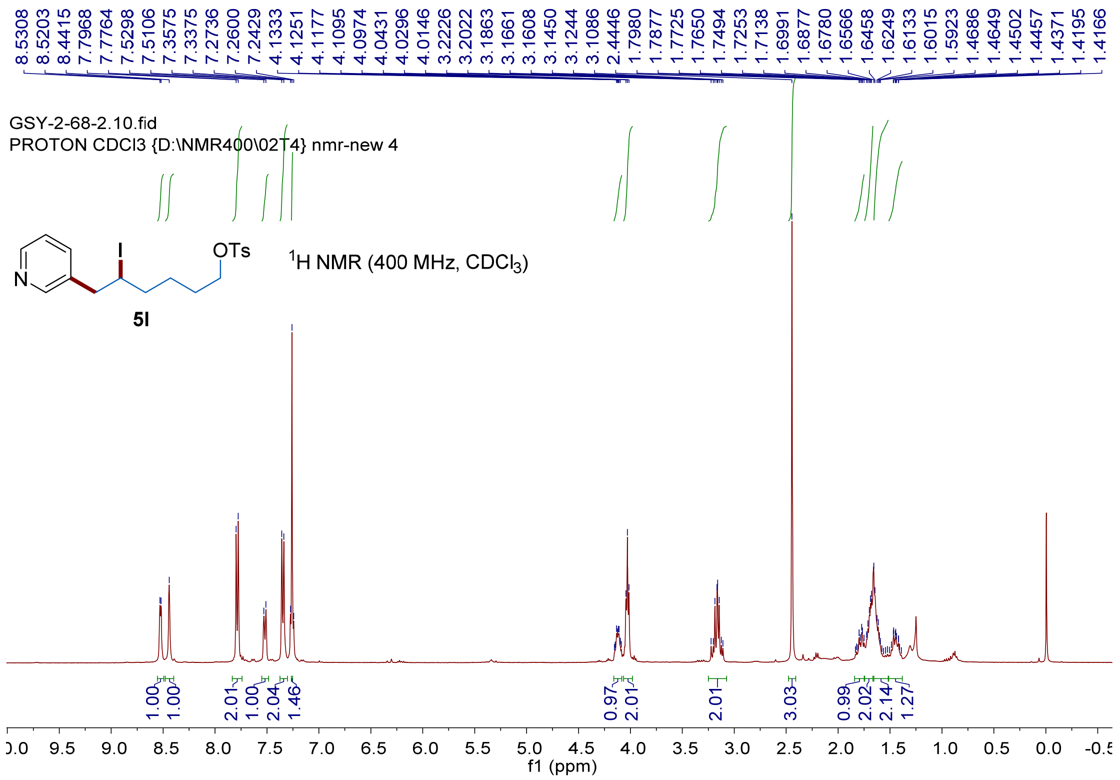

Supplementary Figure 118. ^{1}H NMR spectra of compound 5l

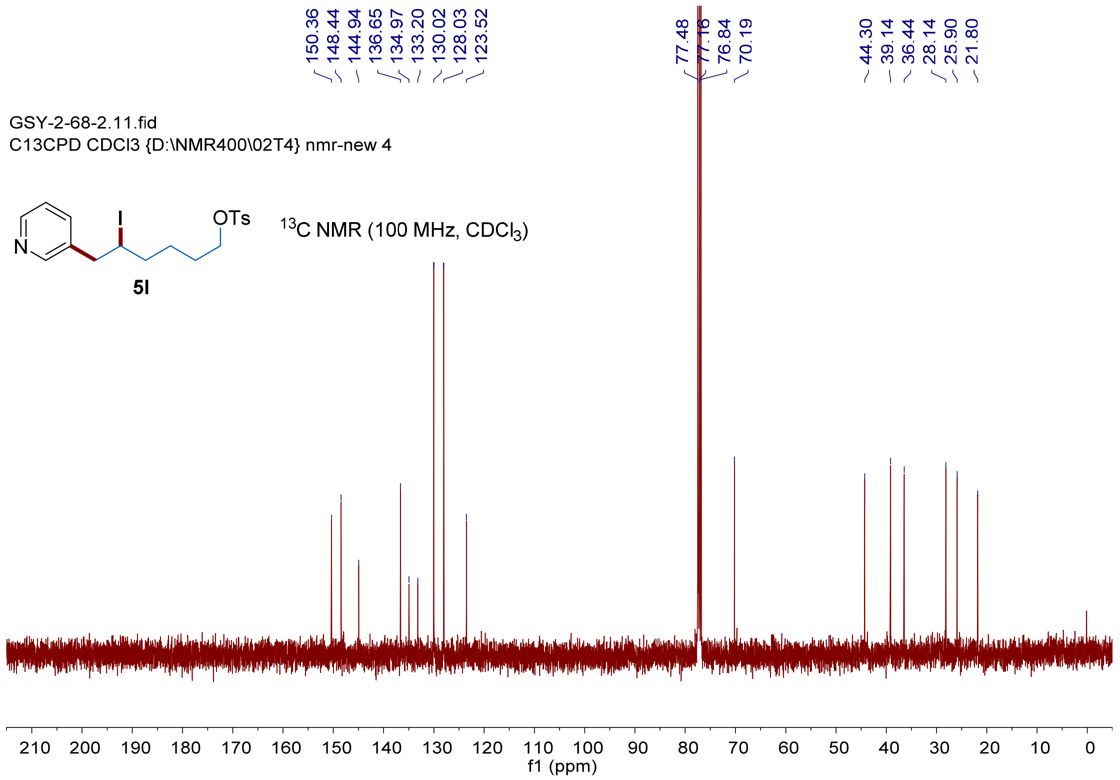

Supplementary Figure 119. ^{13}C NMR spectra of compound 5l

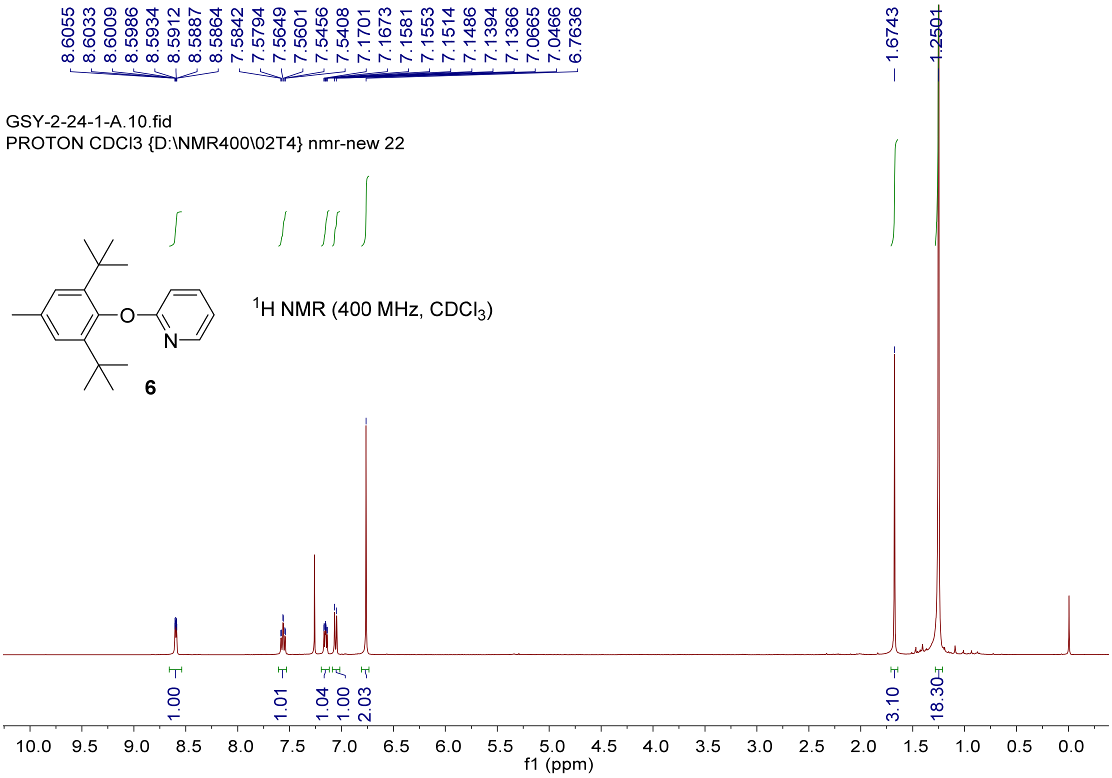

Supplementary Figure 120. ^{1}**H NMR spectra of compound 6**

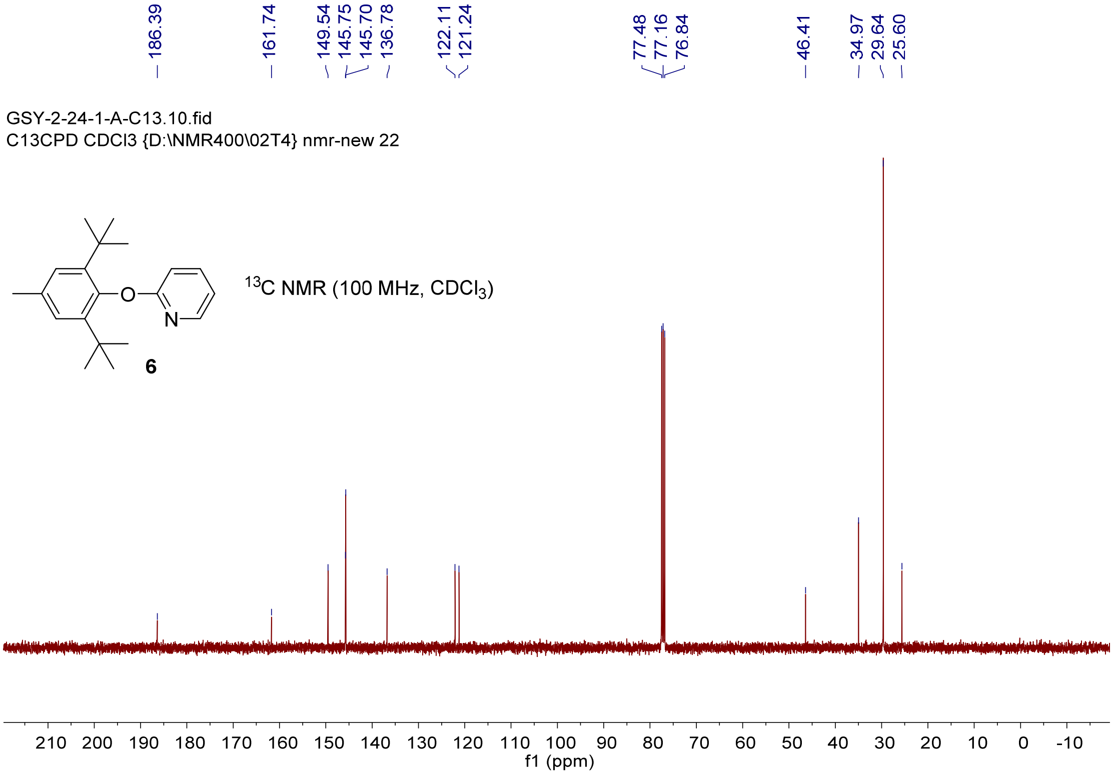

Supplementary Figure 121. ^{13}**C NMR spectra of compound 6**

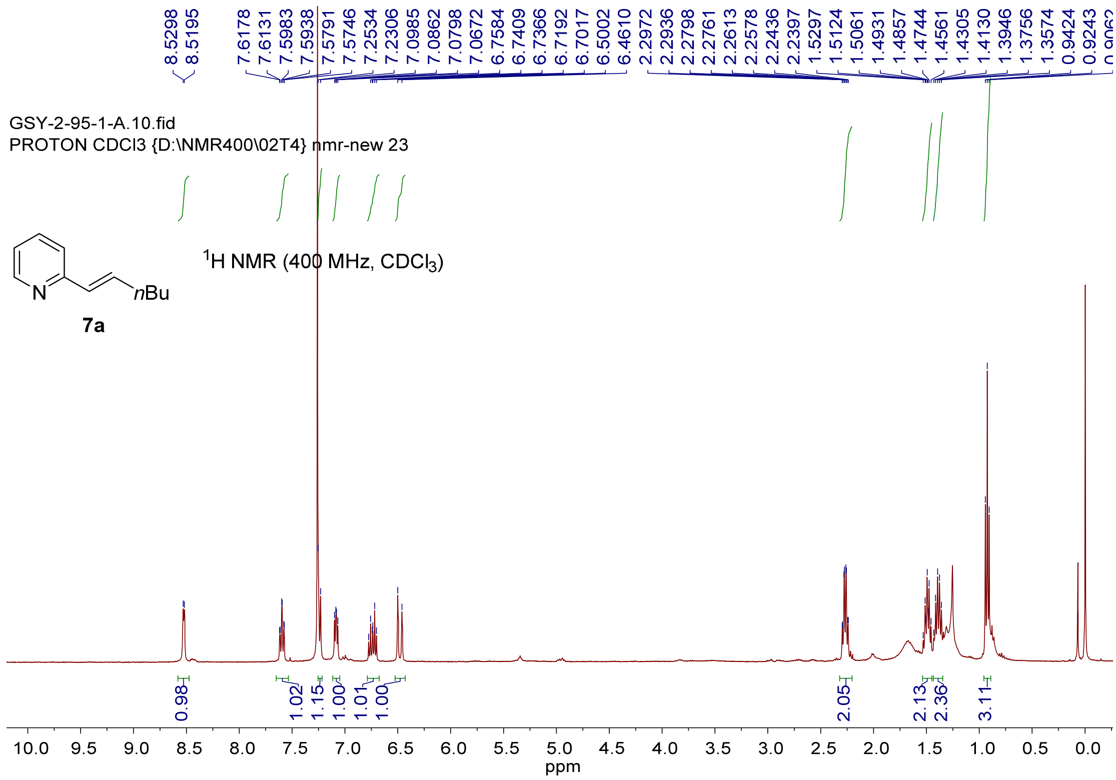

Supplementary Figure 122. ^{1}H NMR spectra of compound 7a

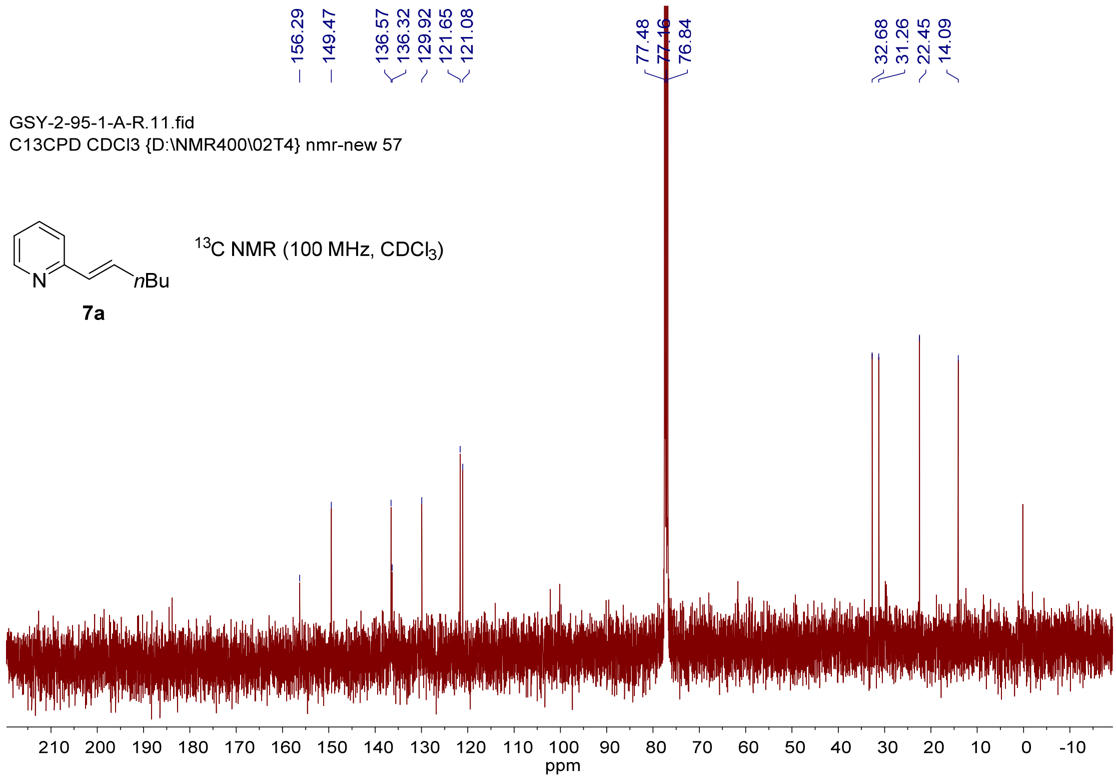

Supplementary Figure 123. ^{13}C NMR spectra of compound 7a

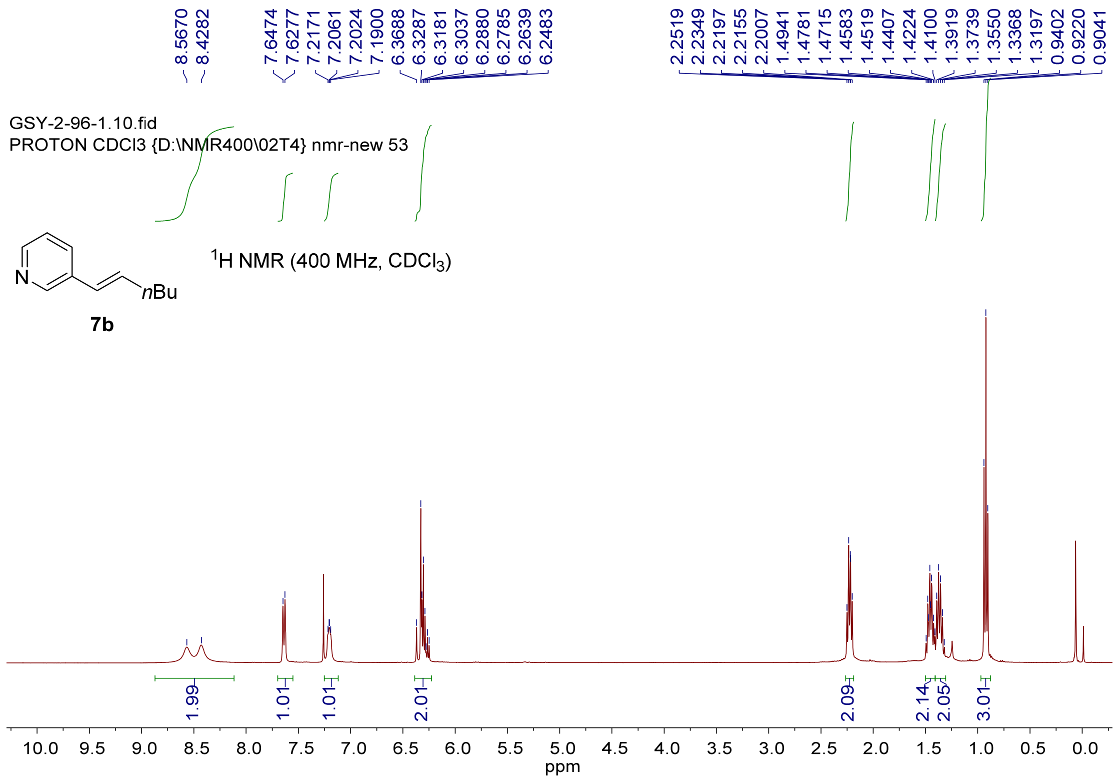

Supplementary Figure 124. ^{1}H NMR spectra of compound 7b

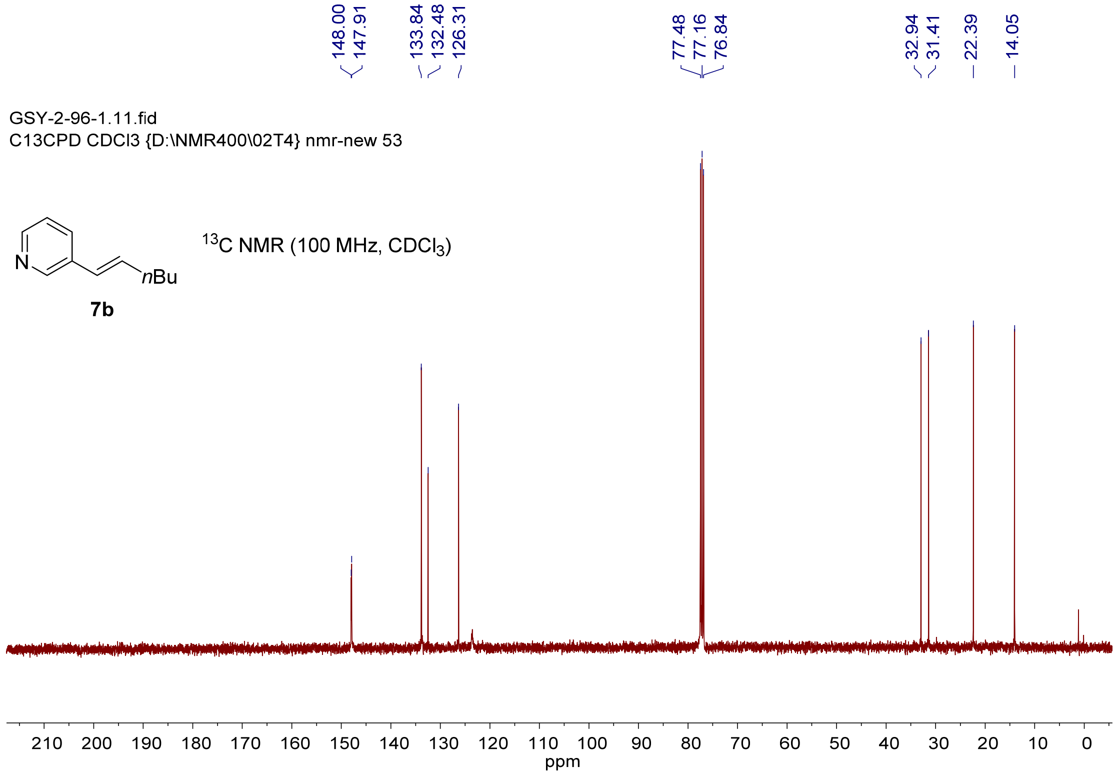

Supplementary Figure 125. ^{13}C NMR spectra of compound 7b

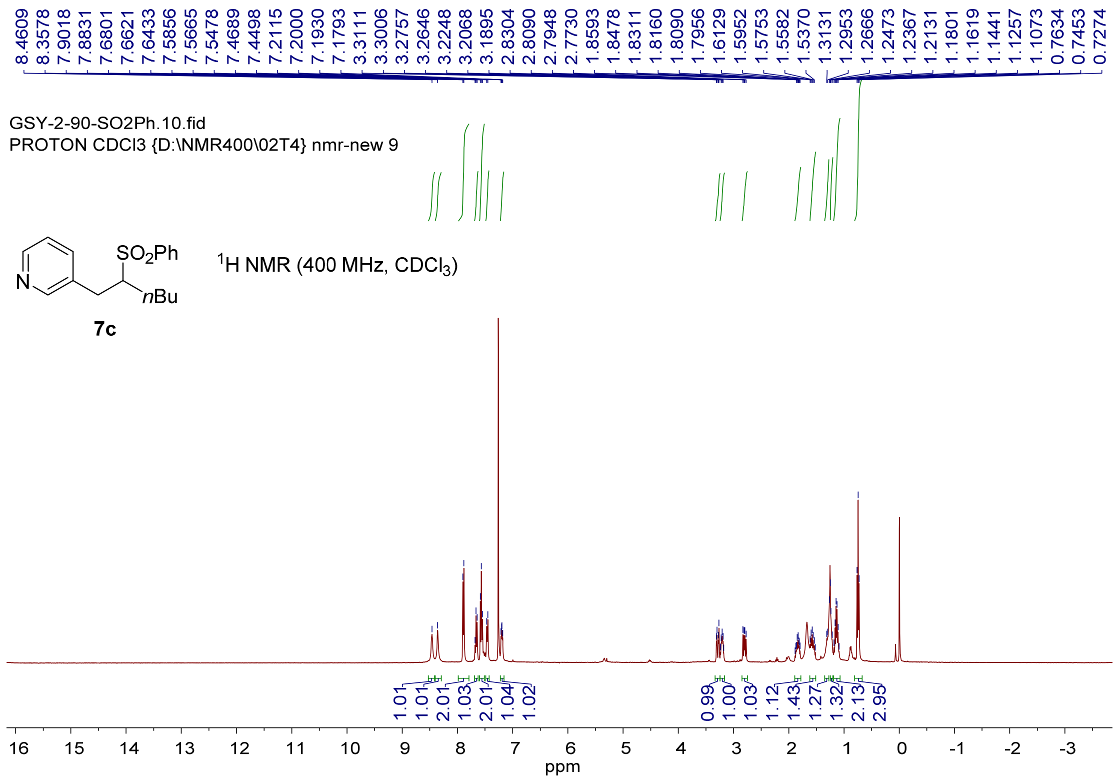

Supplementary Figure 126. ^{1}H NMR spectra of compound 7c

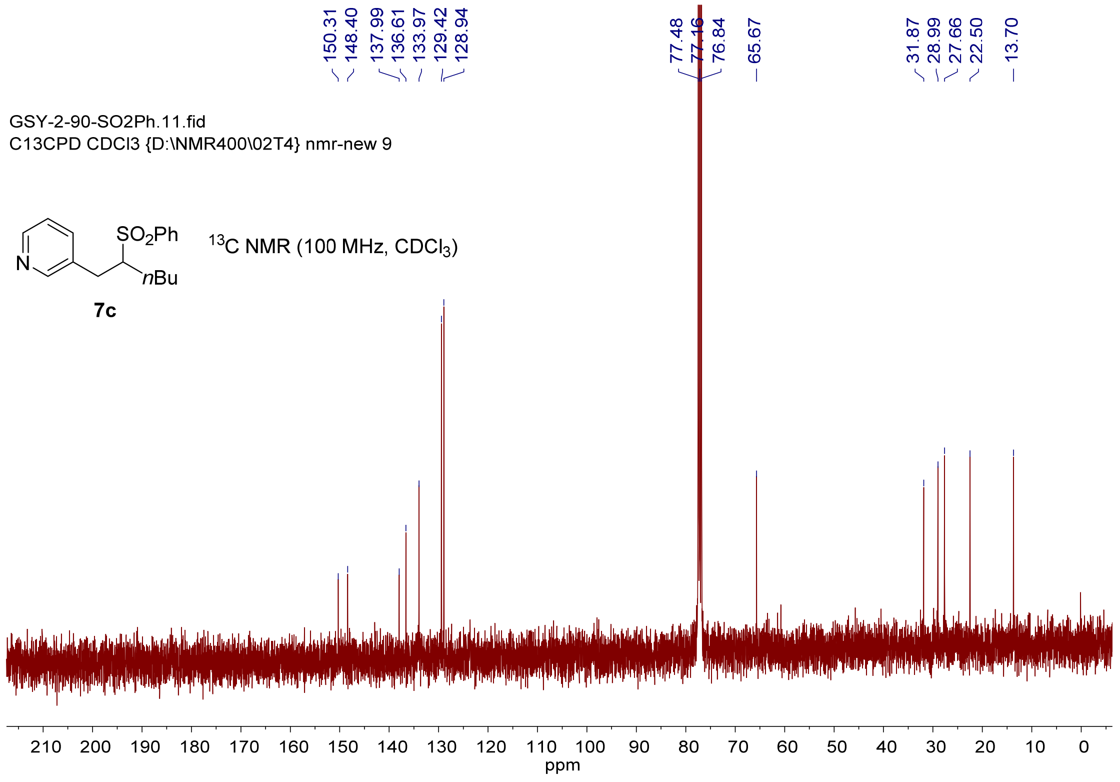

Supplementary Figure 127. ^{13}C NMR spectra of compound 7c

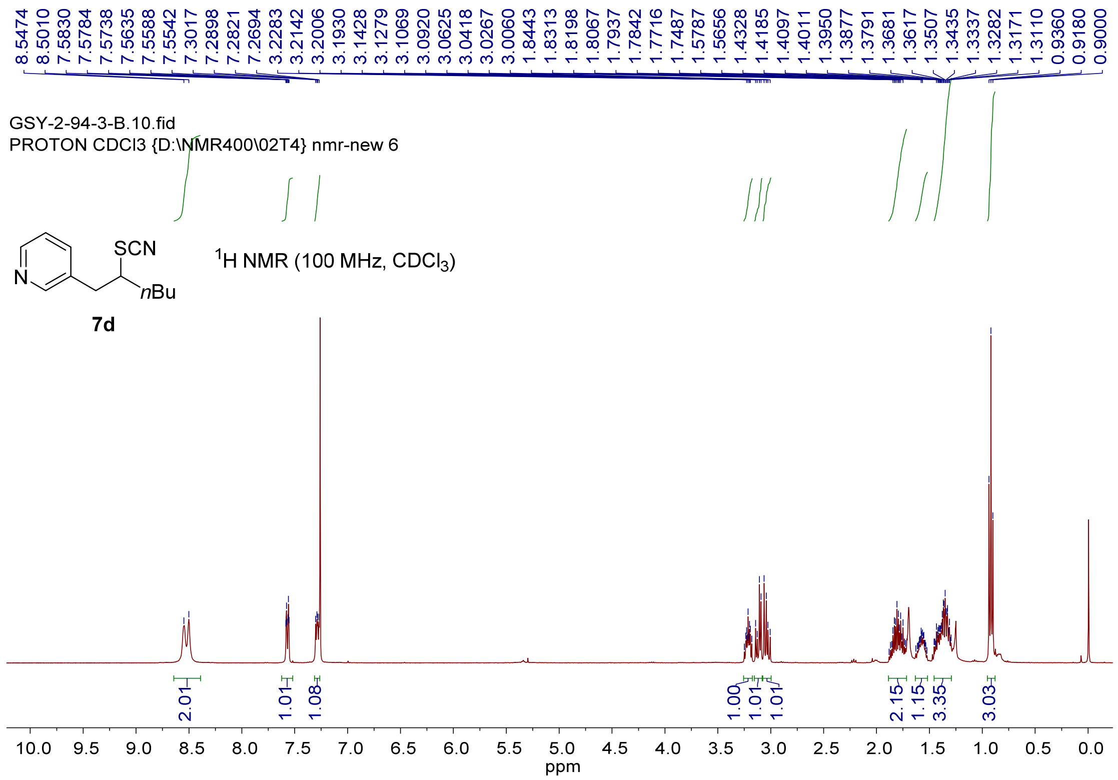

Supplementary Figure 128. ^{1}H NMR spectra of compound 7d

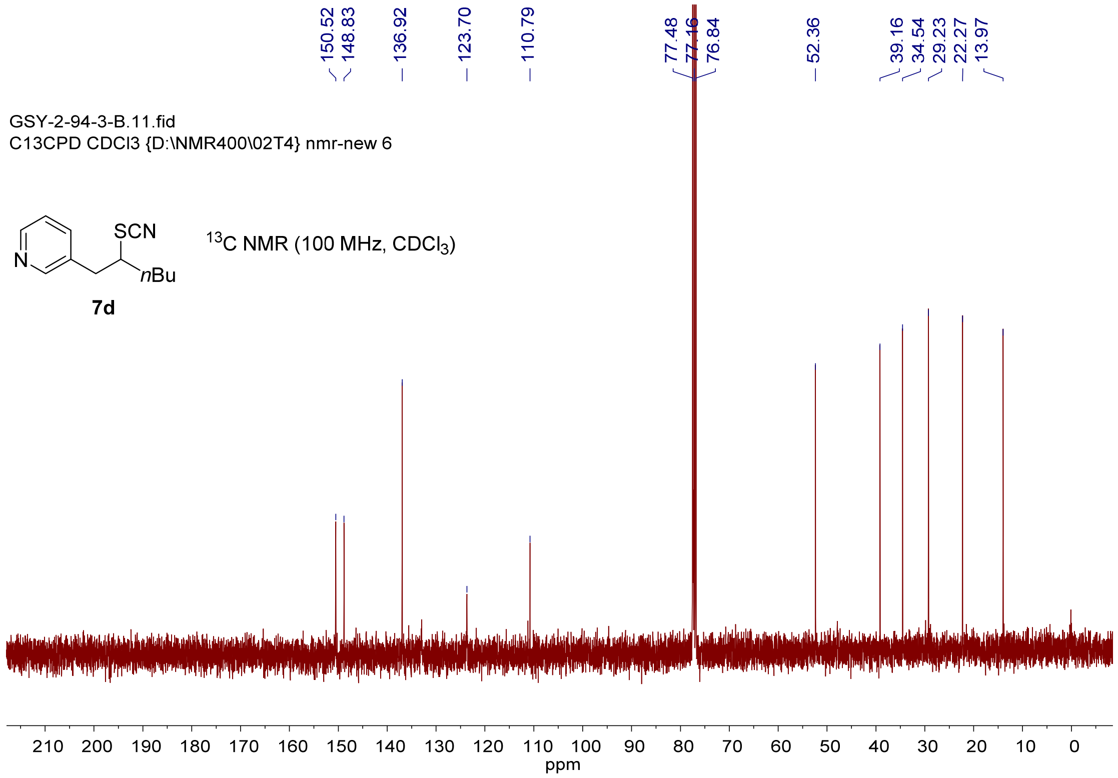

Supplementary Figure 129. ^{13}C NMR spectra of compound 7d

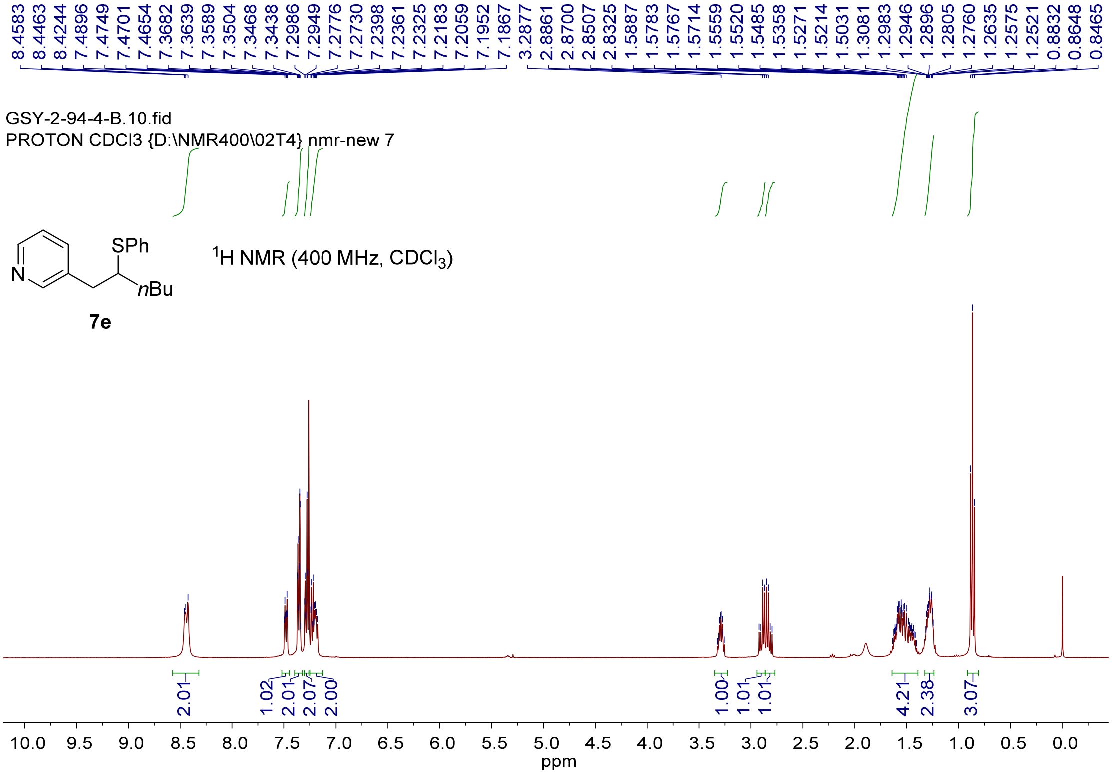

Supplementary Figure 130. ^{1}H NMR spectra of compound 7e

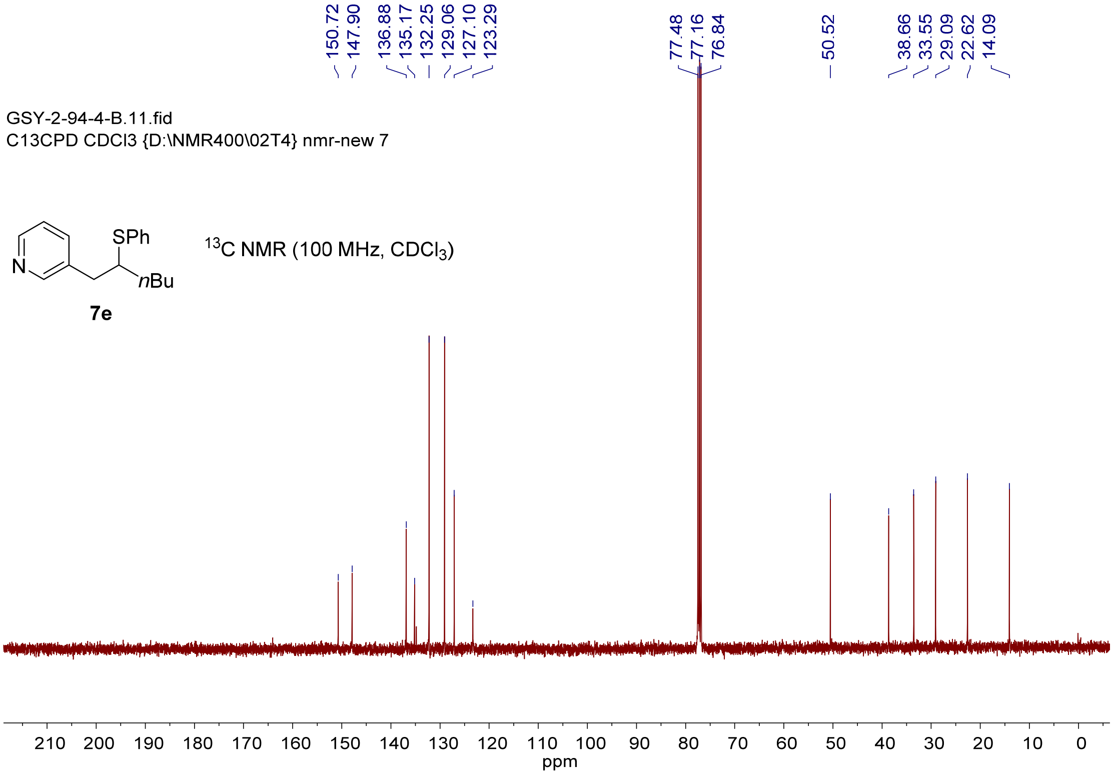

Supplementary Figure 131. ^{13}C NMR spectra of compound 7e

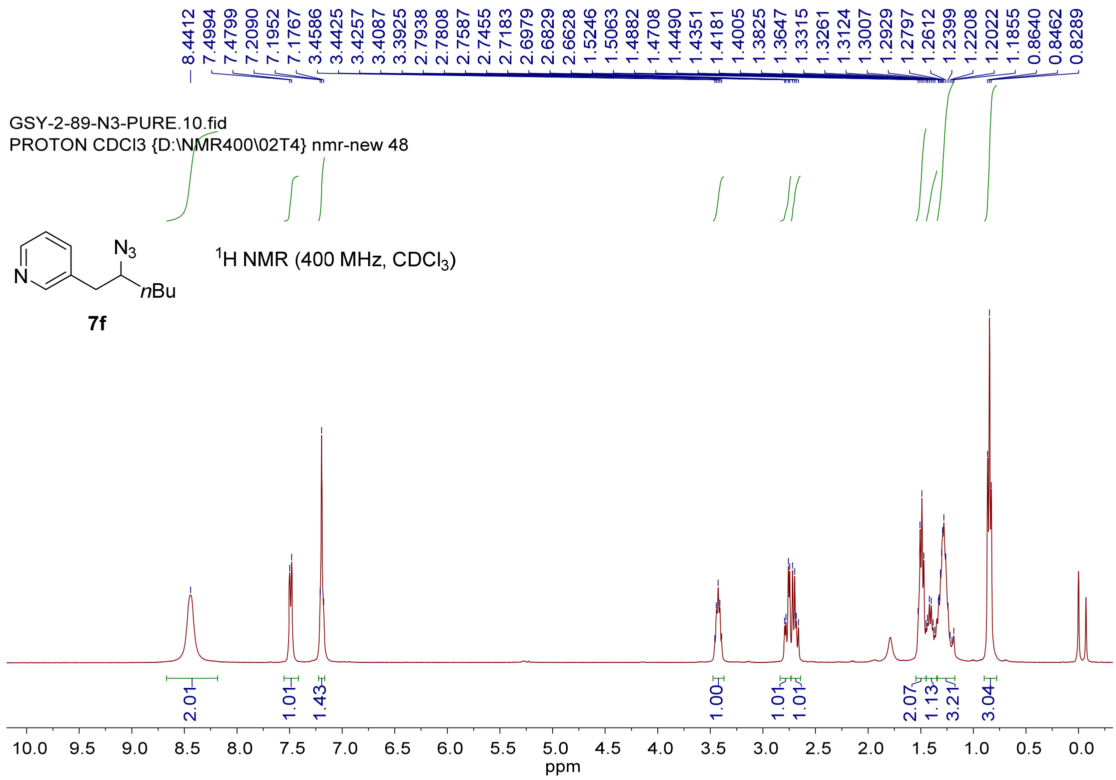

Supplementary Figure 132. ^{1}H NMR spectra of compound 7f

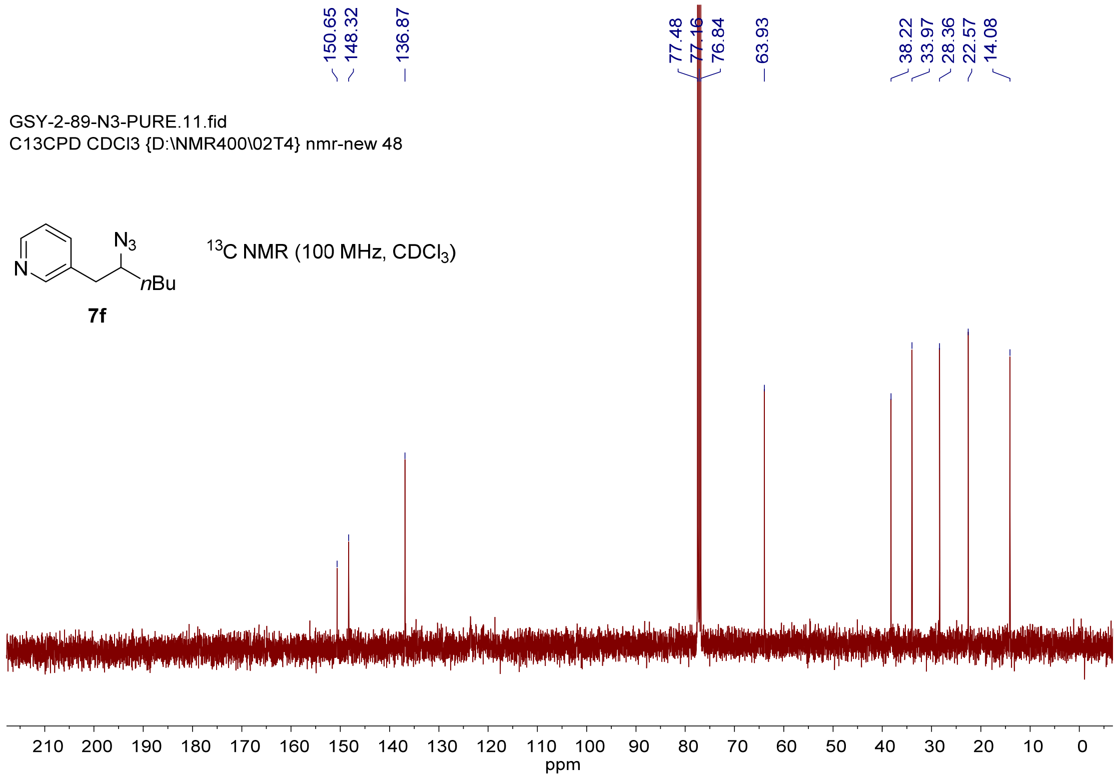

Supplementary Figure 133. ^{13}C NMR spectra of compound 7f

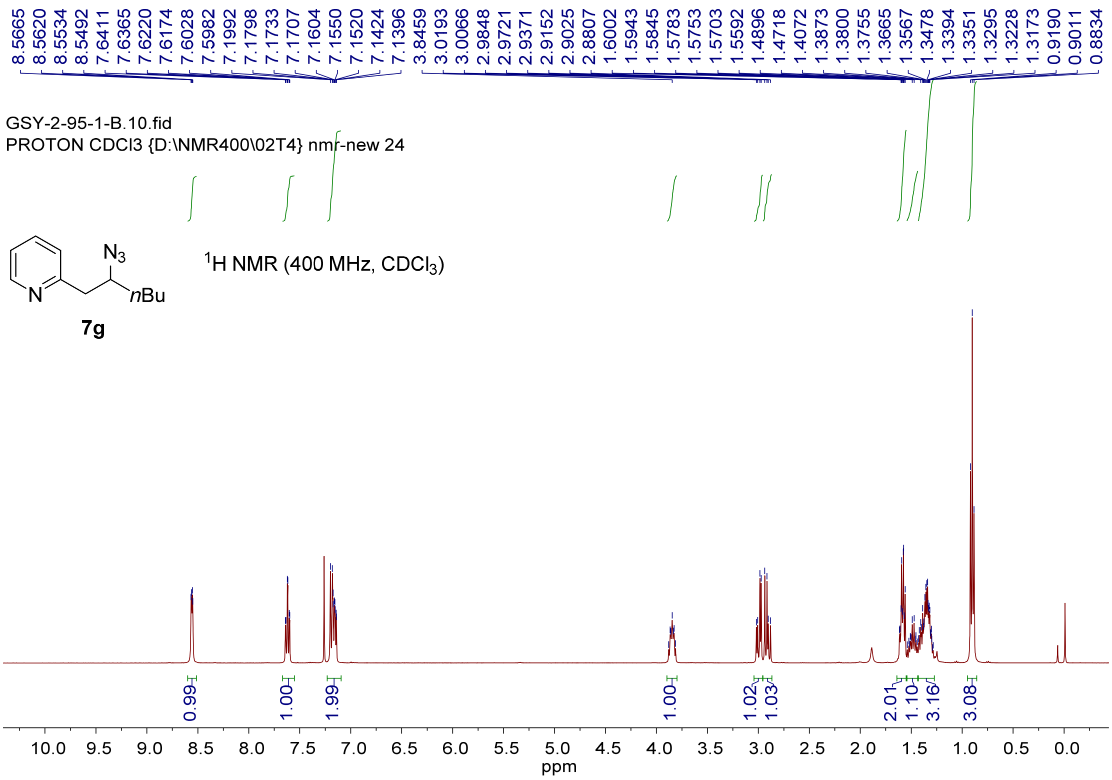

Supplementary Figure 134. ^{1}H NMR spectra of compound 7g

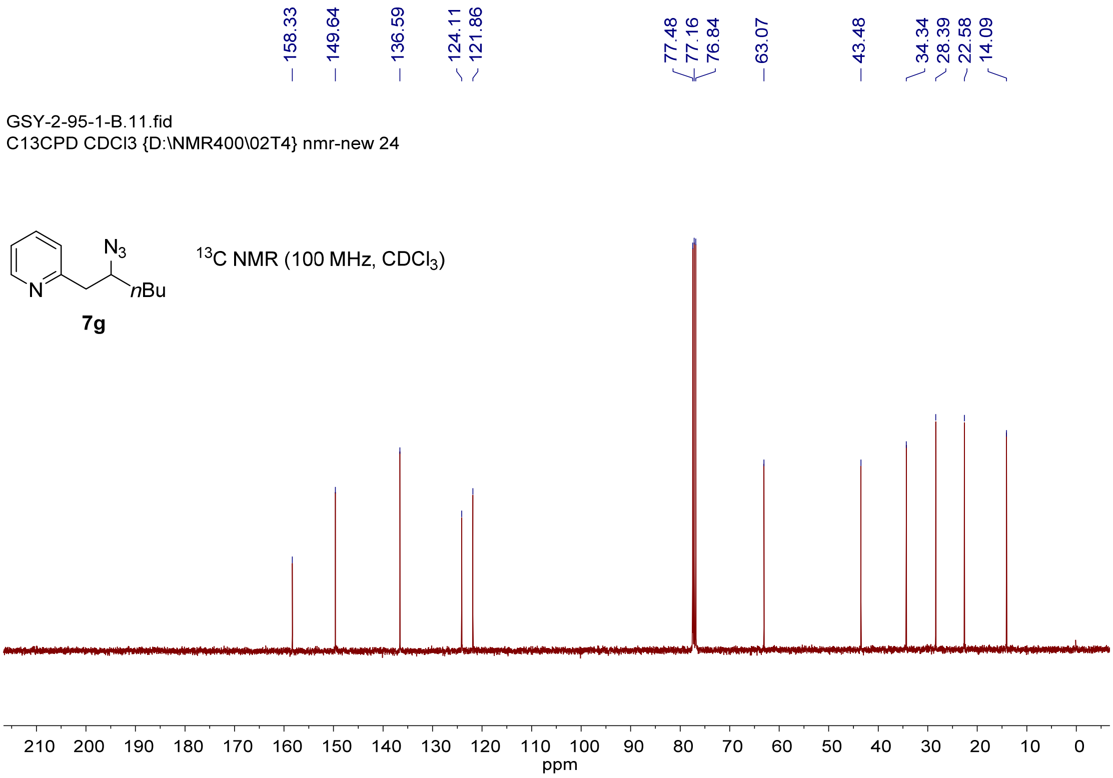

Supplementary Figure 135. ^{13}C NMR spectra of compound 7g

3. Supplementary References

[1] Cismesia, M. A. & Yoon, T. P. Characterizing chain processes in visible light photoredox catalysis. *Chem. Sci.* **6**, 5426-5434 (2015).

[2] Kuhn, H. J., Braslavsky, S. E. & Schmidt, R. Chemical actinometry (IUPAC Technical Report). *Pure Appl. Chem.* **76**, 2105-2146 (2004).

[3] Demas, J. N., Bowman, W. D., Zalewski, E. F. & Velapoldi, R. A. Determination of the quantum yield of the ferrioxalate actinometer with electrically calibrated radiometers. *J. Phys. Chem.* **85**, 2766-2771 (1981).
